# A Cross-Session Dataset for Collaborative Brain-Computer Interfaces Based on Rapid Serial Visual Presentation

**DOI:** 10.3389/fnins.2020.579469

**Published:** 2020-10-22

**Authors:** Li Zheng, Sen Sun, Hongze Zhao, Weihua Pei, Hongda Chen, Xiaorong Gao, Lijian Zhang, Yijun Wang

**Affiliations:** ^1^State Key Laboratory on Integrated Optoelectronics, Institute of Semiconductors, Chinese Academy of Sciences, Beijing, China; ^2^School of Future Technology, University of Chinese Academy of Sciences, Beijing, China; ^3^Department of Control Engineering, School of Information Science and Engineering, East China University of Science and Technology, Shanghai, China; ^4^Department of Biomedical Engineering, School of Medicine, Tsinghua University, Beijing, China; ^5^Beijing Machine and Equipment Institute, Beijing, China

**Keywords:** brain-computer interfaces (BCI), rapid serial visual presentation (RSVP), collaborative BCI, cross-session transfer, event related potentials (ERP), electroencephalogram (EEG)

## Abstract

Brain-computer interfaces (BCIs) based on rapid serial visual presentation (RSVP) have been widely used to categorize target and non-target images. However, it is still a challenge to detect single-trial event related potentials (ERPs) from electroencephalography (EEG) signals. Besides, the variability of EEG signal over time may cause difficulties of calibration in long-term system use. Recently, collaborative BCIs have been proposed to improve the overall BCI performance by fusing brain activities acquired from multiple subjects. For both individual and collaborative BCIs, feature extraction and classification algorithms that can be transferred across sessions can significantly facilitate system calibration. Although open datasets are highly efficient for developing algorithms, currently there is still a lack of datasets for a collaborative RSVP-based BCI. This paper presents a cross-session EEG dataset of a collaborative RSVP-based BCI system from 14 subjects, who were divided into seven groups. In collaborative BCI experiments, two subjects did the same target image detection tasks synchronously. All subjects participated in the same experiment twice with an average interval of ∼23 days. The results in data evaluation indicate that adequate signal processing algorithms can greatly enhance the cross-session BCI performance in both individual and collaborative conditions. Besides, compared with individual BCIs, the collaborative methods that fuse information from multiple subjects obtain significantly improved BCI performance. This dataset can be used for developing more efficient algorithms to enhance performance and practicality of a collaborative RSVP-based BCI system.

## Introduction

Brain-computer interfaces (BCIs) establish a communication channel between human brain and the external world (Wolpaw et al., 2002; Gao et al., 2014). As one of the well-known BCI paradigms, rapid serial visual presentation (RSVP)-based BCIs have been usually used for target image detection. Although computer vision (CV) has become a major method to deal with the image recognition problem recently, it consumes a large amount of resource (image source, training time, computing power, etc.) to get a good performance, and is still lack of generalization ability. By contrast, human vision (HV) can achieve general purposes of object recognition. HV can cope with more difficult tasks and detect targets with different characteristics (e.g., scale, lighting, background, etc.; Mathan et al., 2008; Sajda et al., 2010; Pohlmeyer et al., 2011). Human visual system can recognize objects with just a glance (Oliva, 2005) and detect targets in under 150 ms (Thorpe et al., 1996). However, manual image analysis is slow because of the motor response time, and the variability of response time makes it difficult to locate the target images in RSVP tasks (Gerson et al., 2005; Mathan et al., 2008; Sajda et al., 2010). Therefore, RSVP-based BCIs, which have stronger generalization ability and are faster than behavioral response, have become a useful method to detect targets by using the human brain activities. By presenting multiple images sequentially in a high presentation rate (e.g., 10 images per second), the RSVP-based BCI can enhance the target detection performance of HV (Lees et al., 2018).

In earlier times, RSVP was often used to do behavioral research focusing on attentional blink (AB; Broadbent and Broadbent, 1987; Chun and Potter, 1995; Jolicoeur, 1998) and manual target detection (Lawrence, 1971; Broadbent and Broadbent, 1987). With the rapid development of computer technology and electroencephalography (EEG)-based BCIs, RSVP was introduced to design BCI systems for target detection. The RSVP-based BCI is realized by single-trial event related potential (ERP) detection. ERPs typically contain multiple components with different temporal and spatial characters. In an RSVP-based BCI system, the P300 component, which occurs approximately 300 ms after the target stimulation, is the major ERP component used for target detection (Picton, 1992; Chun and Potter, 1995). Since the system performance of RSVP-based BCIs can be influenced by many factors such as presentation rate (Sajda et al., 2003; Acqualagna et al., 2010; Lees et al., 2019), target probability (Cecotti et al., 2011), stimulus onset asynchrony and stimulus repetition (Cecotti et al., 2014a), image size (Rousselet et al., 2004; Serre et al., 2007), type of targets (Lees et al., 2019), saccadic eye movements (Bigdely-Shamlo et al., 2008), attention blink (Broadbent and Broadbent, 1987; Chun and Potter, 1995; Jolicoeur, 1998), and other subjective or objective factors (Jolicoeur, 1998; Acqualagna et al., 2010; Touryan et al., 2011), the experimental paradigm should be carefully designed.

Besides the design of system paradigm, the main challenge in RSVP-based BCIs is single-trial ERP detection. In the RSVP-based BCI system, multi-channel EEG recording leads to a high dimensionality of features, and the small number of trials is always not sufficient for solving the classification problem toward accurate ERP detection (Huang et al., 2011). To deal with the problem of single-trial ERP detection, suitable signal processing and classification algorithms are required to extract discriminative information from single-trial data and improve the performance in classifying target and non-target trials. Various algorithms have been proposed and developed for the RSVP-based BCIs (Lees et al., 2018; Lotte et al., 2018). Major feature extraction algorithms include xDAWN (Rivet et al., 2009), signal-to-noise ratio (SNR) maximizer for ERP (SIM; Wu and Gao, 2011), common spatial pattern (CSP; Ramoser et al., 2000), independent component analysis (ICA; Makeig et al., 1996), and etc. Typical classification algorithms include spatially weighted fisher’s linear discriminant (FLD)-principal component analysis [PCA; spatially weighted FLD-PCA (SWFP); Alpert et al., 2013], support vectors machine (SVM; Burges, 1998), linear discriminate analysis (LDA; Blankertz et al., 2011), hierarchical discriminant component analysis (HDCA; Sajda et al., 2010), convolutional neural network (CNN; Cecotti and Graser, 2010; Cecotti et al., 2014b), and etc. Since real targets can only appear once in the RSVP paradigm, averaging across multiple trials is not practical in the RSVP-based BCIs. By combining brain activities of multiple subjects, collaborative BCIs can improve the performance of single-trial ERP detection (Wang and Jung, 2011). A series of studies have demonstrated collaborative BCIs for target detection and decision making (Wang et al., 2011; Yuan et al., 2012; Matran-Fernandez et al., 2013; Cecotti and Rivet, 2014; Poli et al., 2014; Touyama, 2014; Valeriani et al., 2015, 2016, 2017; Bhattacharyya et al., 2019). For both individual and collaborative RSVP-based BCIs, system calibration remains another big challenge in practical applications. It has been claimed that high variability of EEG makes it difficult to transfer models across different sessions (Krauledat et al., 2008). Besides, the training session in system calibration is time-consuming and the system performance may probably decrease over time (Bigdely-Shamlo et al., 2008; Huang et al., 2011; Zhao et al., 2019). Therefore, it is of great significance to develop efficient algorithms to solve the cross-session classification problem in the RSVP-based BCIs.

Recently, open BCI datasets have pushed forward the development of data processing algorithms. However, there are very few freely available datasets for the RSVP-based BCIs (Acqualagna and Blankertz, 2013; Matran-Fernandez and Poli, 2017). To our knowledge, a benchmark dataset for collaborative RSVP-based BCIs is still missing. Besides, the existing datasets only provide data recorded from a single session, which is not suitable for studying the problem of cross-session transfer. This paper therefore presents a cross-session dataset for collaborative RSVP-based BCIs. The dataset has the following characteristics: (1) EEG data from two subjects were recorded simultaneously with a collaborative BCI where two subjects performed the same target detection tasks synchronously, (2) two separate sessions were recorded for each of seven groups (14 subjects) on two different days with an average interval of ∼23 days, and (3) whole-head 62-channel EEG data were recorded and the raw data were provided without further processing. Note that, all event triggers for target and non-target images were synchronously marked in the EEG data. Therefore, the data epochs extracted from both subjects could be precisely synchronized. During the experiments, subjects were asked to find target images with human in street images sequences presented at 10 Hz (10 images per second). The experiment included three blocks, and each block contained 14 trials. Each trial had 100 images, including 4 target images. In total, the dataset contains 84 blocks (1,176 trials) of data recorded from 14 subjects. The dataset can be especially useful for studying cross-session ERP detection algorithms for both individual and collaborative RSVP-based BCI systems.

The rest of this paper is organized as follows. Section “Methods” explains the experimental paradigm, data acquisition, the algorithms in data analysis, and the criterion in performance evaluation. Section “Data Record” describes the data record and other relevant information. Section “Data Evaluation” presents results of BCI performance in data evaluation. Section “Conclusion and Discussions” concludes and discusses future works.

## Methods

### Participants

Fourteen healthy subjects (10 females, mean age: 24.9 ± 1.5 years, all right-handed) with normal or corrected-to-normal vision participated in the experiments. The subjects were divided into seven groups with two subjects in each group. For each group, the experiments contained two sessions recorded on different days. For all groups, the average time interval between two sessions was ∼23 days. All subjects were asked to read and sign an informed consent form before the experiment. This study was approved by the Ethics Committee of Tsinghua University.

### Collaborative System

**FIGURE 1:**
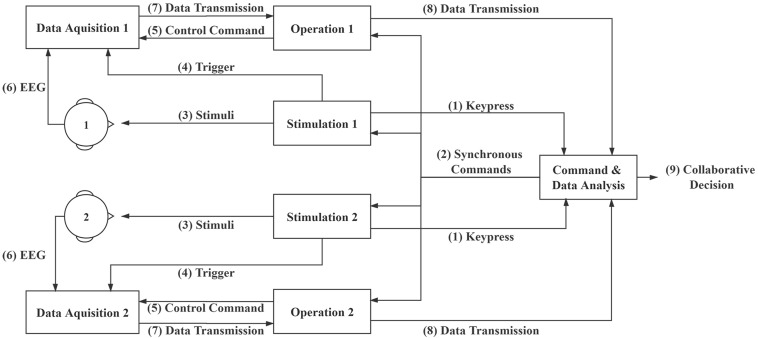
System diagram of the collaborative brain-computer interfaces (BCI) system.

Figure 1 illustrates the diagram of the online collaborative BCI system. The system consists of four major components: Stimulation module, Operation module, Data Acquisition module, and Command and Data Analysis module. The system performs the following steps: (1) The Command and Data Analysis module waits for keypress information from the Stimulation modules to start a trial; (2) The Command and Data Analysis module sends synchronous commands to the Operation and Stimulation modules; (3) The Stimulation modules present the RSVP stimuli to the subjects and (4) send event triggers to the Data Acquisition modules; (5) The Operation modules send control commands to the Data Acquisition modules and (6) record EEG data from the subjects; (7) The Operation modules receive EEG data from the Data Acquisition modules and (8) transfer to the Command and Data Analysis module; (9) The Command and Data Analysis module analyzes data and outputs online collaborative decisions. Data packages and commands are sent using transmission control protocol/internet protocol (TCP/IP) and triggers are sent using parallel ports. In the collaborative experiment, two subjects watched the same RSVP stimuli synchronously, and EEG data from them were fused to improve the overall detection performance. The same stimulations were presented to the two subjects using two separate computers. To synchronize EEG data from the two subjects, event triggers from the two stimulation computers were sent separately. The Command and Data Analysis module sent messages to synchronize the other modules. Therefore, although the Stimulation, Operation, and Data Acquisition modules were separated for each subject, the Command and Data analysis module fused the EEG data from two subjects and performed collaborative target detection in real time.

### Collaborative Experiment Design

**FIGURE 2:**
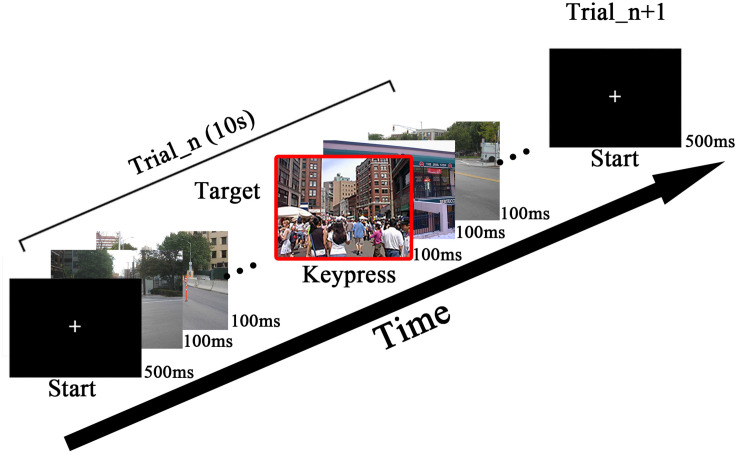
The overview of rapid serial visual presentation (RSVP) stimulation. The images are presented at 10 Hz. Subjects were asked to press the key immediately when finding a target. The sample of target is highlighted with a red frame.

The stimulation pattern of the RSVP paradigm is shown in Figure 2. The stimulation is presented by a 24.5-inch liquid crystal display (LCD) monitor with a resolution of 1,920 × 1,080 and a vertical refresh rate of 60 Hz. The images were downloaded from the internet. The stimulation was generated using the Psychophysics Toolbox Ver. 3 (PTB-3; Brainard, 1997). Street scene images were presented at 10 Hz (10 images per second) in the center of the screen within a 1,200 × 800-pixel square. The images containing human were regarded as target images.

The procedures of the collaborative experiments are depicted as follows. Subjects were asked to sit comfortably approximately 70 cm in front of the screen. When the subjects were ready, both of them were supposed to press keys to start one trial. The stimulation would not begin until both subjects pressed keys. If one subject pressed the key first, he or she had to wait for the second subject’s keypress to start the trial at the same time. After receiving two keypresses, the command module sent commands to the stimulation modules to start the same image sequence presentation synchronously to two subjects. As shown in Figure 2, a cross symbol appeared at the center of the screen for 500 ms to make subjects fix their sights, then the RSVP stimulation began. Each trial contained 100 images (10 s at the rate of 10 Hz), including four target images. The images shown in the first and last 1 s in one trial were all non-target images to avoid the target from appearing during the onset or offset of steady-state visual evoked potentials (SSVEP) evoked by RSVP. The interval of two target images was at least 500 ms to reduce the influence of the attention blink (Broadbent and Broadbent, 1987; Chun and Potter, 1995; Lees et al., 2018). Subjects were asked to press keys immediately after they detected a target. The keypress task was used to make subjects concentrate on target detection. Since there was a time delay between the target image and keypress, the keypress within 500ms after a target image was considered a correct response to the target image during the experiments. In the experiments, subjects needed to find four targets from 100 images and made four keystrokes. If the subjects missed some targets, the system would show the missed targets at the end of the trial. For the same group of subjects, the experiments included two sessions on different days, where the stimulation paradigms were totally same. The RSVP stimulation was presented in blocks. Each session consisted of three blocks and each block contained 14 trials (1,400 images, including 56 targets). Subjects were allowed to take a short rest after each block. During the experiment, the first block was used for training, while the second and the third blocks were used for testing. In the testing blocks, online classification results were provided by the Command and Data Analysis module. The online visual feedback was a 3 × 3 image matrix including nine images with the highest scores among the 100 images in each trial.

### Data Acquisition

The EEG data from two subjects were simultaneously recorded by two Neuroscan Synamps2 systems. 64-electrode EEG caps based on the 10–20 system were used to record 62-channel EEG data (M1 and M2 were not used) from two subjects. The reference electrode was at the vertex. The impedances of the electrodes were kept under 10 kΩ. The sample rate was 1,000 Hz. A notch filter at 50 Hz was used to remove the common power-line noise. The pass-band of the amplifier was set to 0.15–200 Hz. All the event triggers were transmitted and marked on the EEG data by parallel ports. Two stimulation computers sent triggers separately to the two EEG systems. The dataset provides raw data from the experiments without any processing.

### Data Preprocessing

To validate the quality of the data through performance evaluation, data preprocessing was performed as follows. The EEG data were first down-sampled to 250 Hz. After that, epochs corresponding to all images were extracted according to the event triggers. Each epoch began at 0.2 second before the event trigger, and ended at 1 second after the event trigger. The epochs were band-pass filtered within 2–30 Hz. For the analysis of EEG characteristics, the EEG data were re-referenced to the average of all electrodes [i.e., common average reference (CAR)], and the ERP waveforms were plotted using data at Cz. For performance evaluation, the time window 0–500 ms after the event trigger of each epoch was extracted for feature extraction and classification.

### Data Analysis

#### Individual Data Analysis

In this paper, several existing algorithms were utilized for feature extraction and classification. The HDCA algorithm, which can extract both spatial and temporal features, has been widely used in the RSVP-based BCIs (Lees et al., 2018; Zhao et al., 2019; Sajda et al., 2010). In our previous study, the combination of SIM and HDCA was employed to deal with the cross-session transfer problem (Zhao et al., 2019). SIM can extract the EEG components that maximize the SNR of ERPs (Wu and Gao, 2011). In this paper, several other feature extraction algorithms including CSP, task-related component analysis (TRCA), and PCA whitening were employed for comparison. CSP can build a spatial filter to extract features from two classes toward the best discrimination (Ramoser et al., 2000). TRCA is a method to extract task-related components by maximizing the reproducibility of repetitive tasks (Nakanishi et al., 2018). PCA whitening is usually used before ICA to reduce the complexity of the classification problem (Hyvärinen and Oja, 2000). To estimate performance for each subject, the first block of data was used for training and the other two blocks were used for testing.

#### Collaborative Data Analysis

**FIGURE 3:**
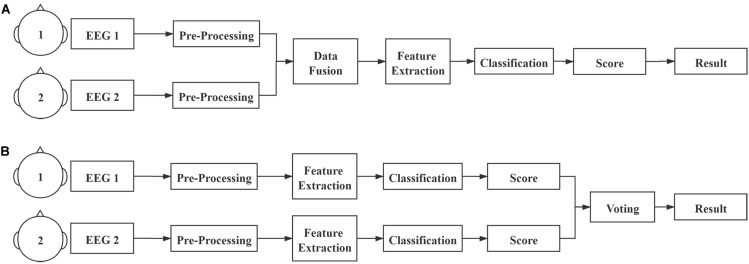
**(A)** Centralized and **(B)** distributed diagrams of collaborative data analysis.

The diagrams of collaborative data analysis are depicted in Figure 3. For the collaborative experiments, the EEG data of two subjects were fused by three methods: ERP averaging, feature concatenating, and voting (Wang and Jung, 2011). ERP averaging and feature concatenating are centralized methods, which fuse the data before further feature extraction and classification algorithms. Voting is a distributed method, which analyzes data of each subject first and then fuses the scores generated by the individual classifiers. In the ERP averaging method, the synchronous data epochs of two subjects were averaged. In the feature concatenating method, data epochs of two subjects were concatenated for further analysis. In the voting method, the weighted sum of the output scores of the classifiers of two subjects were used for classification, and the weights were the performance [i.e., area under curve (AUC)] of each subject from the training procedure. During the experiments, the online feedback, which consisted of nine images with the highest output scores, was calculated using the voting method.

#### Cross-Session Data Analysis

For the cross-session data analysis, the algorithms used for evaluation were the same as the separate experiments. However, the number of components extracted by the feature extraction algorithms (e.g., spatial filtering methods such as CSP, TRCA, and SIM) was optimized separately for each algorithm. The number of components can influence the cross-session performance because of the cross-session variability of EEG data. To estimate the cross-session performance, the first block of data on Day 1 was used for training and the second and third blocks on Day 2 were used for testing. The validation strategy was the same for individual and collaborative data analysis.

### Metric

This paper used the area under receiver operating characteristic (ROC) to evaluate the BCI performance. This metric is suitable for the RSVP paradigm where the class distribution is unbalanced (Lees et al., 2018). AUC can reflect the relationship between true positive rate (TPR) and false positive rate (FPR). In the RSVP-based BCI system, higher AUC indicates better performance.

## Data Record

### EEG Data

The dataset is freely available at https://doi.org/10.6084/m9.figshare.12824771.v1. The dataset is about 6.58 GB including collaborative and cross-session data from 14 subjects. All data are saved as MATLAB MAT files. The sample rate is 1,000 Hz and all data are raw data without any processing. Each file is named as “Group index + Session index” (i.e., G1D1.mat, G1D2.mat, …, G7D2.mat). “G*n*” is the *n*th group (totally seven). “D1” and “D2” indicate the first and second sessions respectively. Each file contains two cells named “Sa” and “Sb” indicating two subjects in the group. Each 1 × 3 cell array (“Sa” and “Sb”) contains three blocks of data recorded in one session. Each element in the cell array corresponds to one block of data. Each element is a matrix with a dimension of [63, *N*], which indicates 62-channel EEG data and a trigger channel with a length of *N*. *N* of each matrix is different because of the different experiment duration, but *N* of a group of subjects in the same block is the same. For the trigger channel, the onset of target image is defined as “1” and the onset of non-target image is defined as “2.” Since each element corresponds to one block, each matrix contains data of 14 trials (1,400 image events, including 56 targets). Details of data information are also summarized in a “Readme.txt” file.

### Supplementary Information

Three supplementary files are provided including subject information and channel location, and the image set. Subject information is saved in a “sub_info.txt” file, which includes the gender, age, handedness, group, and the interval between the two sessions. Channel locations are saved in a “62-channels.loc” file, in which the information for each channel consists of four columns: channel index, degree, radius, and label. The origin is at Cz (i.e., the radius is 0). The image set used for RSVP stimulation is also included in the supplementary files and saved in a “Image.zip” file. Target and non-target images are saved in two folders.

## Data Evaluation

### Individual BCI Performance

#### Within-Session Individual Performance

**FIGURE 4:**
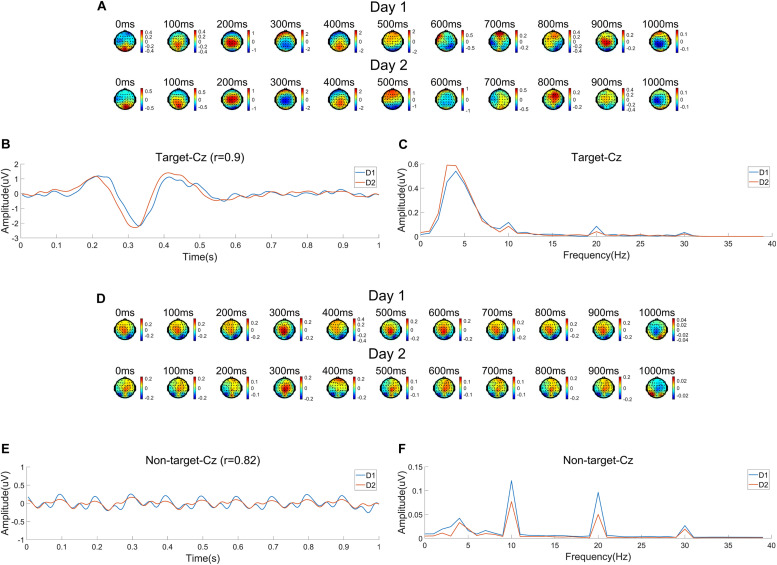
Electroencephalogram (EEG) characteristics averaged across subjects corresponding to two sessions (Day 1 and Day 2). **(A)** Time course of topographic maps of average event related potentials (ERPs) related to target images. **(B)** Average ERP waveform for targets at Cz. **(C)** Spectrum of ERPs for targets at Cz. **(D)** Time course of topographic maps of average EEG waveforms related to non-target images. **(E)** Average EEG waveform for non-target images at Cz. **(F)** Spectrum of the average EEG waveform for non-target images at Cz.

Figure 4 shows the EEG characteristics on Day 1 (D1) and Day 2 (D2) related to targets and non-targets using the average data of all subjects. Figure 4A shows the time course of scalp map series of average ERP amplitudes. It is clearly shown that the P300 component peaked around 400 ms and was mainly distributed at the central-parietal areas. Figure 4B shows the average ERP waveform at Cz. The waveform shows large N2 and P3 components after the target onset, which are obviously higher than other ERP components. Figure 4C shows the spectrum of ERPs at Cz calculated by fast Fourier transform (FFT). The EEG power mainly focuses at a low frequency range under 10 Hz with a peak around 4 Hz. It should be noted that there are frequency peaks at 10 Hz and its harmonic frequencies, which means there are SSVEP components in ERPs. Figures 4D–F show the EEG characteristics related to the non-target images. Figure 4D shows the average topographic map series, which indicate significant distribution of SSVEPs mainly focused at the occipital area. The average EEG waveform at Cz in Figure 4E indicates strong SSVEP components evoked by the 10 Hz RSVP stimulation. Figure 4F shows the spectrum of the average EEG waveform at Cz with peaks at 10 Hz and its harmonic frequencies. In summary, EEG signals are different when subjects watch target and non-target images. During the RSVP task, SSVEP components are dominant when there are no targets, while the ERP components (i.e., N2 and P3) are evoked when detecting a target. For target images, the amplitude of ERPs are significantly higher than SSVEPs (Figure 4C). The amplitudes of SSVEPs for target and non-target images are close (Figures 4C,F).

**FIGURE 5:**
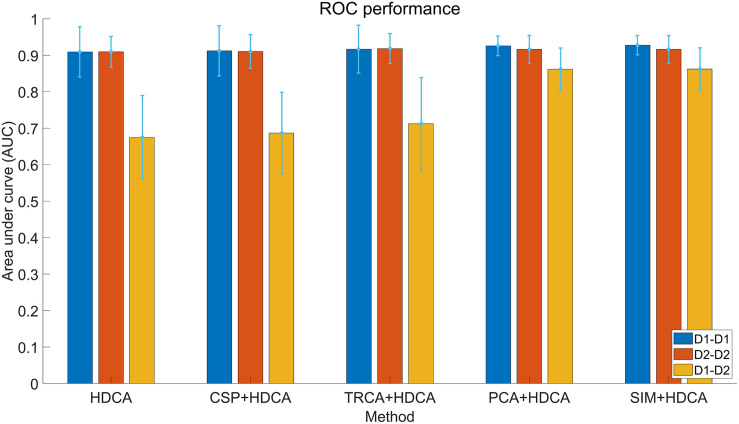
Receiver operating characteristic (ROC) performance of different feature extraction and classification algorithms in cross-session analysis with individual classification. The color bars of each method indicate performance of within-session (Day 1 and Day 2) and cross-session classification. The error bars indicate standard deviations.

The within-session BCI performance of individual classification is illustrated in Figure 5. For each subject, the first block of data is used for training and the other two blocks are used for testing. Four feature extraction methods are combined with HDCA for comparison: (1) CSP, (2) TRCA, (3) PCA, and (4) SIM. Because the number of components (i.e., the number of spatial filters) can influence the performance, all the component numbers are calculated, and the number of components with the maximum AUC values is shown in Figure 5. For each feature extraction method, the numbers of used components in the within-session conditions (D1–D1, D2–D2) and the cross-session condition (D1–D2) are different (CSP: 50, 50, 13; TRCA: 7, 3, 3; PCA: 24, 36, 14; SIM: 24, 36, 21). For the within-session classification, the combination methods cannot outperform the standard HDCA algorithm. Feature extraction algorithms can help to enhance the average performance (e.g., max ΔAUC = 0.018 when using the SIM + HDCA method on Day 1), but the improvement is not substantial.

#### Cross-Session Individual Performance

The cross-session variances of EEG are illustrated in Figure 4. In Figure 4A, ERP scalp map series for the two sessions (Day 1 and Day 2) show similar spatial and temporal trends, but also indicate different amplitudes and spatial distributions. In Figure 4B, differences in amplitudes and latencies of ERP components (i.e., N2 and P3) can be observed. The correlation of two average ERP waveforms in the two sessions still obtains a high correlation coefficient of 0.90. The spectral distributions shown in Figure 4C are consistent for the two sessions. The cross-session differences of SSVEPs related to the non-target images can be observed in Figures 4D–F. There are cross-session differences in terms of spatial distributions, amplitudes and latencies. The correlation coefficient of waveforms is 0.82. Although peaks of the spectra in both sessions are at 10 Hz and its harmonic frequencies, the peak amplitudes in the two sessions seem different in Figure 4F. The consistency of ERP characteristics across sessions suggests it is possible to transfer information from a previous session to facilitate system calibration.

**FIGURE 6:**
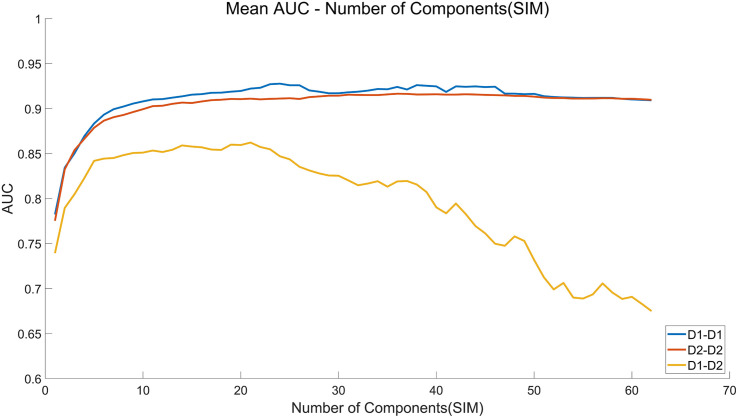
Number of components-area under curve (AUC) curve of the SIM + hierarchical discriminant component analysis (HDCA) algorithm in cross-session individual data analysis. The *x* axis indicates the number of components selected from the spatial filters calculated by the SIM algorithm.

**FIGURE 7:**
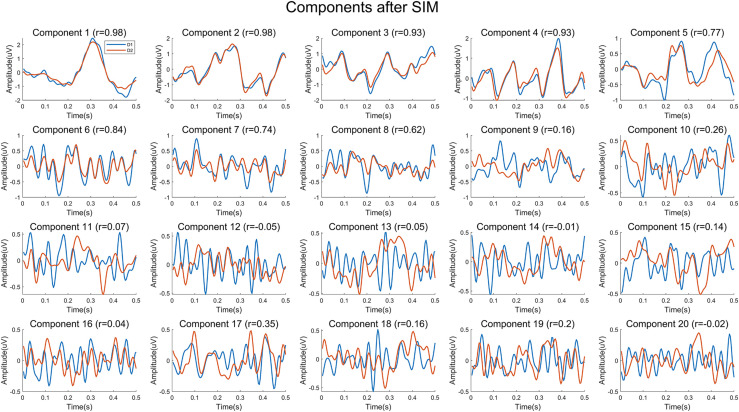
Average waveforms of components extracted by the SIM algorithm of one subject. The waveforms indicate the 1st–20th components of Day 1 and Day 2 extracted by the spatial filters obtained from data on Day 1.

The cross-session BCI performance of individual experiments is illustrated in Figure 5. Compared with the within-session performance, the cross-session performance decreases sharply due to the non-stationarity of EEG over time. For example, the AUC for HDCA decreases from 0.90 (Day 1 and Day 2) to 0.67. As described above, feature extraction algorithms do not improve the within-session performance, but the PCA and SIM algorithms significantly improve the cross-session classification performance. As shown in Figure 5, AUC values of the SIM + HDCA (0.86 ± 0.06) and PCA + HDCA (0.86 ± 0.06) methods are significantly better than HDCA (0.67 ± 0.11), CSP + HDCA (0.69 ± 0.11) and TRCA + HDCA (0.71 ± 0.13; *p* < 0.001). There is no significant difference between SIM + HDCA and PCA + HDCA (*p* > 0.05). Figure 6 shows an example of the relationship between AUC and the number of components when using the combination of SIM and HDCA algorithms. For the within-session condition, AUC increases as the number of components in SIM increases until AUC saturates when the number of components increases to about 30. However, for the cross-session condition, AUC first increases from 0.74 with 1 component, reaches a peak value of 0.87 with 21 components, and then decreases to 0.67 with 62 components. This finding indicates that not all spatial filters are suitable for cross-session transfer. Figure 7 shows averaged waveforms of the 1st–20th components from one subject extracted by SIM in the cross-session condition. It is clear that the first several components show strong ERP components. When the spatial filters obtained from Day 1 are directly applied to Day 2, the first several components have high cross-session correlation coefficients. For example, for the 1st–5th components, the correlation coefficients are 0.98, 0.98, 0.93, 0.93, and 0.73 respectively. However, the correlation coefficients decrease at the latter components (e.g., the correlation coefficients are less than 0.5 for the 9th–20th components). Therefore, the first several components that show stable ERP characters in both sessions contribute most to the cross-session classification. The involvement of latter components that exhibit large difference between two sessions cannot improve the classification performance. On the contrary, the increase of feature dimension might increase the risk of overfitting and thereby deteriorates the cross-session performance.

### Collaborative BCI Performance

#### Within-Session Collaborative Performance

**FIGURE 8:**
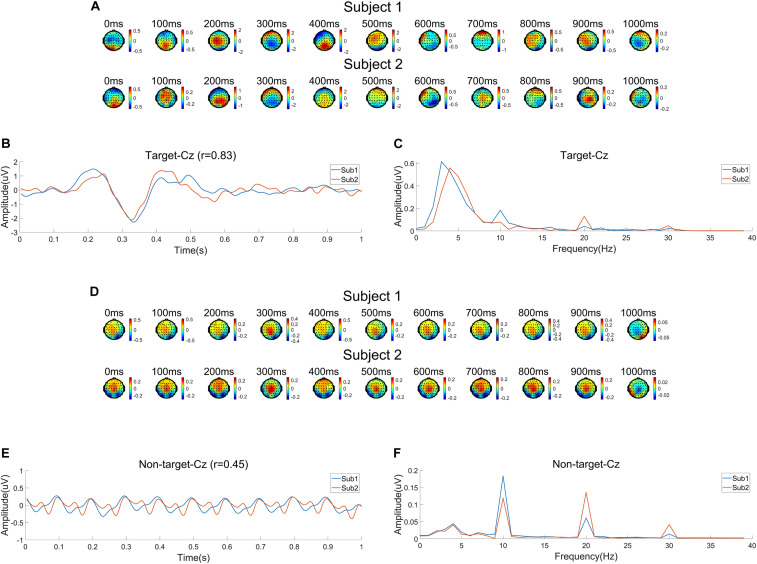
Electroencephalogram (EEG) characteristics for a group of subjects (Subject 1 and Subject 2) on Day 1. **(A)** Time course of topographic maps of average event related potentials (ERPs) related to target images. **(B)** Average ERP waveform for targets at Cz. **(C)** Spectrum of ERPs for targets at Cz. **(D)** Time course of topographic maps of average EEG waveform related to non-target images. **(E)** Average EEG waveform for non-target images at Cz. **(F)** Spectrum of the average EEG waveform for non-target images at Cz.

Example data recorded on Day 1 from subject 1 (Sub1) and subject 2 (Sub2) are used to analyze the EEG features for different subjects. The preprocessing procedures were the same as those in Figure 4. Figure 8 illustrates the common and different characters of EEG signals for Sub1 and Sub2. Specifically, Figures 8A–C show the ERP characteristics related to target images. As shown in Figure 8A, the time courses of topographic map series for both subjects show generally similar patterns. During about 200–400 ms after the target onset, N2 and P3 components are dominant over the central-parietal areas. There is clear difference in amplitudes and latencies for ERP waveforms at Cz (Figure 8B), which leads to a correlation coefficient of 0.83. Figure 8C shows the spectral distributions of ERPs at Cz. EEG powers for the two subjects are mainly under 10 Hz with slight difference, and the amplitudes of SSVEP components are very different. Figures 8D–F show the EEG characteristics related to non-target images. For SSVEP components evoked by non-target images, individual difference can be observed regarding to scalp topographic maps, amplitudes and latencies, as well as spectral distributions. As shown in Figure 8E, the correlation coefficient of EEG waveforms is 0.45. The low correlation can be attributed to the amplitude and latency difference of the fundamental and harmonic SSVEP components shown in Figures 8E,F. These results suggest that, as expected, the collaborative classification will improve the individual classification by fusing useful information from multiple subjects. However, the individual difference should be carefully considered in designing the data fusion method.

**FIGURE 9:**
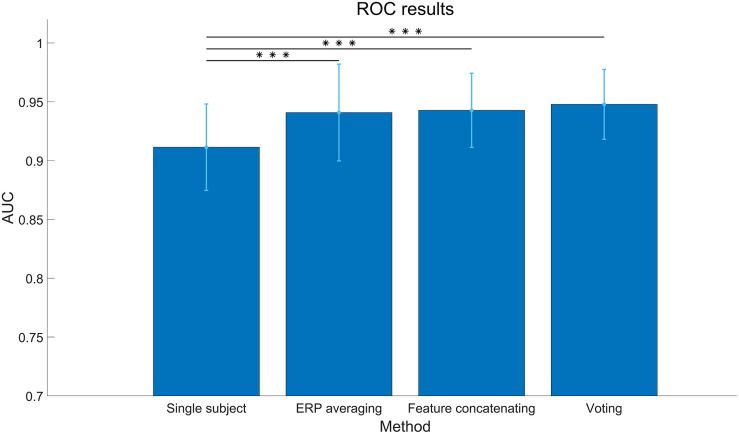
Area under curve (AUC) of different feature fusion methods in the collaborative data analysis. The classification algorithm is SIM + hierarchical discriminant component analysis (HDCA; *m* = 30). The asterisks indicate that the performance of the fusion method is significantly higher than the single subject (***:*p* < 0.001). The error bars indicate standard deviations.

**FIGURE 10:**
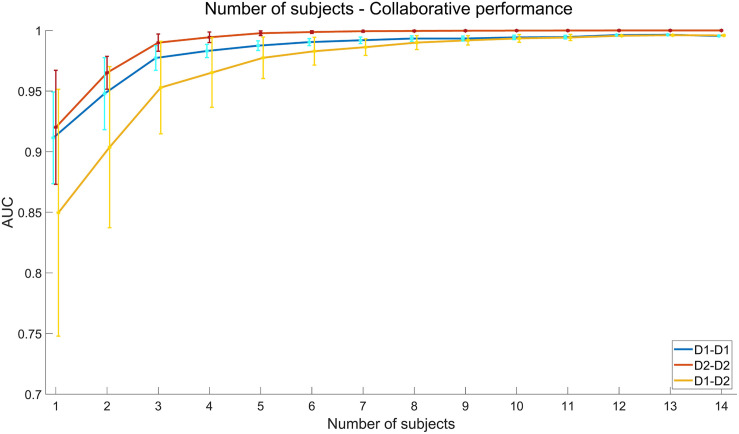
Simulated performance of multi-subject collaborative data analysis. The *x* axis indicates the number of subjects in each group. The feature fusion method is voting. The error bars indicate standard deviations.

The results of collaborative BCI performance are illustrated in Figure 9. For each group, the first block of data is used for training and the other two blocks are used for testing. The classification algorithm is SIM + HDCA (*m* = 30, according to the individual results in Figure 6). The feature fusion methods include ERP averaging, feature concatenating, and voting. As shown in Figure 9, all feature fusion algorithms can significantly improve the average individual performance (Single subject: 0.91 ± 0.03, ERP averaging: 0.94 ± 0.04, Feature concatenating: 0.94 ± 0.03, Voting: 0.94 ± 0.02, *p* < 0.001). The voting method achieves the highest AUC value. The performance of multi-subject collaborative experiments can be simulated by regrouping the subjects into new groups with more members. In addition to the individual condition and the collaborative condition with two subjects, all 14 subjects are regrouped to groups with 3–14 subjects. Since the number of random combinations is too large to compute, the maximum number of random groups with a fixed number of subjects in one group is set to 100. The simulation results in Figure 10 show that AUC increases significantly when the number of subjects is small, but AUC saturates (over 0.99 for both sessions) when the number of subjects reaches 5.

#### Cross-Session Collaborative Performance

**FIGURE 11:**
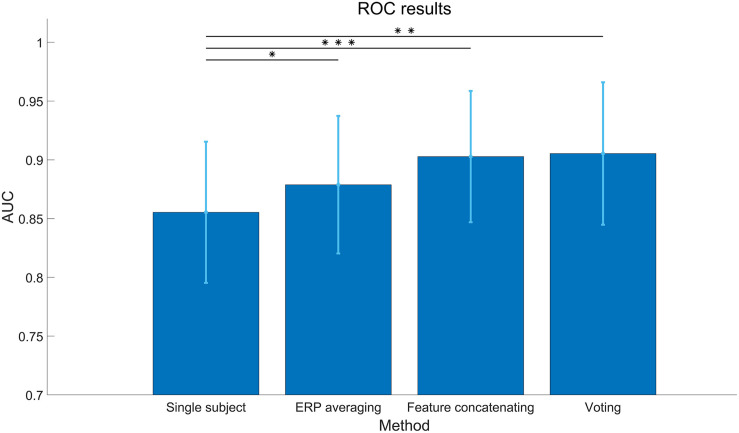
Cross-session area under curve (AUC) of different feature fusion methods in the collaborative data analysis. The classification algorithm is SIM + hierarchical discriminant component analysis (HDCA; *m* = 21). The asterisks indicate that the performance of the fusion method is significantly higher than the single subject (*: *p* < 0.05, **: *p* < 0.01, ***: *p* < 0.001). The error bars indicate standard deviations.

The results of collaborative BCI performance in the cross-session condition are illustrated in Figure 11. The classification algorithm is SIM + HDCA (*m* = 21, according to the individual results in Figure 6). As shown in Figure 11, all feature fusion algorithms can significantly improve the average performance of single subject (Single subject: 0.85 ± 0.06, ERP averaging: 0.87 ± 0.05, Feature concatenating: 0.90 ± 0.05, Voting: 0.90 ± 0.06, *p* < 0.05), and the voting method obtains the best performance. The ERP averaging method may be affected by the individual difference of amplitude and latency of ERPs, while the feature concatenating method increases the feature dimension which might increase the risk of overfitting. The voting method can avoid these problems by fusing the output scores together instead of fusing the EEG features. With the voting method, the cross-session collaborative performance is only slightly lower than the within-session collaborative performance and the difference is not significant (AUC: 0.90 ± 0.06 vs. 0.94 ± 0.02, *p* > 0.05). These results suggest that the cross-session method is efficient for the collaborative BCI. The simulated cross-session performance of a multi-subject BCI system is further shown in Figure 10. By increasing the number of subjects, the collaborative performance for the cross-session condition can also be significantly improved. The cross-session condition achieves similar performance to the within-session condition (Day 1) when the number of subjects increases to 10.

## Conclusion and Discussion

This study presents a cross-session dataset of a collaborative RSVP-based BCI. The results illustrate the distinct spatial and temporal features of ERPs related to target and non-target images. The comparison between different feature extraction and classification algorithms indicates that the combination of spatial filtering algorithms and HDCA can achieve good BCI performance in the individual condition, and the collaborative method can further improve the system performance by fusing information from multiple subjects. In the cross-session validation, the system performance can be optimized by selecting the number of components in the process by SNR maximizer for ERPs (SIM) algorithm. With the voting method, the cross-session collaborative performance is very close to the within-session collaborative performance (AUC: 0.90 vs 0.94). Although the cross-session AUC is still lower than the within-session AUC, the cross-session transfer can totally eliminate the system calibration procedure, which can substantially improve the practicality of the RSVP-based BCIs.

Since single-trial EEG data are recorded by multiple electrodes with various spatial and temporal features, suitable feature extraction and classification algorithms play important roles for ERP detection. In this paper, the SIM + HDCA algorithm achieves the best performance, but there is still room for improvement. First, the SSVEP component might contribute to ERP detection. As shown in the results, the single-trial EEG in RSVP tasks includes both ERPs and SSVEPs. However, the existing algorithms focus on the ERP components and ignore the SSVEP components in target detection. The difference of SSVEPs between target and non-target images requires further investigation by dissociating SSVEPs and ERPs (Zhang et al., 2018). Second, the number of components after spatial filtering was selected manually toward the highest AUC averaged across subjects and groups. The performance could be further improved by optimizing the number of components for each individual or group. In addition, the best number of components can be determined automatically by the algorithm toward a practical application.

The collaborative BCI method can be further improved by considering the following three directions. First, the feature fusion method can be improved by considering new features such as subject-to-subject synchronization or the response time of subjects (Poli et al., 2014; Valeriani et al., 2017). For instance, the EEG data of multiple subjects can be aligned by dynamic time warping (DTW) or canonical time warping (CTW) to synchronize the brain activities (Zhou and Torre, 2009). Second, the efficiency of the collaborative system can be optimized. For example, the BCI performance can be improved by collaborative paradigms with more subjects. However, a tradeoff between performance improvement and costs, which include equipment and labor costs in simultaneous EEG recording from multiple subjects, should be considered. When an individual subject achieves a high AUC value, the collaborative system can only obtain a minor improvement. Instead of the collaborative paradigm where the subjects perform the same detection tasks, another paradigm is to assign different tasks to each subject. This mode of division can improve the total efficiency of target detection tasks by reducing total time, but the individual performance remains the same. Third, CV can be combined with the RSVP-based BCI system. By optimally combining CV and HV, the system performance can be further improved (Sajda et al., 2010; Pohlmeyer et al., 2011).

In addition to the validation of collaborative and cross-session BCI performance in this study, this dataset can be used to study the following topics: (1) brain dynamics of ERPs and SSVEPs in the RSVP-based BCI paradigm, (2) data analysis algorithms for single-trial ERP detection, (3) data fusion methods for collaborative BCIs, and (4) transfer learning algorithms for the cross-session ERP-based BCIs.

## Data Availability Statement

The dataset analyzed in this study can be downloaded at https://doi.org/10.6084/m9.figshare.12824771.v1.

## Ethics Statement

The studies involving human participants were reviewed and approved by the Ethics Committee of Tsinghua University. The patients/participants provided their written informed consent to participate in this study.

## Author Contributions

LZhe performed the data collection, data analysis, and wrote the manuscript. SS developed the experimental system and performed the data collection. HZ performed the data analysis. WP, HC, LZha, and XG revised the manuscript. YW supervised the study. All authors contributed to the article and approved the submitted version.

## Conflict of Interest

The authors declare that the research was conducted in the absence of any commercial or financial relationships that could be construed as a potential conflict of interest.
