# Supplementary figures and images for: A Cross-Session Dataset for Collaborative Brain-Computer Interfaces Based on Rapid Serial Visual Presentation (part 1 of 5)
Source: Front Neurosci. 2020 Oct 22;14:579469. doi: 10.3389/fnins.2020.579469 (PMC7642747; doi:10.3389/fnins.2020.579469)

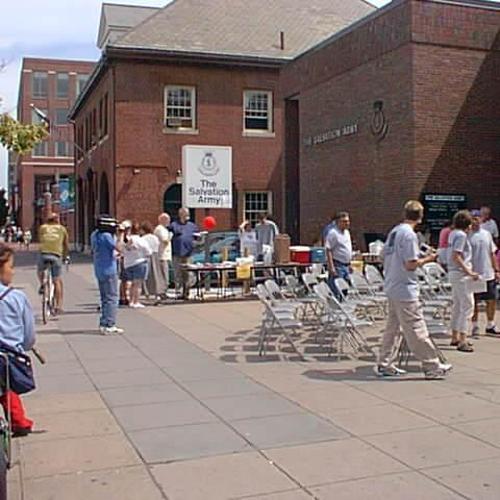

Supplement: Supplementary file 2 [file Presentation_2.zip › Targets/image_0001.jpg]

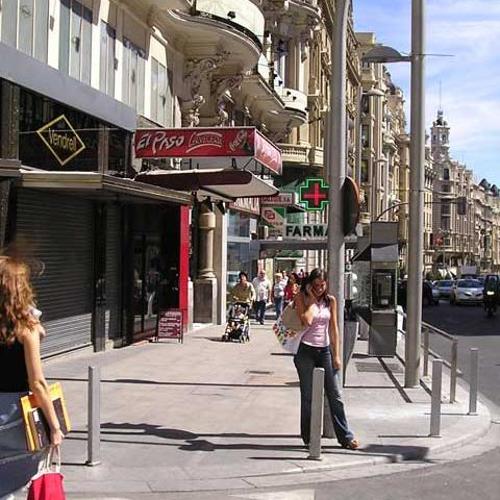

Supplement: Supplementary file 2 [file Presentation_2.zip › Targets/image_0002.jpg]

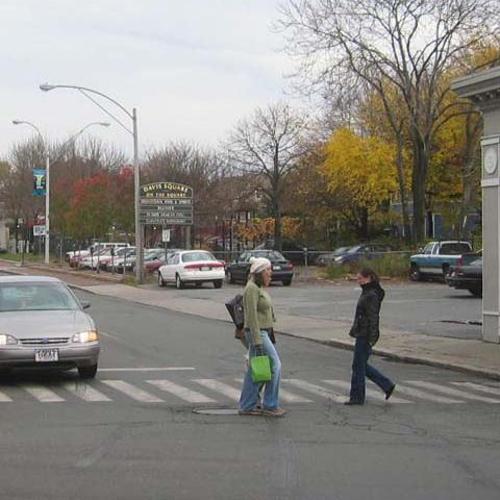

Supplement: Supplementary file 2 [file Presentation_2.zip › Targets/image_0003.jpg]

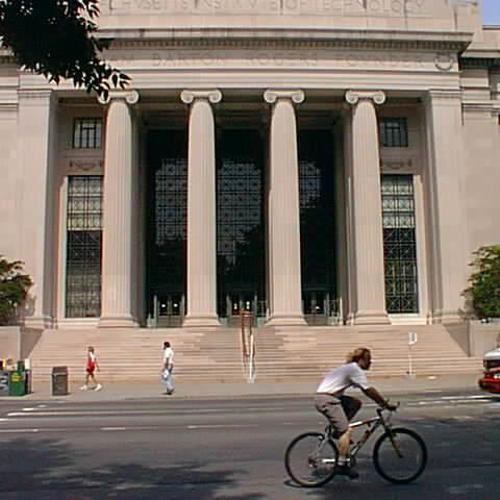

Supplement: Supplementary file 2 [file Presentation_2.zip › Targets/image_0004.jpg]

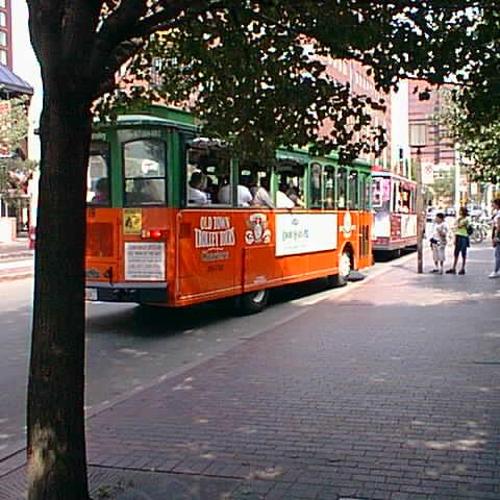

Supplement: Supplementary file 2 [file Presentation_2.zip › Targets/image_0005.jpg]

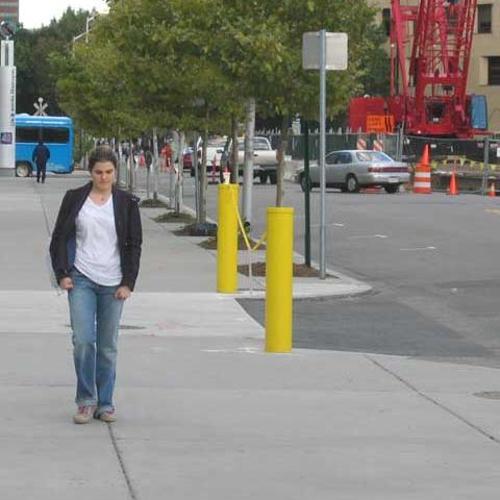

Supplement: Supplementary file 2 [file Presentation_2.zip › Targets/image_0006.jpg]

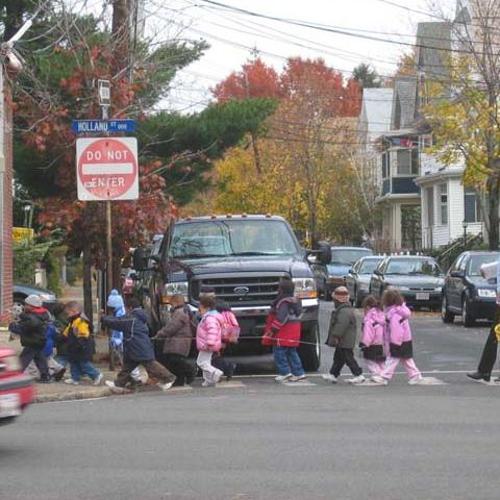

Supplement: Supplementary file 2 [file Presentation_2.zip › Targets/image_0007.jpg]

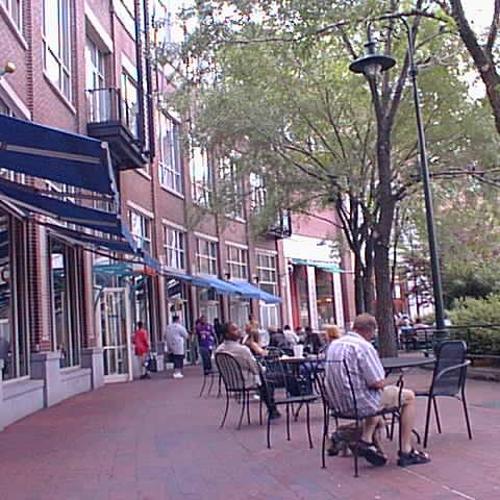

Supplement: Supplementary file 2 [file Presentation_2.zip › Targets/image_0008.jpg]

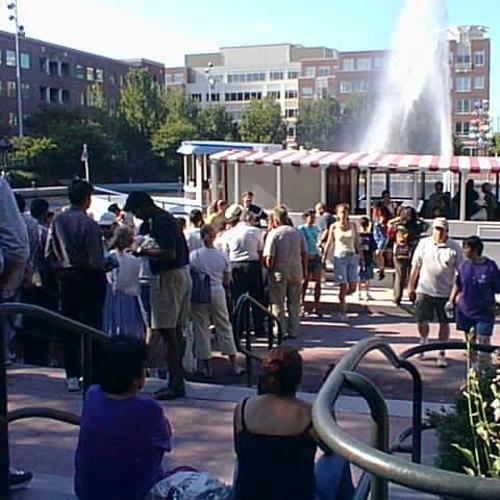

Supplement: Supplementary file 2 [file Presentation_2.zip › Targets/image_0009.jpg]

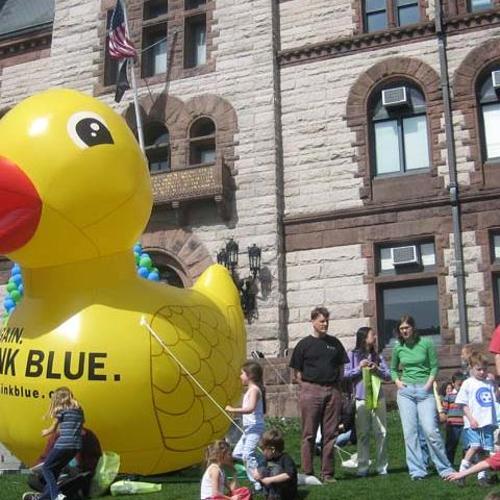

Supplement: Supplementary file 2 [file Presentation_2.zip › Targets/image_0010.jpg]

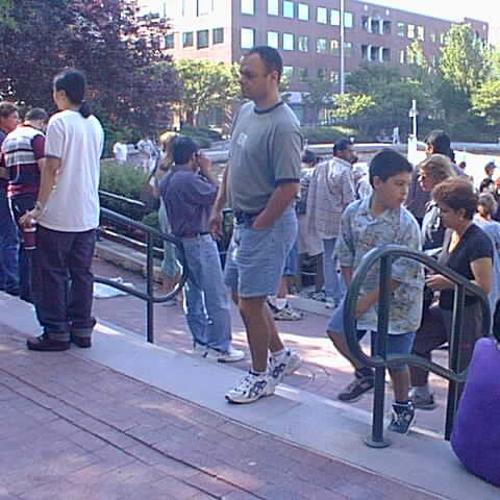

Supplement: Supplementary file 2 [file Presentation_2.zip › Targets/image_0011.jpg]

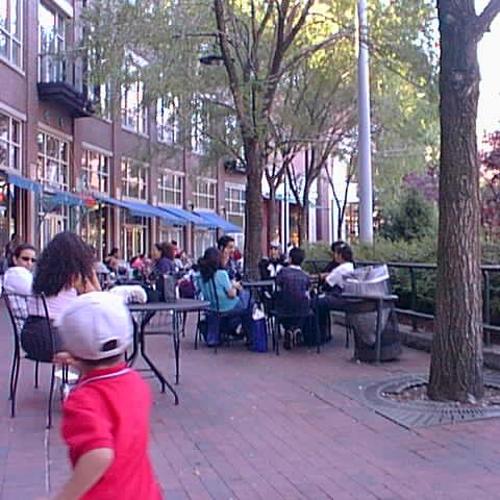

Supplement: Supplementary file 2 [file Presentation_2.zip › Targets/image_0012.jpg]

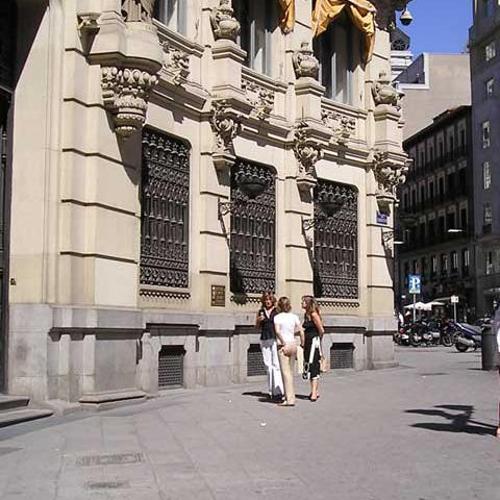

Supplement: Supplementary file 2 [file Presentation_2.zip › Targets/image_0013.jpg]

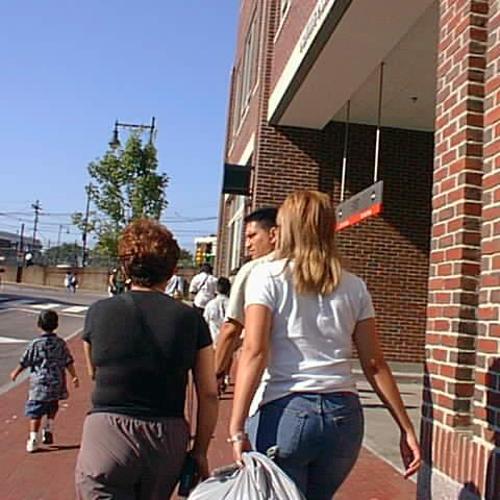

Supplement: Supplementary file 2 [file Presentation_2.zip › Targets/image_0014.jpg]

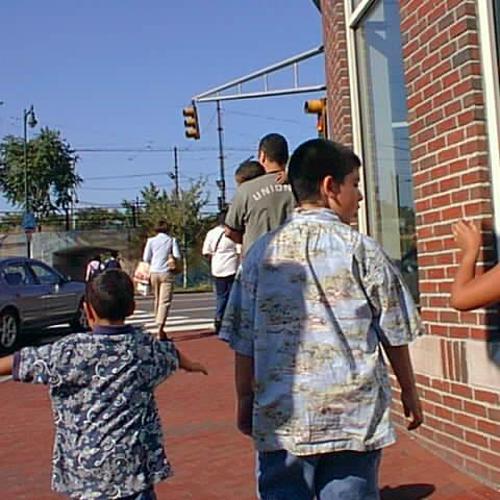

Supplement: Supplementary file 2 [file Presentation_2.zip › Targets/image_0015.jpg]

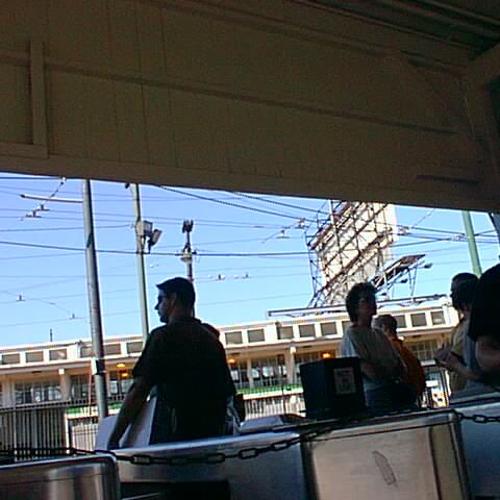

Supplement: Supplementary file 2 [file Presentation_2.zip › Targets/image_0016.jpg]

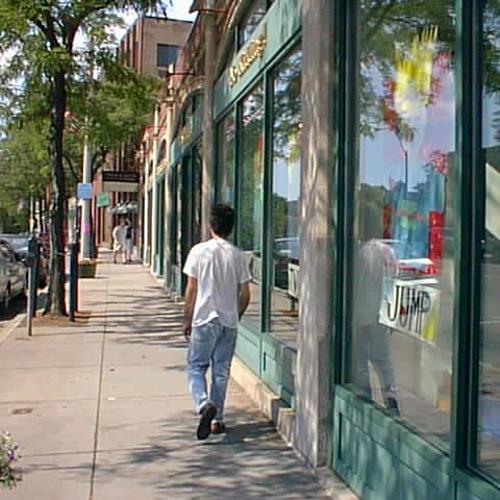

Supplement: Supplementary file 2 [file Presentation_2.zip › Targets/image_0017.jpg]

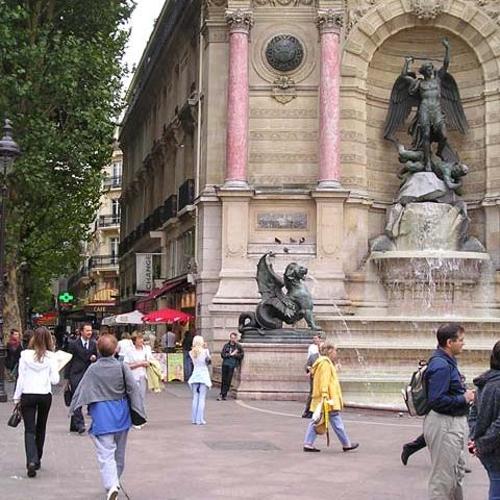

Supplement: Supplementary file 2 [file Presentation_2.zip › Targets/image_0018.jpg]

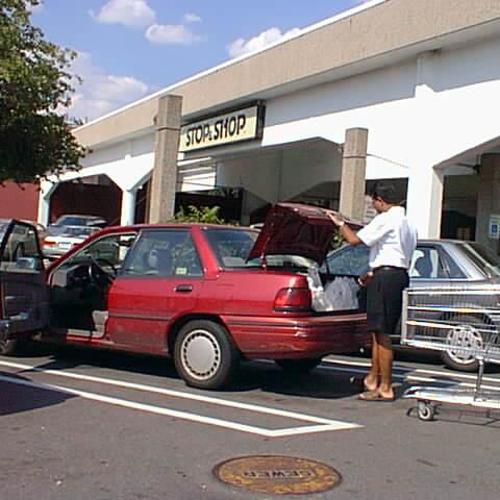

Supplement: Supplementary file 2 [file Presentation_2.zip › Targets/image_0019.jpg]

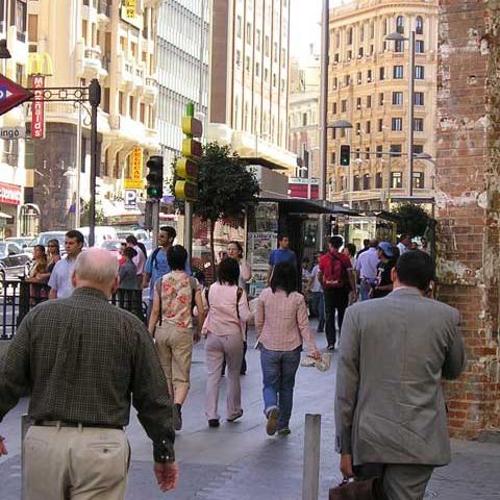

Supplement: Supplementary file 2 [file Presentation_2.zip › Targets/image_0020.jpg]

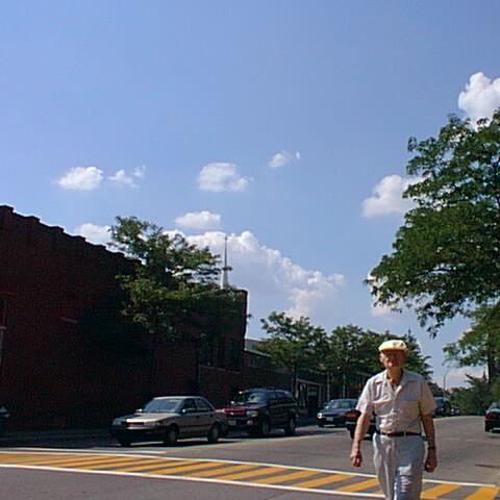

Supplement: Supplementary file 2 [file Presentation_2.zip › Targets/image_0021.jpg]

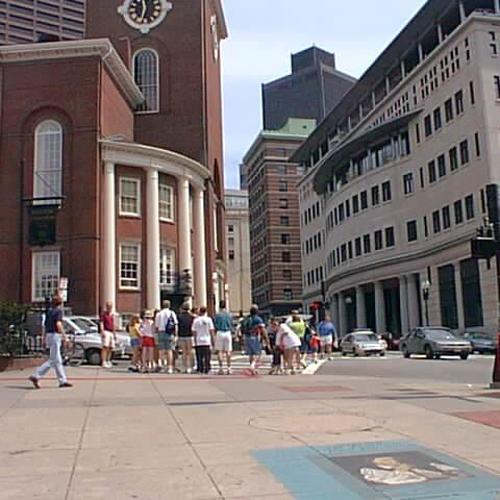

Supplement: Supplementary file 2 [file Presentation_2.zip › Targets/image_0022.jpg]

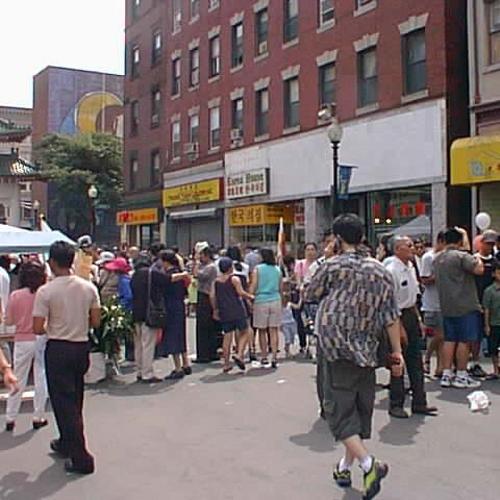

Supplement: Supplementary file 2 [file Presentation_2.zip › Targets/image_0023.jpg]

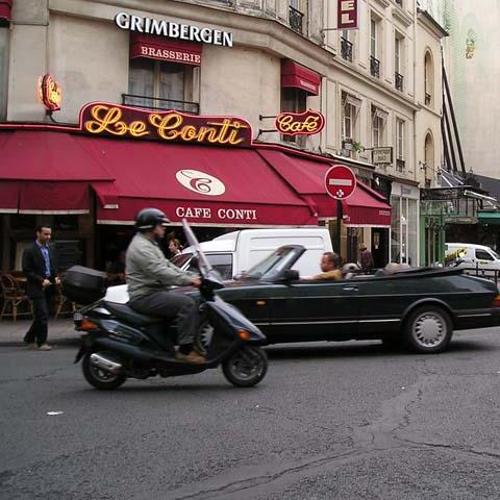

Supplement: Supplementary file 2 [file Presentation_2.zip › Targets/image_0024.jpg]

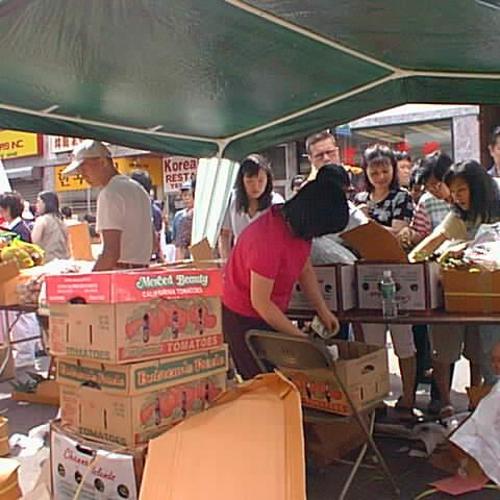

Supplement: Supplementary file 2 [file Presentation_2.zip › Targets/image_0025.jpg]

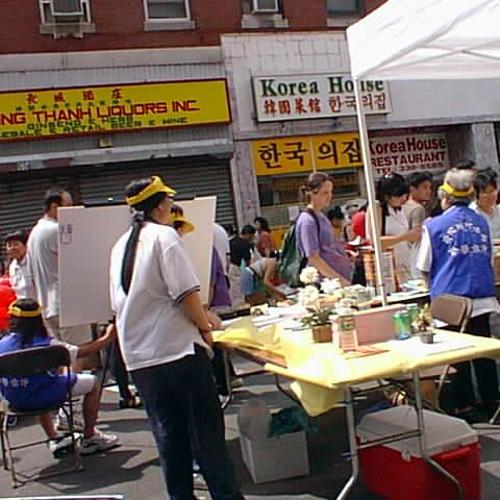

Supplement: Supplementary file 2 [file Presentation_2.zip › Targets/image_0026.jpg]

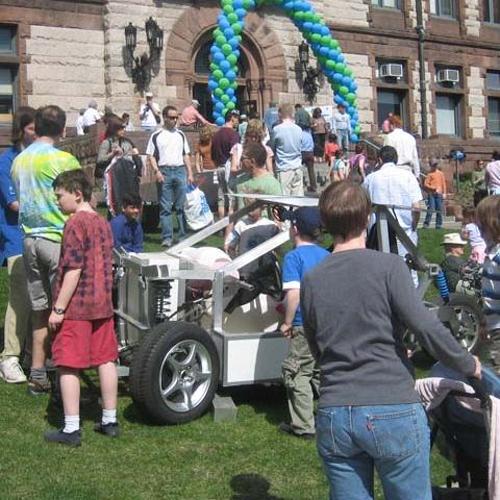

Supplement: Supplementary file 2 [file Presentation_2.zip › Targets/image_0027.jpg]

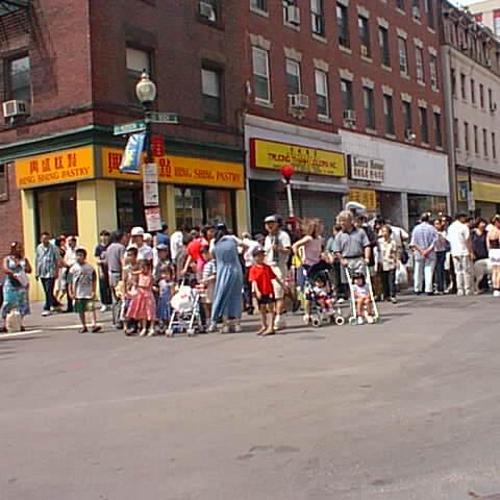

Supplement: Supplementary file 2 [file Presentation_2.zip › Targets/image_0028.jpg]

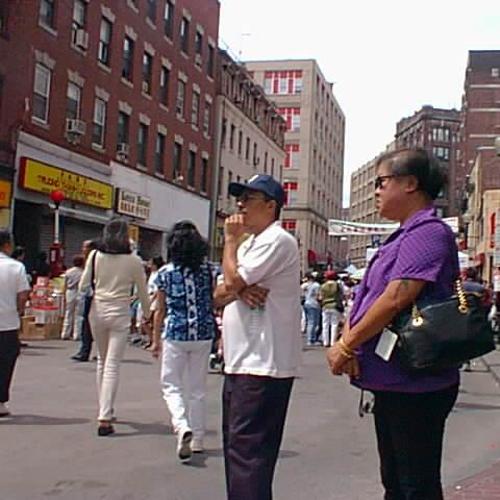

Supplement: Supplementary file 2 [file Presentation_2.zip › Targets/image_0029.jpg]

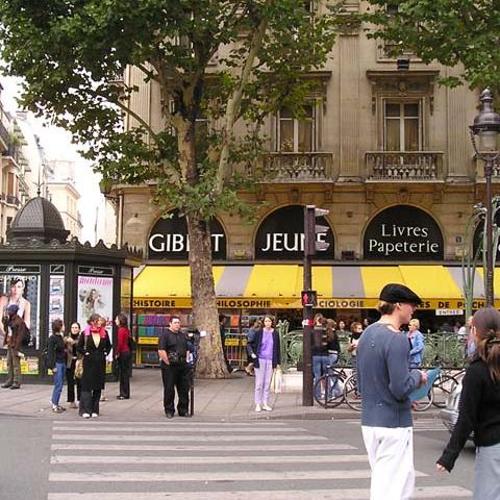

Supplement: Supplementary file 2 [file Presentation_2.zip › Targets/image_0030.jpg]

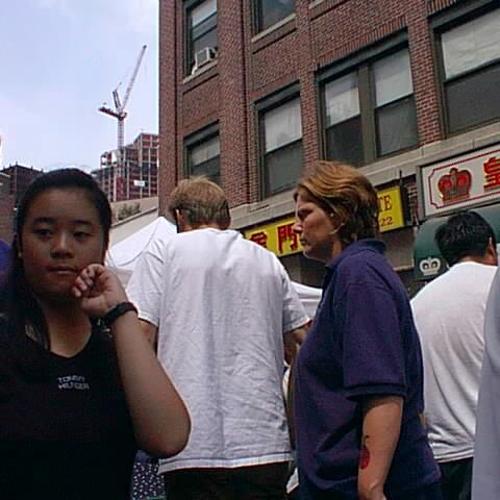

Supplement: Supplementary file 2 [file Presentation_2.zip › Targets/image_0031.jpg]

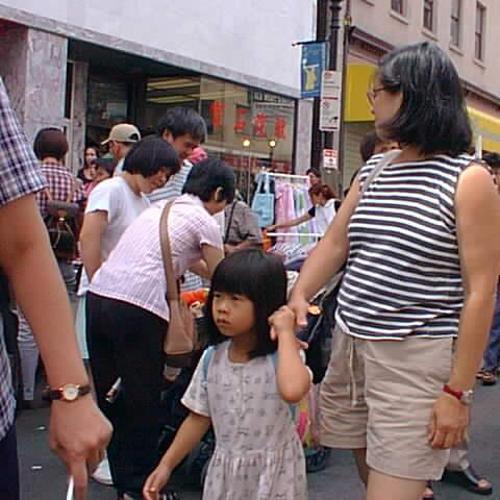

Supplement: Supplementary file 2 [file Presentation_2.zip › Targets/image_0032.jpg]

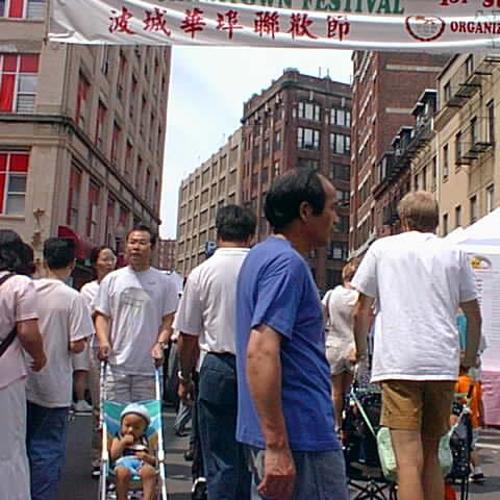

Supplement: Supplementary file 2 [file Presentation_2.zip › Targets/image_0033.jpg]

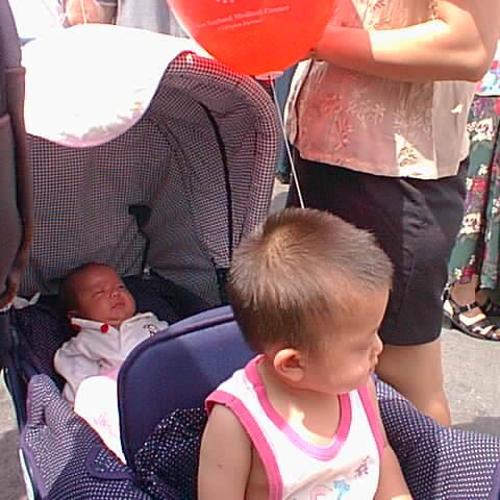

Supplement: Supplementary file 2 [file Presentation_2.zip › Targets/image_0034.jpg]

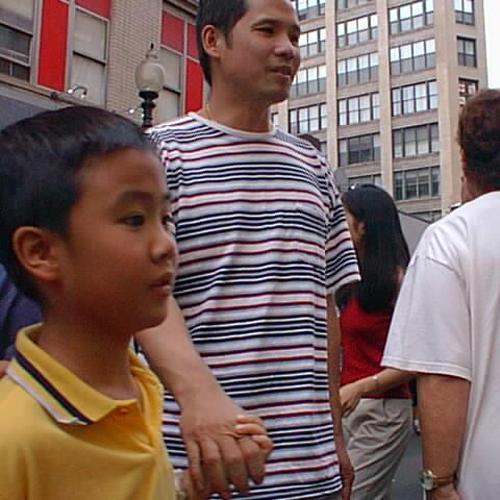

Supplement: Supplementary file 2 [file Presentation_2.zip › Targets/image_0035.jpg]

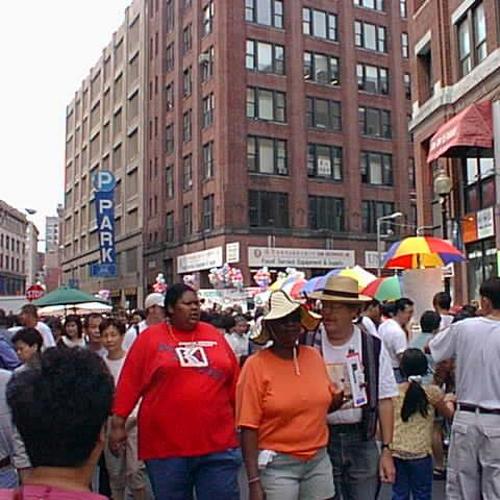

Supplement: Supplementary file 2 [file Presentation_2.zip › Targets/image_0036.jpg]

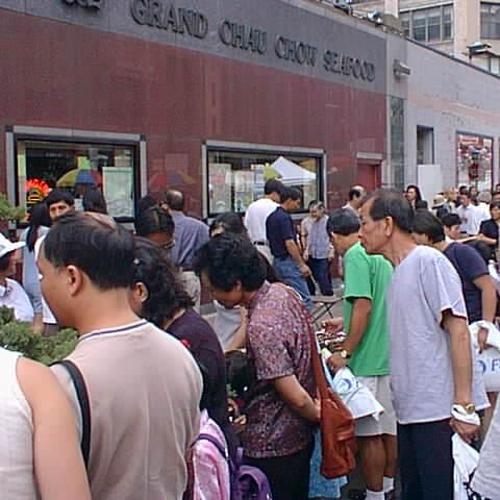

Supplement: Supplementary file 2 [file Presentation_2.zip › Targets/image_0037.jpg]

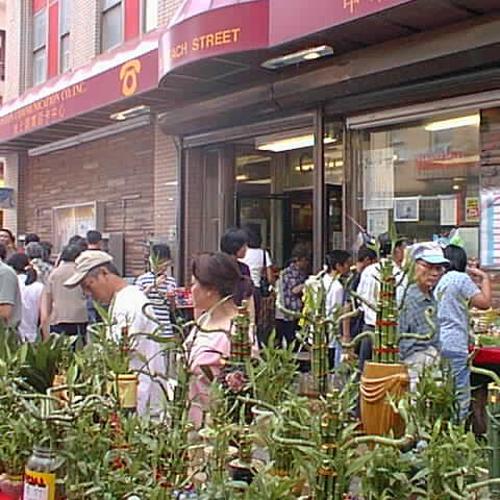

Supplement: Supplementary file 2 [file Presentation_2.zip › Targets/image_0038.jpg]

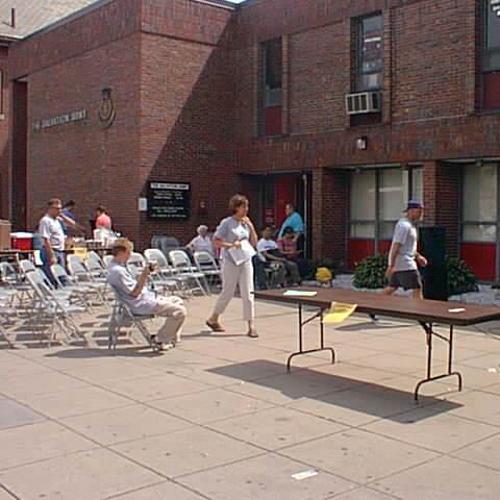

Supplement: Supplementary file 2 [file Presentation_2.zip › Targets/image_0039.jpg]

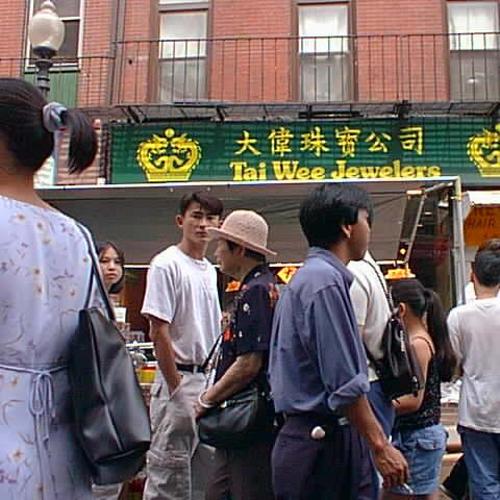

Supplement: Supplementary file 2 [file Presentation_2.zip › Targets/image_0040.jpg]

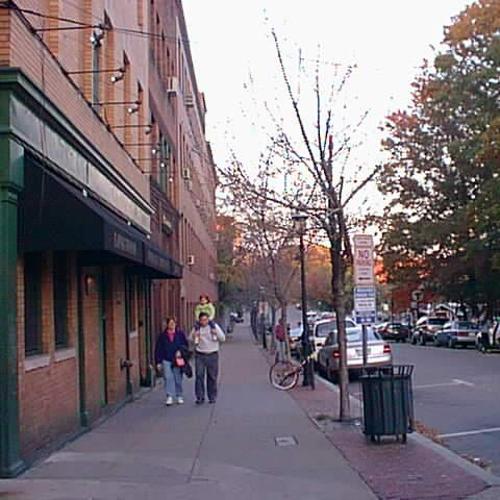

Supplement: Supplementary file 2 [file Presentation_2.zip › Targets/image_0041.jpg]

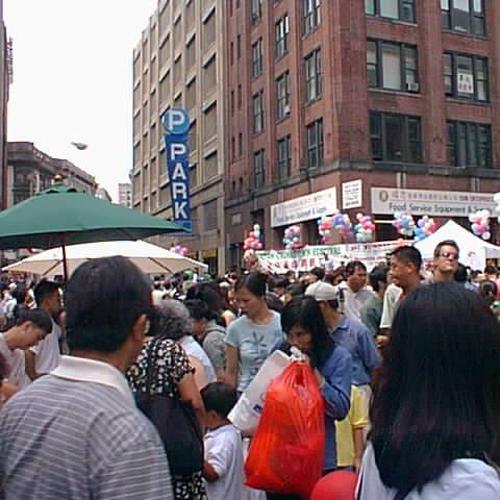

Supplement: Supplementary file 2 [file Presentation_2.zip › Targets/image_0042.jpg]

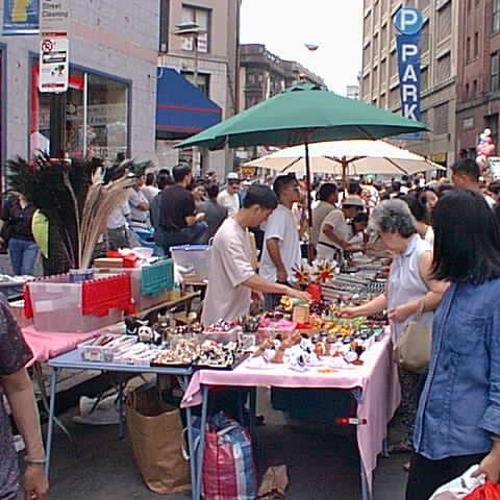

Supplement: Supplementary file 2 [file Presentation_2.zip › Targets/image_0043.jpg]

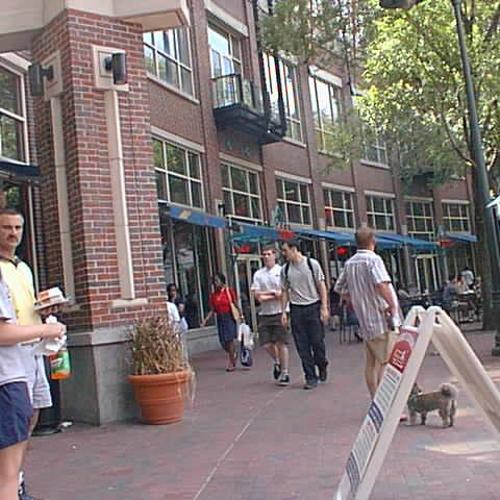

Supplement: Supplementary file 2 [file Presentation_2.zip › Targets/image_0044.jpg]

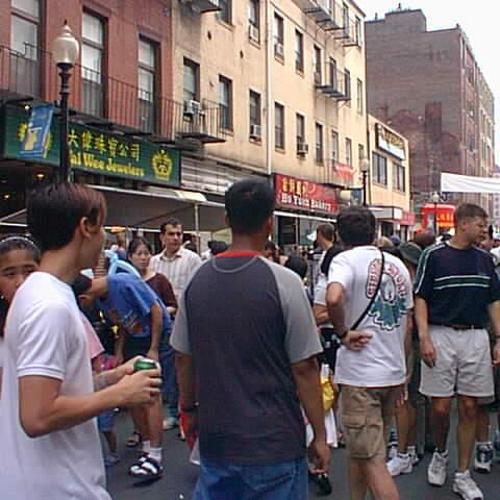

Supplement: Supplementary file 2 [file Presentation_2.zip › Targets/image_0045.jpg]

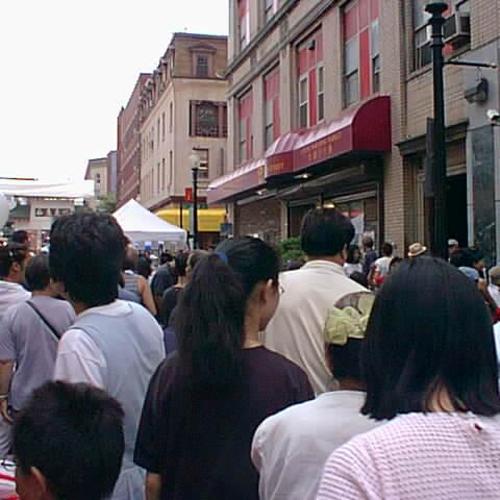

Supplement: Supplementary file 2 [file Presentation_2.zip › Targets/image_0046.jpg]

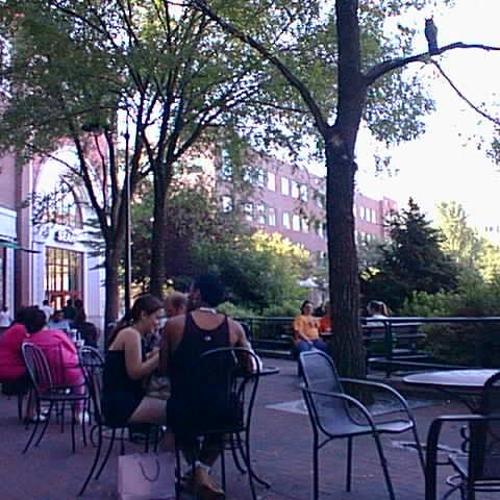

Supplement: Supplementary file 2 [file Presentation_2.zip › Targets/image_0047.jpg]

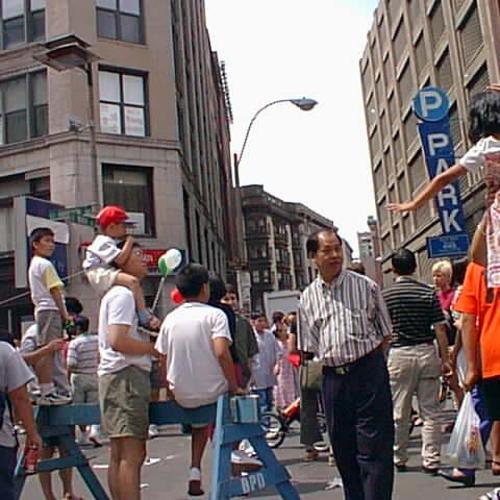

Supplement: Supplementary file 2 [file Presentation_2.zip › Targets/image_0048.jpg]

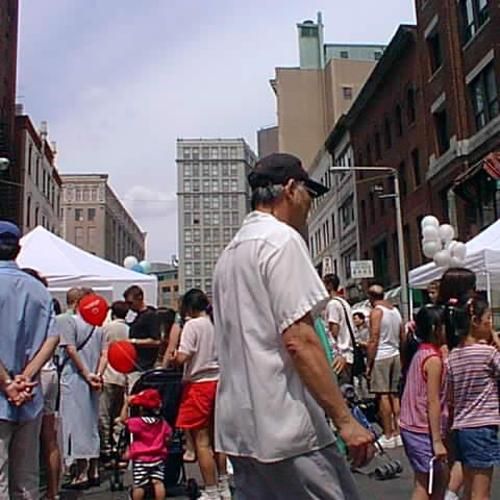

Supplement: Supplementary file 2 [file Presentation_2.zip › Targets/image_0049.jpg]

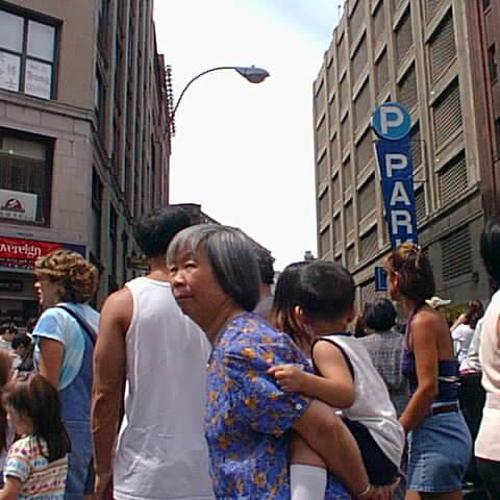

Supplement: Supplementary file 2 [file Presentation_2.zip › Targets/image_0050.jpg]

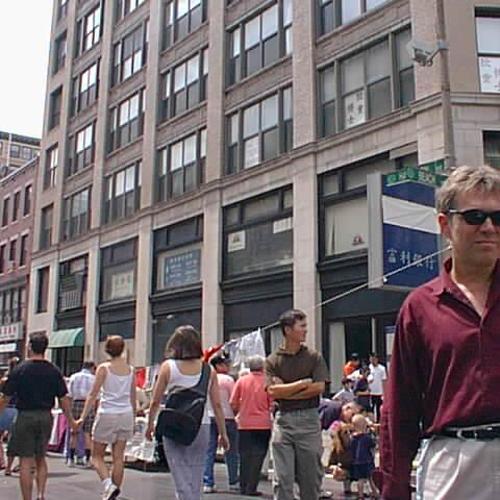

Supplement: Supplementary file 2 [file Presentation_2.zip › Targets/image_0051.jpg]

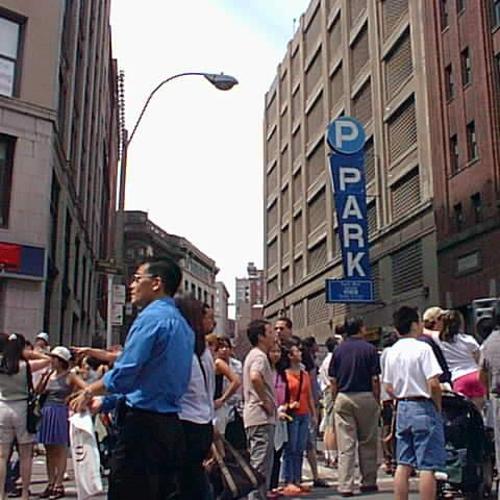

Supplement: Supplementary file 2 [file Presentation_2.zip › Targets/image_0052.jpg]

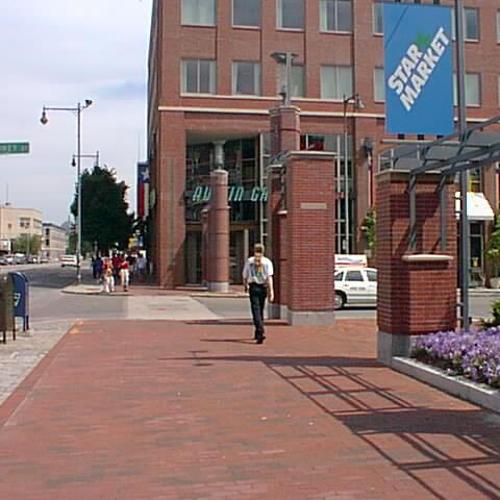

Supplement: Supplementary file 2 [file Presentation_2.zip › Targets/image_0053.jpg]

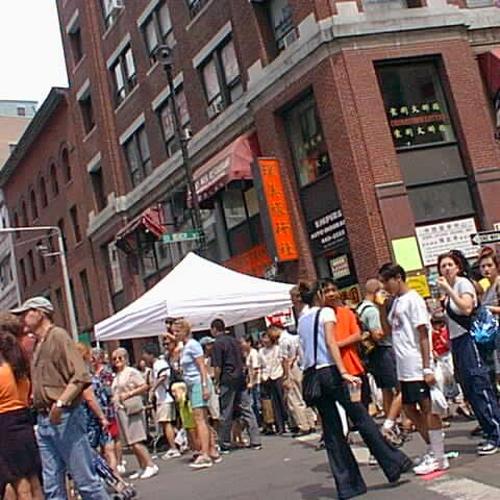

Supplement: Supplementary file 2 [file Presentation_2.zip › Targets/image_0054.jpg]

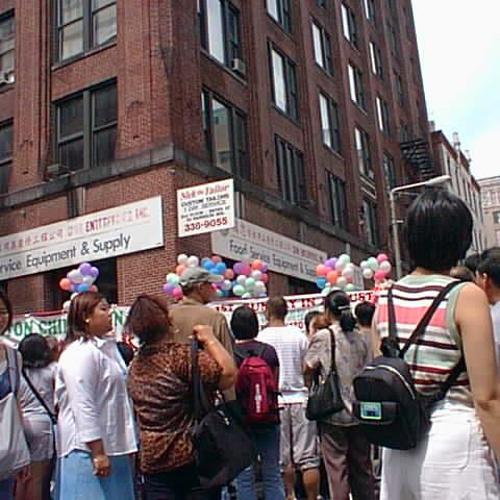

Supplement: Supplementary file 2 [file Presentation_2.zip › Targets/image_0055.jpg]

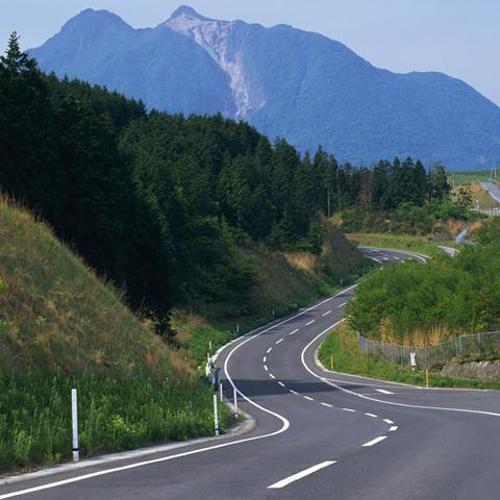

Supplement: Supplementary file 3 [file Presentation_3.zip › Non-targets_1/image_0001.jpg]

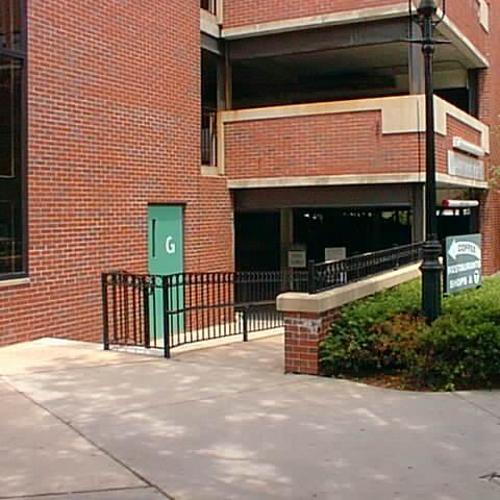

Supplement: Supplementary file 3 [file Presentation_3.zip › Non-targets_1/image_0002.jpg]

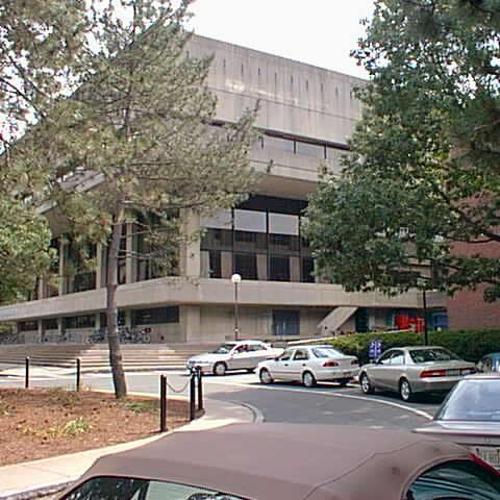

Supplement: Supplementary file 3 [file Presentation_3.zip › Non-targets_1/image_0003.jpg]

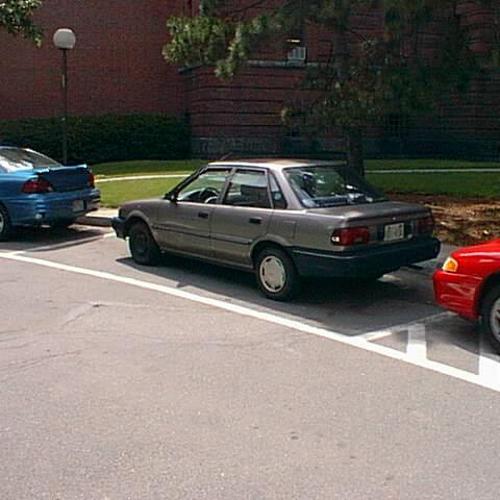

Supplement: Supplementary file 3 [file Presentation_3.zip › Non-targets_1/image_0004.jpg]

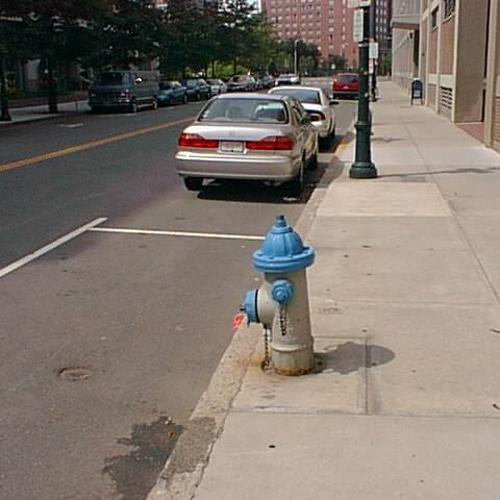

Supplement: Supplementary file 3 [file Presentation_3.zip › Non-targets_1/image_0005.jpg]

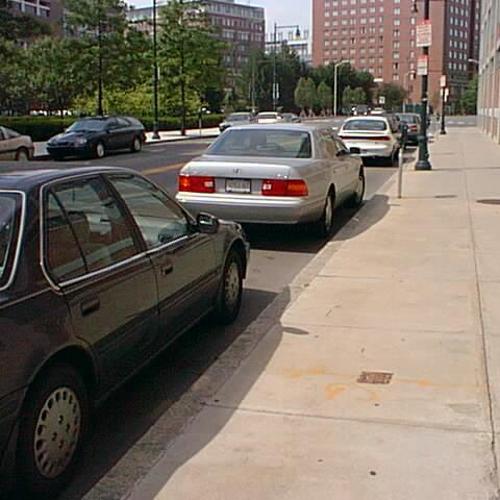

Supplement: Supplementary file 3 [file Presentation_3.zip › Non-targets_1/image_0006.jpg]

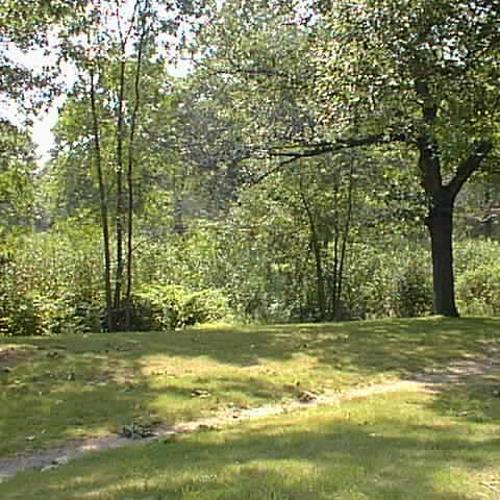

Supplement: Supplementary file 3 [file Presentation_3.zip › Non-targets_1/image_0007.jpg]

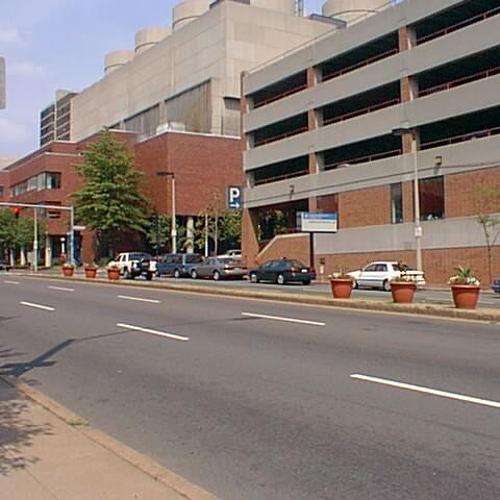

Supplement: Supplementary file 3 [file Presentation_3.zip › Non-targets_1/image_0008.jpg]

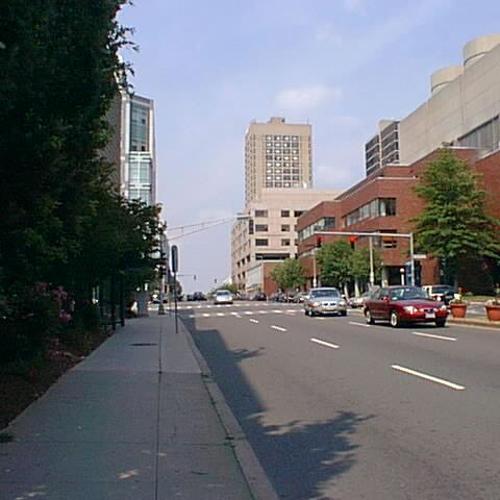

Supplement: Supplementary file 3 [file Presentation_3.zip › Non-targets_1/image_0009.jpg]

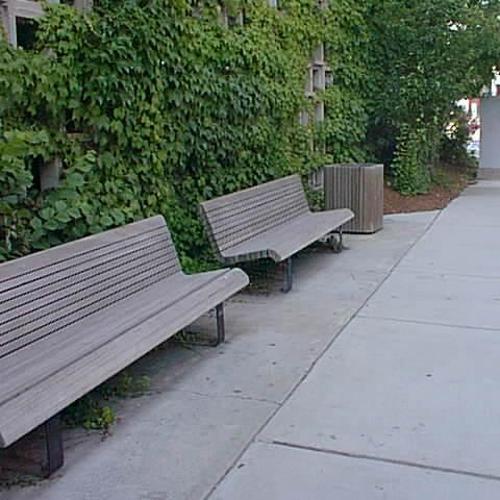

Supplement: Supplementary file 3 [file Presentation_3.zip › Non-targets_1/image_0010.jpg]

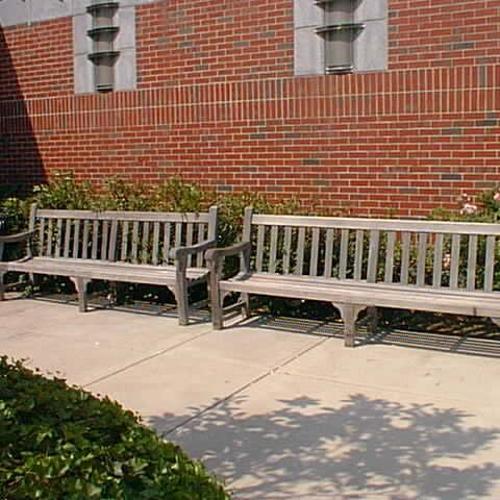

Supplement: Supplementary file 3 [file Presentation_3.zip › Non-targets_1/image_0011.jpg]

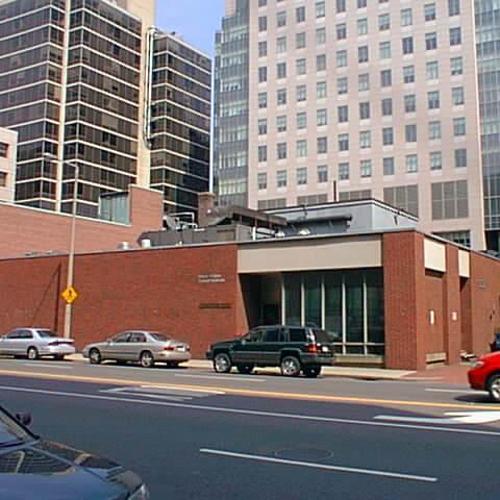

Supplement: Supplementary file 3 [file Presentation_3.zip › Non-targets_1/image_0012.jpg]

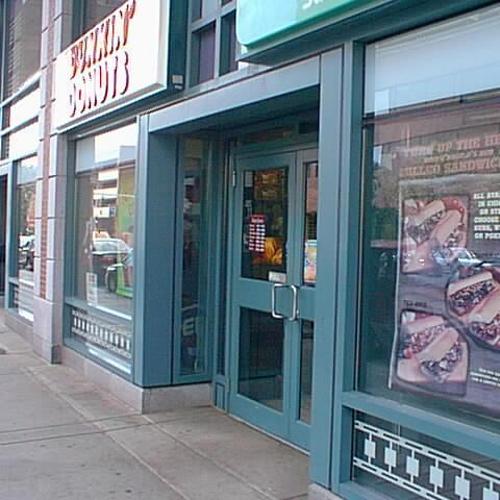

Supplement: Supplementary file 3 [file Presentation_3.zip › Non-targets_1/image_0013.jpg]

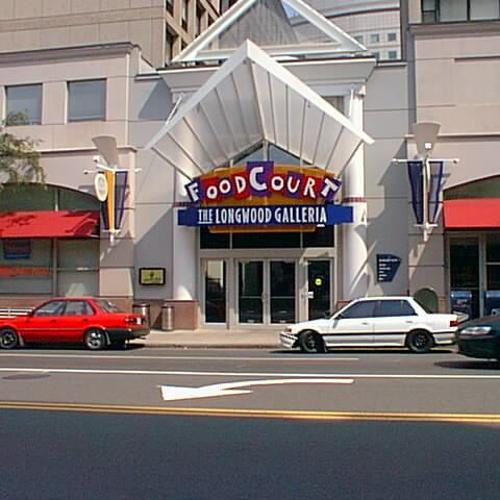

Supplement: Supplementary file 3 [file Presentation_3.zip › Non-targets_1/image_0014.jpg]

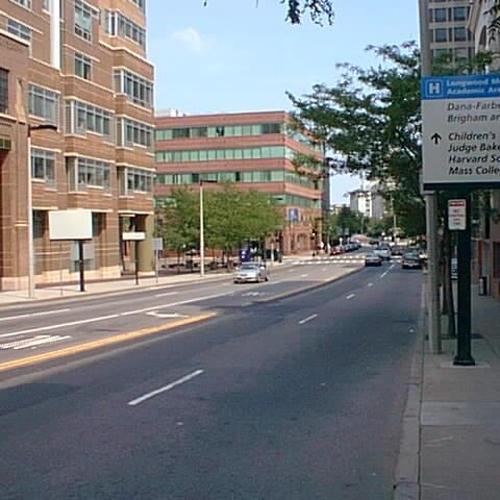

Supplement: Supplementary file 3 [file Presentation_3.zip › Non-targets_1/image_0015.jpg]

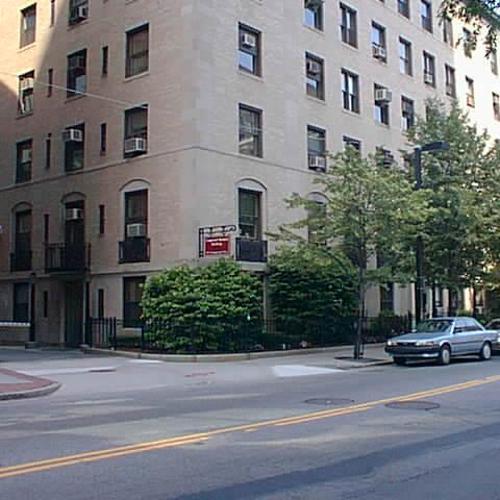

Supplement: Supplementary file 3 [file Presentation_3.zip › Non-targets_1/image_0016.jpg]

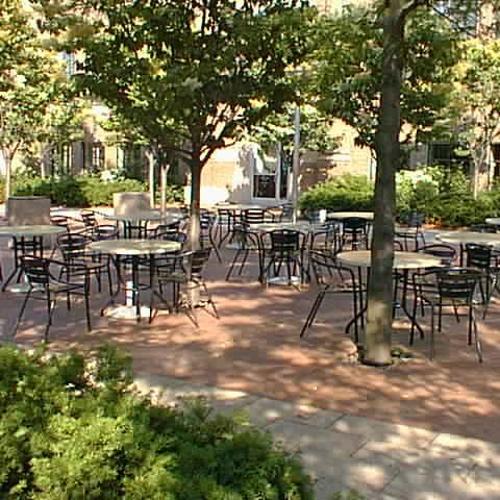

Supplement: Supplementary file 3 [file Presentation_3.zip › Non-targets_1/image_0017.jpg]

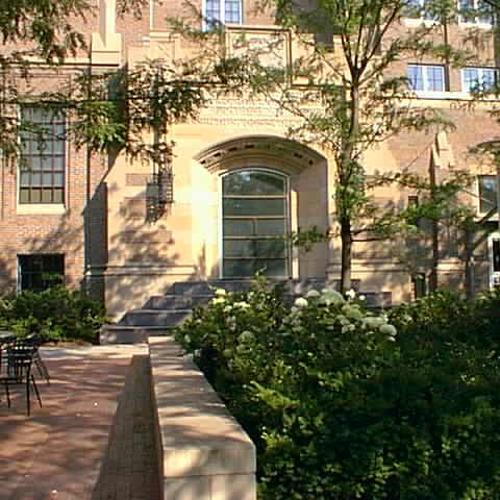

Supplement: Supplementary file 3 [file Presentation_3.zip › Non-targets_1/image_0018.jpg]

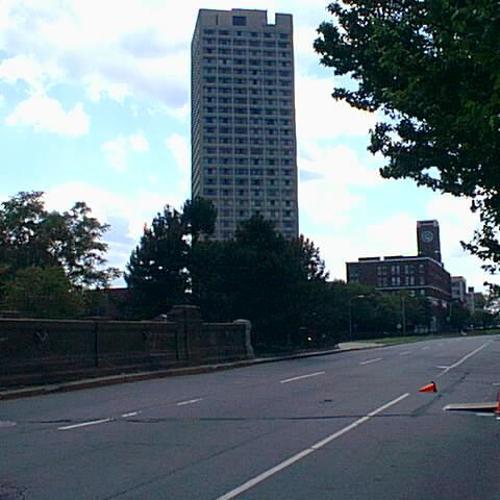

Supplement: Supplementary file 3 [file Presentation_3.zip › Non-targets_1/image_0019.jpg]

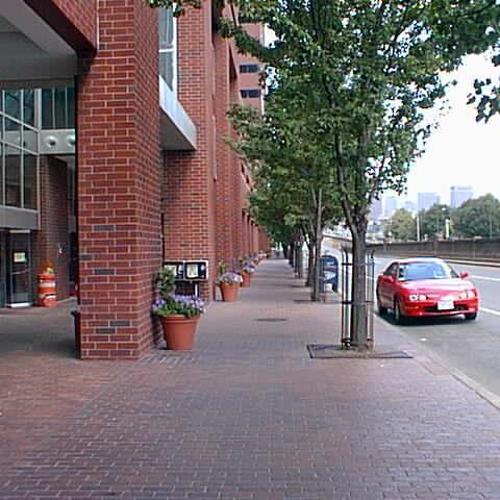

Supplement: Supplementary file 3 [file Presentation_3.zip › Non-targets_1/image_0020.jpg]

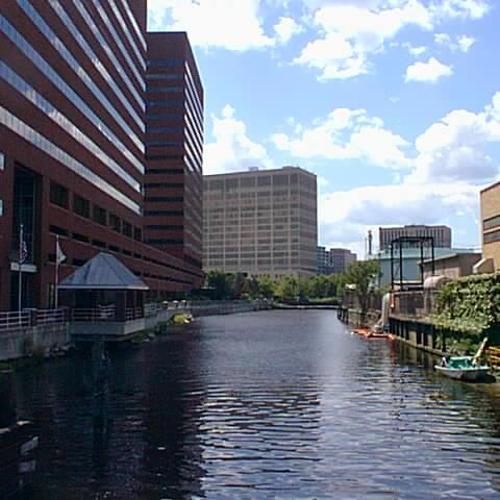

Supplement: Supplementary file 3 [file Presentation_3.zip › Non-targets_1/image_0021.jpg]

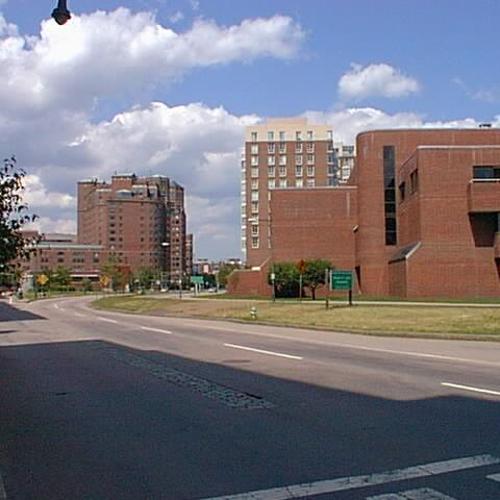

Supplement: Supplementary file 3 [file Presentation_3.zip › Non-targets_1/image_0022.jpg]

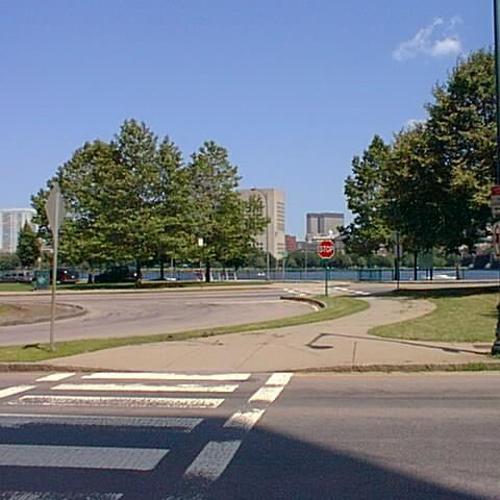

Supplement: Supplementary file 3 [file Presentation_3.zip › Non-targets_1/image_0023.jpg]

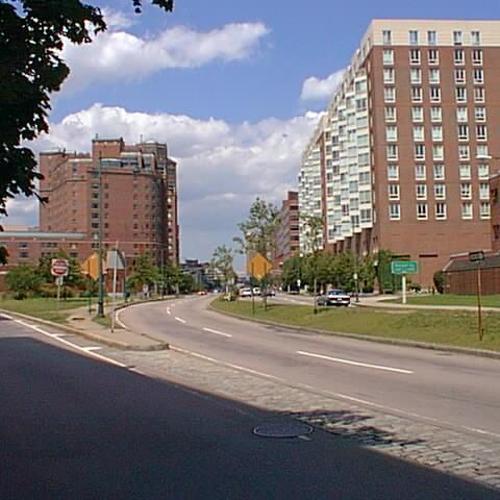

Supplement: Supplementary file 3 [file Presentation_3.zip › Non-targets_1/image_0024.jpg]

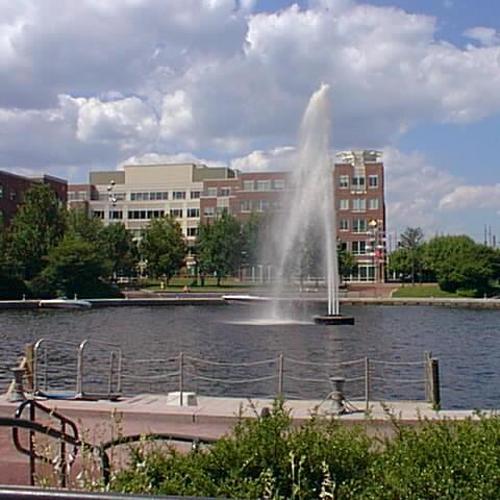

Supplement: Supplementary file 3 [file Presentation_3.zip › Non-targets_1/image_0025.jpg]

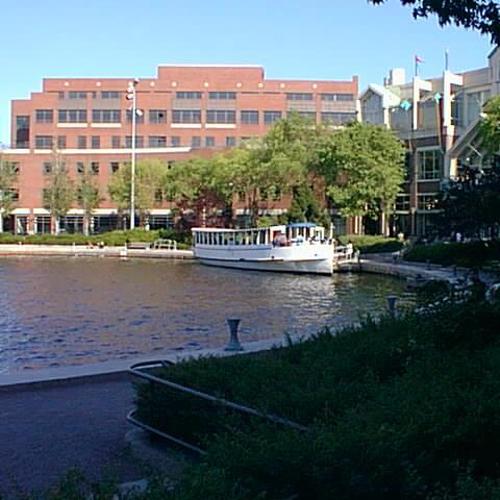

Supplement: Supplementary file 3 [file Presentation_3.zip › Non-targets_1/image_0026.jpg]

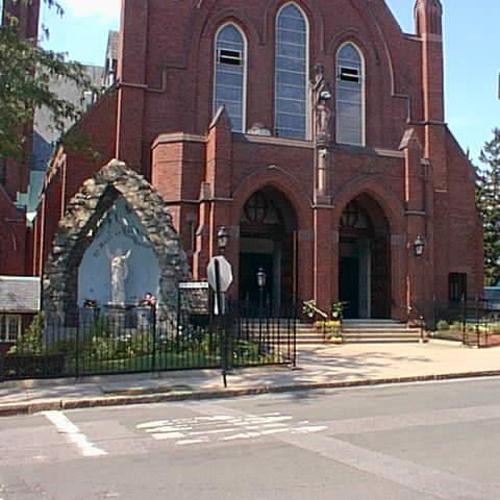

Supplement: Supplementary file 3 [file Presentation_3.zip › Non-targets_1/image_0027.jpg]

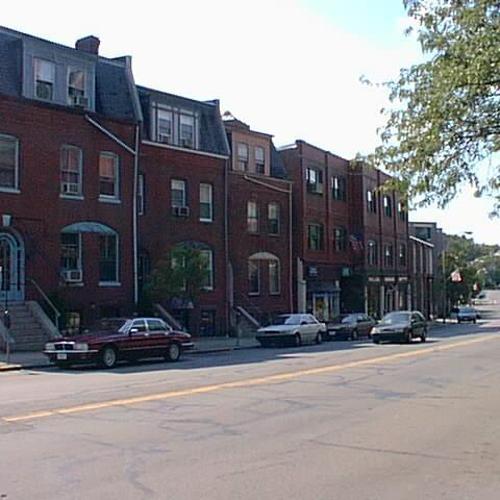

Supplement: Supplementary file 3 [file Presentation_3.zip › Non-targets_1/image_0028.jpg]

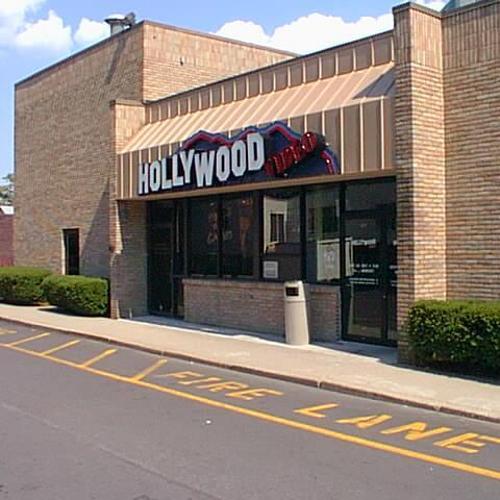

Supplement: Supplementary file 3 [file Presentation_3.zip › Non-targets_1/image_0029.jpg]

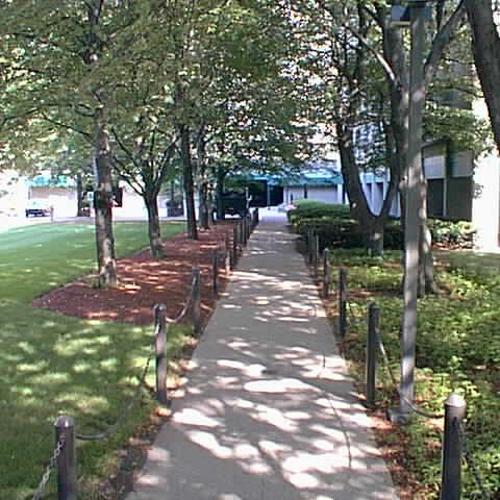

Supplement: Supplementary file 3 [file Presentation_3.zip › Non-targets_1/image_0030.jpg]

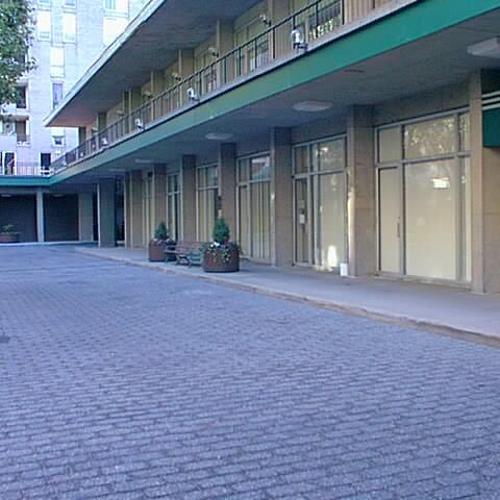

Supplement: Supplementary file 3 [file Presentation_3.zip › Non-targets_1/image_0031.jpg]

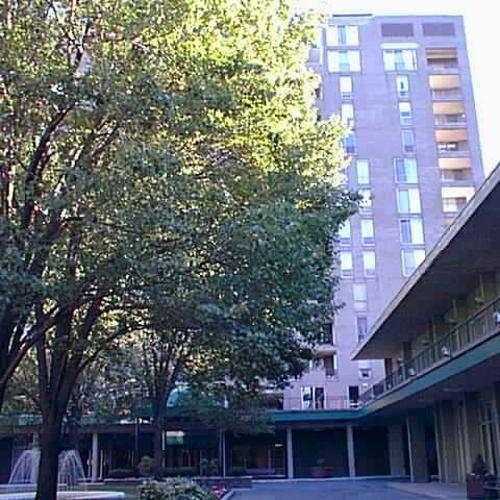

Supplement: Supplementary file 3 [file Presentation_3.zip › Non-targets_1/image_0032.jpg]

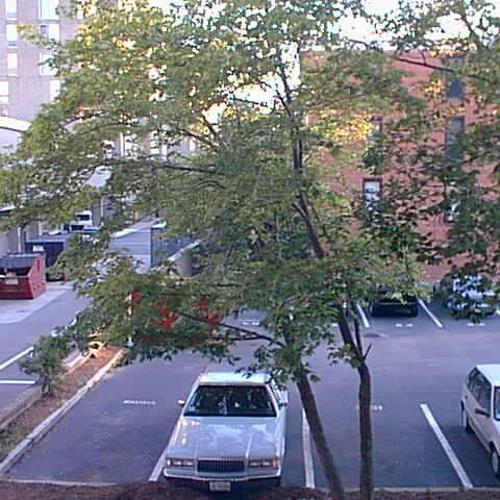

Supplement: Supplementary file 3 [file Presentation_3.zip › Non-targets_1/image_0033.jpg]

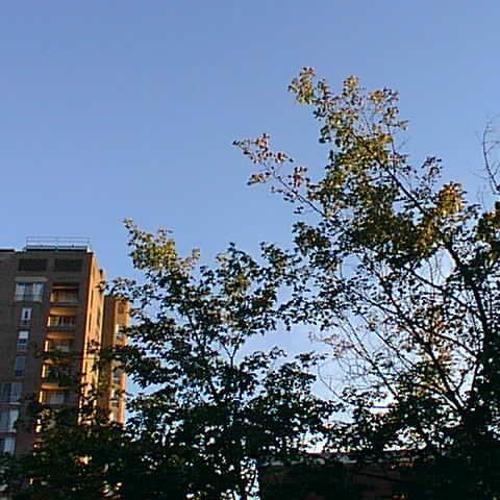

Supplement: Supplementary file 3 [file Presentation_3.zip › Non-targets_1/image_0034.jpg]

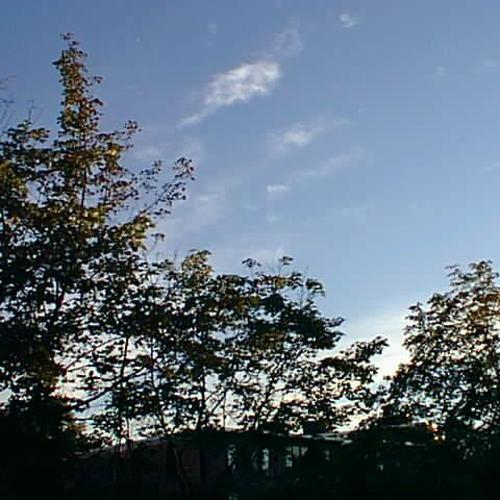

Supplement: Supplementary file 3 [file Presentation_3.zip › Non-targets_1/image_0035.jpg]

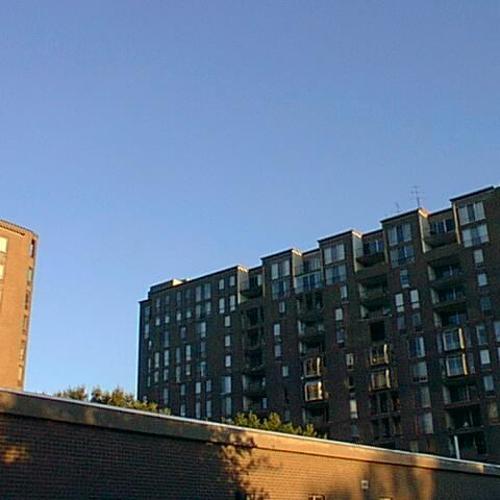

Supplement: Supplementary file 3 [file Presentation_3.zip › Non-targets_1/image_0036.jpg]

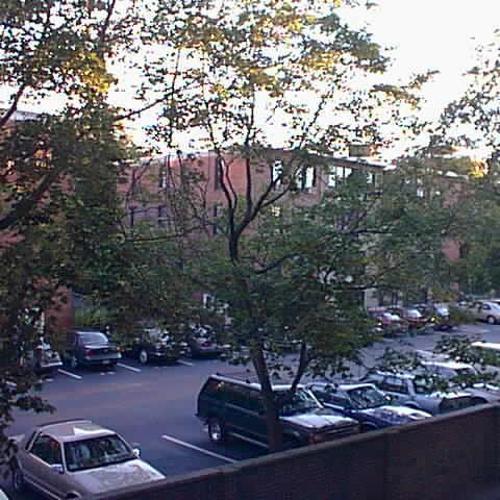

Supplement: Supplementary file 3 [file Presentation_3.zip › Non-targets_1/image_0037.jpg]

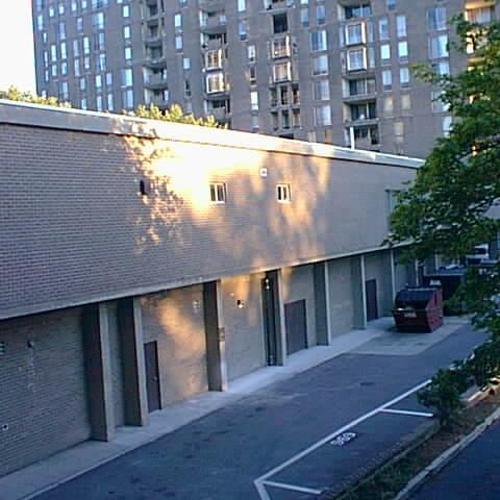

Supplement: Supplementary file 3 [file Presentation_3.zip › Non-targets_1/image_0038.jpg]

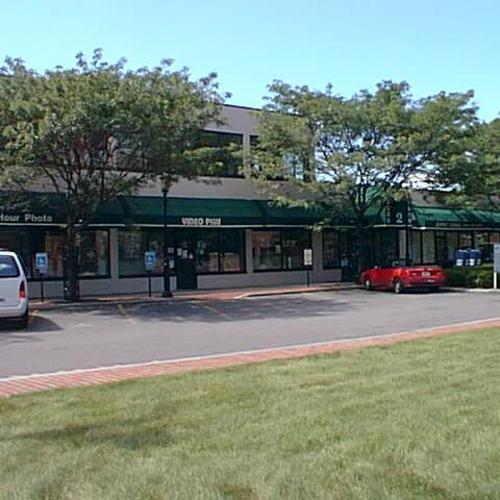

Supplement: Supplementary file 3 [file Presentation_3.zip › Non-targets_1/image_0039.jpg]

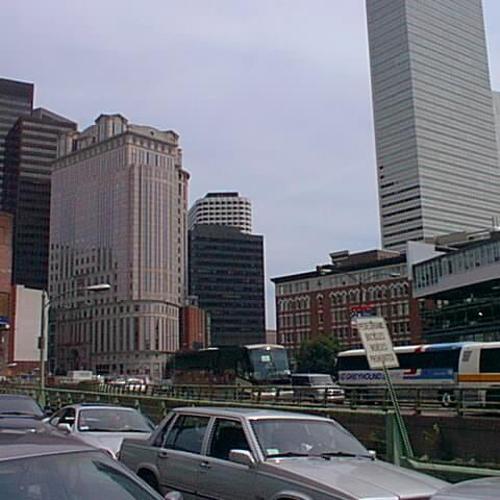

Supplement: Supplementary file 3 [file Presentation_3.zip › Non-targets_1/image_0040.jpg]

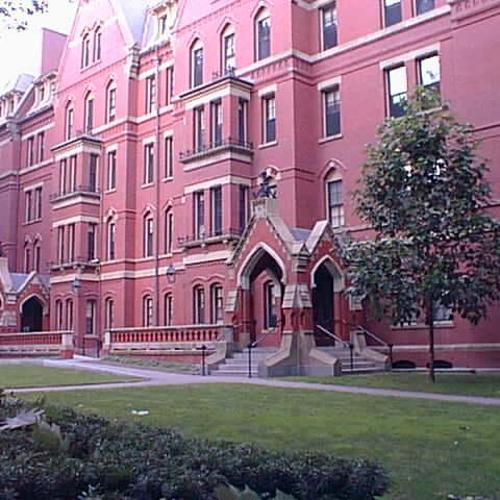

Supplement: Supplementary file 3 [file Presentation_3.zip › Non-targets_1/image_0041.jpg]

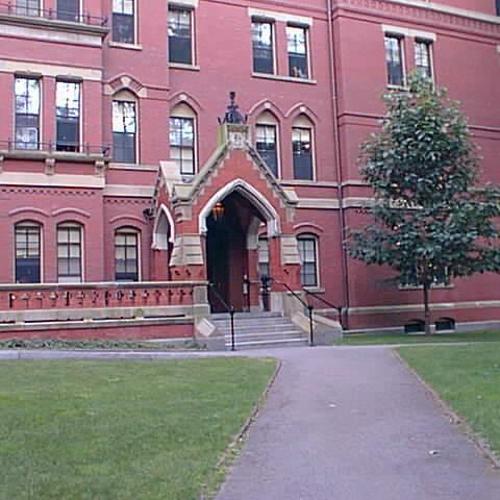

Supplement: Supplementary file 3 [file Presentation_3.zip › Non-targets_1/image_0042.jpg]

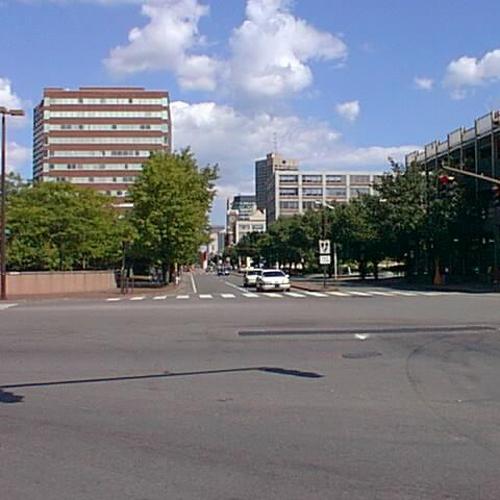

Supplement: Supplementary file 3 [file Presentation_3.zip › Non-targets_1/image_0043.jpg]

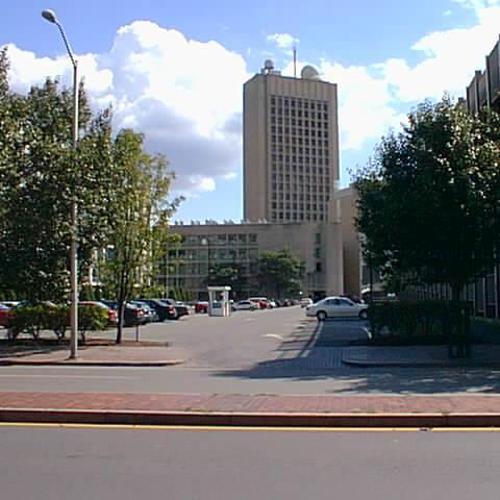

Supplement: Supplementary file 3 [file Presentation_3.zip › Non-targets_1/image_0044.jpg]

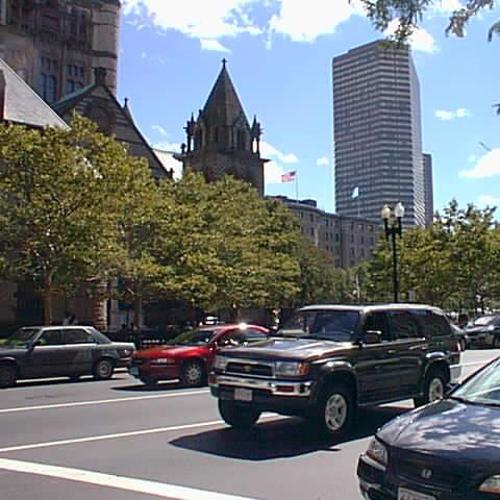

Supplement: Supplementary file 3 [file Presentation_3.zip › Non-targets_1/image_0045.jpg]
