# Supplementary figures and images for: A Cross-Session Dataset for Collaborative Brain-Computer Interfaces Based on Rapid Serial Visual Presentation (part 3 of 5)
Source: Front Neurosci. 2020 Oct 22;14:579469. doi: 10.3389/fnins.2020.579469 (PMC7642747; doi:10.3389/fnins.2020.579469)

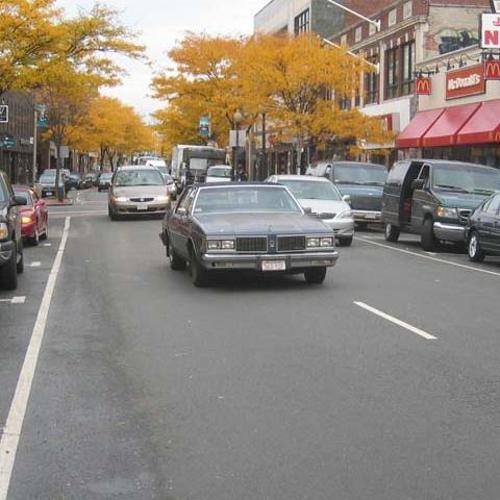

Supplement: Supplementary file 3 [file Presentation_3.zip › Non-targets_1/image_0146.jpg]

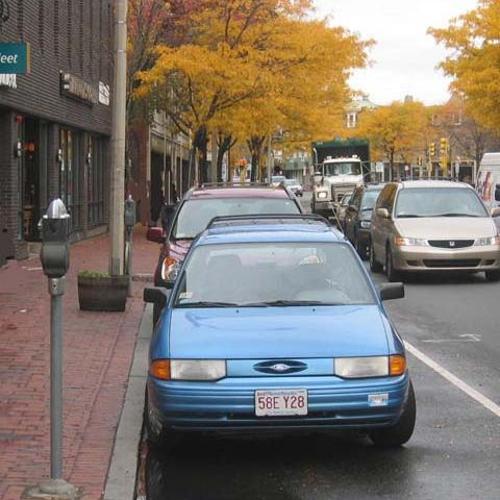

Supplement: Supplementary file 3 [file Presentation_3.zip › Non-targets_1/image_0147.jpg]

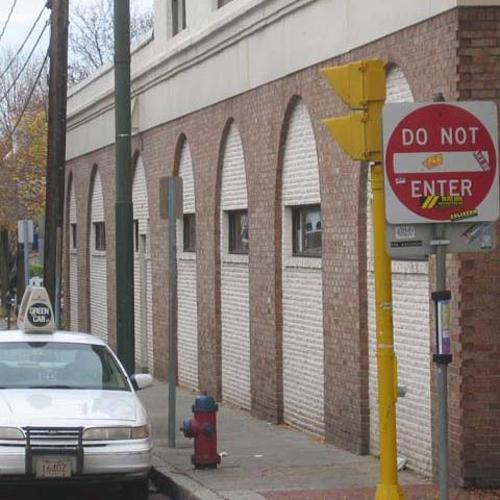

Supplement: Supplementary file 3 [file Presentation_3.zip › Non-targets_1/image_0148.jpg]

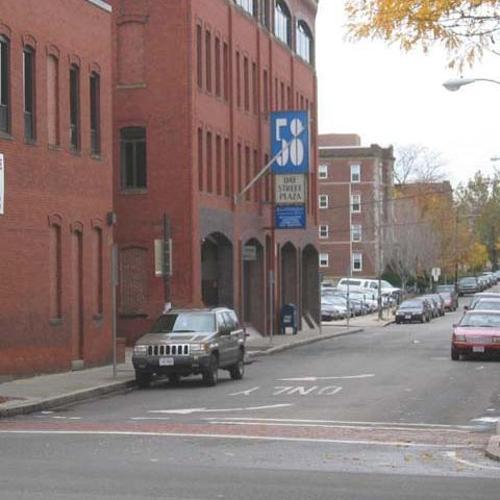

Supplement: Supplementary file 3 [file Presentation_3.zip › Non-targets_1/image_0149.jpg]

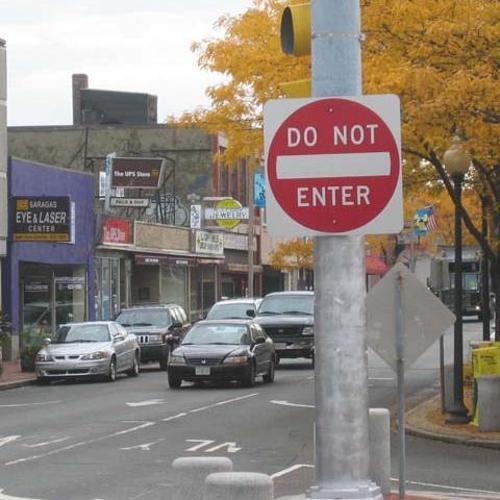

Supplement: Supplementary file 3 [file Presentation_3.zip › Non-targets_1/image_0150.jpg]

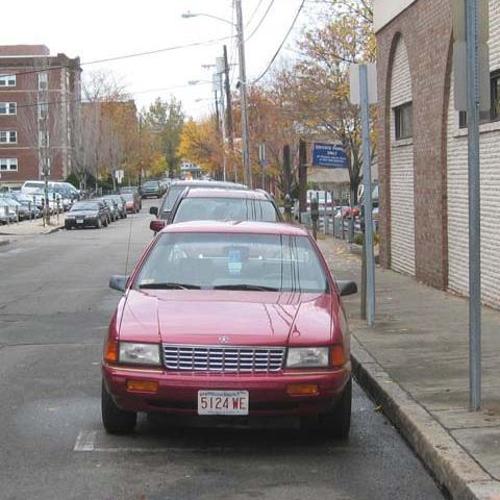

Supplement: Supplementary file 3 [file Presentation_3.zip › Non-targets_1/image_0151.jpg]

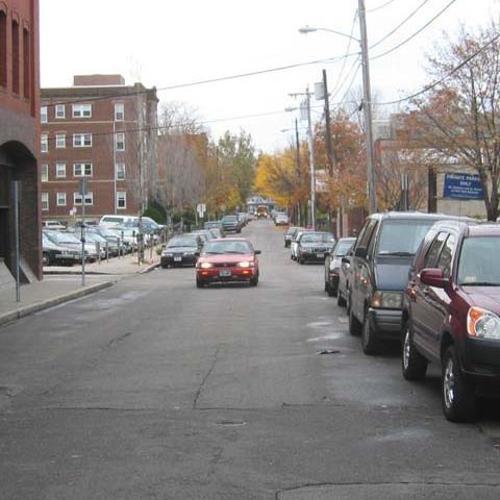

Supplement: Supplementary file 3 [file Presentation_3.zip › Non-targets_1/image_0152.jpg]

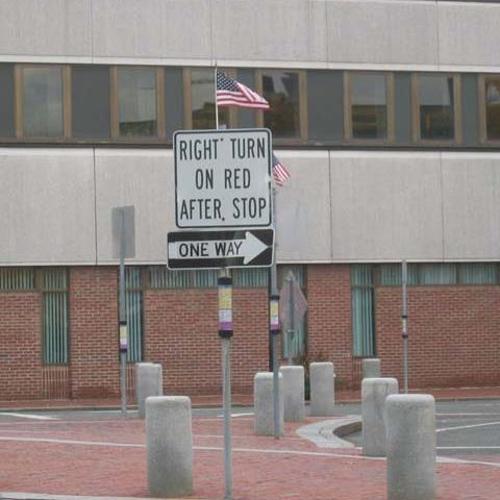

Supplement: Supplementary file 3 [file Presentation_3.zip › Non-targets_1/image_0153.jpg]

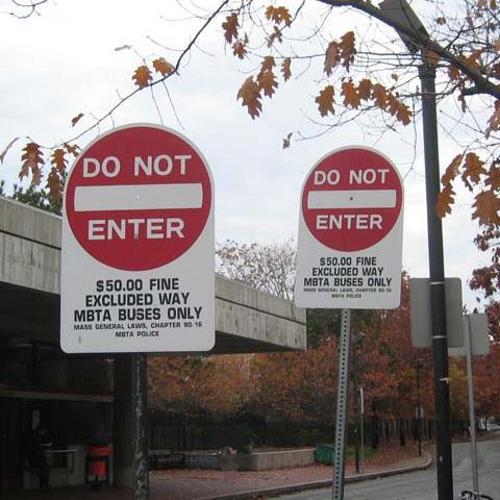

Supplement: Supplementary file 3 [file Presentation_3.zip › Non-targets_1/image_0154.jpg]

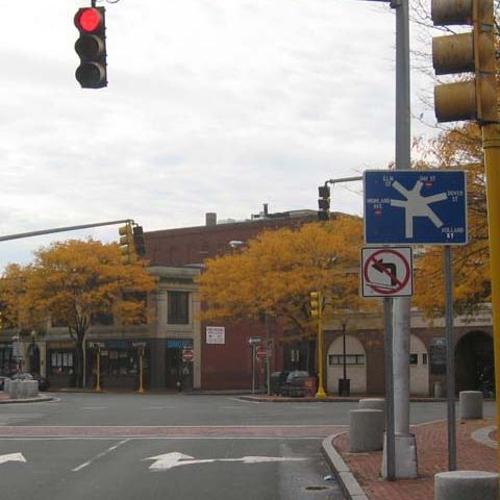

Supplement: Supplementary file 3 [file Presentation_3.zip › Non-targets_1/image_0155.jpg]

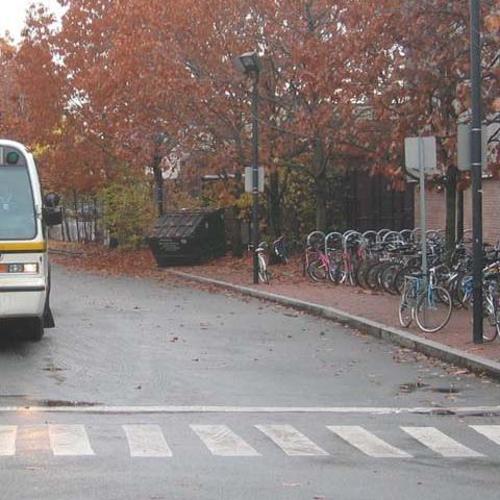

Supplement: Supplementary file 3 [file Presentation_3.zip › Non-targets_1/image_0156.jpg]

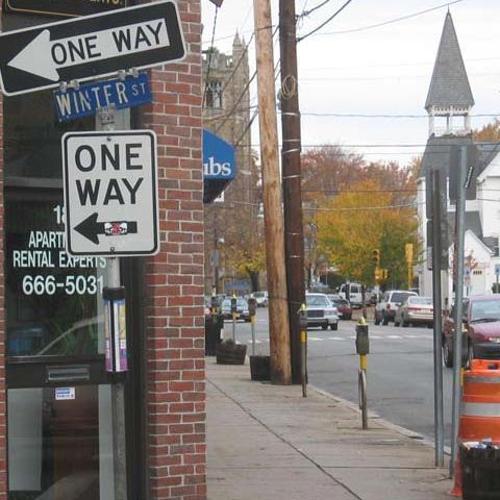

Supplement: Supplementary file 3 [file Presentation_3.zip › Non-targets_1/image_0157.jpg]

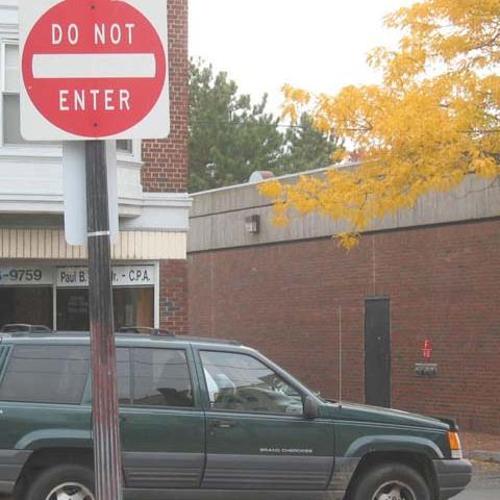

Supplement: Supplementary file 3 [file Presentation_3.zip › Non-targets_1/image_0158.jpg]

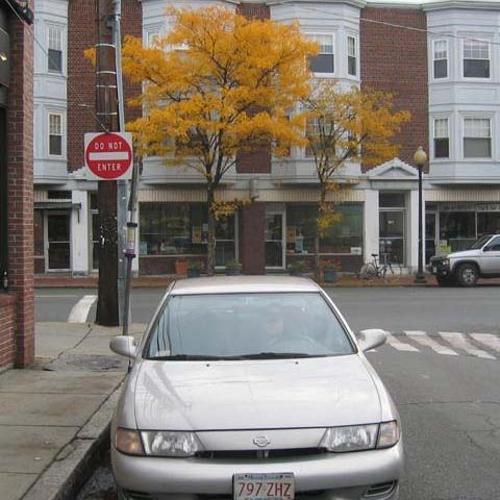

Supplement: Supplementary file 3 [file Presentation_3.zip › Non-targets_1/image_0159.jpg]

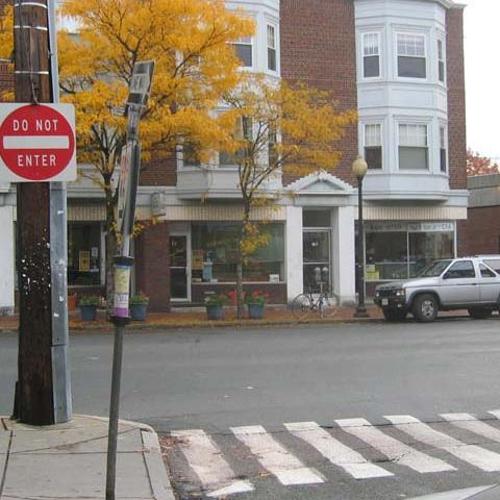

Supplement: Supplementary file 3 [file Presentation_3.zip › Non-targets_1/image_0160.jpg]

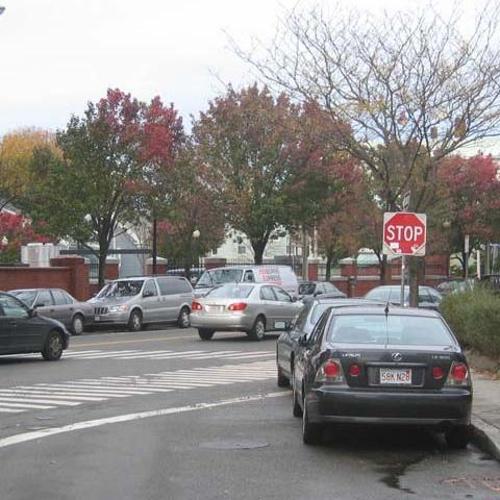

Supplement: Supplementary file 3 [file Presentation_3.zip › Non-targets_1/image_0161.jpg]

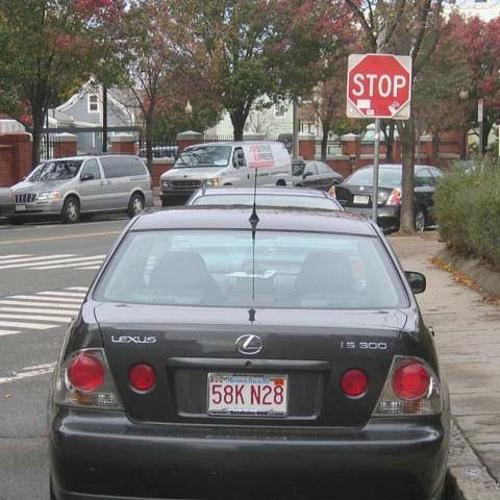

Supplement: Supplementary file 3 [file Presentation_3.zip › Non-targets_1/image_0162.jpg]

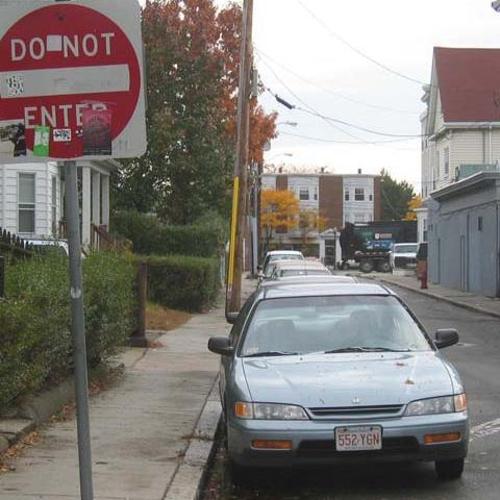

Supplement: Supplementary file 3 [file Presentation_3.zip › Non-targets_1/image_0163.jpg]

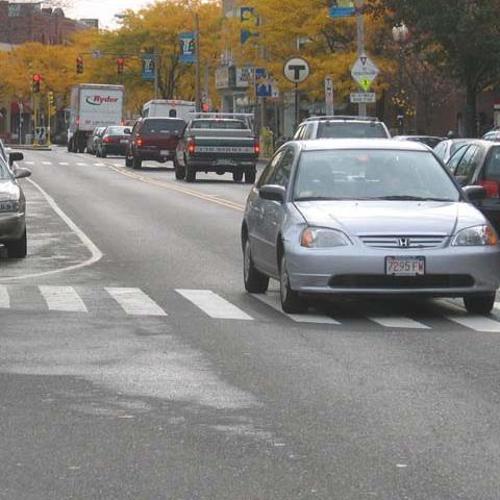

Supplement: Supplementary file 3 [file Presentation_3.zip › Non-targets_1/image_0164.jpg]

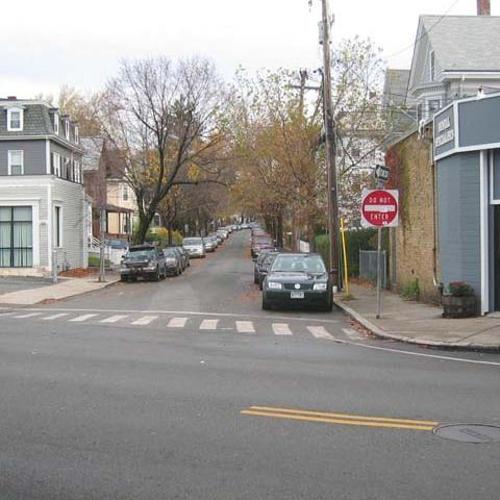

Supplement: Supplementary file 3 [file Presentation_3.zip › Non-targets_1/image_0165.jpg]

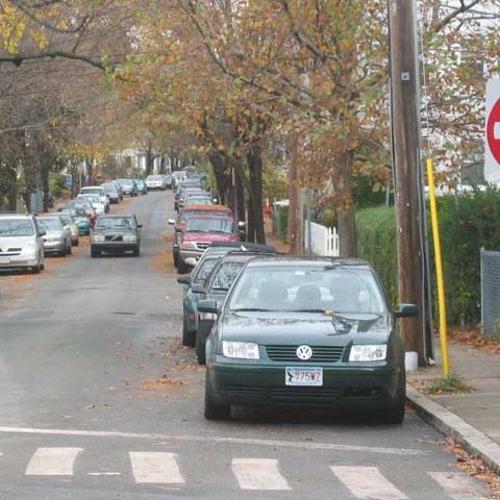

Supplement: Supplementary file 3 [file Presentation_3.zip › Non-targets_1/image_0166.jpg]

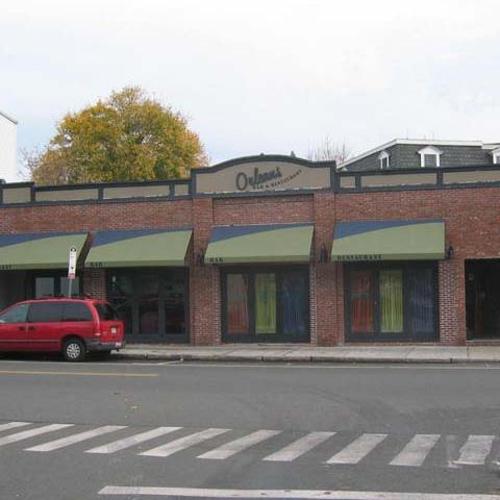

Supplement: Supplementary file 3 [file Presentation_3.zip › Non-targets_1/image_0167.jpg]

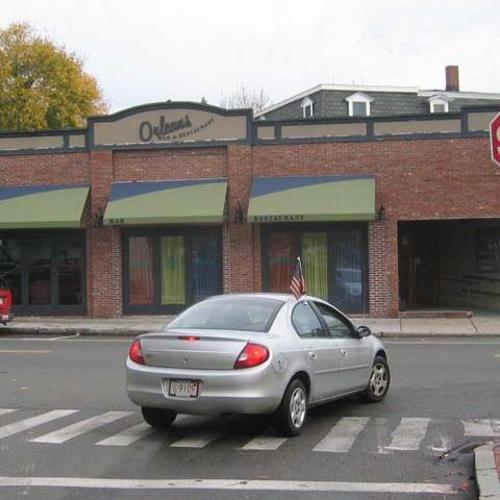

Supplement: Supplementary file 3 [file Presentation_3.zip › Non-targets_1/image_0168.jpg]

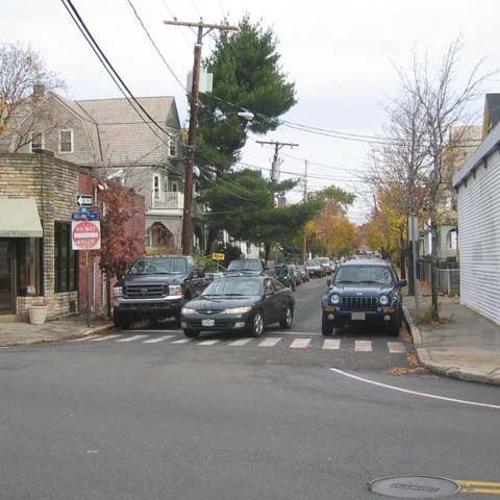

Supplement: Supplementary file 3 [file Presentation_3.zip › Non-targets_1/image_0169.jpg]

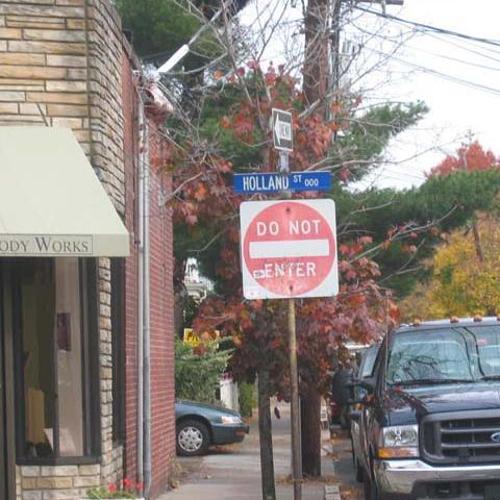

Supplement: Supplementary file 3 [file Presentation_3.zip › Non-targets_1/image_0170.jpg]

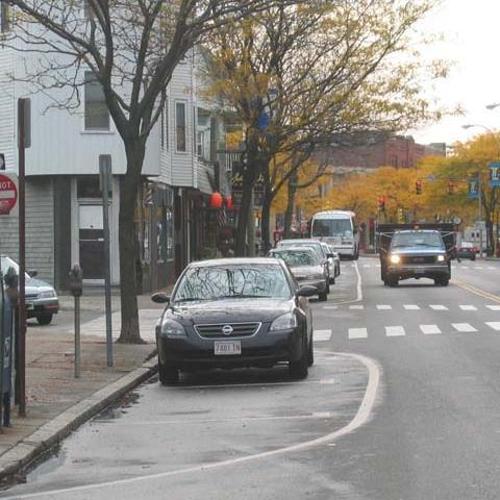

Supplement: Supplementary file 3 [file Presentation_3.zip › Non-targets_1/image_0171.jpg]

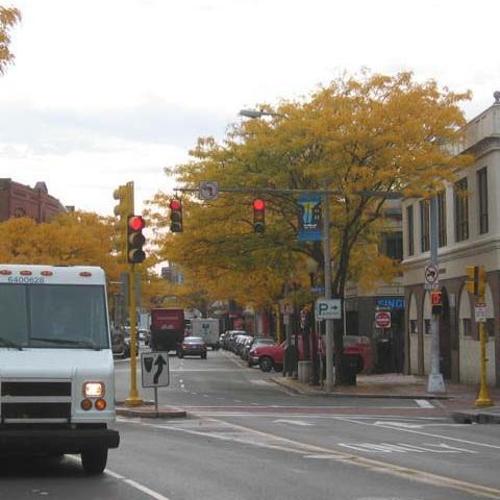

Supplement: Supplementary file 3 [file Presentation_3.zip › Non-targets_1/image_0172.jpg]

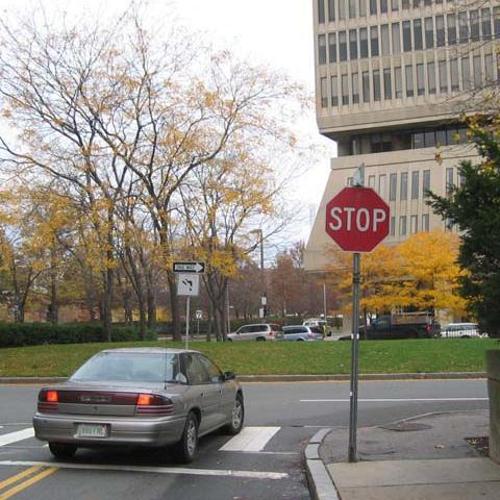

Supplement: Supplementary file 3 [file Presentation_3.zip › Non-targets_1/image_0173.jpg]

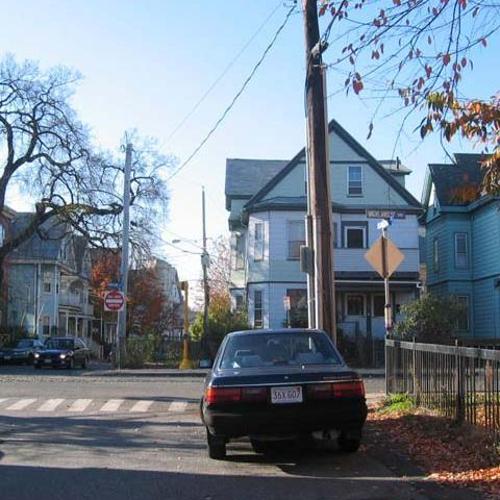

Supplement: Supplementary file 3 [file Presentation_3.zip › Non-targets_1/image_0174.jpg]

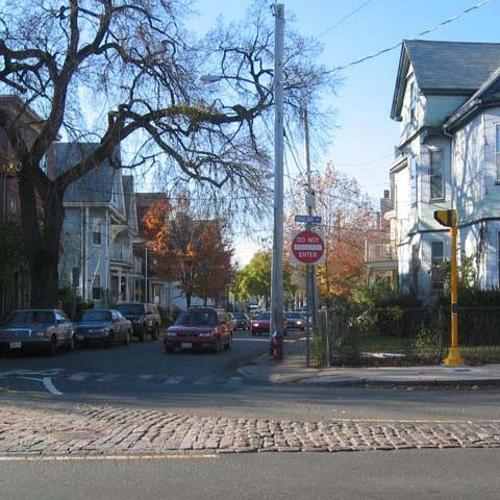

Supplement: Supplementary file 3 [file Presentation_3.zip › Non-targets_1/image_0175.jpg]

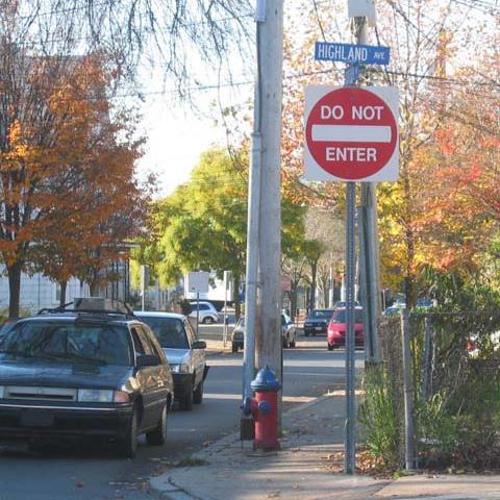

Supplement: Supplementary file 3 [file Presentation_3.zip › Non-targets_1/image_0176.jpg]

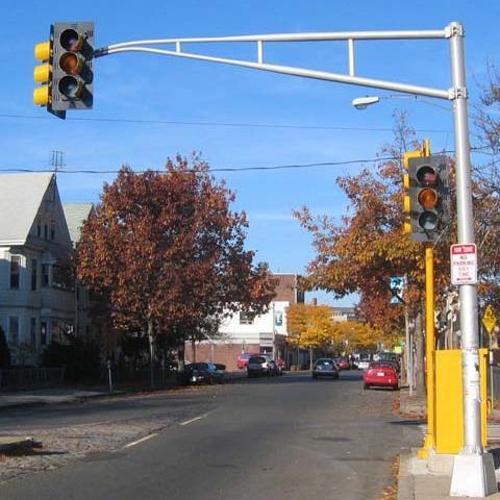

Supplement: Supplementary file 3 [file Presentation_3.zip › Non-targets_1/image_0177.jpg]

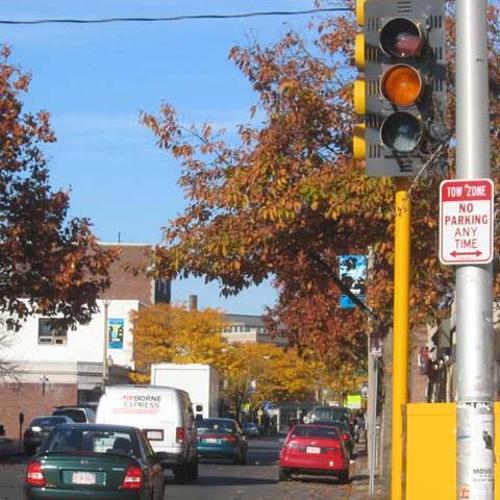

Supplement: Supplementary file 3 [file Presentation_3.zip › Non-targets_1/image_0178.jpg]

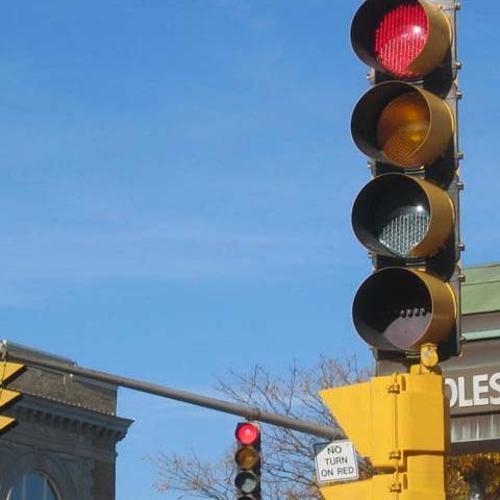

Supplement: Supplementary file 3 [file Presentation_3.zip › Non-targets_1/image_0179.jpg]

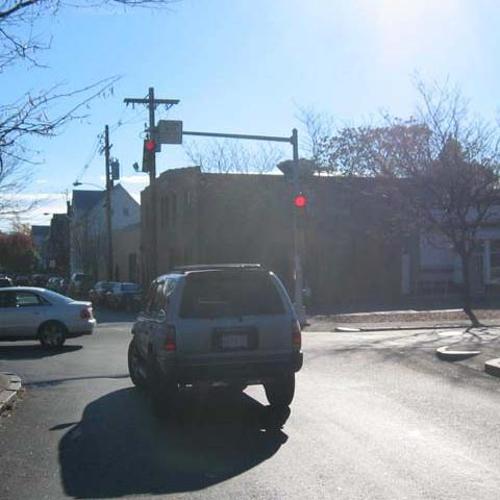

Supplement: Supplementary file 3 [file Presentation_3.zip › Non-targets_1/image_0180.jpg]

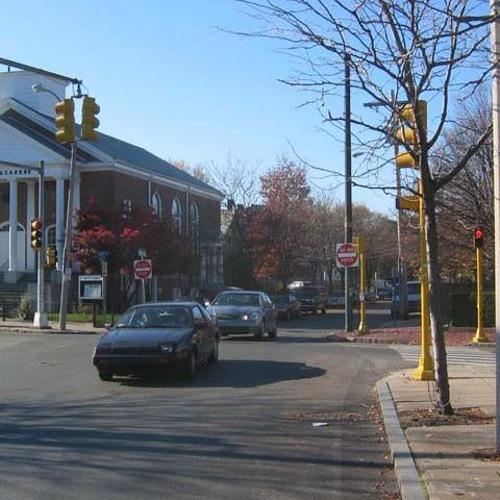

Supplement: Supplementary file 3 [file Presentation_3.zip › Non-targets_1/image_0181.jpg]

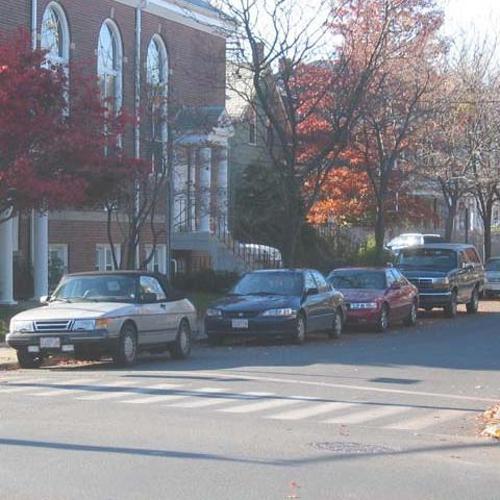

Supplement: Supplementary file 3 [file Presentation_3.zip › Non-targets_1/image_0182.jpg]

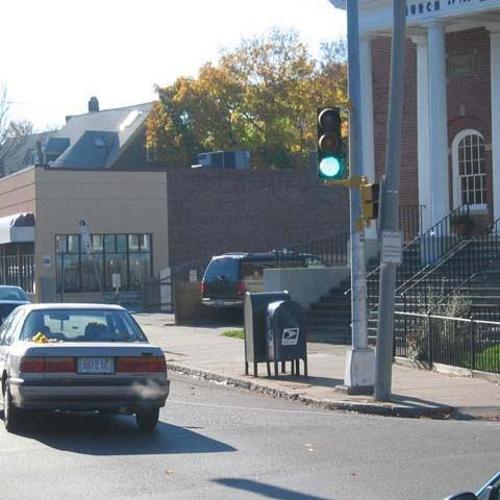

Supplement: Supplementary file 3 [file Presentation_3.zip › Non-targets_1/image_0183.jpg]

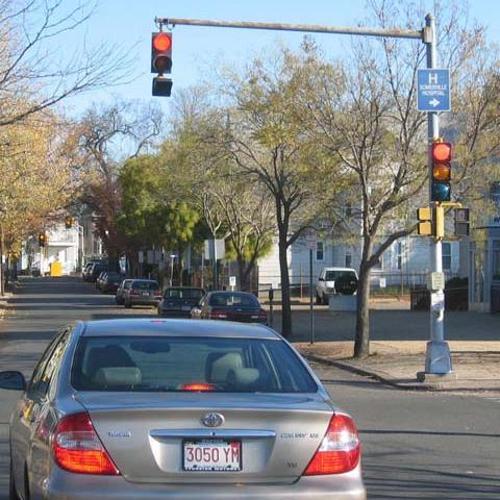

Supplement: Supplementary file 3 [file Presentation_3.zip › Non-targets_1/image_0184.jpg]

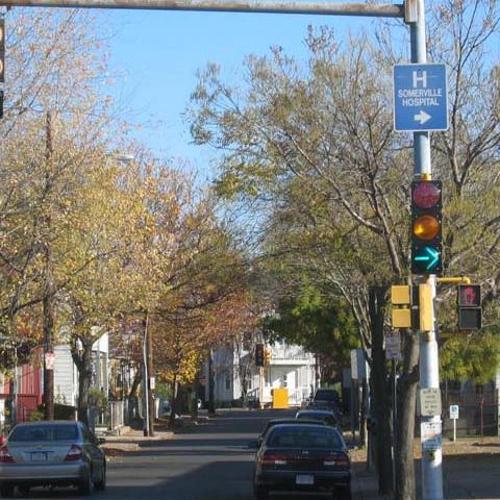

Supplement: Supplementary file 3 [file Presentation_3.zip › Non-targets_1/image_0185.jpg]

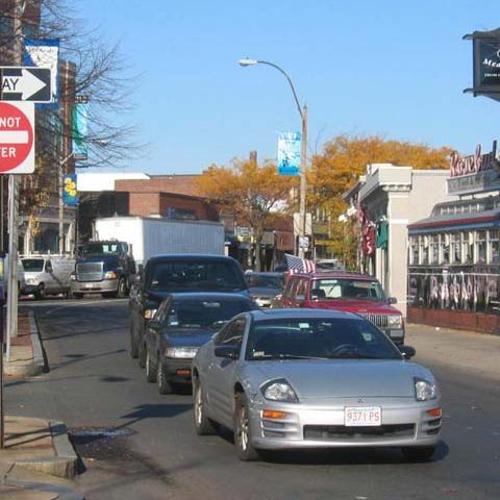

Supplement: Supplementary file 3 [file Presentation_3.zip › Non-targets_1/image_0186.jpg]

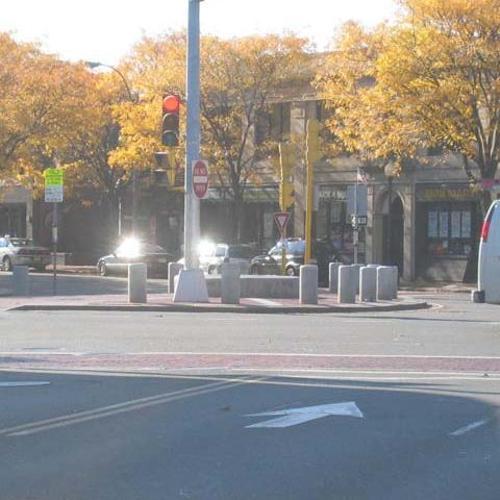

Supplement: Supplementary file 3 [file Presentation_3.zip › Non-targets_1/image_0187.jpg]

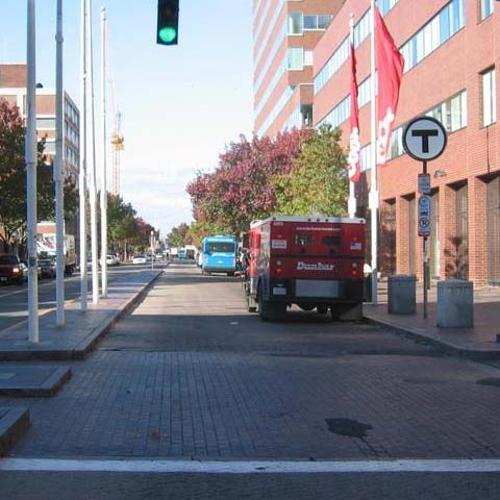

Supplement: Supplementary file 3 [file Presentation_3.zip › Non-targets_1/image_0188.jpg]

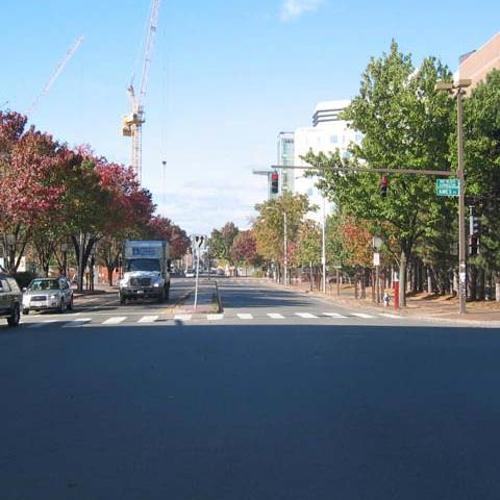

Supplement: Supplementary file 3 [file Presentation_3.zip › Non-targets_1/image_0189.jpg]

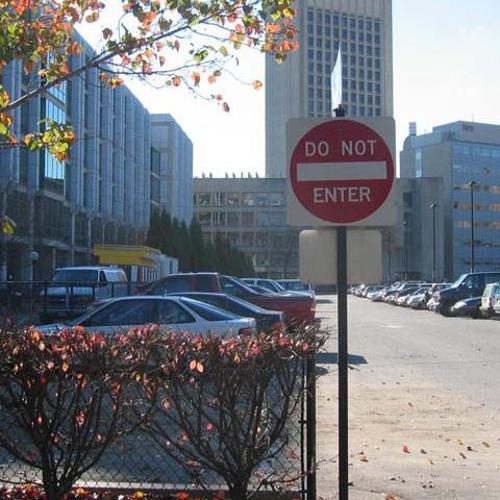

Supplement: Supplementary file 3 [file Presentation_3.zip › Non-targets_1/image_0190.jpg]

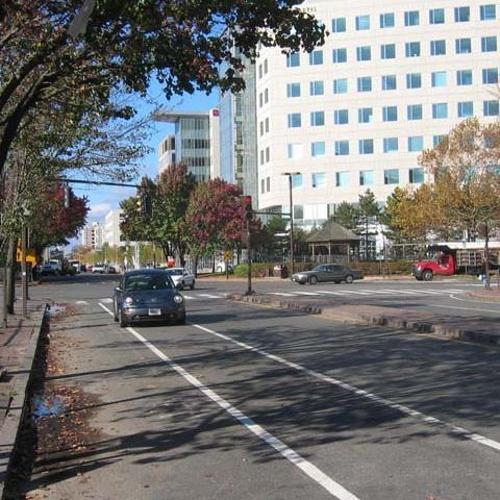

Supplement: Supplementary file 3 [file Presentation_3.zip › Non-targets_1/image_0191.jpg]

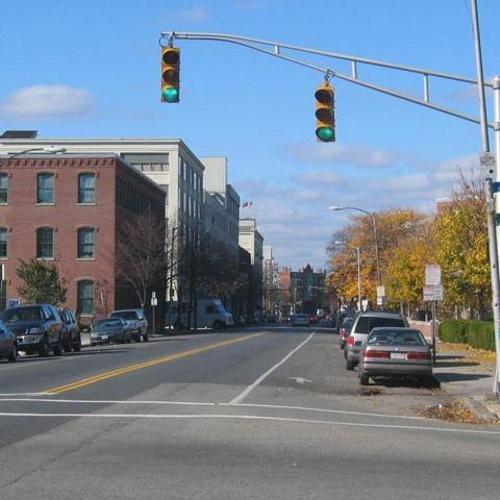

Supplement: Supplementary file 3 [file Presentation_3.zip › Non-targets_1/image_0192.jpg]

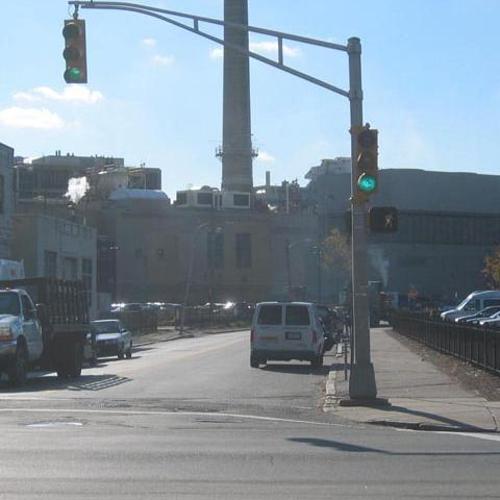

Supplement: Supplementary file 3 [file Presentation_3.zip › Non-targets_1/image_0193.jpg]

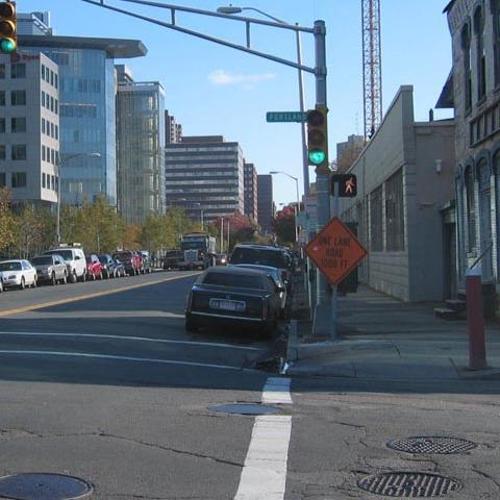

Supplement: Supplementary file 3 [file Presentation_3.zip › Non-targets_1/image_0194.jpg]

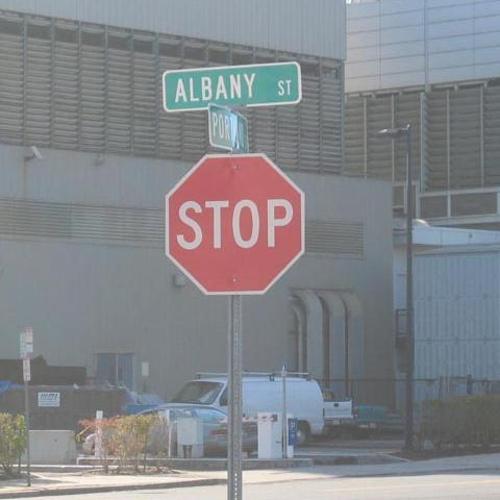

Supplement: Supplementary file 3 [file Presentation_3.zip › Non-targets_1/image_0195.jpg]

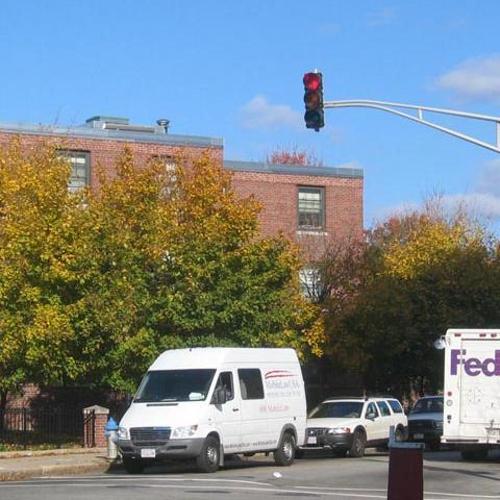

Supplement: Supplementary file 3 [file Presentation_3.zip › Non-targets_1/image_0196.jpg]

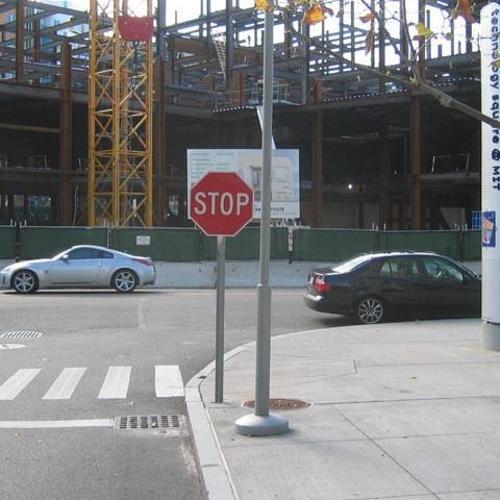

Supplement: Supplementary file 3 [file Presentation_3.zip › Non-targets_1/image_0197.jpg]

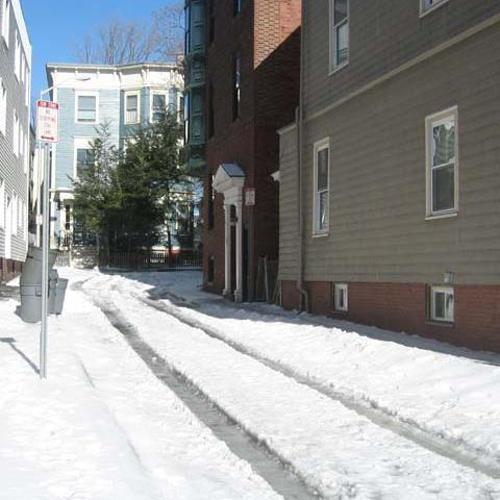

Supplement: Supplementary file 3 [file Presentation_3.zip › Non-targets_1/image_0198.jpg]

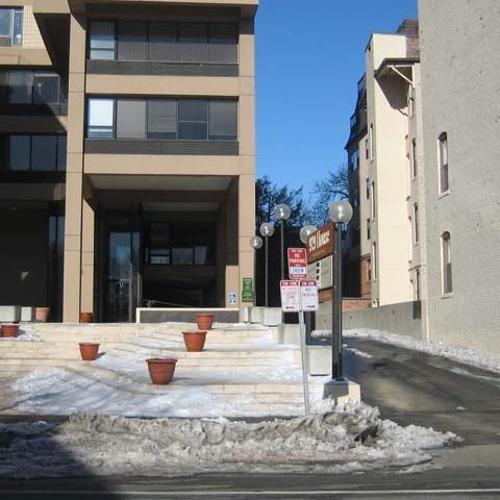

Supplement: Supplementary file 3 [file Presentation_3.zip › Non-targets_1/image_0199.jpg]

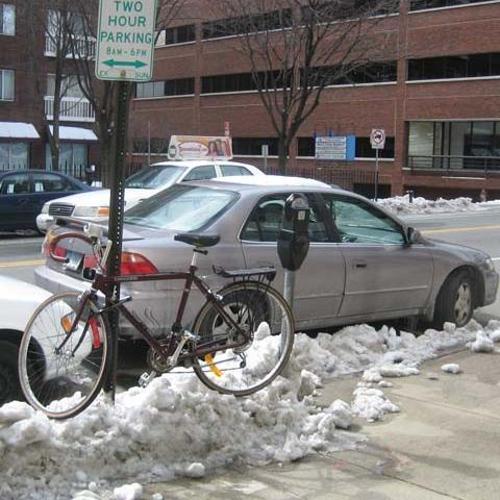

Supplement: Supplementary file 3 [file Presentation_3.zip › Non-targets_1/image_0200.jpg]

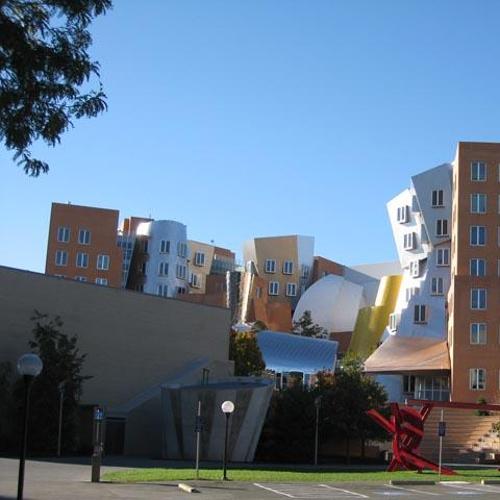

Supplement: Supplementary file 4 [file Presentation_4.zip › Non-targets_2/image_0701.jpg]

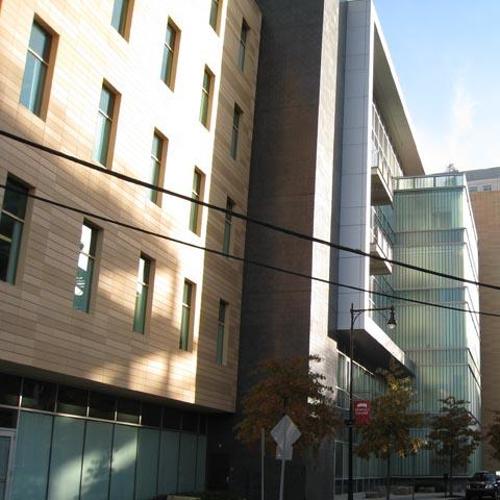

Supplement: Supplementary file 4 [file Presentation_4.zip › Non-targets_2/image_0702.jpg]

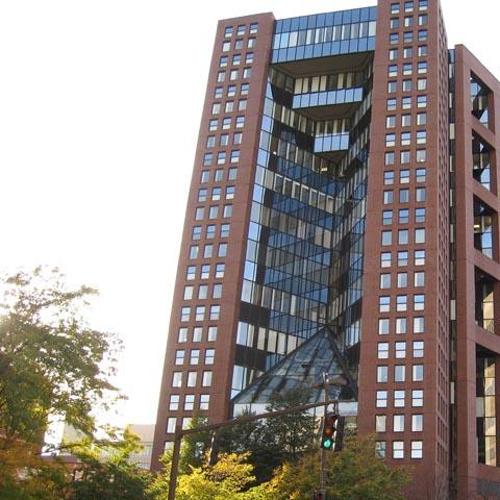

Supplement: Supplementary file 4 [file Presentation_4.zip › Non-targets_2/image_0703.jpg]

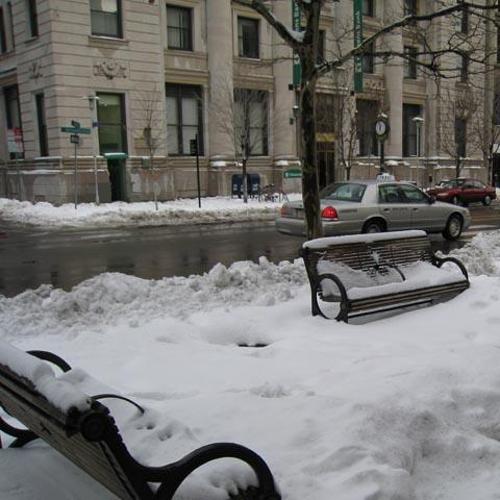

Supplement: Supplementary file 4 [file Presentation_4.zip › Non-targets_2/image_0704.jpg]

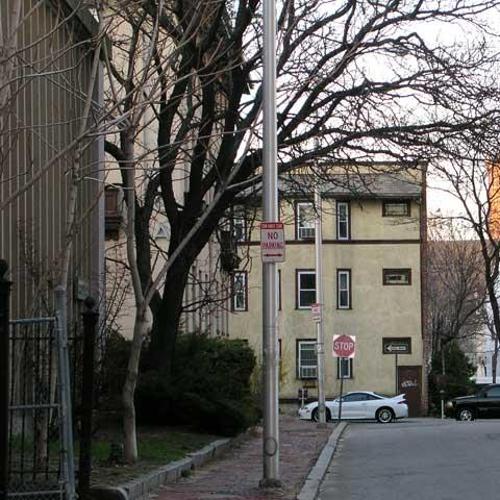

Supplement: Supplementary file 4 [file Presentation_4.zip › Non-targets_2/image_0705.jpg]

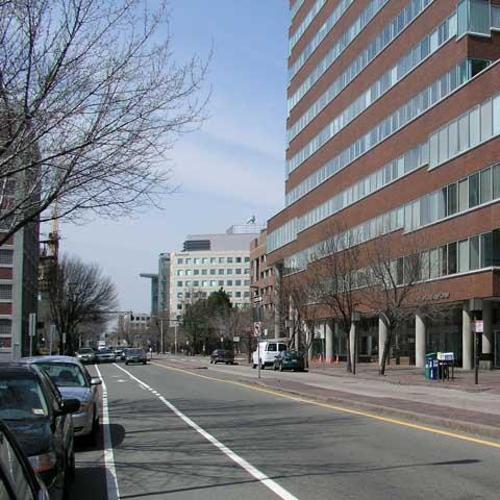

Supplement: Supplementary file 4 [file Presentation_4.zip › Non-targets_2/image_0706.jpg]

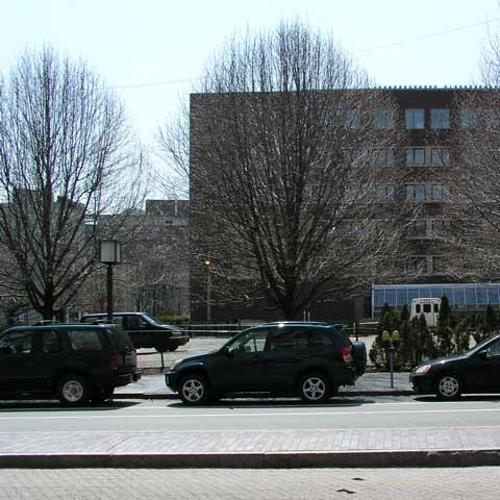

Supplement: Supplementary file 4 [file Presentation_4.zip › Non-targets_2/image_0707.jpg]

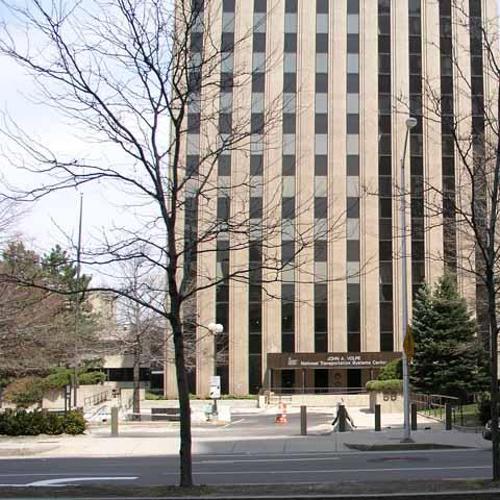

Supplement: Supplementary file 4 [file Presentation_4.zip › Non-targets_2/image_0708.jpg]

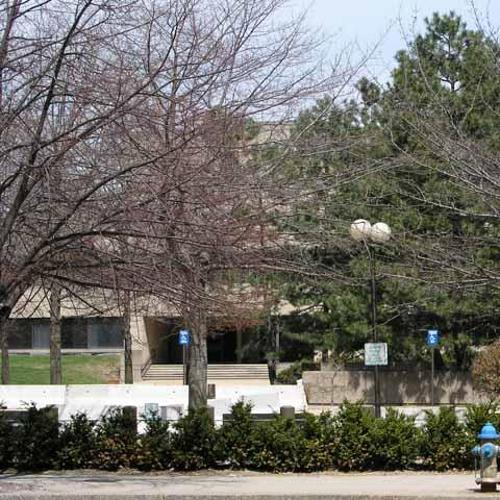

Supplement: Supplementary file 4 [file Presentation_4.zip › Non-targets_2/image_0709.jpg]

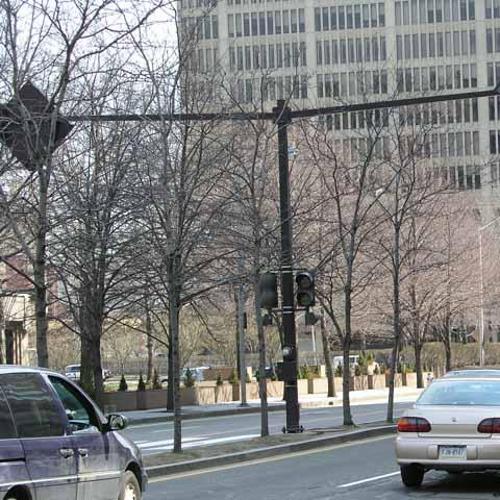

Supplement: Supplementary file 4 [file Presentation_4.zip › Non-targets_2/image_0710.jpg]

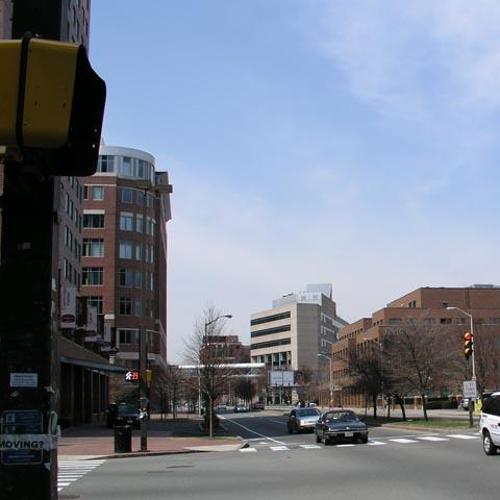

Supplement: Supplementary file 4 [file Presentation_4.zip › Non-targets_2/image_0711.jpg]

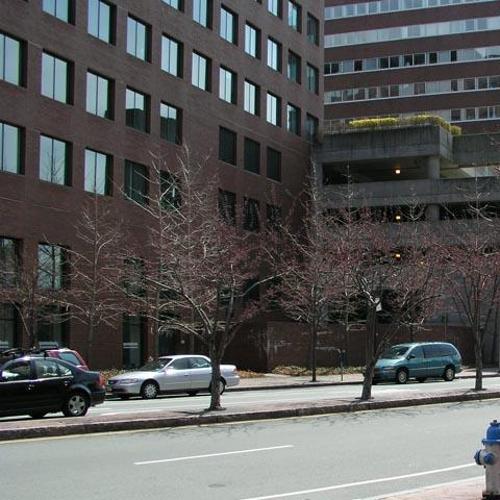

Supplement: Supplementary file 4 [file Presentation_4.zip › Non-targets_2/image_0712.jpg]

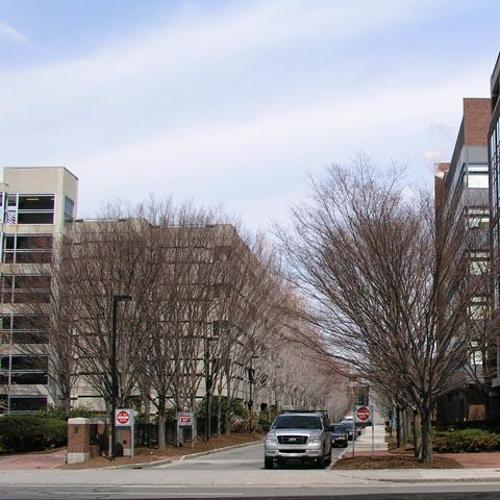

Supplement: Supplementary file 4 [file Presentation_4.zip › Non-targets_2/image_0713.jpg]

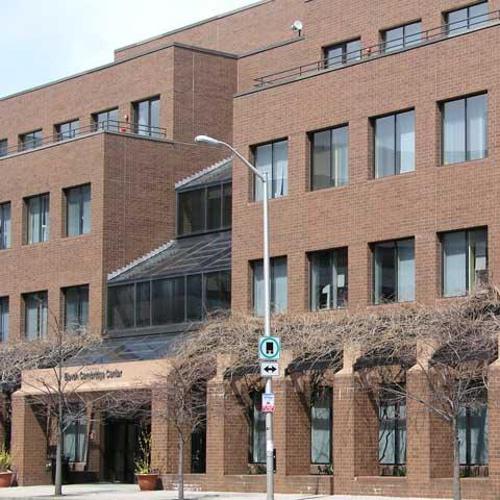

Supplement: Supplementary file 4 [file Presentation_4.zip › Non-targets_2/image_0714.jpg]

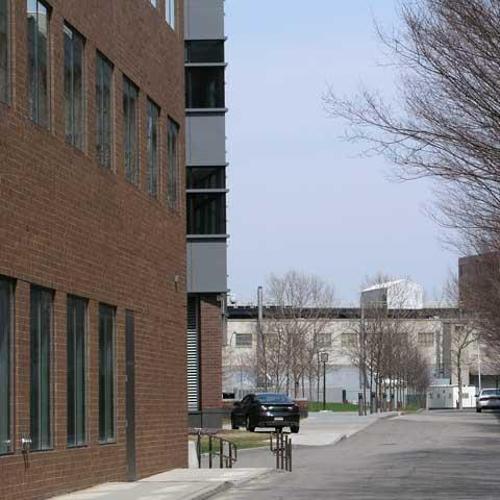

Supplement: Supplementary file 4 [file Presentation_4.zip › Non-targets_2/image_0715.jpg]

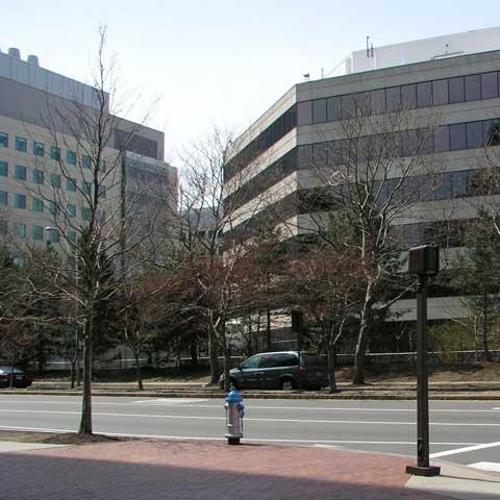

Supplement: Supplementary file 4 [file Presentation_4.zip › Non-targets_2/image_0716.jpg]

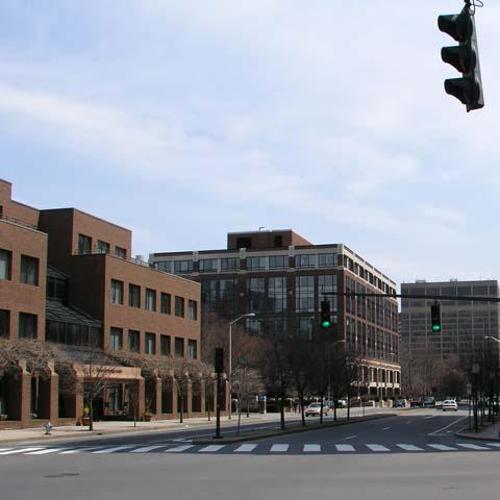

Supplement: Supplementary file 4 [file Presentation_4.zip › Non-targets_2/image_0717.jpg]

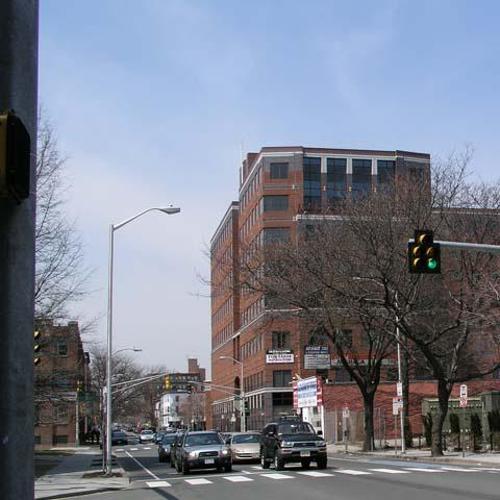

Supplement: Supplementary file 4 [file Presentation_4.zip › Non-targets_2/image_0718.jpg]

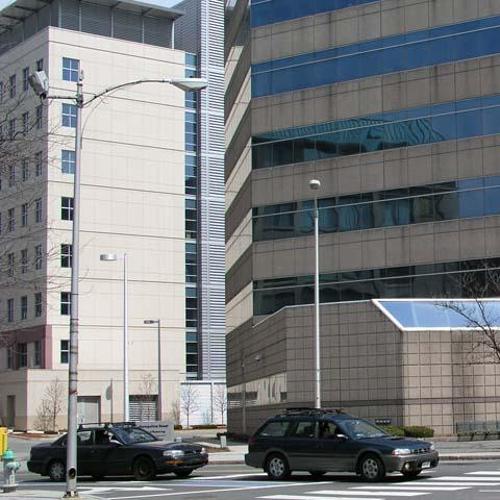

Supplement: Supplementary file 4 [file Presentation_4.zip › Non-targets_2/image_0719.jpg]

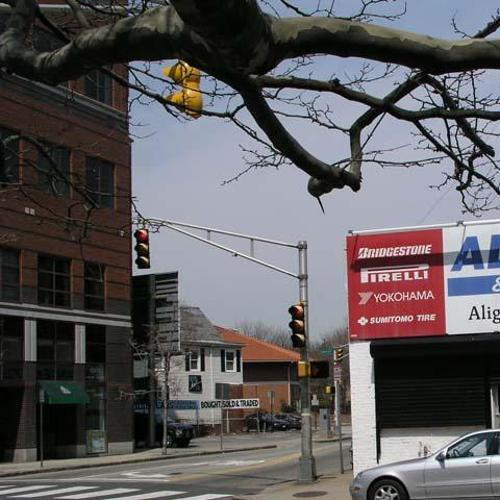

Supplement: Supplementary file 4 [file Presentation_4.zip › Non-targets_2/image_0720.jpg]

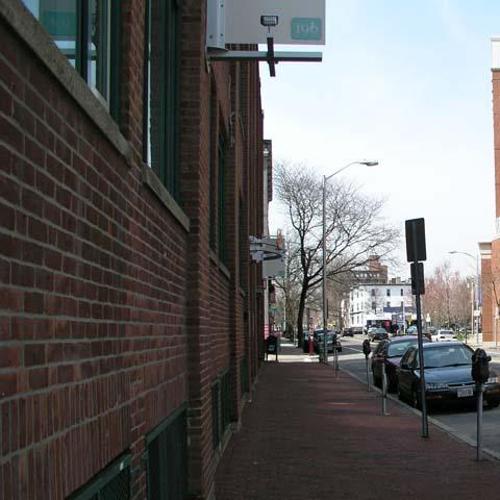

Supplement: Supplementary file 4 [file Presentation_4.zip › Non-targets_2/image_0721.jpg]

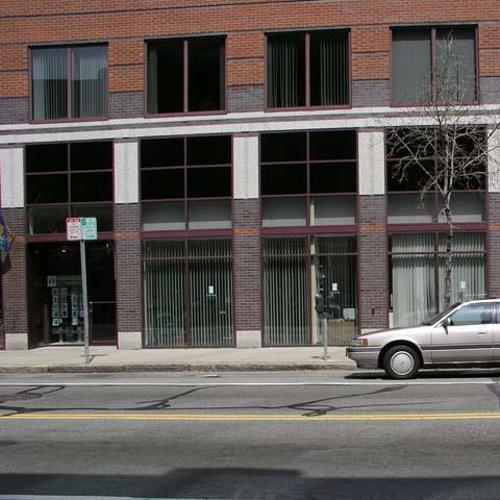

Supplement: Supplementary file 4 [file Presentation_4.zip › Non-targets_2/image_0722.jpg]

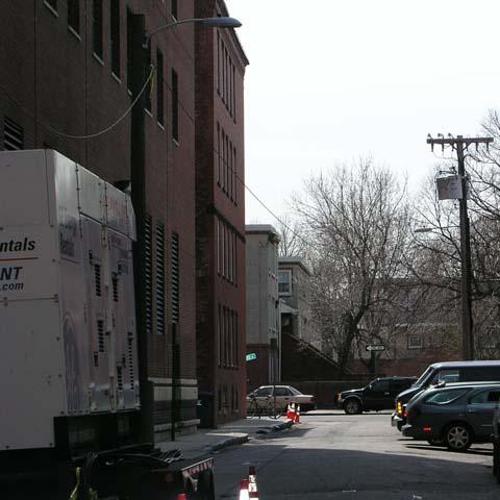

Supplement: Supplementary file 4 [file Presentation_4.zip › Non-targets_2/image_0723.jpg]

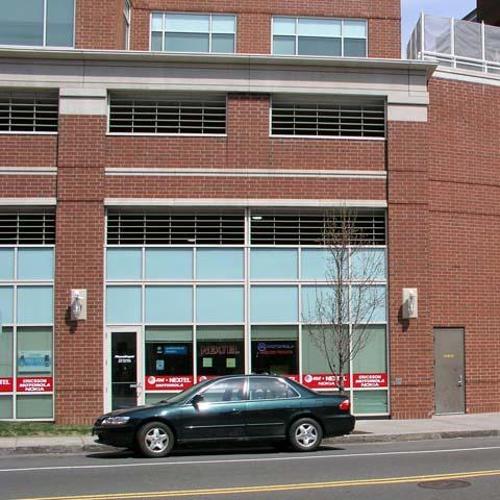

Supplement: Supplementary file 4 [file Presentation_4.zip › Non-targets_2/image_0724.jpg]

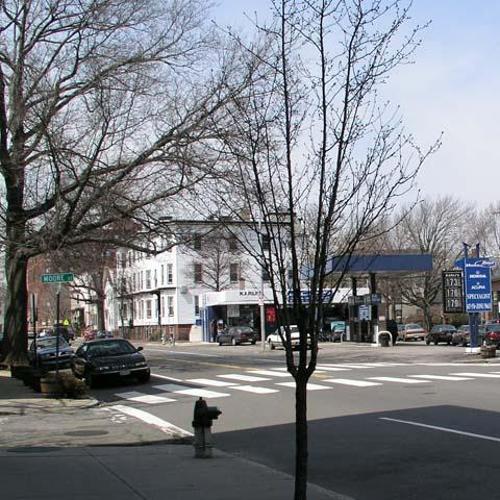

Supplement: Supplementary file 4 [file Presentation_4.zip › Non-targets_2/image_0725.jpg]

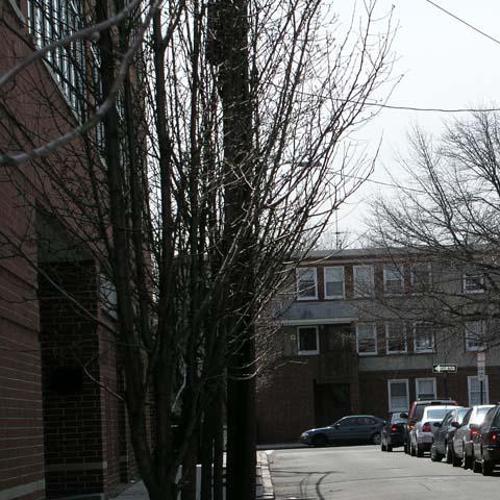

Supplement: Supplementary file 4 [file Presentation_4.zip › Non-targets_2/image_0726.jpg]

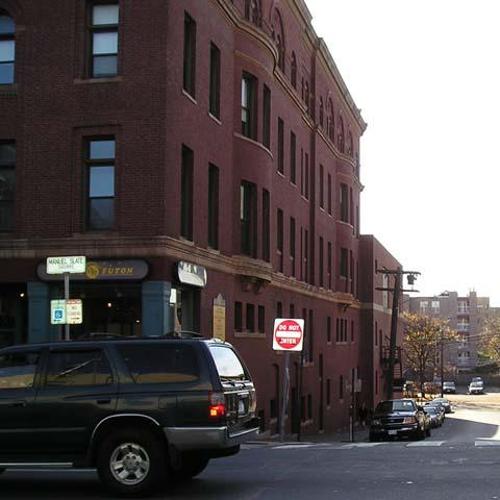

Supplement: Supplementary file 4 [file Presentation_4.zip › Non-targets_2/image_0727.jpg]

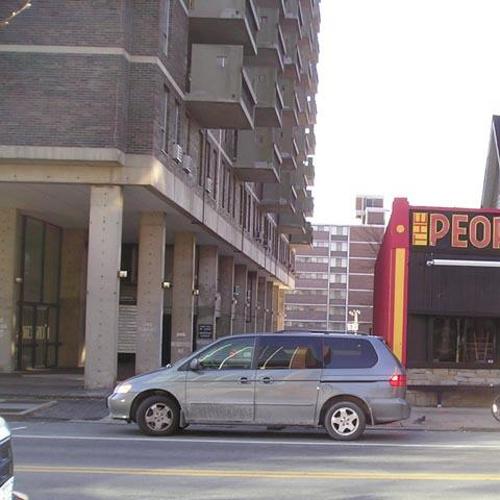

Supplement: Supplementary file 4 [file Presentation_4.zip › Non-targets_2/image_0728.jpg]

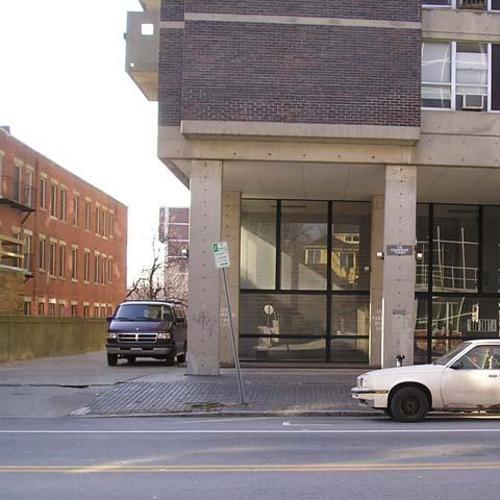

Supplement: Supplementary file 4 [file Presentation_4.zip › Non-targets_2/image_0729.jpg]

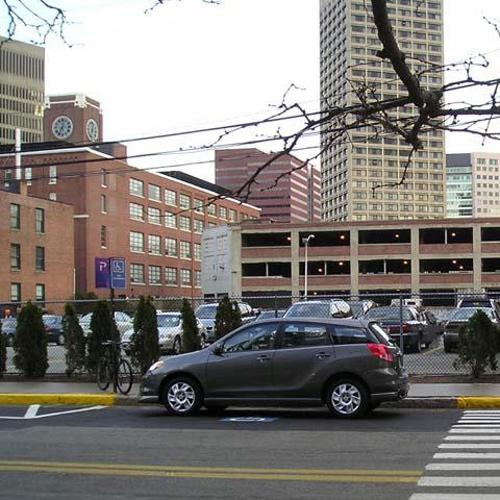

Supplement: Supplementary file 4 [file Presentation_4.zip › Non-targets_2/image_0730.jpg]

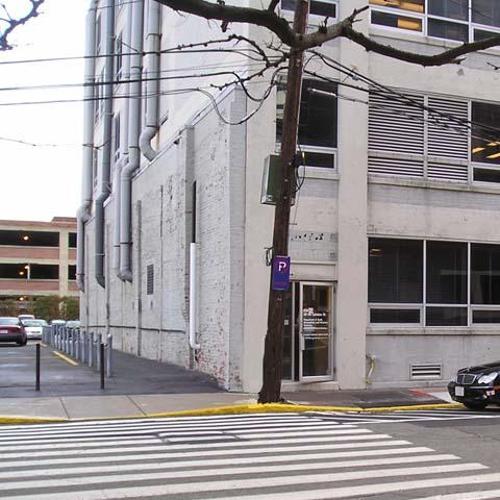

Supplement: Supplementary file 4 [file Presentation_4.zip › Non-targets_2/image_0731.jpg]

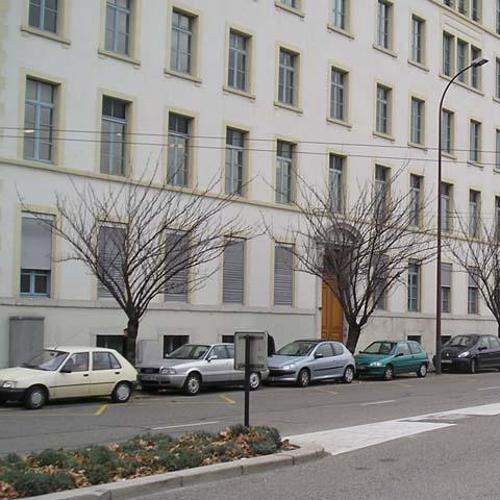

Supplement: Supplementary file 4 [file Presentation_4.zip › Non-targets_2/image_0732.jpg]

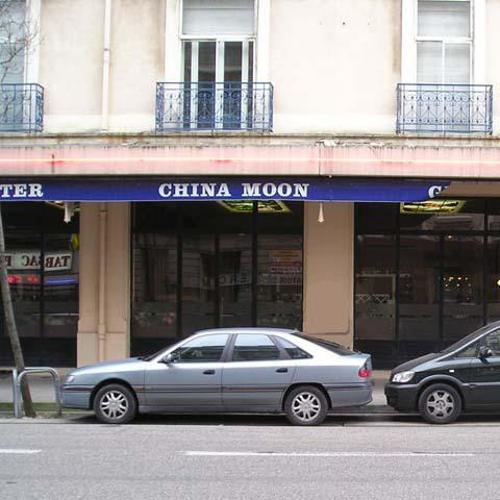

Supplement: Supplementary file 4 [file Presentation_4.zip › Non-targets_2/image_0733.jpg]

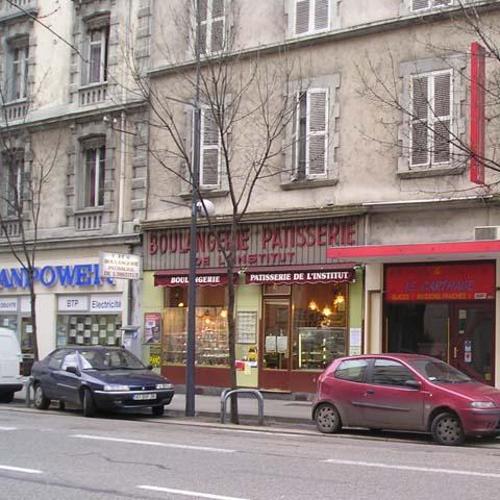

Supplement: Supplementary file 4 [file Presentation_4.zip › Non-targets_2/image_0734.jpg]

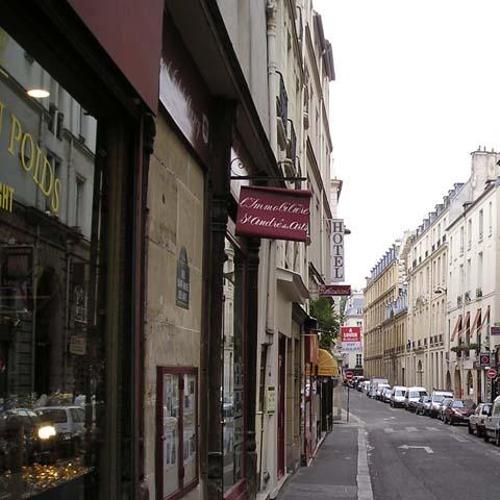

Supplement: Supplementary file 4 [file Presentation_4.zip › Non-targets_2/image_0735.jpg]

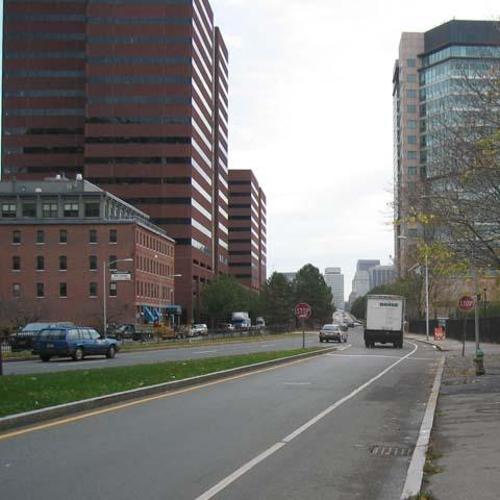

Supplement: Supplementary file 4 [file Presentation_4.zip › Non-targets_2/image_0736.jpg]

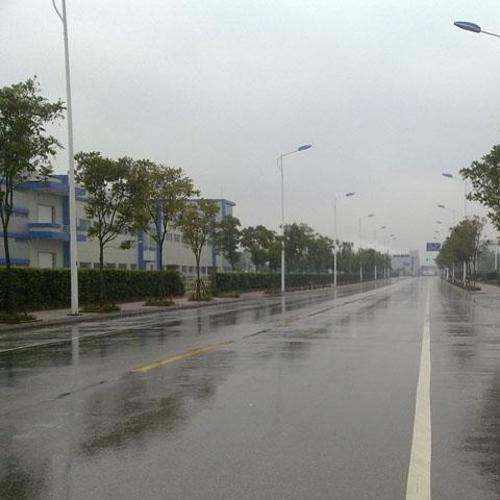

Supplement: Supplementary file 4 [file Presentation_4.zip › Non-targets_2/image_0737.jpg]

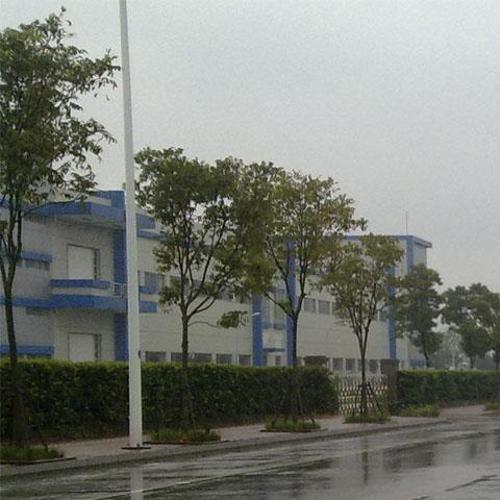

Supplement: Supplementary file 4 [file Presentation_4.zip › Non-targets_2/image_0738.jpg]

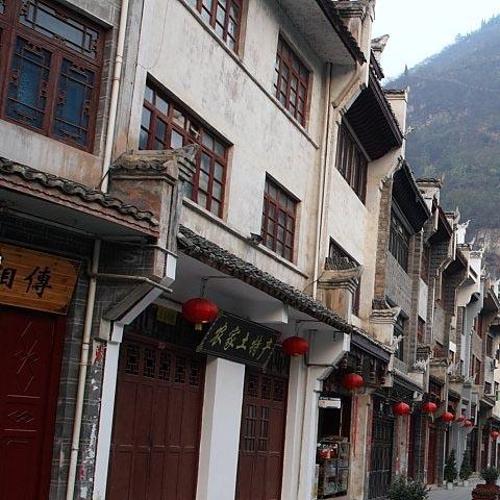

Supplement: Supplementary file 4 [file Presentation_4.zip › Non-targets_2/image_0739.jpg]

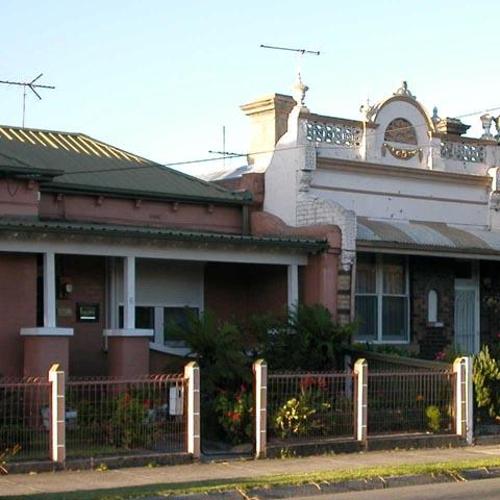

Supplement: Supplementary file 4 [file Presentation_4.zip › Non-targets_2/image_0740.jpg]

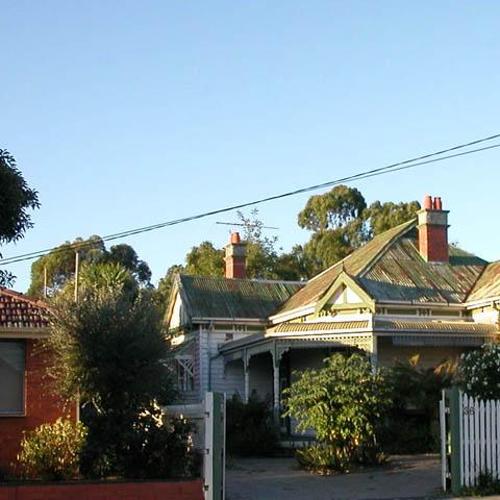

Supplement: Supplementary file 4 [file Presentation_4.zip › Non-targets_2/image_0741.jpg]

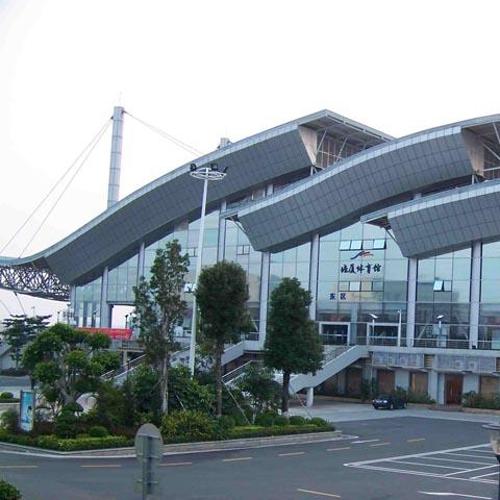

Supplement: Supplementary file 4 [file Presentation_4.zip › Non-targets_2/image_0742.jpg]

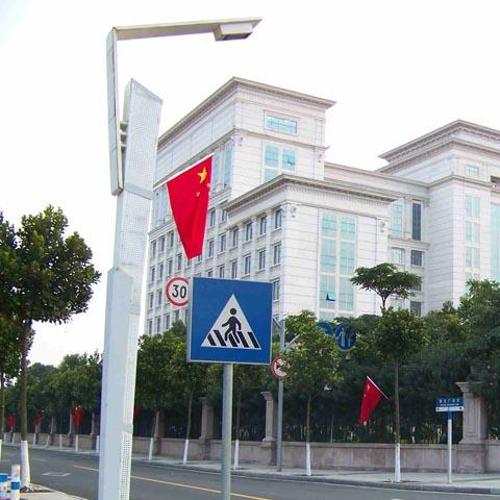

Supplement: Supplementary file 4 [file Presentation_4.zip › Non-targets_2/image_0743.jpg]

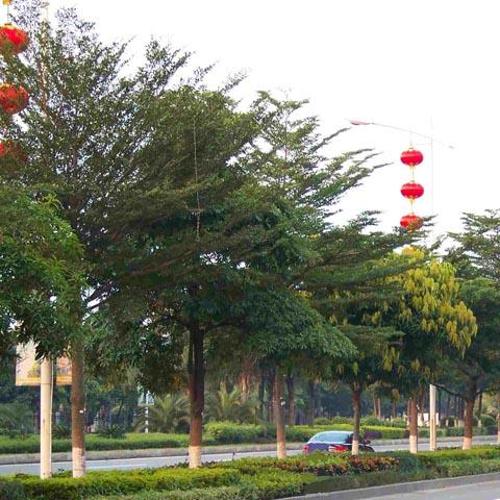

Supplement: Supplementary file 4 [file Presentation_4.zip › Non-targets_2/image_0744.jpg]

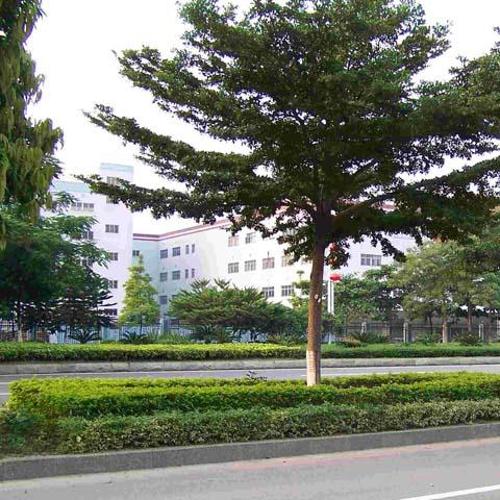

Supplement: Supplementary file 4 [file Presentation_4.zip › Non-targets_2/image_0745.jpg]
