# Supplementary figures and images for: A Cross-Session Dataset for Collaborative Brain-Computer Interfaces Based on Rapid Serial Visual Presentation (part 2 of 5)
Source: Front Neurosci. 2020 Oct 22;14:579469. doi: 10.3389/fnins.2020.579469 (PMC7642747; doi:10.3389/fnins.2020.579469)

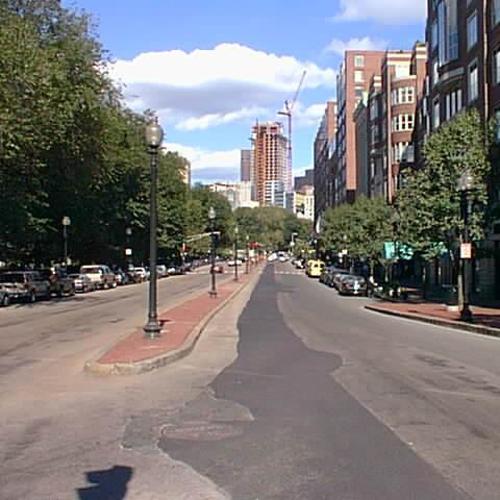

Supplement: Supplementary file 3 [file Presentation_3.zip › Non-targets_1/image_0046.jpg]

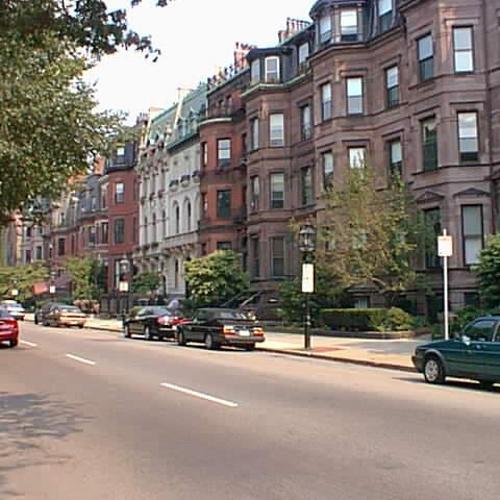

Supplement: Supplementary file 3 [file Presentation_3.zip › Non-targets_1/image_0047.jpg]

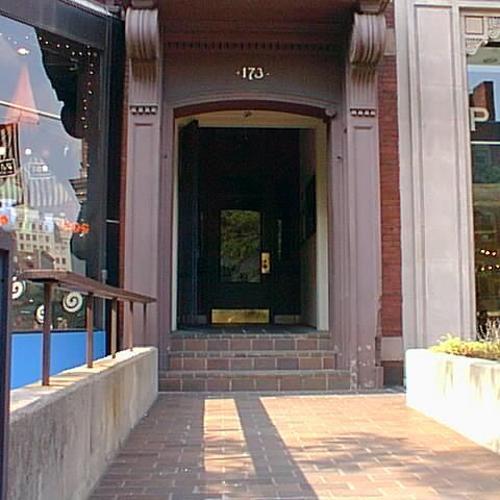

Supplement: Supplementary file 3 [file Presentation_3.zip › Non-targets_1/image_0048.jpg]

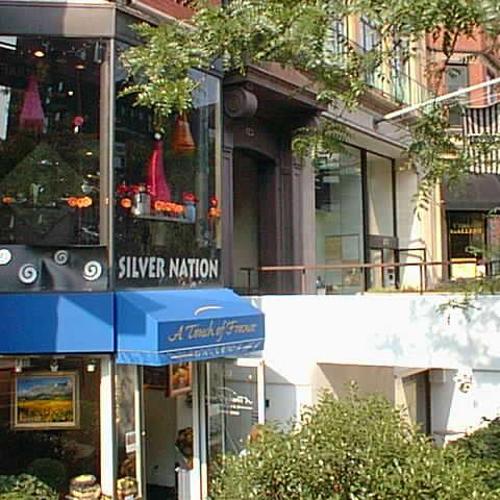

Supplement: Supplementary file 3 [file Presentation_3.zip › Non-targets_1/image_0049.jpg]

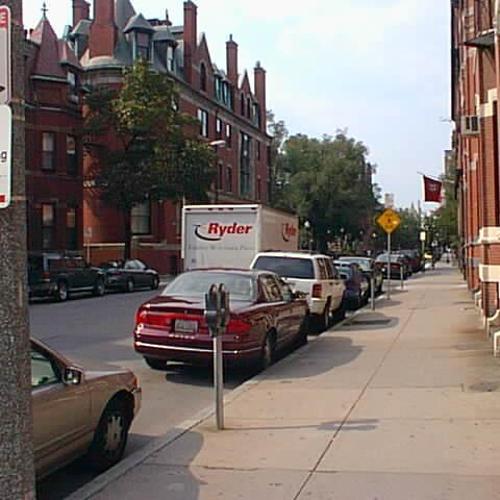

Supplement: Supplementary file 3 [file Presentation_3.zip › Non-targets_1/image_0050.jpg]

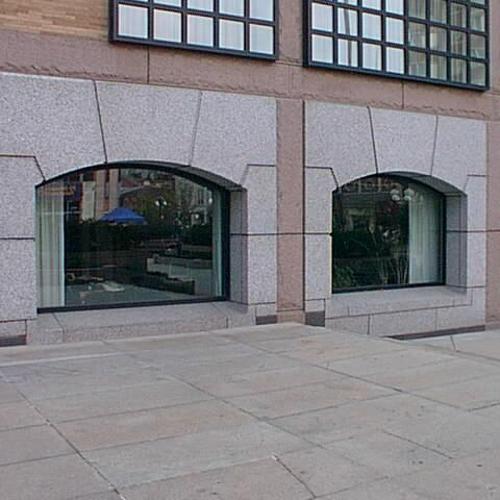

Supplement: Supplementary file 3 [file Presentation_3.zip › Non-targets_1/image_0051.jpg]

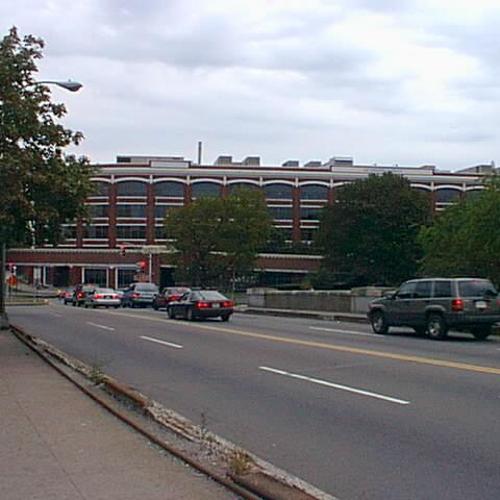

Supplement: Supplementary file 3 [file Presentation_3.zip › Non-targets_1/image_0052.jpg]

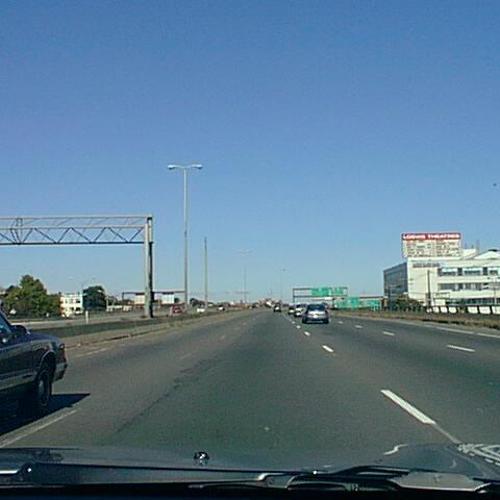

Supplement: Supplementary file 3 [file Presentation_3.zip › Non-targets_1/image_0053.jpg]

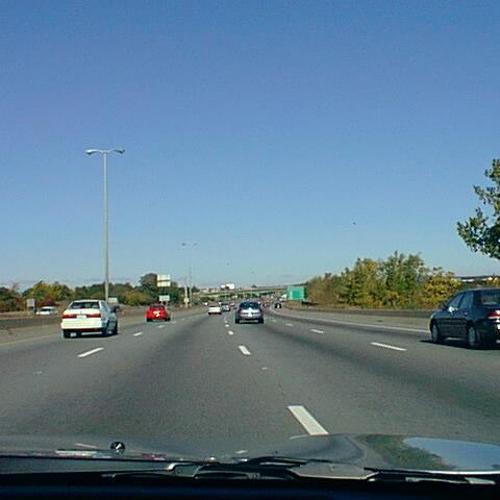

Supplement: Supplementary file 3 [file Presentation_3.zip › Non-targets_1/image_0054.jpg]

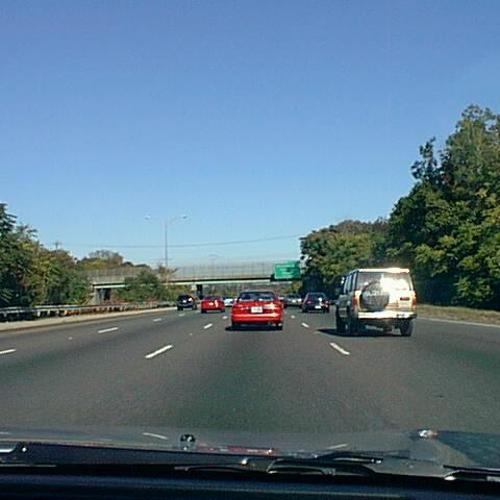

Supplement: Supplementary file 3 [file Presentation_3.zip › Non-targets_1/image_0055.jpg]

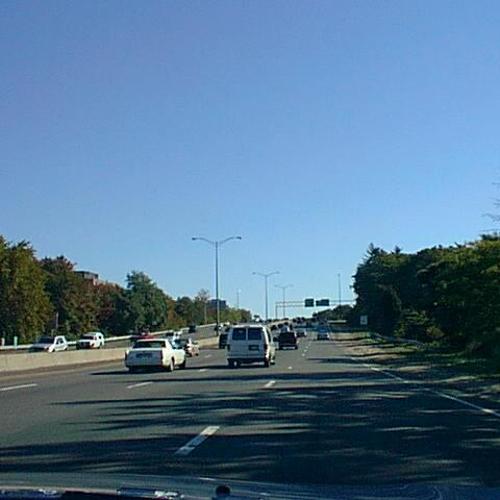

Supplement: Supplementary file 3 [file Presentation_3.zip › Non-targets_1/image_0056.jpg]

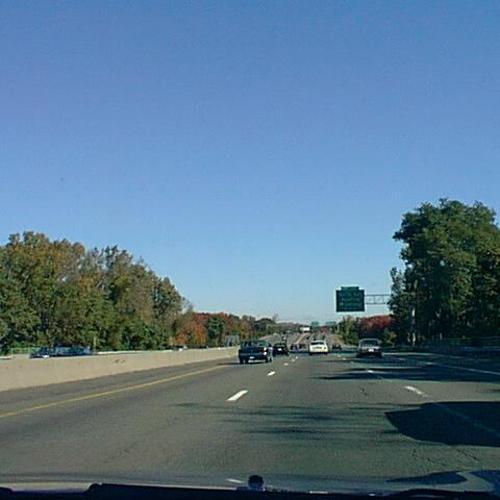

Supplement: Supplementary file 3 [file Presentation_3.zip › Non-targets_1/image_0057.jpg]

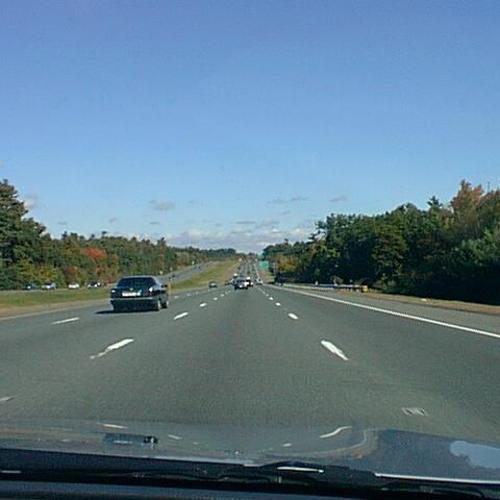

Supplement: Supplementary file 3 [file Presentation_3.zip › Non-targets_1/image_0058.jpg]

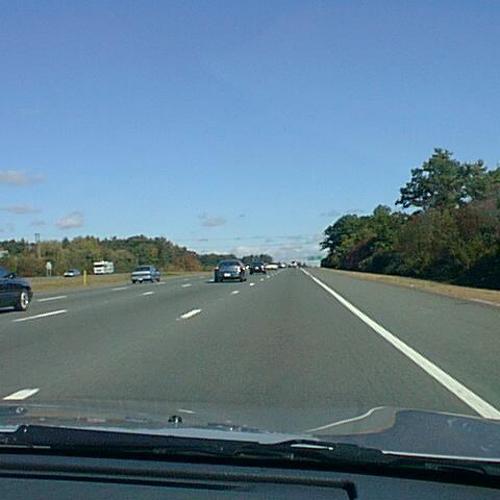

Supplement: Supplementary file 3 [file Presentation_3.zip › Non-targets_1/image_0059.jpg]

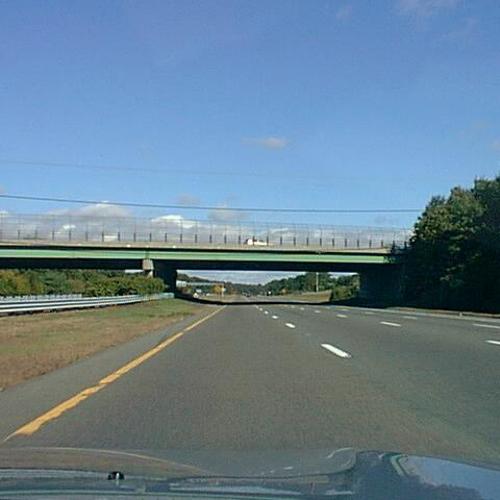

Supplement: Supplementary file 3 [file Presentation_3.zip › Non-targets_1/image_0060.jpg]

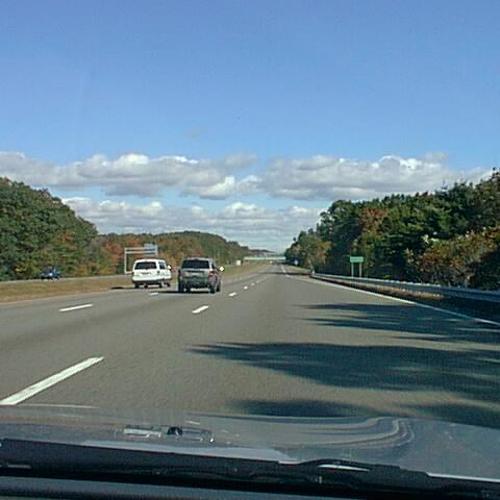

Supplement: Supplementary file 3 [file Presentation_3.zip › Non-targets_1/image_0061.jpg]

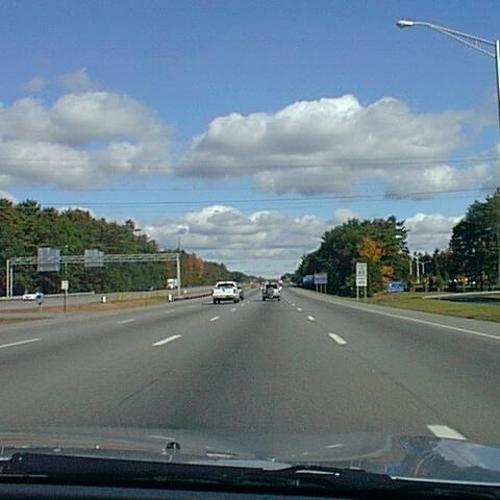

Supplement: Supplementary file 3 [file Presentation_3.zip › Non-targets_1/image_0062.jpg]

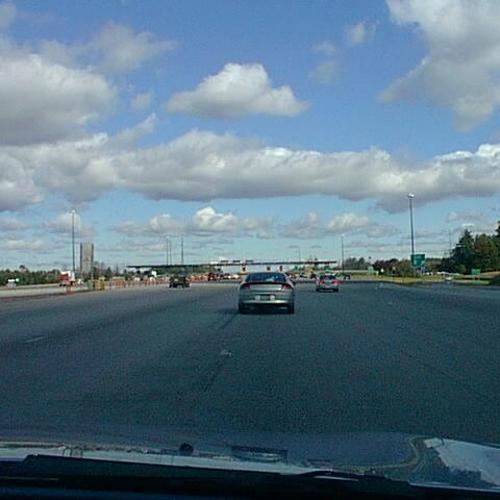

Supplement: Supplementary file 3 [file Presentation_3.zip › Non-targets_1/image_0063.jpg]

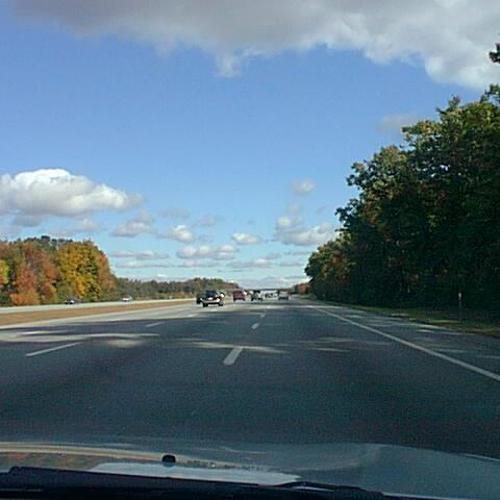

Supplement: Supplementary file 3 [file Presentation_3.zip › Non-targets_1/image_0064.jpg]

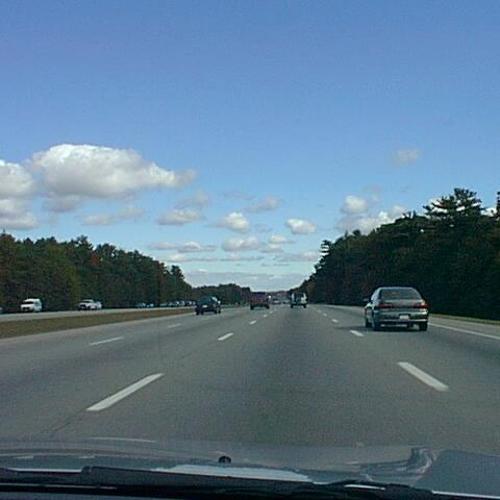

Supplement: Supplementary file 3 [file Presentation_3.zip › Non-targets_1/image_0065.jpg]

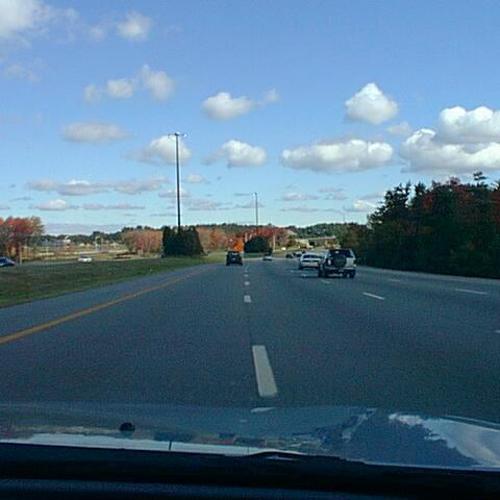

Supplement: Supplementary file 3 [file Presentation_3.zip › Non-targets_1/image_0066.jpg]

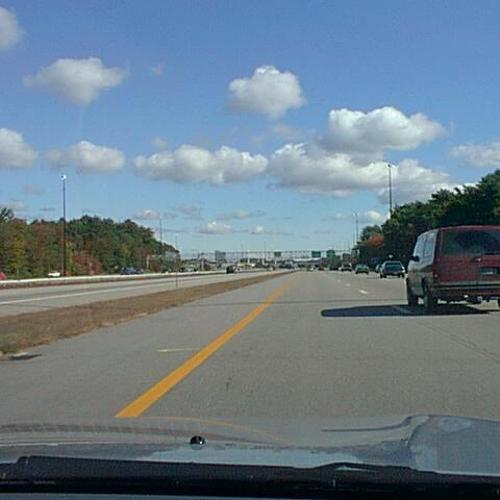

Supplement: Supplementary file 3 [file Presentation_3.zip › Non-targets_1/image_0067.jpg]

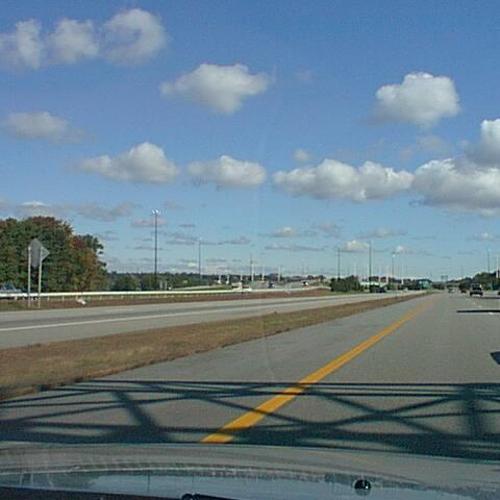

Supplement: Supplementary file 3 [file Presentation_3.zip › Non-targets_1/image_0068.jpg]

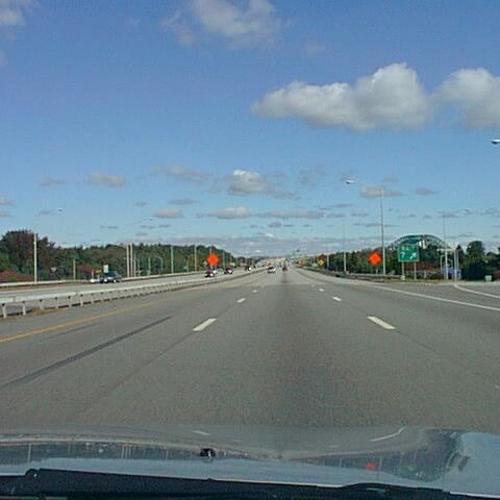

Supplement: Supplementary file 3 [file Presentation_3.zip › Non-targets_1/image_0069.jpg]

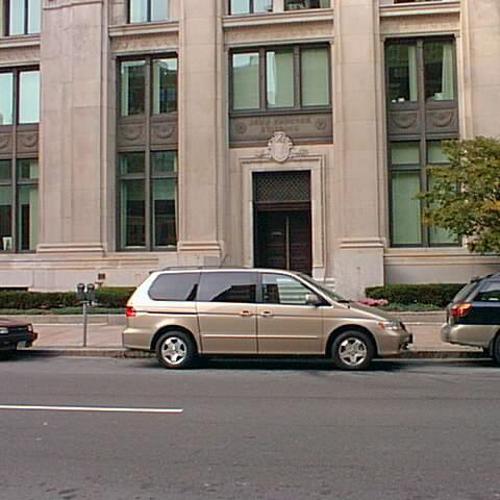

Supplement: Supplementary file 3 [file Presentation_3.zip › Non-targets_1/image_0070.jpg]

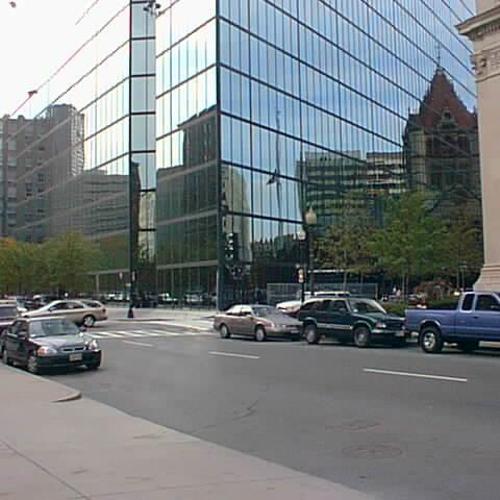

Supplement: Supplementary file 3 [file Presentation_3.zip › Non-targets_1/image_0071.jpg]

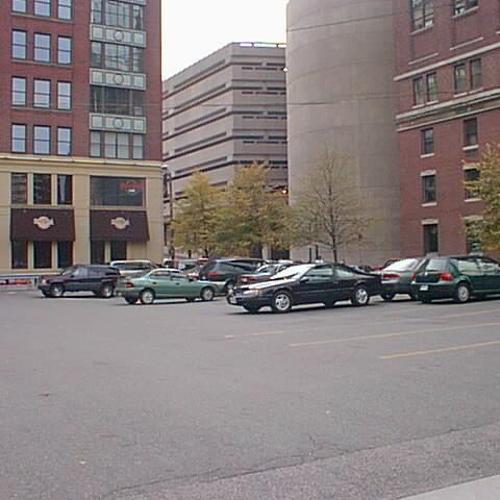

Supplement: Supplementary file 3 [file Presentation_3.zip › Non-targets_1/image_0072.jpg]

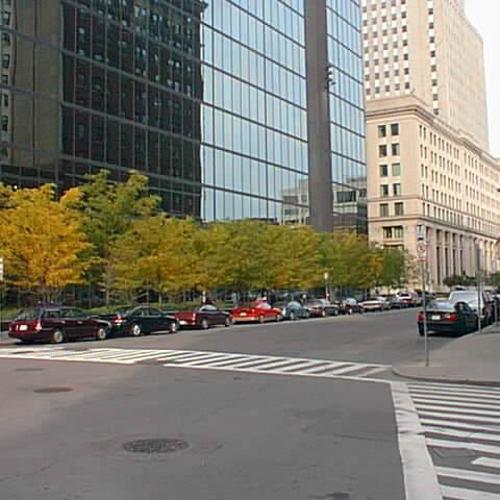

Supplement: Supplementary file 3 [file Presentation_3.zip › Non-targets_1/image_0073.jpg]

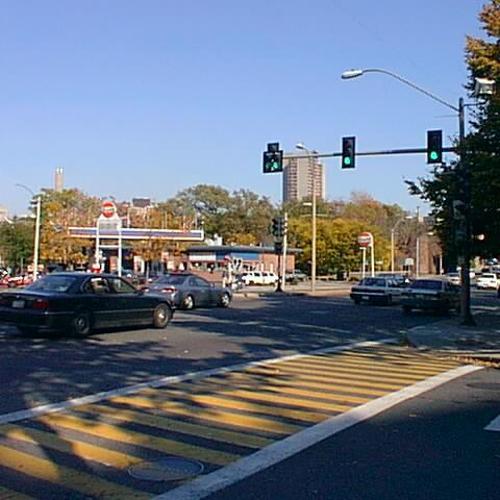

Supplement: Supplementary file 3 [file Presentation_3.zip › Non-targets_1/image_0074.jpg]

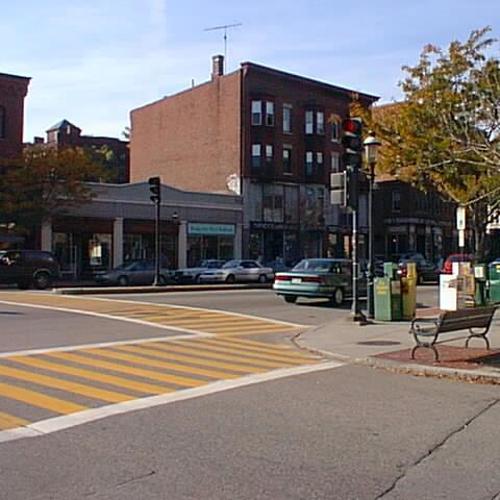

Supplement: Supplementary file 3 [file Presentation_3.zip › Non-targets_1/image_0075.jpg]

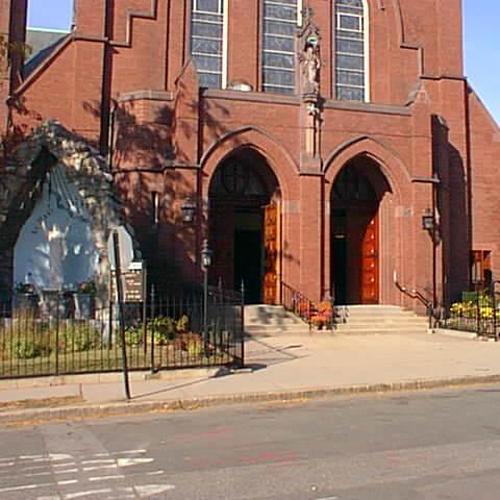

Supplement: Supplementary file 3 [file Presentation_3.zip › Non-targets_1/image_0076.jpg]

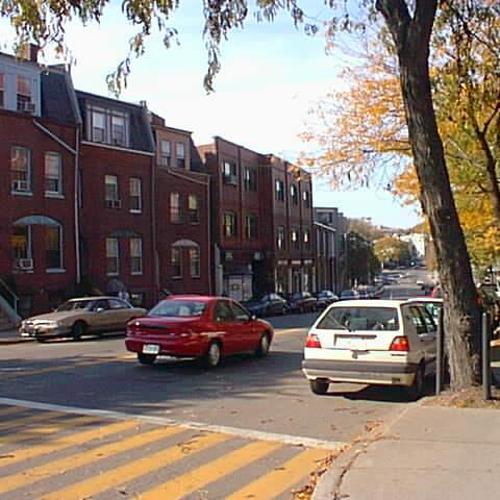

Supplement: Supplementary file 3 [file Presentation_3.zip › Non-targets_1/image_0077.jpg]

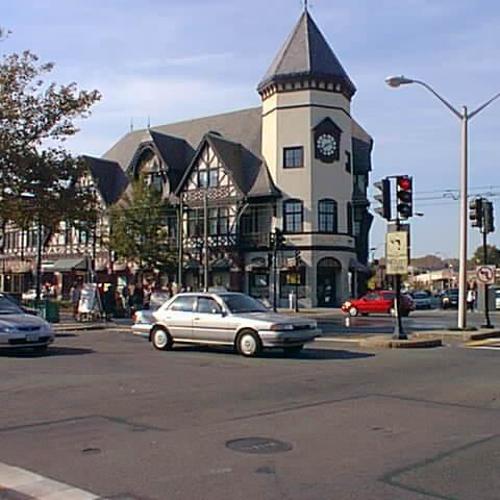

Supplement: Supplementary file 3 [file Presentation_3.zip › Non-targets_1/image_0078.jpg]

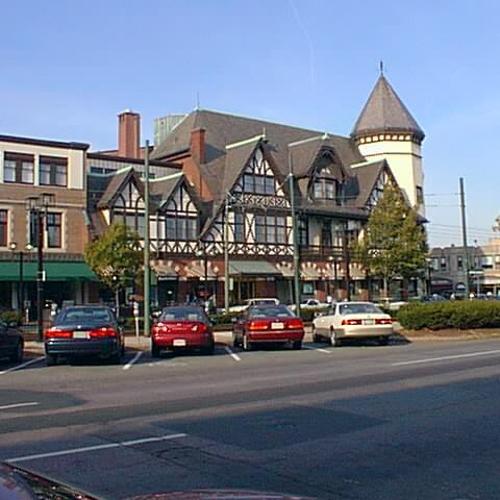

Supplement: Supplementary file 3 [file Presentation_3.zip › Non-targets_1/image_0079.jpg]

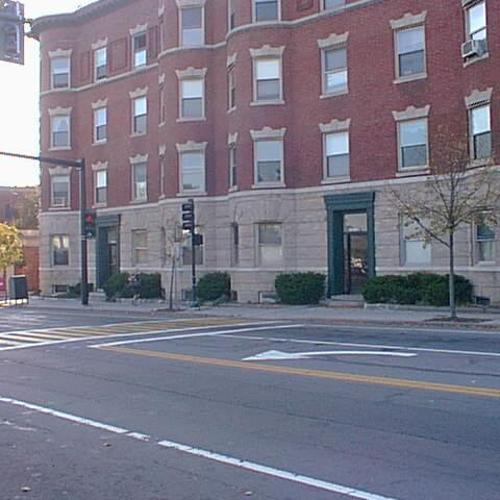

Supplement: Supplementary file 3 [file Presentation_3.zip › Non-targets_1/image_0080.jpg]

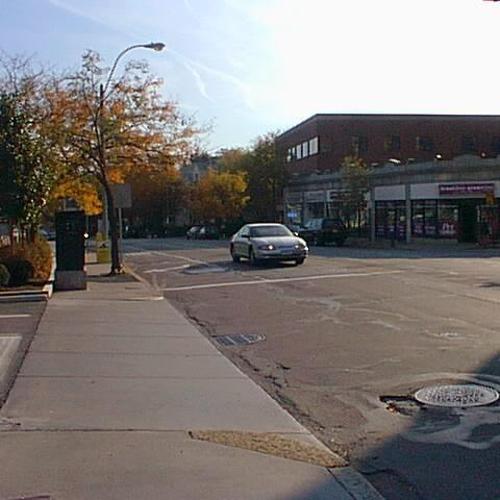

Supplement: Supplementary file 3 [file Presentation_3.zip › Non-targets_1/image_0081.jpg]

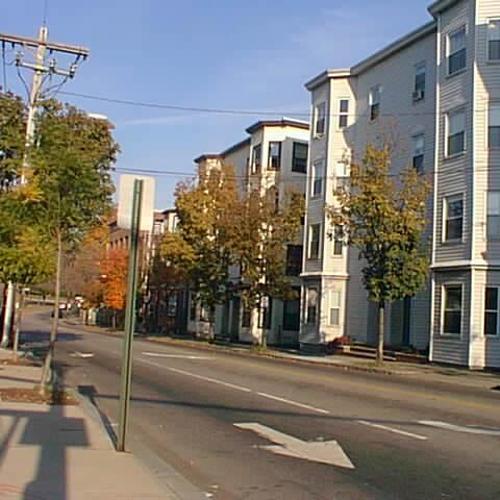

Supplement: Supplementary file 3 [file Presentation_3.zip › Non-targets_1/image_0082.jpg]

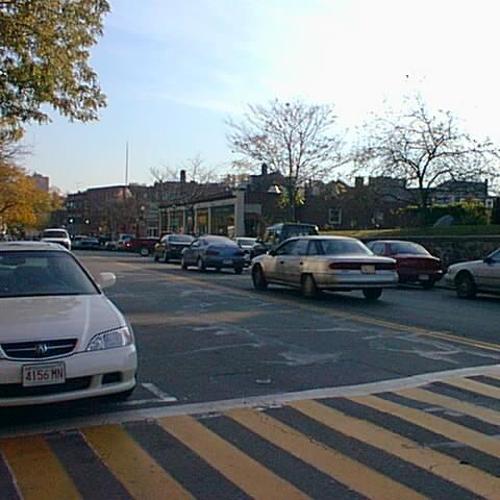

Supplement: Supplementary file 3 [file Presentation_3.zip › Non-targets_1/image_0083.jpg]

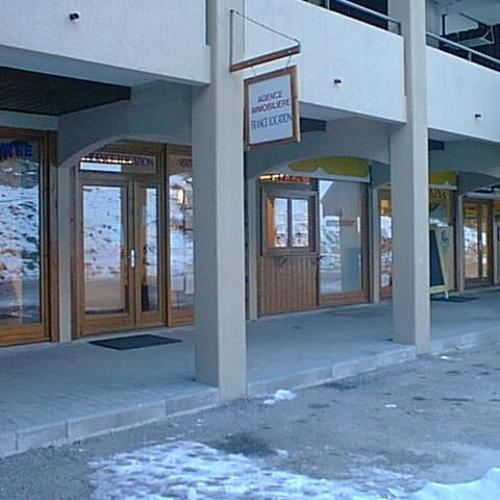

Supplement: Supplementary file 3 [file Presentation_3.zip › Non-targets_1/image_0084.jpg]

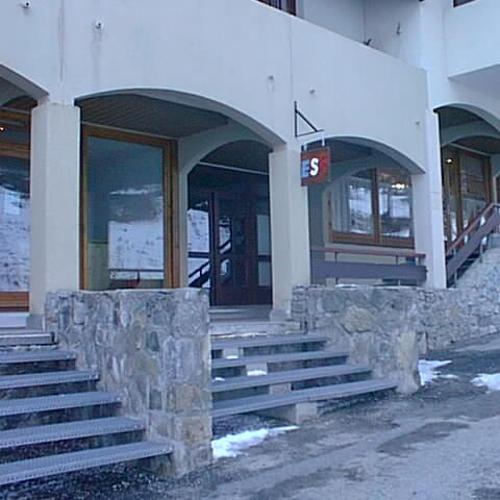

Supplement: Supplementary file 3 [file Presentation_3.zip › Non-targets_1/image_0085.jpg]

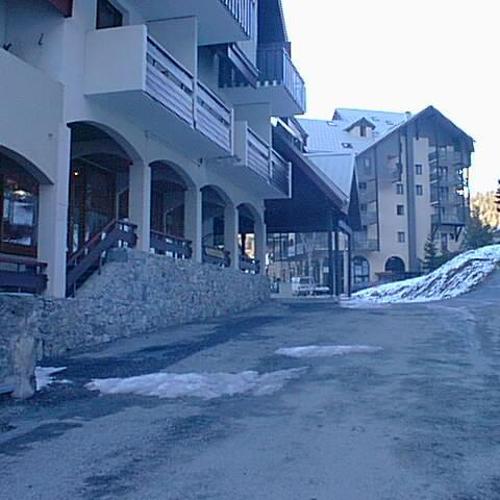

Supplement: Supplementary file 3 [file Presentation_3.zip › Non-targets_1/image_0086.jpg]

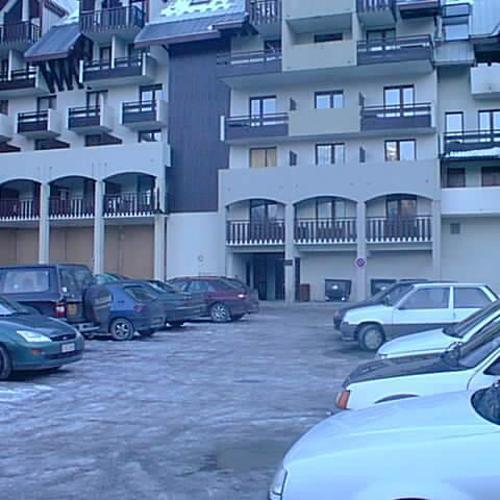

Supplement: Supplementary file 3 [file Presentation_3.zip › Non-targets_1/image_0087.jpg]

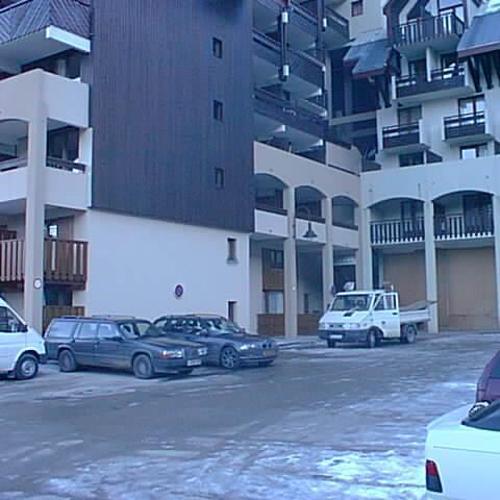

Supplement: Supplementary file 3 [file Presentation_3.zip › Non-targets_1/image_0088.jpg]

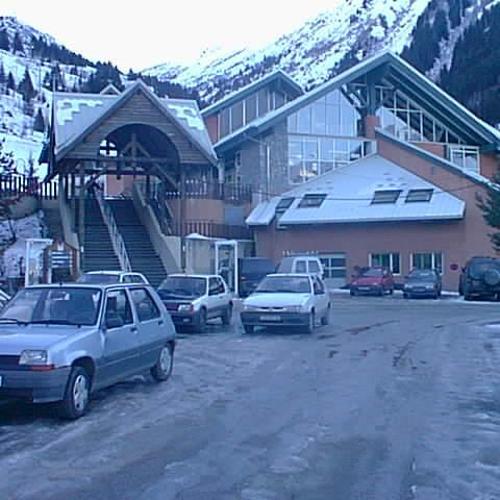

Supplement: Supplementary file 3 [file Presentation_3.zip › Non-targets_1/image_0089.jpg]

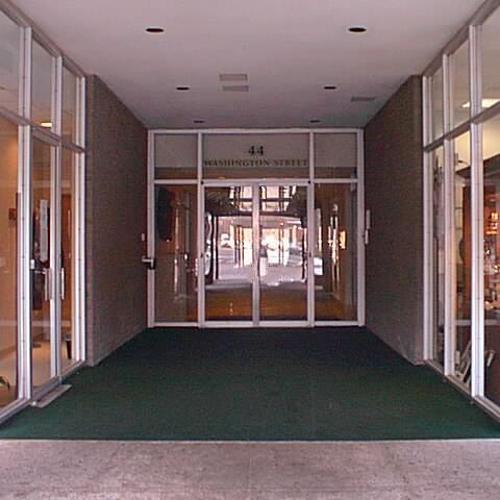

Supplement: Supplementary file 3 [file Presentation_3.zip › Non-targets_1/image_0090.jpg]

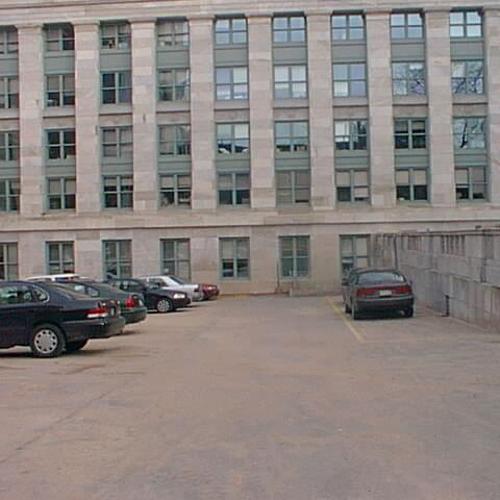

Supplement: Supplementary file 3 [file Presentation_3.zip › Non-targets_1/image_0091.jpg]

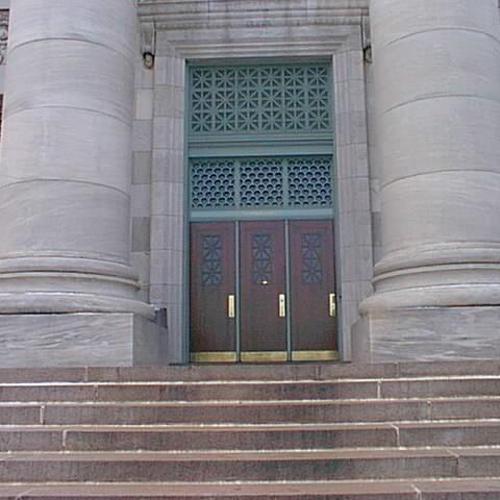

Supplement: Supplementary file 3 [file Presentation_3.zip › Non-targets_1/image_0092.jpg]

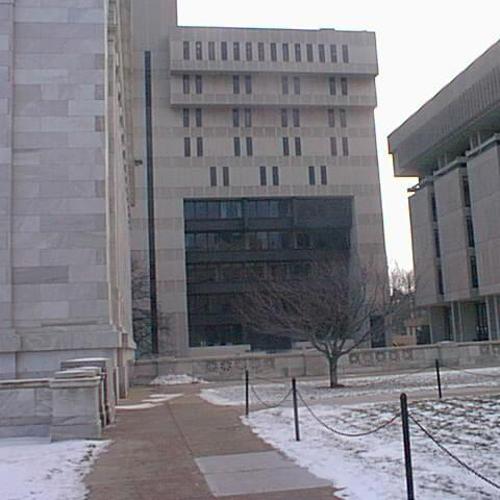

Supplement: Supplementary file 3 [file Presentation_3.zip › Non-targets_1/image_0093.jpg]

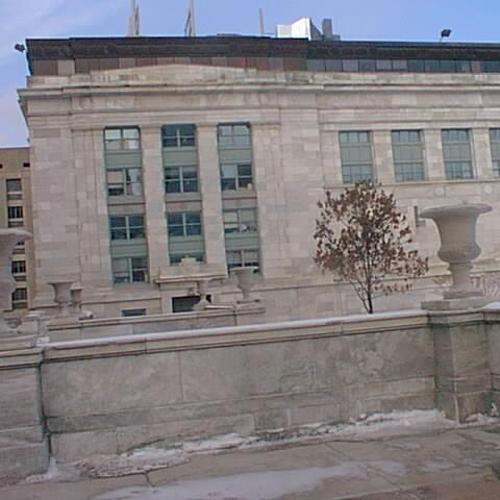

Supplement: Supplementary file 3 [file Presentation_3.zip › Non-targets_1/image_0094.jpg]

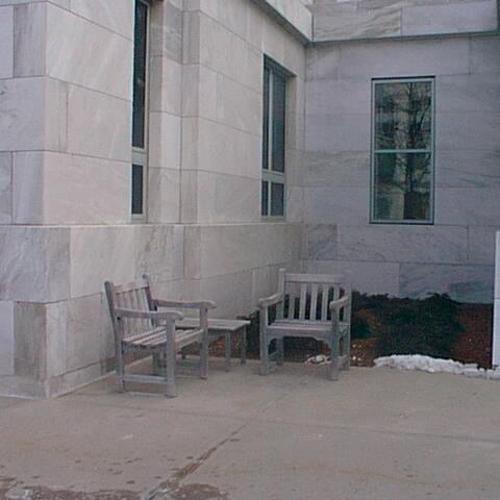

Supplement: Supplementary file 3 [file Presentation_3.zip › Non-targets_1/image_0095.jpg]

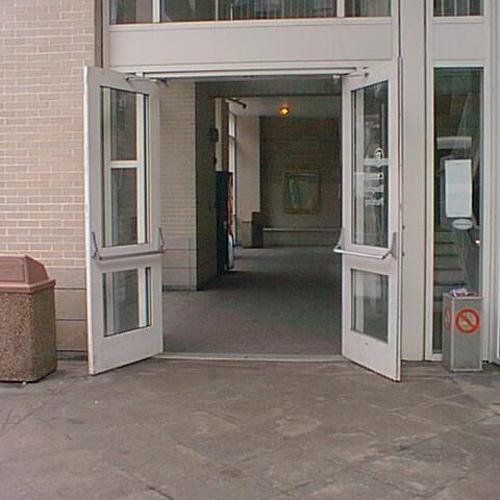

Supplement: Supplementary file 3 [file Presentation_3.zip › Non-targets_1/image_0096.jpg]

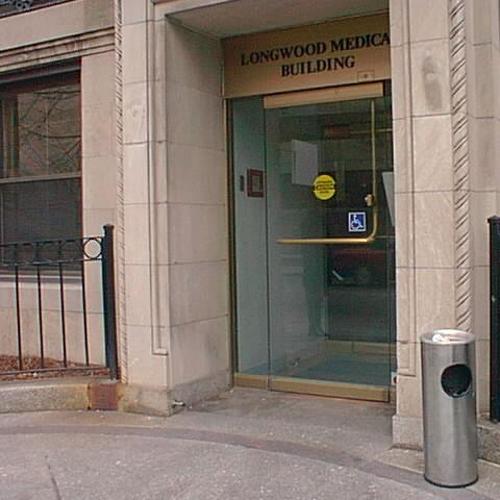

Supplement: Supplementary file 3 [file Presentation_3.zip › Non-targets_1/image_0097.jpg]

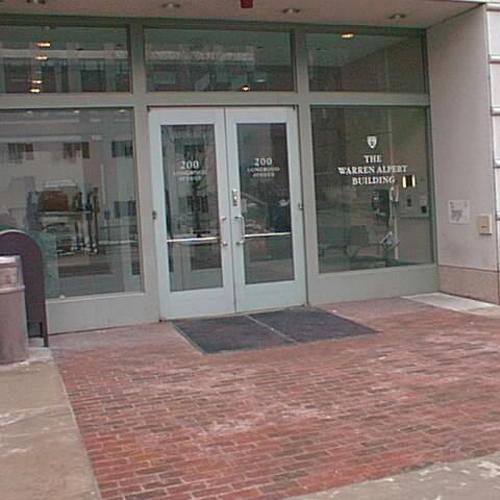

Supplement: Supplementary file 3 [file Presentation_3.zip › Non-targets_1/image_0098.jpg]

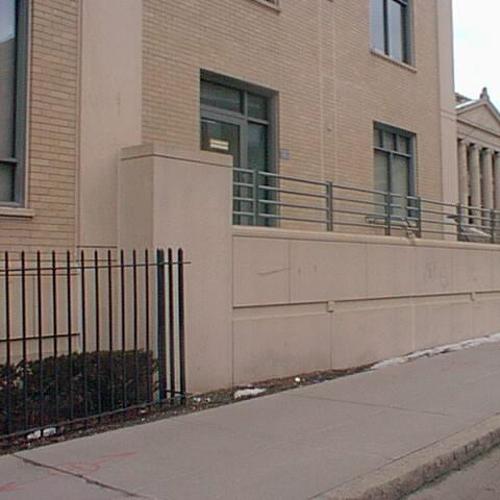

Supplement: Supplementary file 3 [file Presentation_3.zip › Non-targets_1/image_0099.jpg]

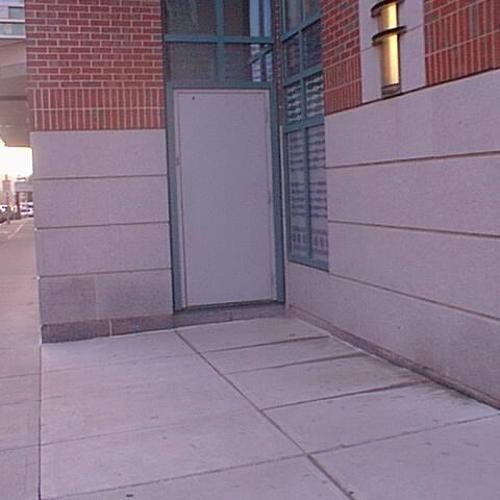

Supplement: Supplementary file 3 [file Presentation_3.zip › Non-targets_1/image_0100.jpg]

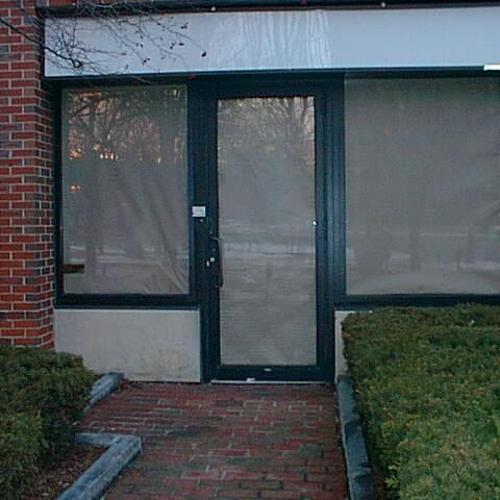

Supplement: Supplementary file 3 [file Presentation_3.zip › Non-targets_1/image_0101.jpg]

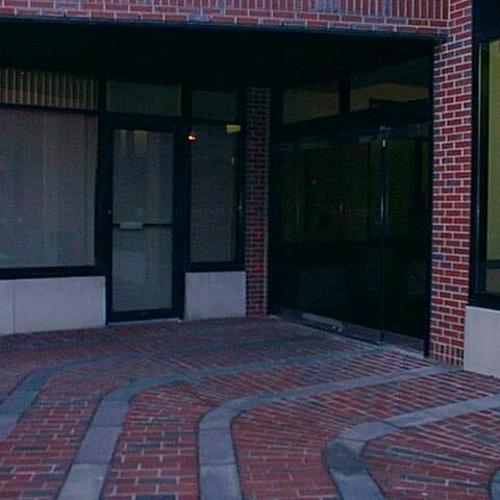

Supplement: Supplementary file 3 [file Presentation_3.zip › Non-targets_1/image_0102.jpg]

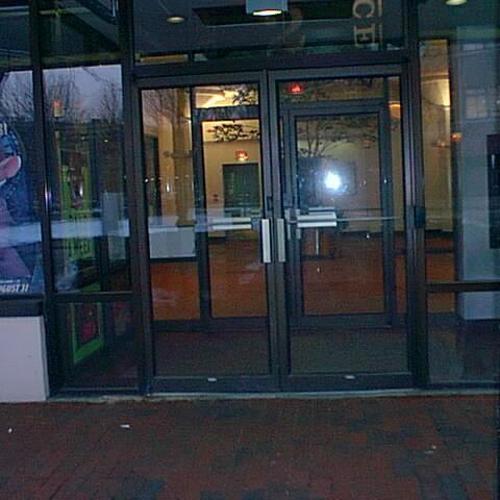

Supplement: Supplementary file 3 [file Presentation_3.zip › Non-targets_1/image_0103.jpg]

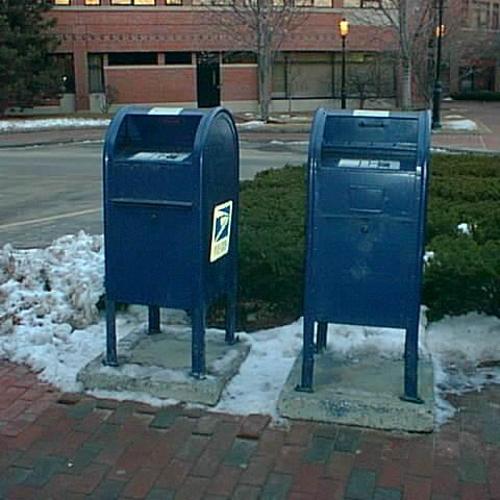

Supplement: Supplementary file 3 [file Presentation_3.zip › Non-targets_1/image_0104.jpg]

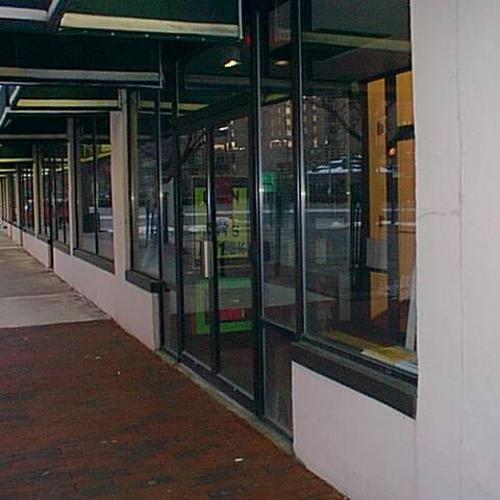

Supplement: Supplementary file 3 [file Presentation_3.zip › Non-targets_1/image_0105.jpg]

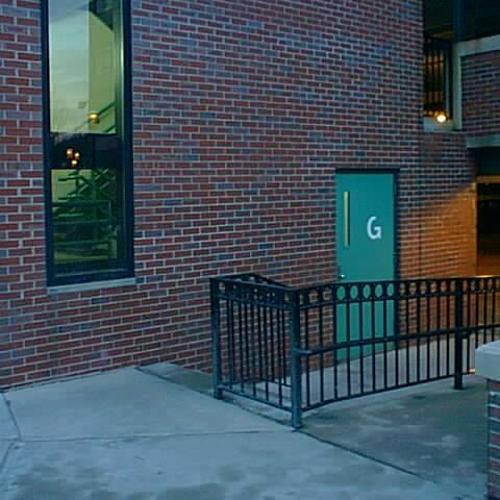

Supplement: Supplementary file 3 [file Presentation_3.zip › Non-targets_1/image_0106.jpg]

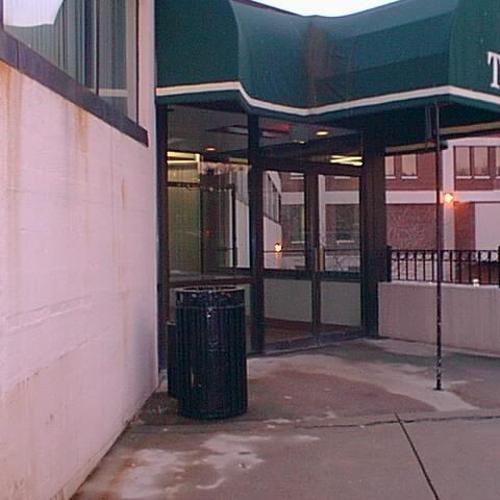

Supplement: Supplementary file 3 [file Presentation_3.zip › Non-targets_1/image_0107.jpg]

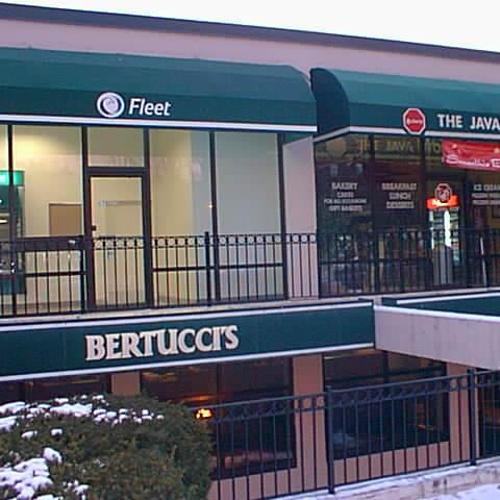

Supplement: Supplementary file 3 [file Presentation_3.zip › Non-targets_1/image_0108.jpg]

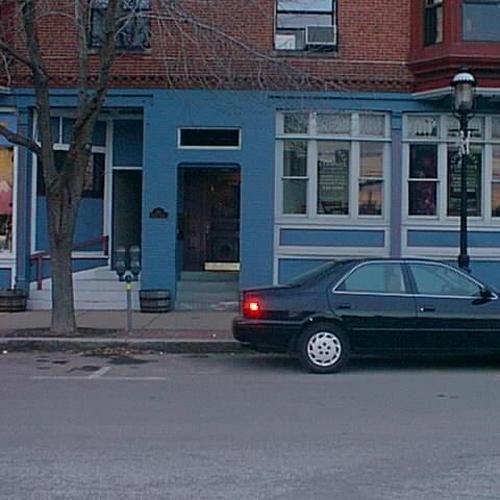

Supplement: Supplementary file 3 [file Presentation_3.zip › Non-targets_1/image_0109.jpg]

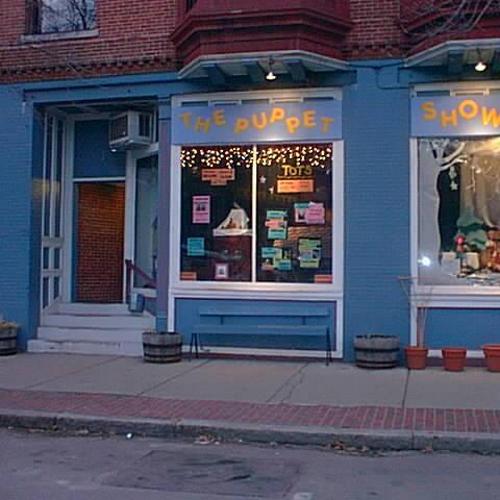

Supplement: Supplementary file 3 [file Presentation_3.zip › Non-targets_1/image_0110.jpg]

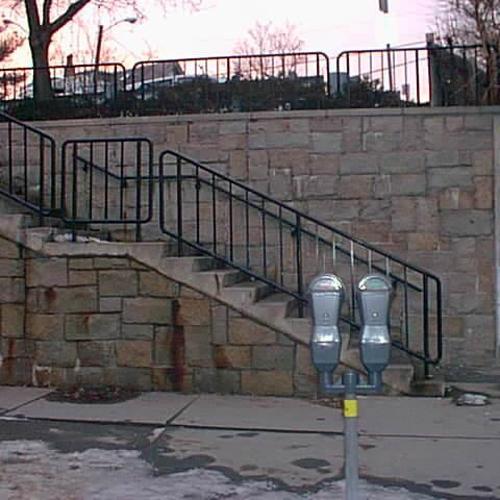

Supplement: Supplementary file 3 [file Presentation_3.zip › Non-targets_1/image_0111.jpg]

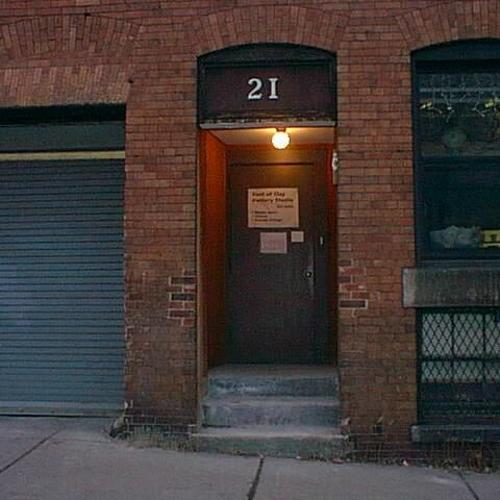

Supplement: Supplementary file 3 [file Presentation_3.zip › Non-targets_1/image_0112.jpg]

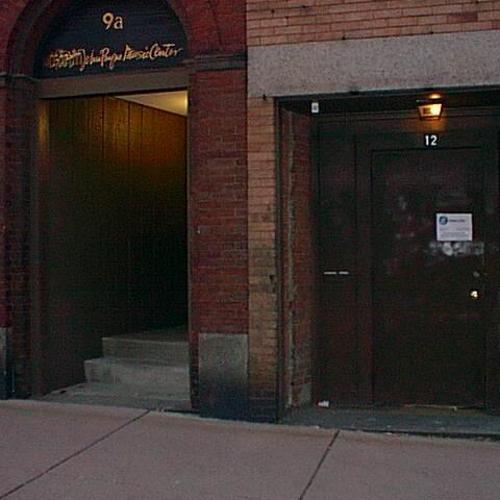

Supplement: Supplementary file 3 [file Presentation_3.zip › Non-targets_1/image_0113.jpg]

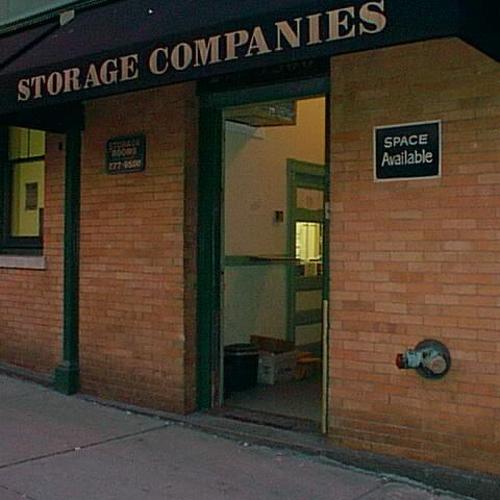

Supplement: Supplementary file 3 [file Presentation_3.zip › Non-targets_1/image_0114.jpg]

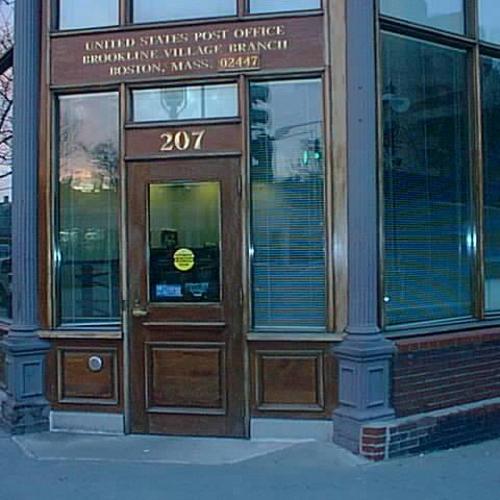

Supplement: Supplementary file 3 [file Presentation_3.zip › Non-targets_1/image_0115.jpg]

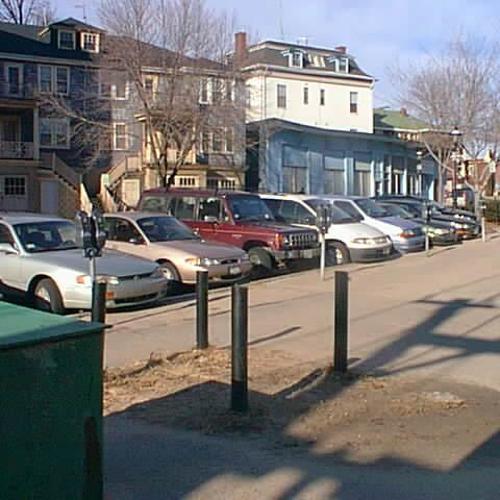

Supplement: Supplementary file 3 [file Presentation_3.zip › Non-targets_1/image_0116.jpg]

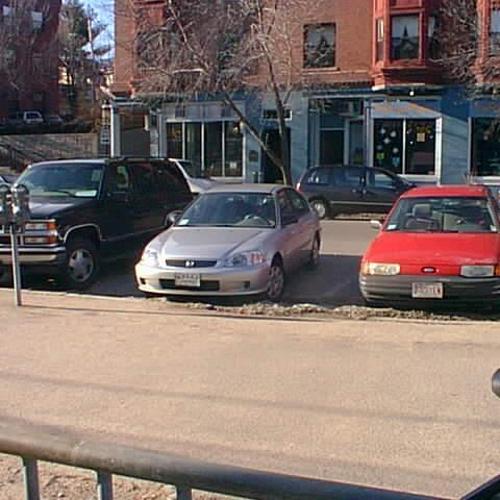

Supplement: Supplementary file 3 [file Presentation_3.zip › Non-targets_1/image_0117.jpg]

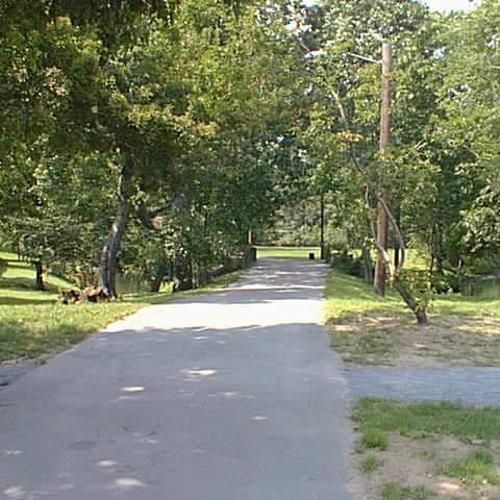

Supplement: Supplementary file 3 [file Presentation_3.zip › Non-targets_1/image_0118.jpg]

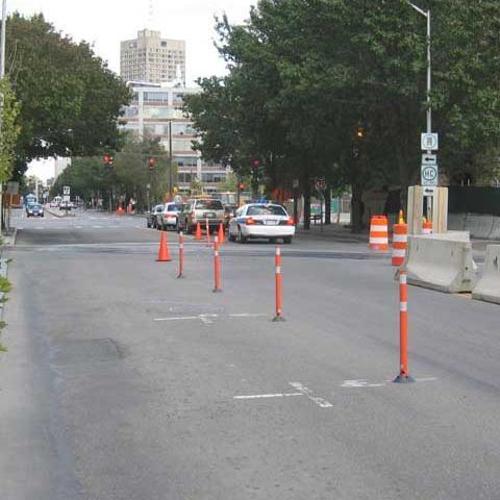

Supplement: Supplementary file 3 [file Presentation_3.zip › Non-targets_1/image_0119.jpg]

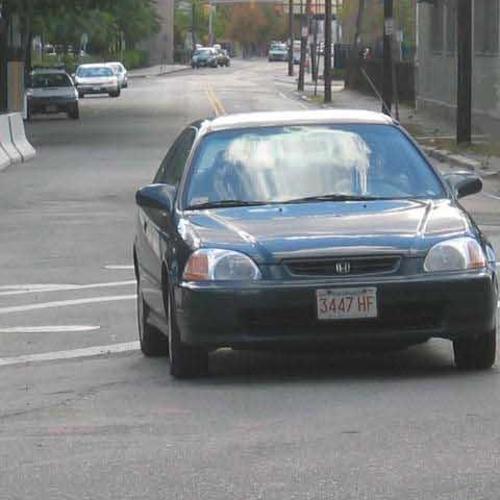

Supplement: Supplementary file 3 [file Presentation_3.zip › Non-targets_1/image_0120.jpg]

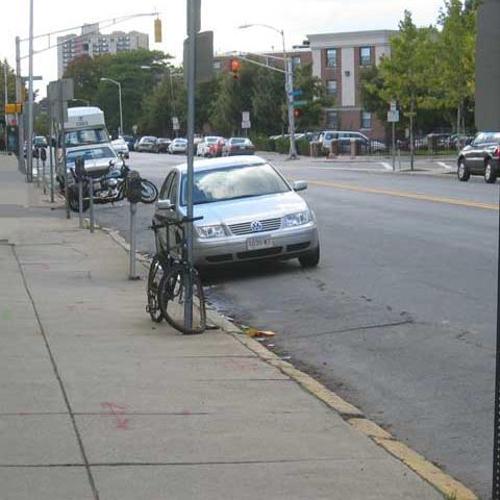

Supplement: Supplementary file 3 [file Presentation_3.zip › Non-targets_1/image_0121.jpg]

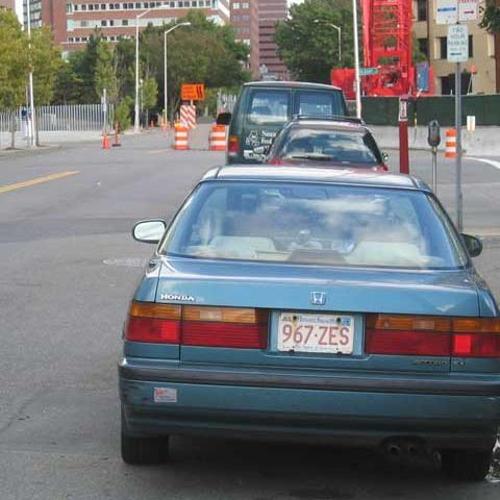

Supplement: Supplementary file 3 [file Presentation_3.zip › Non-targets_1/image_0122.jpg]

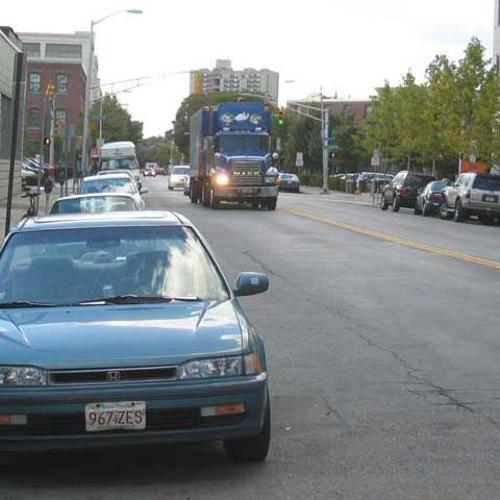

Supplement: Supplementary file 3 [file Presentation_3.zip › Non-targets_1/image_0123.jpg]

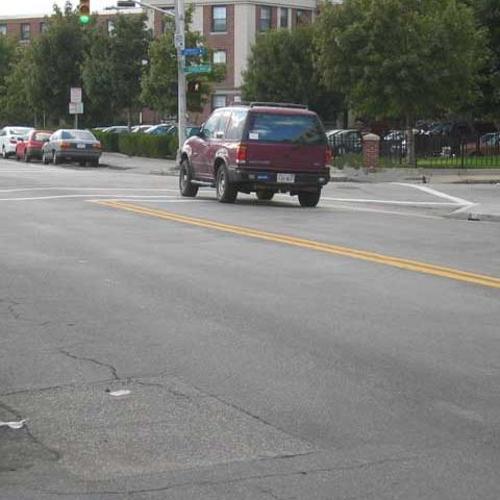

Supplement: Supplementary file 3 [file Presentation_3.zip › Non-targets_1/image_0124.jpg]

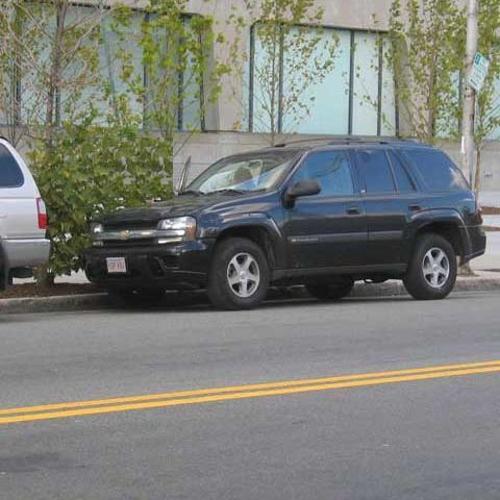

Supplement: Supplementary file 3 [file Presentation_3.zip › Non-targets_1/image_0125.jpg]

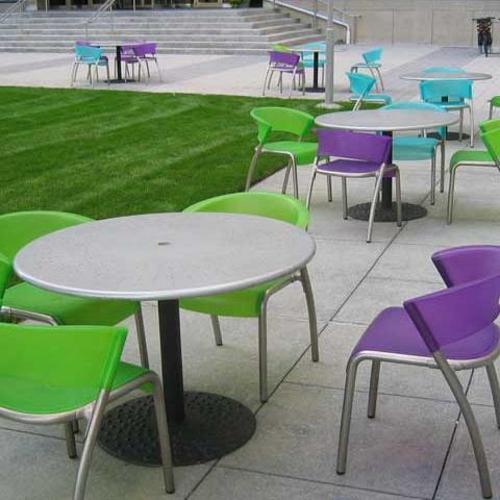

Supplement: Supplementary file 3 [file Presentation_3.zip › Non-targets_1/image_0126.jpg]

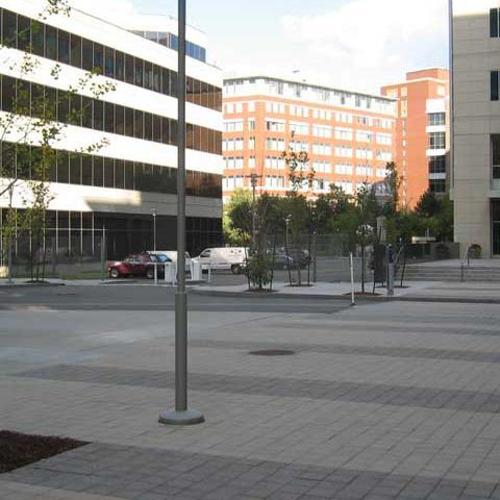

Supplement: Supplementary file 3 [file Presentation_3.zip › Non-targets_1/image_0127.jpg]

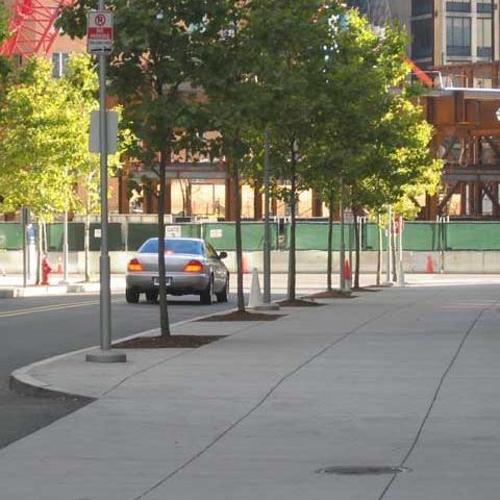

Supplement: Supplementary file 3 [file Presentation_3.zip › Non-targets_1/image_0128.jpg]

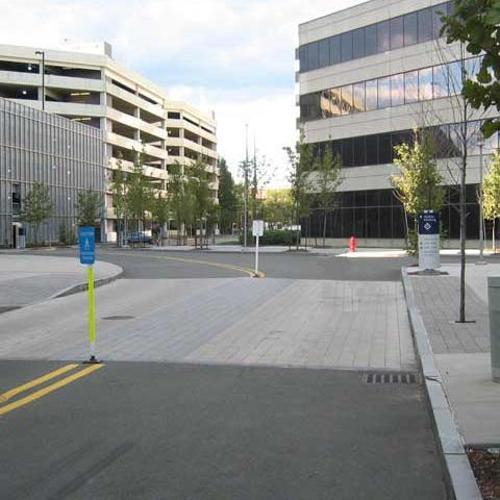

Supplement: Supplementary file 3 [file Presentation_3.zip › Non-targets_1/image_0129.jpg]

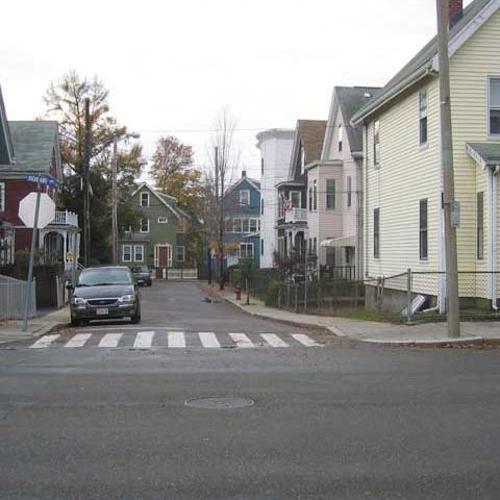

Supplement: Supplementary file 3 [file Presentation_3.zip › Non-targets_1/image_0130.jpg]

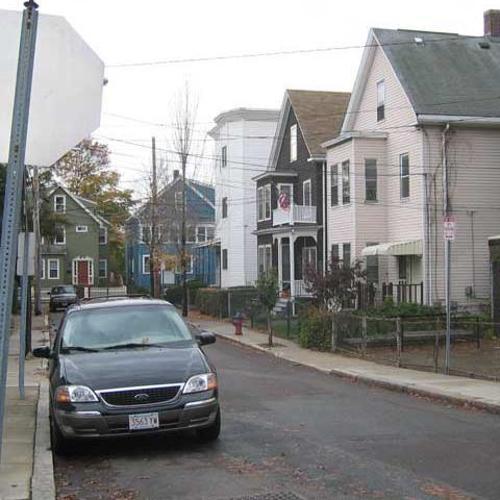

Supplement: Supplementary file 3 [file Presentation_3.zip › Non-targets_1/image_0131.jpg]

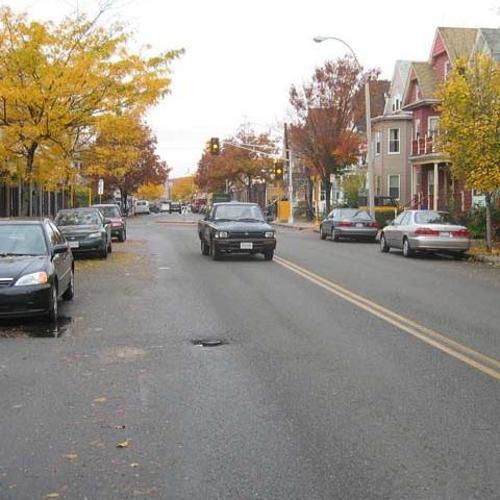

Supplement: Supplementary file 3 [file Presentation_3.zip › Non-targets_1/image_0132.jpg]

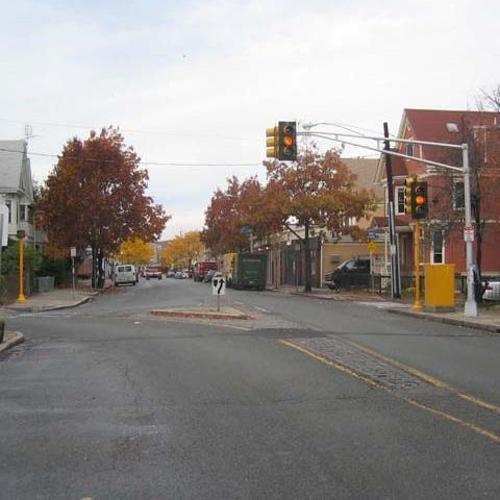

Supplement: Supplementary file 3 [file Presentation_3.zip › Non-targets_1/image_0133.jpg]

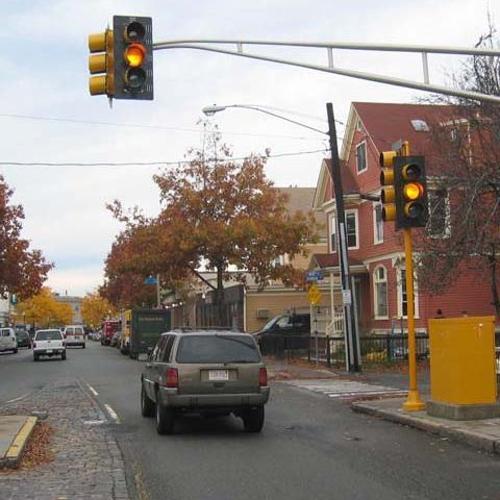

Supplement: Supplementary file 3 [file Presentation_3.zip › Non-targets_1/image_0134.jpg]

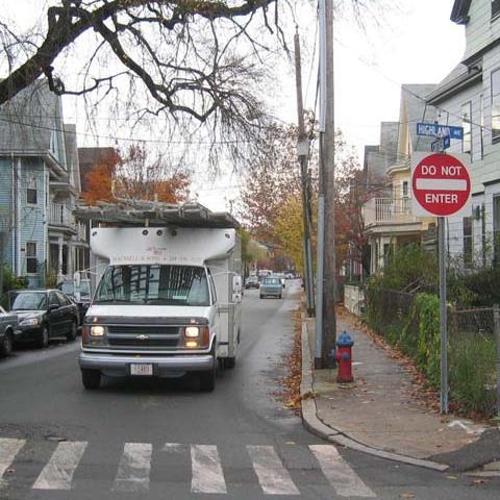

Supplement: Supplementary file 3 [file Presentation_3.zip › Non-targets_1/image_0135.jpg]

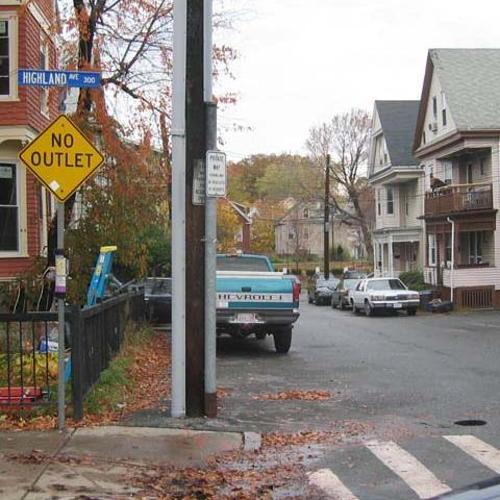

Supplement: Supplementary file 3 [file Presentation_3.zip › Non-targets_1/image_0136.jpg]

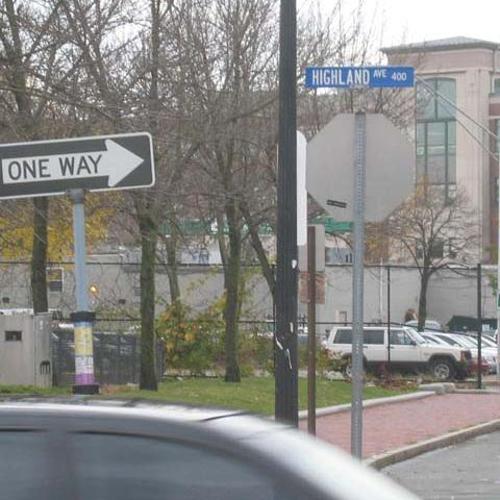

Supplement: Supplementary file 3 [file Presentation_3.zip › Non-targets_1/image_0137.jpg]

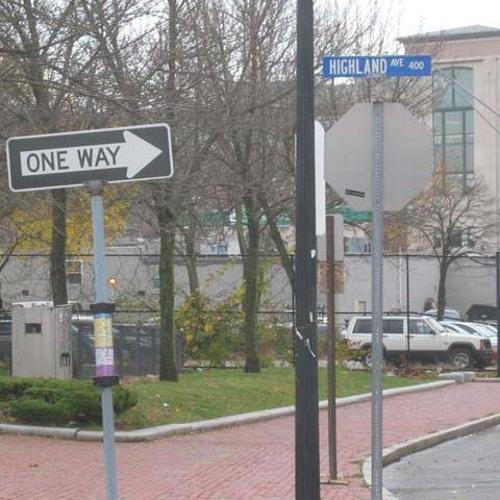

Supplement: Supplementary file 3 [file Presentation_3.zip › Non-targets_1/image_0138.jpg]

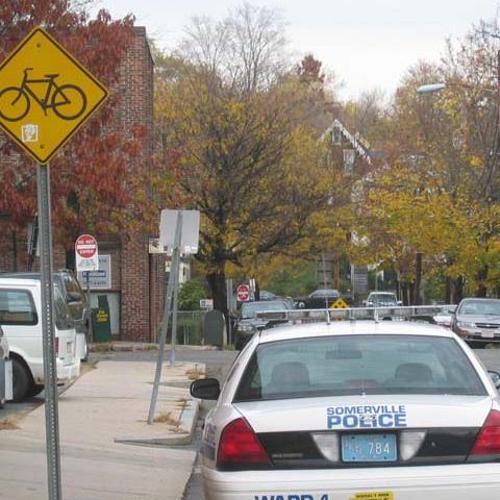

Supplement: Supplementary file 3 [file Presentation_3.zip › Non-targets_1/image_0139.jpg]

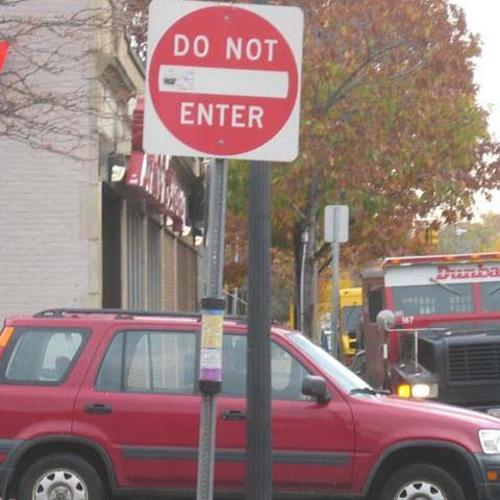

Supplement: Supplementary file 3 [file Presentation_3.zip › Non-targets_1/image_0140.jpg]

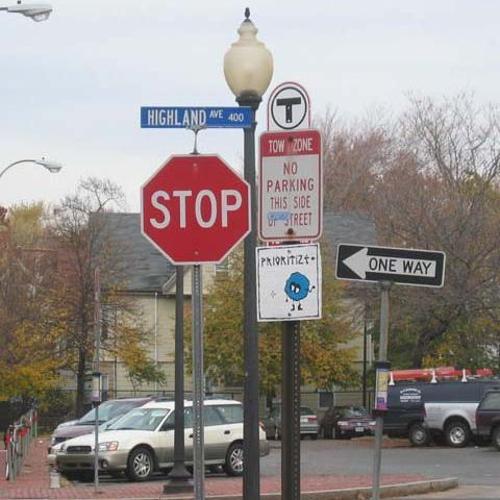

Supplement: Supplementary file 3 [file Presentation_3.zip › Non-targets_1/image_0141.jpg]

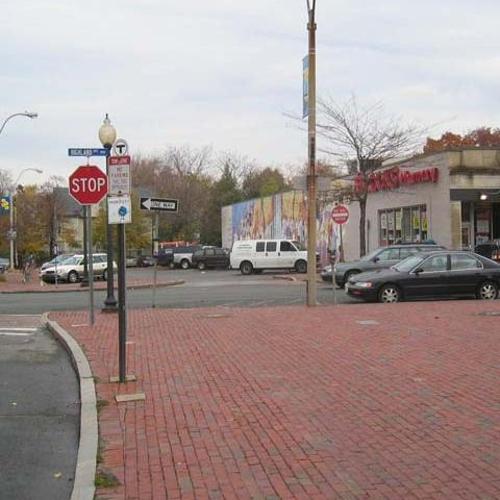

Supplement: Supplementary file 3 [file Presentation_3.zip › Non-targets_1/image_0142.jpg]

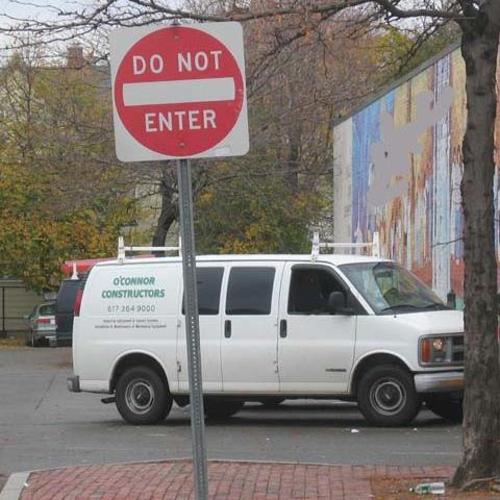

Supplement: Supplementary file 3 [file Presentation_3.zip › Non-targets_1/image_0143.jpg]

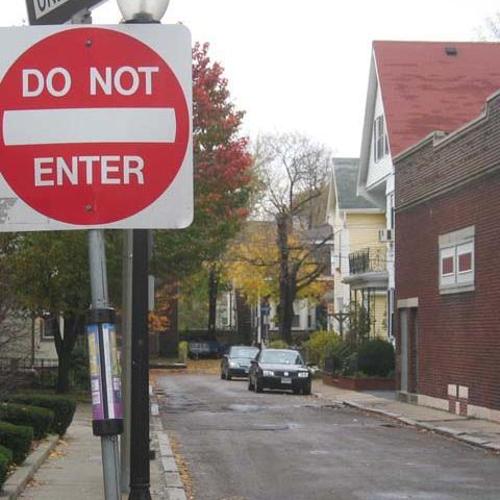

Supplement: Supplementary file 3 [file Presentation_3.zip › Non-targets_1/image_0144.jpg]

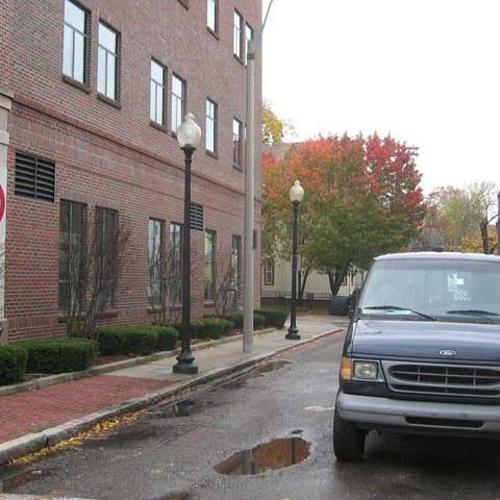

Supplement: Supplementary file 3 [file Presentation_3.zip › Non-targets_1/image_0145.jpg]
