# Supplementary figures and images for: A Cross-Session Dataset for Collaborative Brain-Computer Interfaces Based on Rapid Serial Visual Presentation (part 5 of 5)
Source: Front Neurosci. 2020 Oct 22;14:579469. doi: 10.3389/fnins.2020.579469 (PMC7642747; doi:10.3389/fnins.2020.579469)

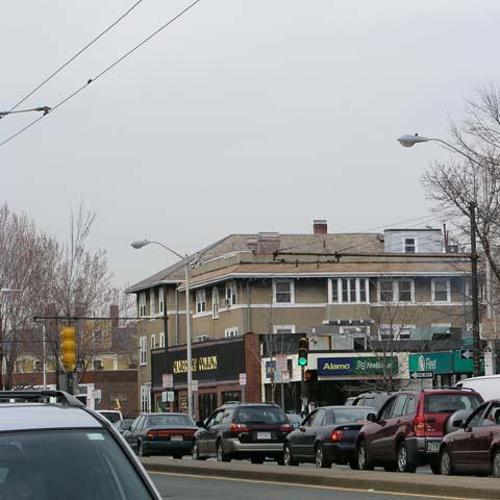

Supplement: Supplementary file 4 [file Presentation_4.zip › Non-targets_2/image_0847.jpg]

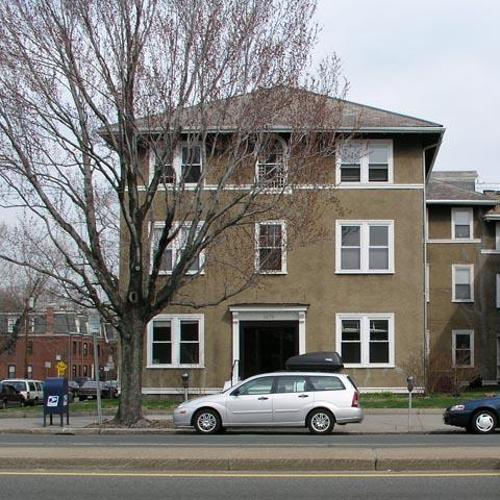

Supplement: Supplementary file 4 [file Presentation_4.zip › Non-targets_2/image_0848.jpg]

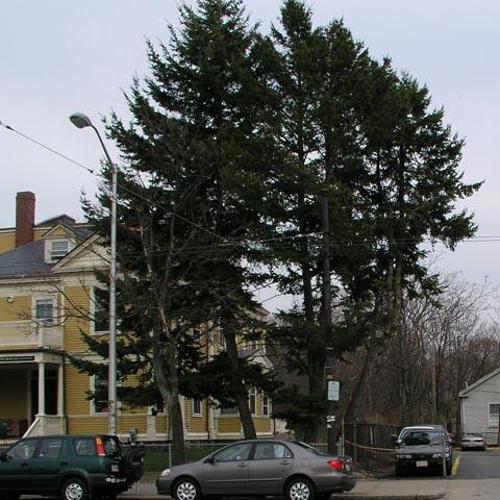

Supplement: Supplementary file 4 [file Presentation_4.zip › Non-targets_2/image_0849.jpg]

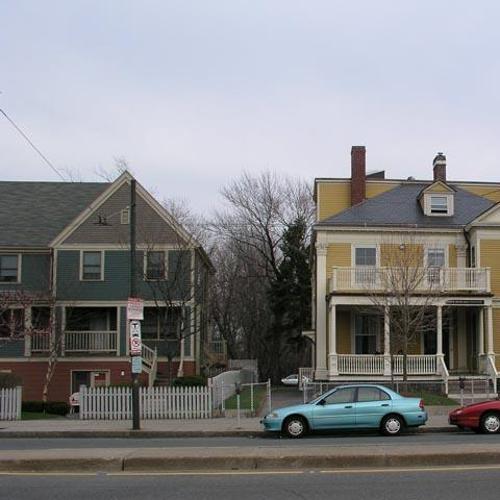

Supplement: Supplementary file 4 [file Presentation_4.zip › Non-targets_2/image_0850.jpg]

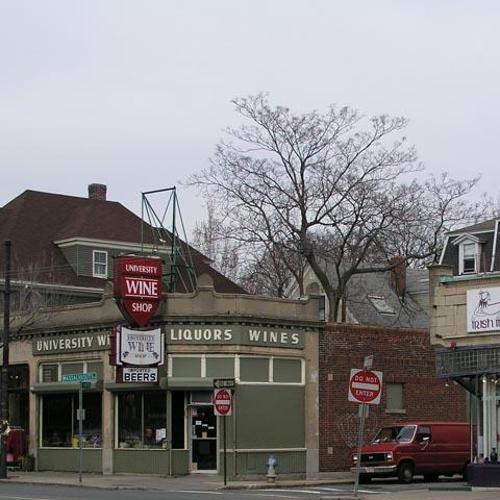

Supplement: Supplementary file 4 [file Presentation_4.zip › Non-targets_2/image_0851.jpg]

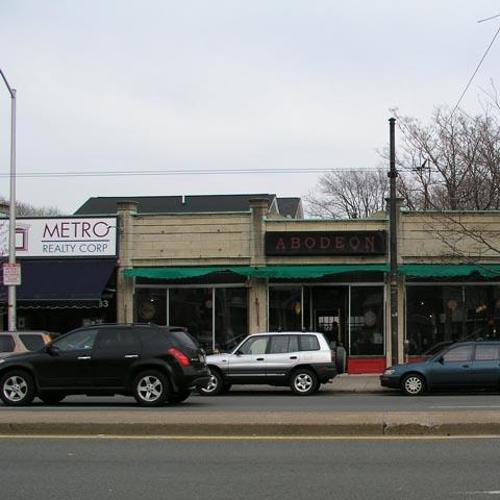

Supplement: Supplementary file 4 [file Presentation_4.zip › Non-targets_2/image_0852.jpg]

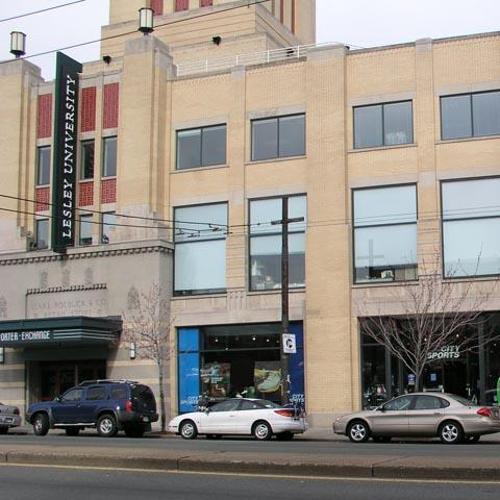

Supplement: Supplementary file 4 [file Presentation_4.zip › Non-targets_2/image_0853.jpg]

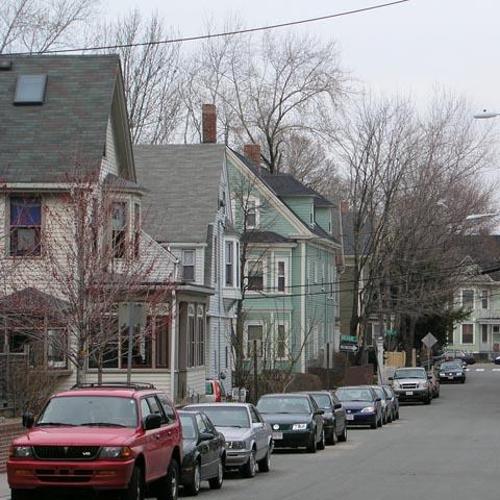

Supplement: Supplementary file 4 [file Presentation_4.zip › Non-targets_2/image_0854.jpg]

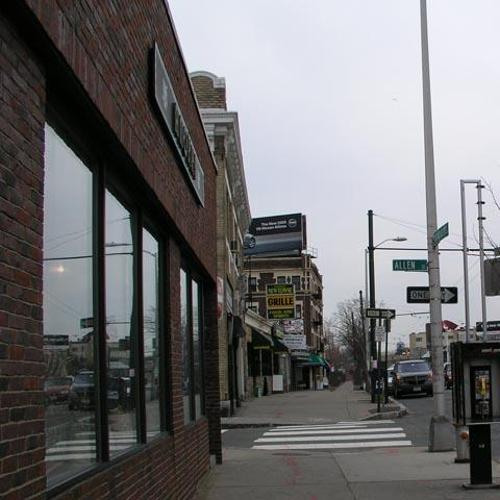

Supplement: Supplementary file 4 [file Presentation_4.zip › Non-targets_2/image_0855.jpg]

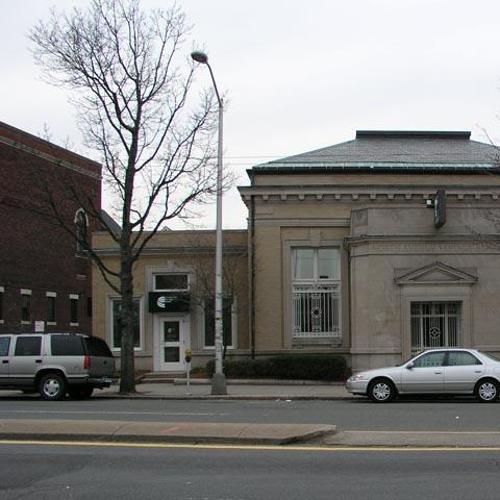

Supplement: Supplementary file 4 [file Presentation_4.zip › Non-targets_2/image_0856.jpg]

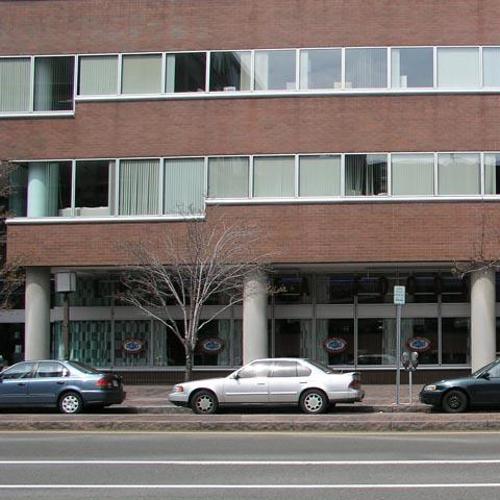

Supplement: Supplementary file 4 [file Presentation_4.zip › Non-targets_2/image_0857.jpg]

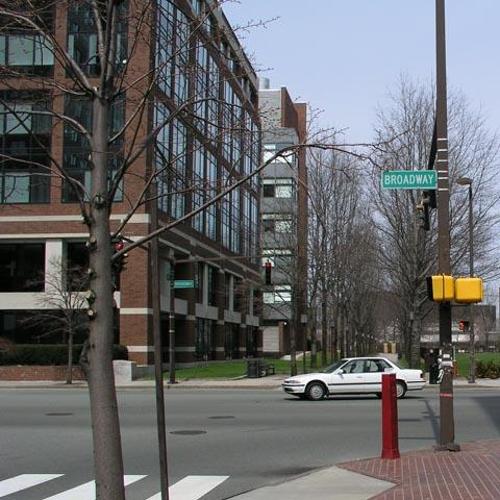

Supplement: Supplementary file 4 [file Presentation_4.zip › Non-targets_2/image_0858.jpg]

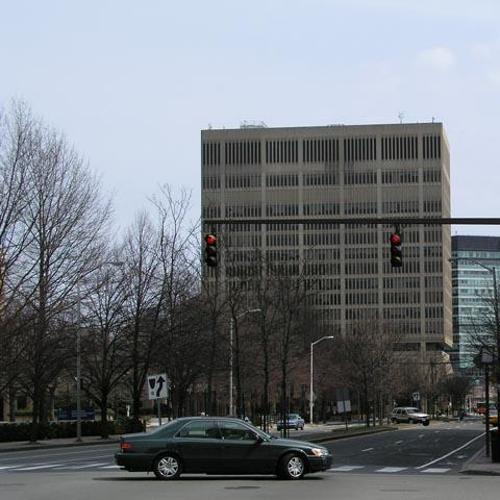

Supplement: Supplementary file 4 [file Presentation_4.zip › Non-targets_2/image_0859.jpg]

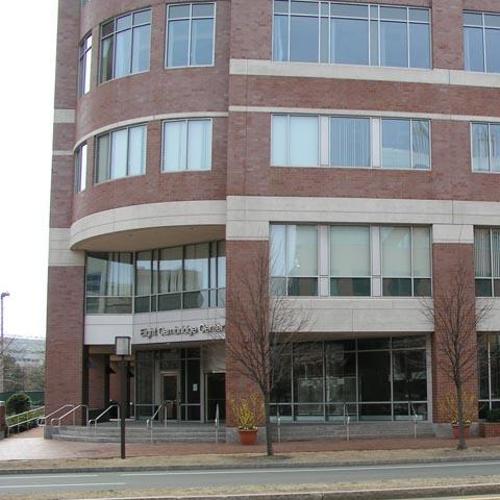

Supplement: Supplementary file 4 [file Presentation_4.zip › Non-targets_2/image_0860.jpg]

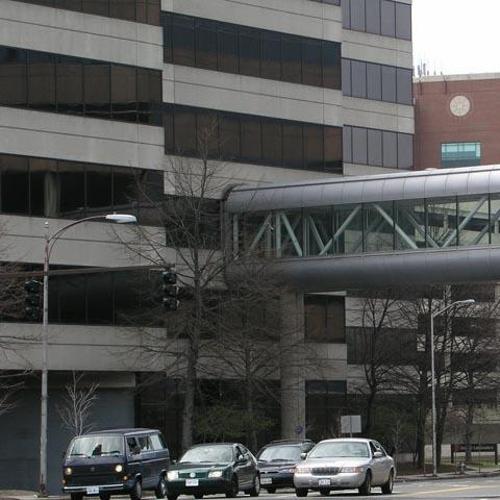

Supplement: Supplementary file 4 [file Presentation_4.zip › Non-targets_2/image_0861.jpg]

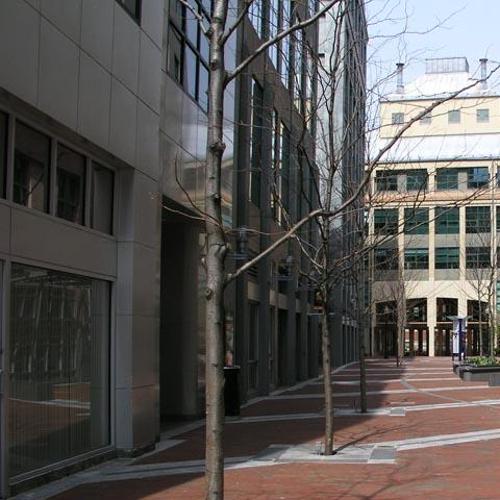

Supplement: Supplementary file 4 [file Presentation_4.zip › Non-targets_2/image_0862.jpg]

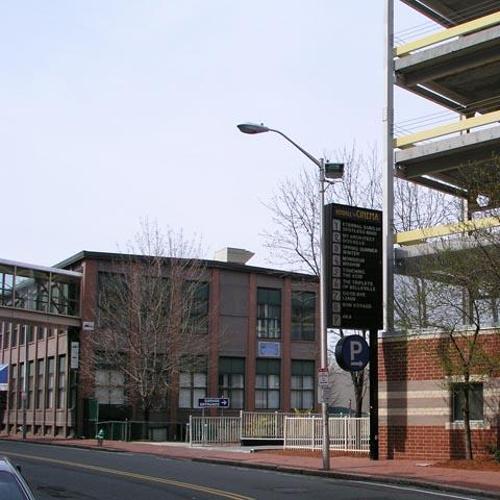

Supplement: Supplementary file 4 [file Presentation_4.zip › Non-targets_2/image_0863.jpg]

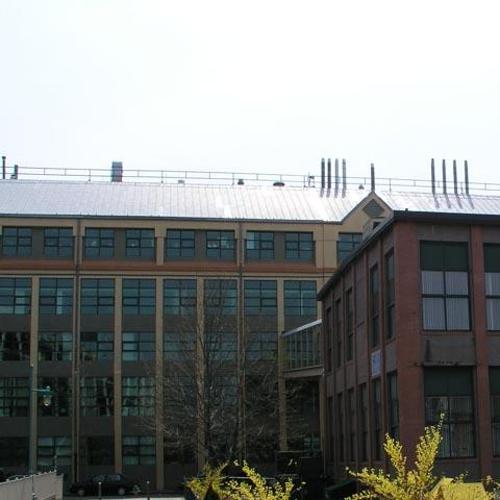

Supplement: Supplementary file 4 [file Presentation_4.zip › Non-targets_2/image_0864.jpg]

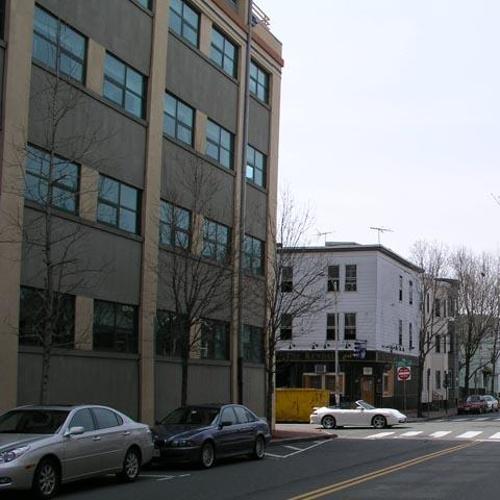

Supplement: Supplementary file 4 [file Presentation_4.zip › Non-targets_2/image_0865.jpg]

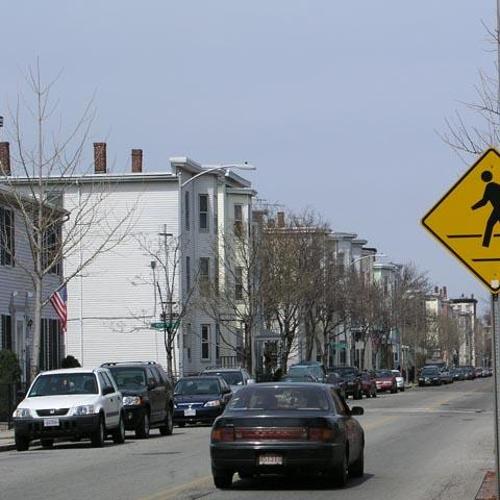

Supplement: Supplementary file 4 [file Presentation_4.zip › Non-targets_2/image_0866.jpg]

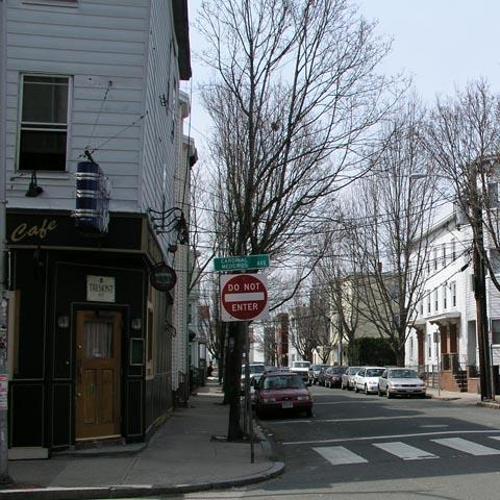

Supplement: Supplementary file 4 [file Presentation_4.zip › Non-targets_2/image_0867.jpg]

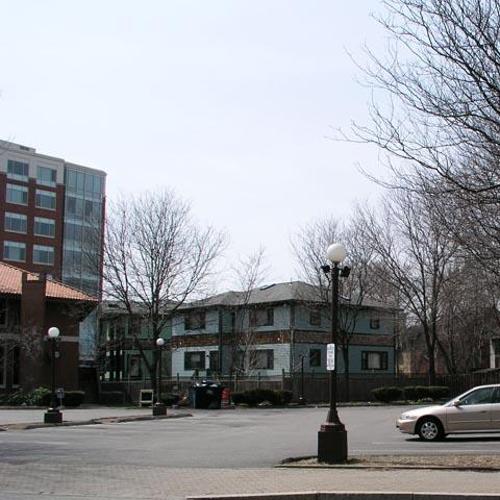

Supplement: Supplementary file 4 [file Presentation_4.zip › Non-targets_2/image_0868.jpg]

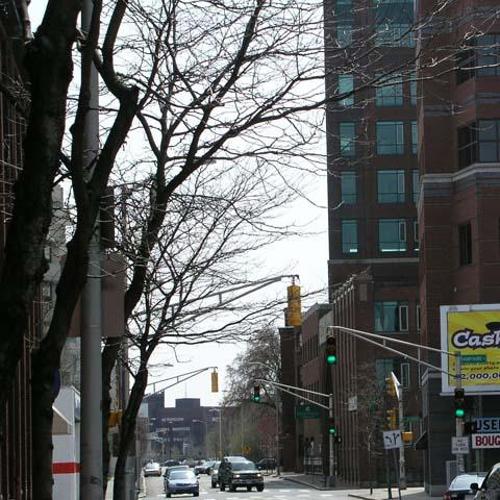

Supplement: Supplementary file 4 [file Presentation_4.zip › Non-targets_2/image_0869.jpg]

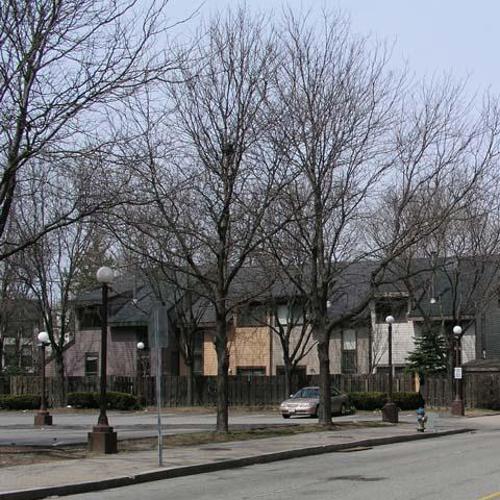

Supplement: Supplementary file 4 [file Presentation_4.zip › Non-targets_2/image_0870.jpg]

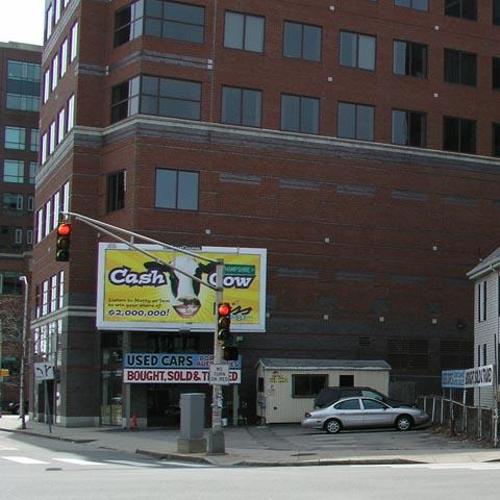

Supplement: Supplementary file 4 [file Presentation_4.zip › Non-targets_2/image_0871.jpg]

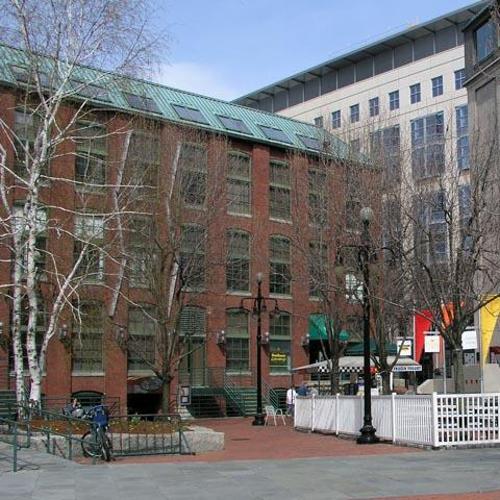

Supplement: Supplementary file 4 [file Presentation_4.zip › Non-targets_2/image_0872.jpg]

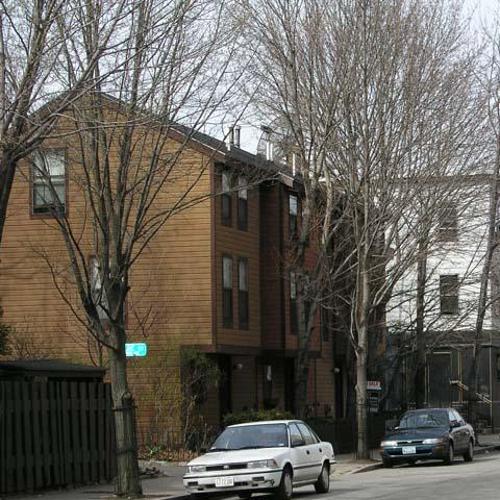

Supplement: Supplementary file 4 [file Presentation_4.zip › Non-targets_2/image_0873.jpg]

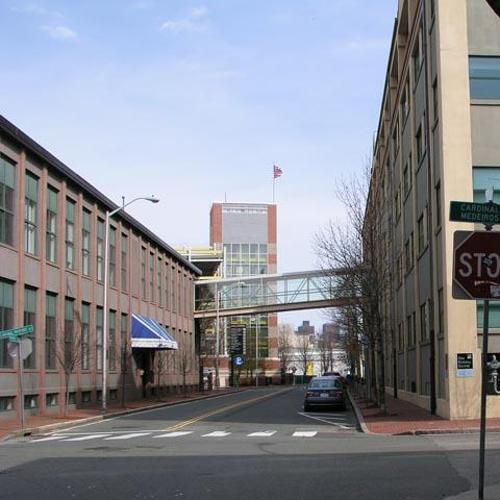

Supplement: Supplementary file 4 [file Presentation_4.zip › Non-targets_2/image_0874.jpg]

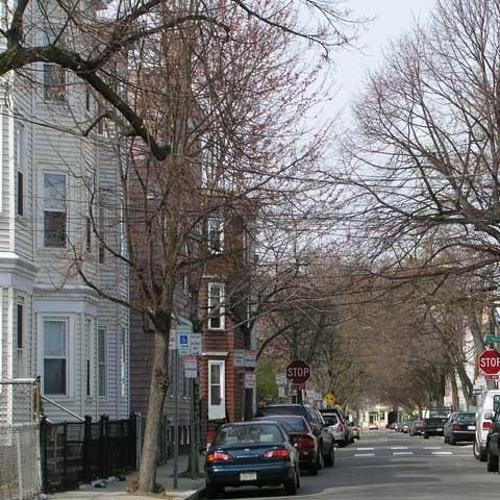

Supplement: Supplementary file 4 [file Presentation_4.zip › Non-targets_2/image_0875.jpg]

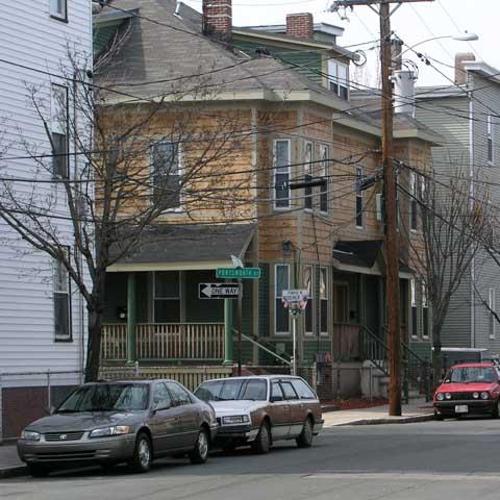

Supplement: Supplementary file 4 [file Presentation_4.zip › Non-targets_2/image_0876.jpg]

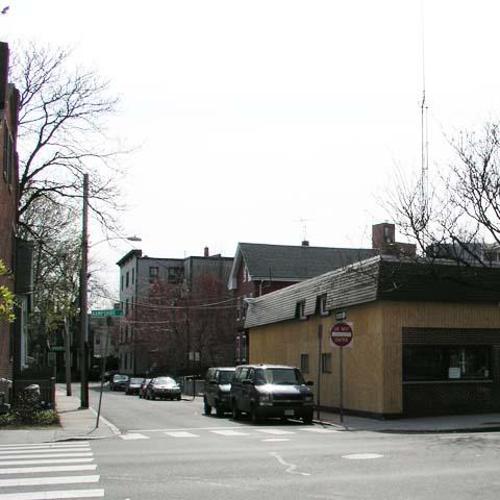

Supplement: Supplementary file 4 [file Presentation_4.zip › Non-targets_2/image_0877.jpg]

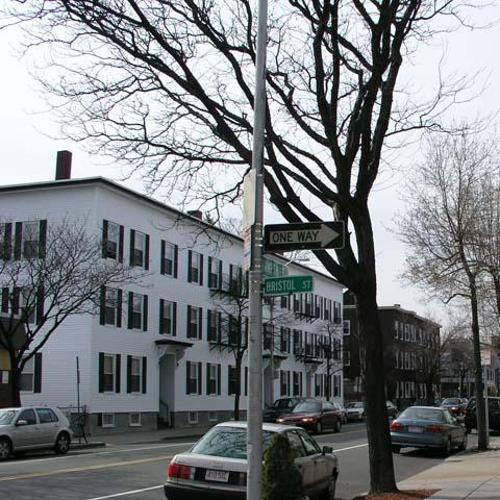

Supplement: Supplementary file 4 [file Presentation_4.zip › Non-targets_2/image_0878.jpg]

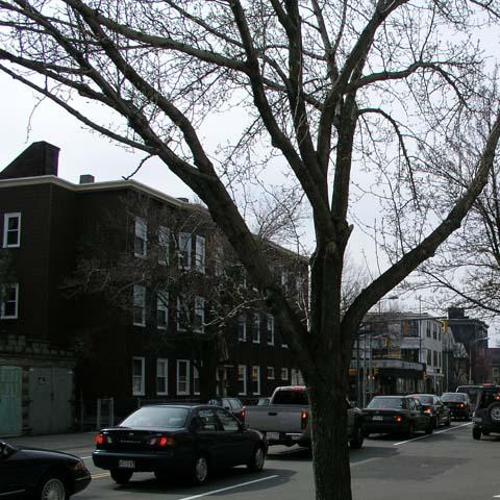

Supplement: Supplementary file 4 [file Presentation_4.zip › Non-targets_2/image_0879.jpg]

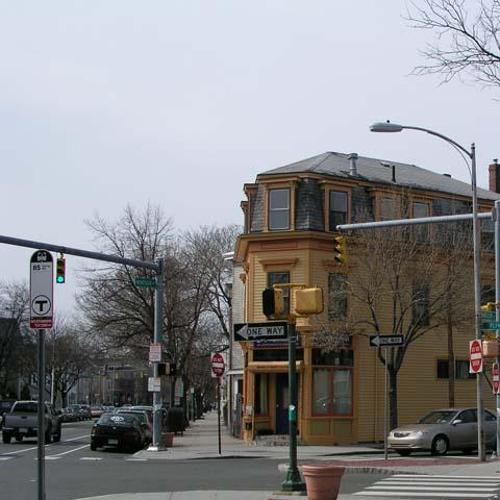

Supplement: Supplementary file 4 [file Presentation_4.zip › Non-targets_2/image_0880.jpg]

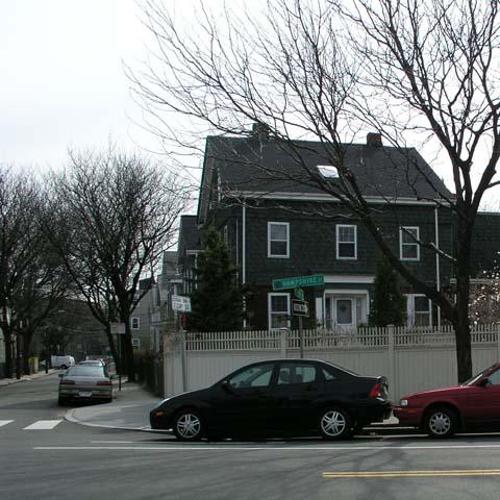

Supplement: Supplementary file 4 [file Presentation_4.zip › Non-targets_2/image_0881.jpg]

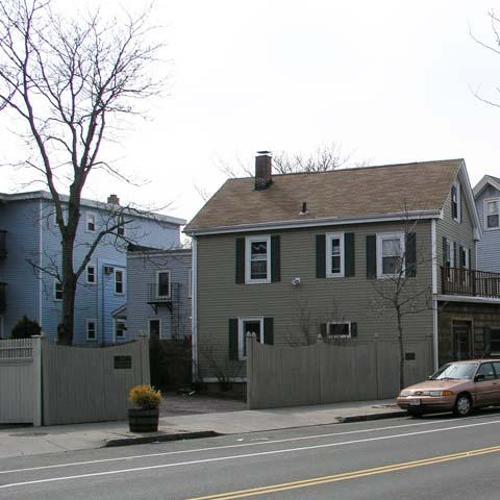

Supplement: Supplementary file 4 [file Presentation_4.zip › Non-targets_2/image_0882.jpg]

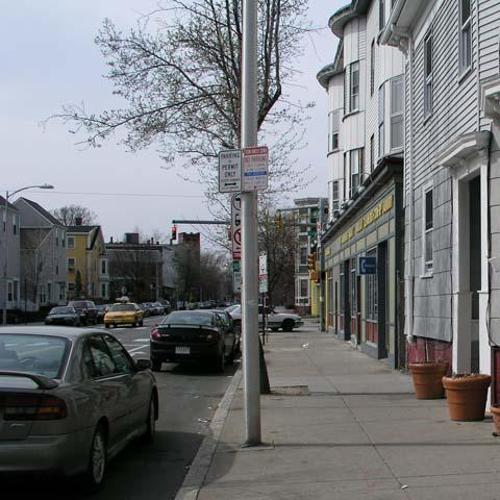

Supplement: Supplementary file 4 [file Presentation_4.zip › Non-targets_2/image_0883.jpg]

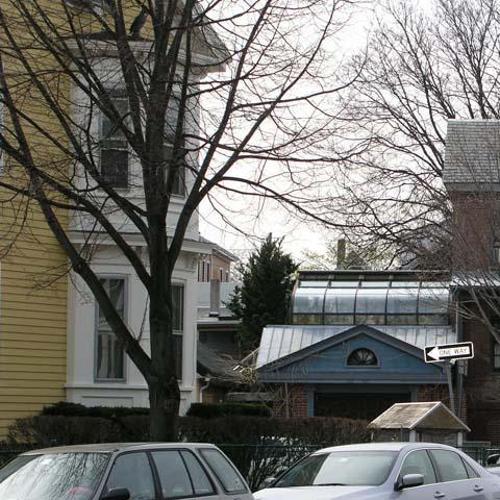

Supplement: Supplementary file 4 [file Presentation_4.zip › Non-targets_2/image_0884.jpg]

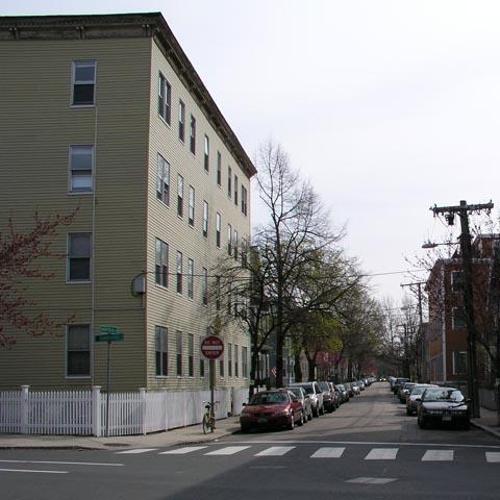

Supplement: Supplementary file 4 [file Presentation_4.zip › Non-targets_2/image_0885.jpg]

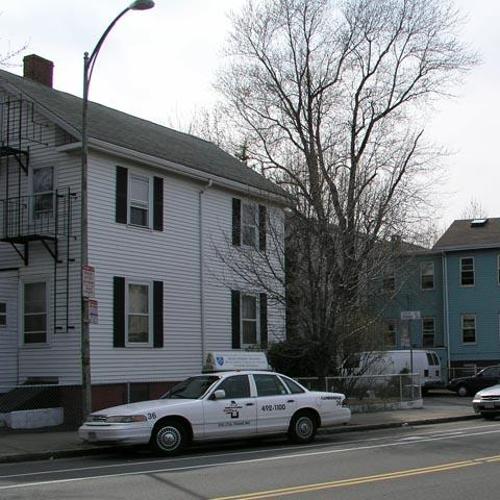

Supplement: Supplementary file 4 [file Presentation_4.zip › Non-targets_2/image_0886.jpg]

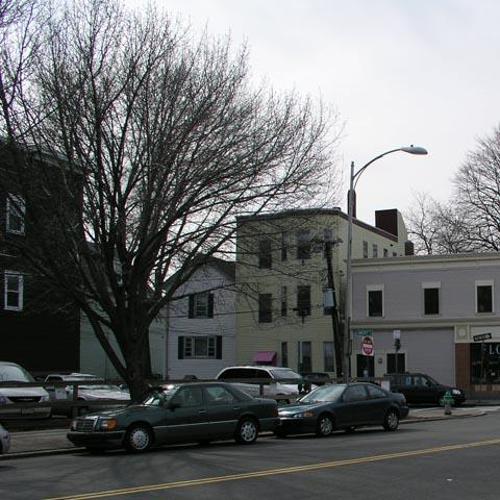

Supplement: Supplementary file 4 [file Presentation_4.zip › Non-targets_2/image_0887.jpg]

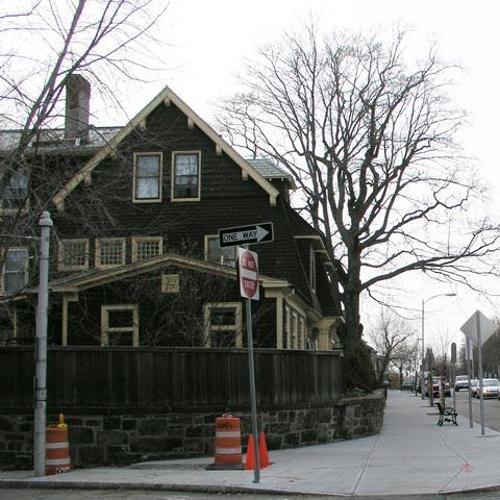

Supplement: Supplementary file 4 [file Presentation_4.zip › Non-targets_2/image_0888.jpg]

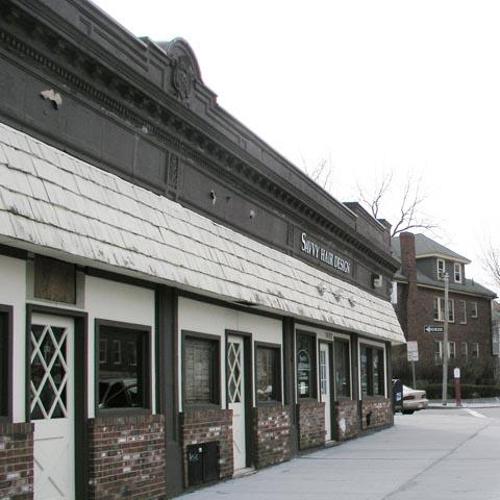

Supplement: Supplementary file 4 [file Presentation_4.zip › Non-targets_2/image_0889.jpg]

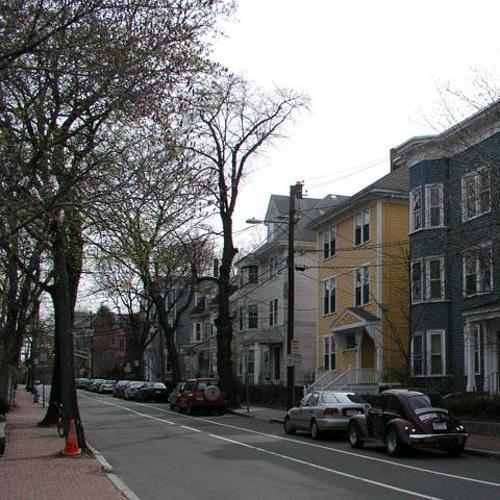

Supplement: Supplementary file 4 [file Presentation_4.zip › Non-targets_2/image_0890.jpg]

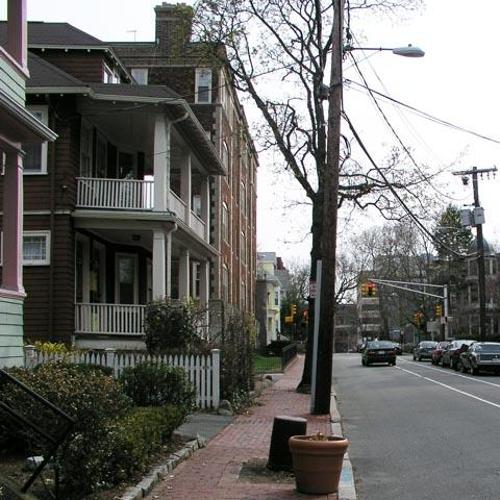

Supplement: Supplementary file 4 [file Presentation_4.zip › Non-targets_2/image_0891.jpg]

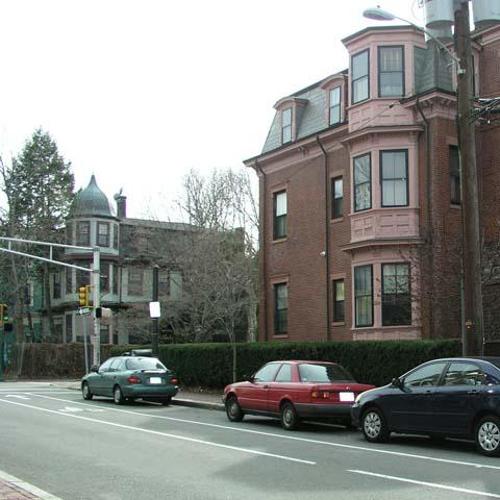

Supplement: Supplementary file 4 [file Presentation_4.zip › Non-targets_2/image_0892.jpg]

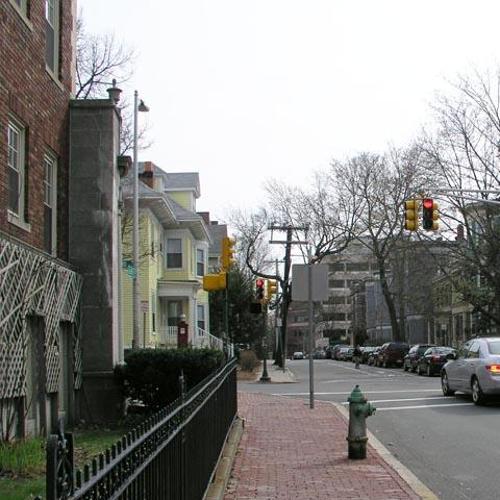

Supplement: Supplementary file 4 [file Presentation_4.zip › Non-targets_2/image_0893.jpg]

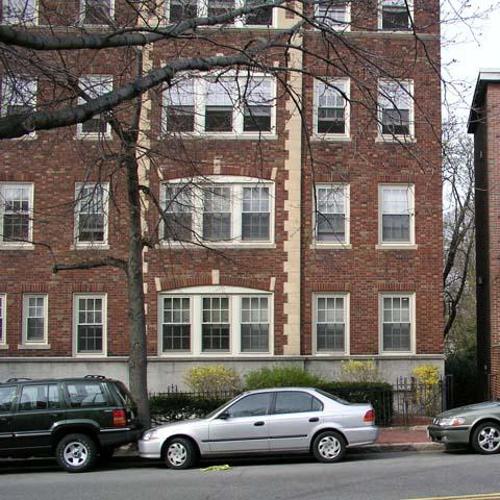

Supplement: Supplementary file 4 [file Presentation_4.zip › Non-targets_2/image_0894.jpg]

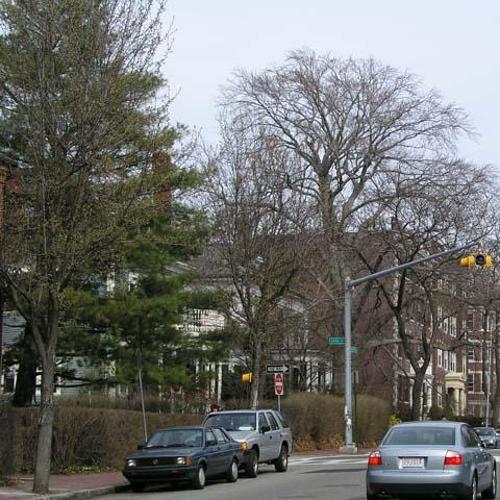

Supplement: Supplementary file 4 [file Presentation_4.zip › Non-targets_2/image_0895.jpg]

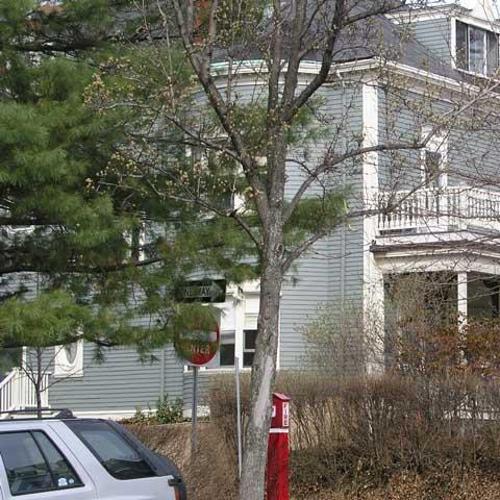

Supplement: Supplementary file 4 [file Presentation_4.zip › Non-targets_2/image_0896.jpg]

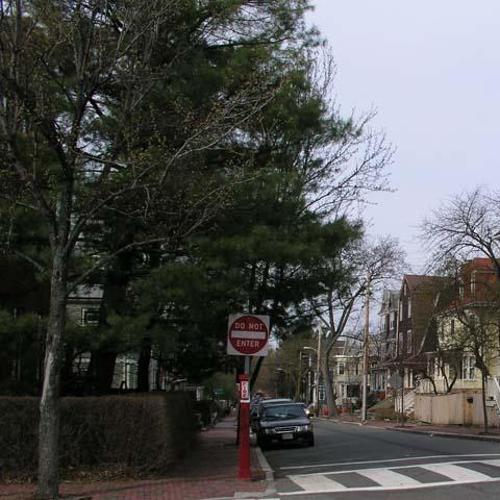

Supplement: Supplementary file 4 [file Presentation_4.zip › Non-targets_2/image_0897.jpg]

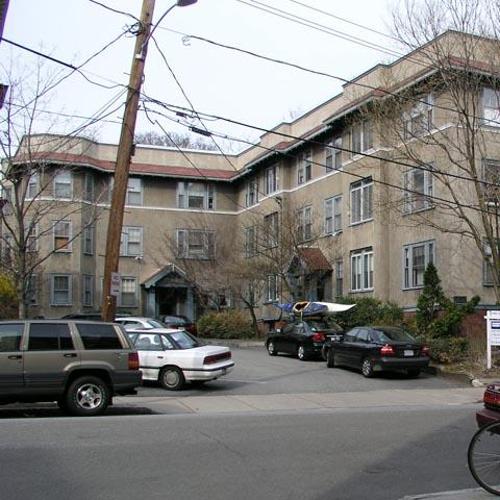

Supplement: Supplementary file 4 [file Presentation_4.zip › Non-targets_2/image_0898.jpg]

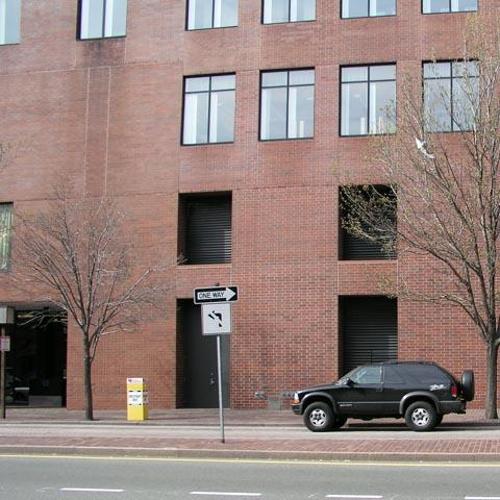

Supplement: Supplementary file 4 [file Presentation_4.zip › Non-targets_2/image_0899.jpg]

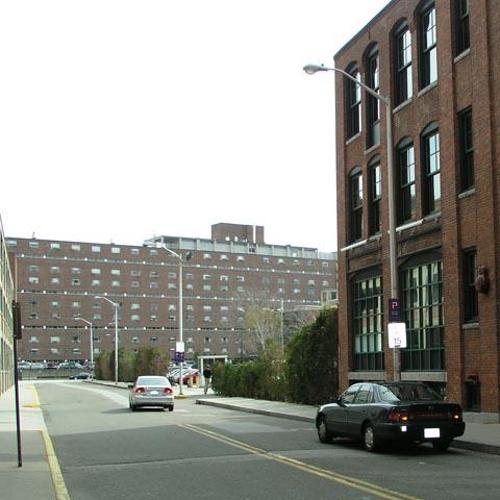

Supplement: Supplementary file 4 [file Presentation_4.zip › Non-targets_2/image_0900.jpg]
