# Supplementary figures and images for: A Cross-Session Dataset for Collaborative Brain-Computer Interfaces Based on Rapid Serial Visual Presentation (part 4 of 5)
Source: Front Neurosci. 2020 Oct 22;14:579469. doi: 10.3389/fnins.2020.579469 (PMC7642747; doi:10.3389/fnins.2020.579469)

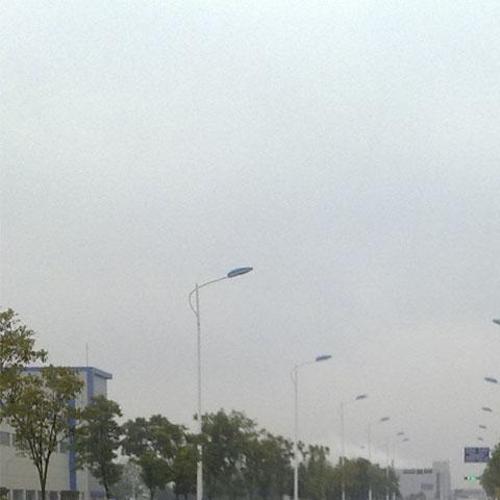

Supplement: Supplementary file 4 [file Presentation_4.zip › Non-targets_2/image_0746.jpg]

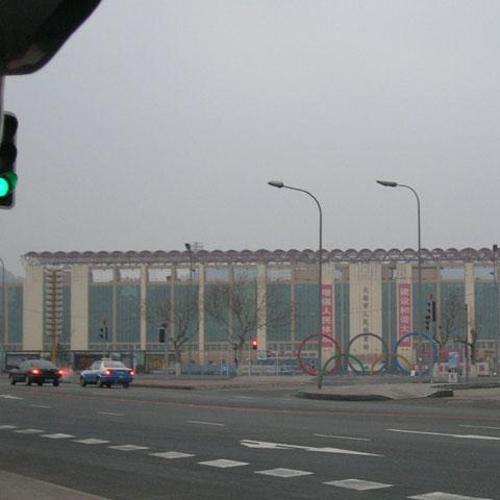

Supplement: Supplementary file 4 [file Presentation_4.zip › Non-targets_2/image_0747.jpg]

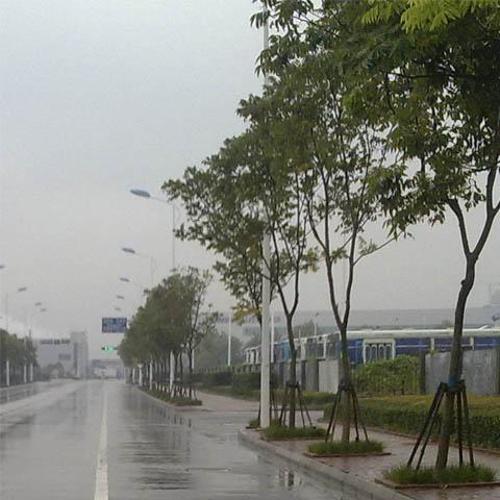

Supplement: Supplementary file 4 [file Presentation_4.zip › Non-targets_2/image_0748.jpg]

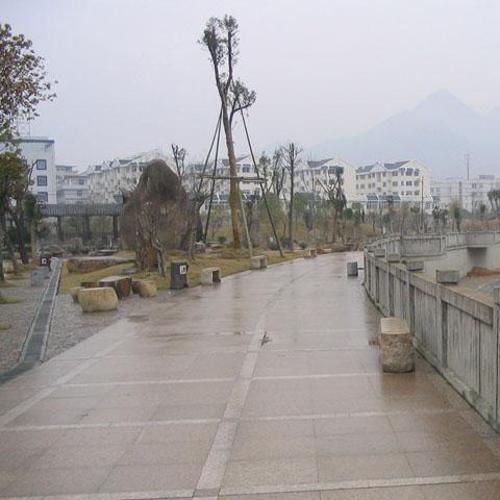

Supplement: Supplementary file 4 [file Presentation_4.zip › Non-targets_2/image_0749.jpg]

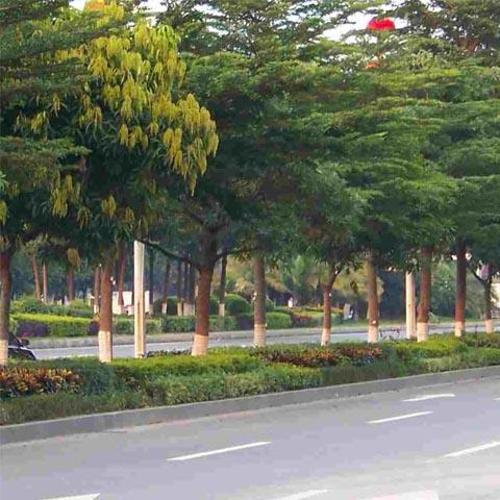

Supplement: Supplementary file 4 [file Presentation_4.zip › Non-targets_2/image_0750.jpg]

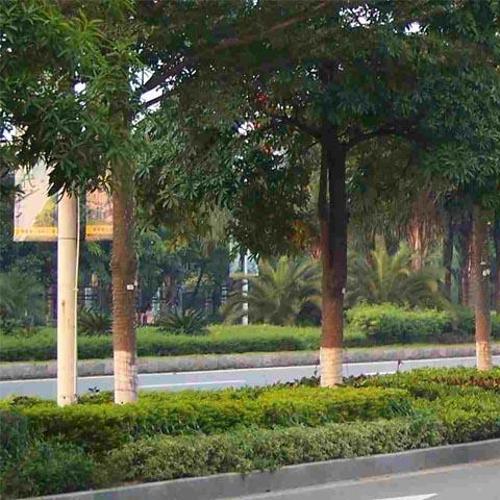

Supplement: Supplementary file 4 [file Presentation_4.zip › Non-targets_2/image_0751.jpg]

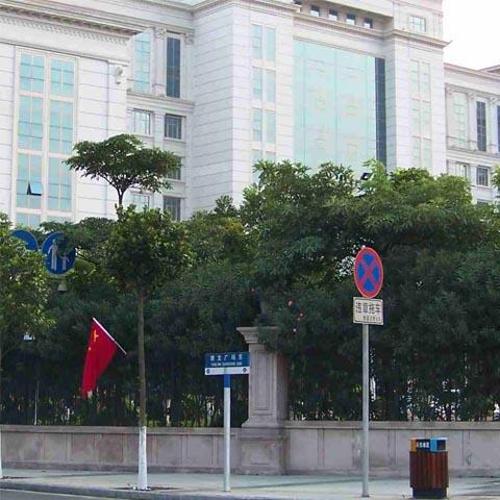

Supplement: Supplementary file 4 [file Presentation_4.zip › Non-targets_2/image_0752.jpg]

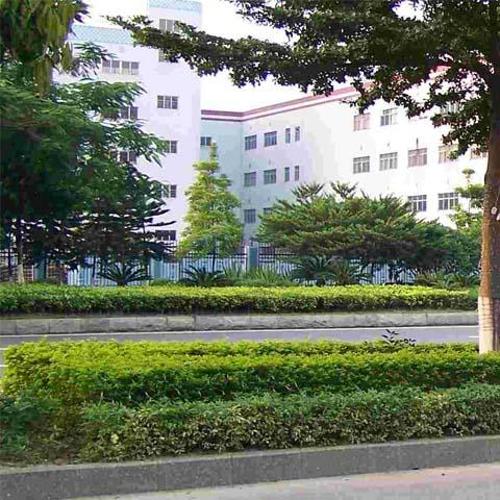

Supplement: Supplementary file 4 [file Presentation_4.zip › Non-targets_2/image_0753.jpg]

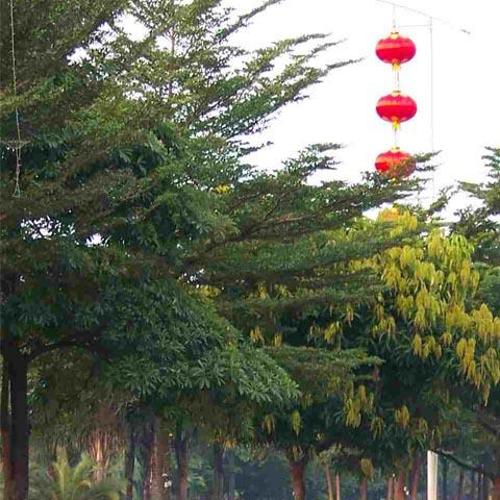

Supplement: Supplementary file 4 [file Presentation_4.zip › Non-targets_2/image_0754.jpg]

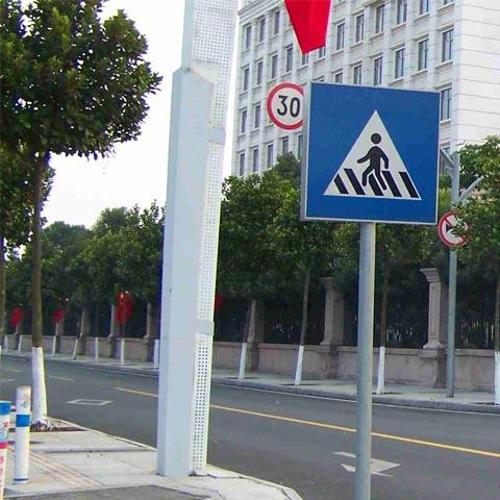

Supplement: Supplementary file 4 [file Presentation_4.zip › Non-targets_2/image_0755.jpg]

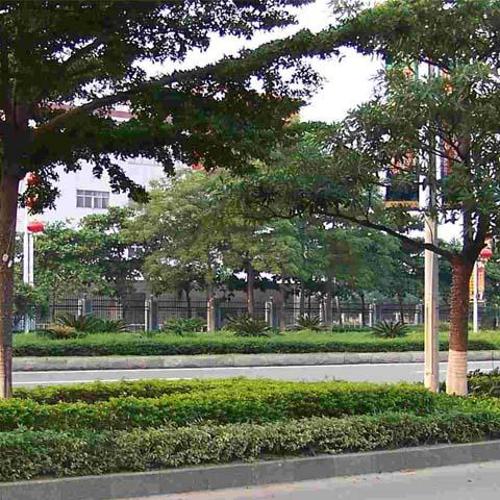

Supplement: Supplementary file 4 [file Presentation_4.zip › Non-targets_2/image_0756.jpg]

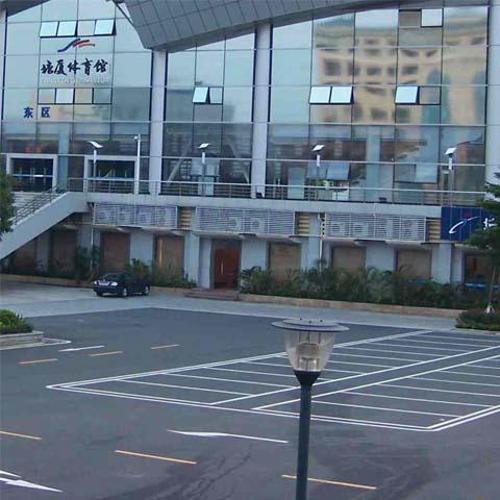

Supplement: Supplementary file 4 [file Presentation_4.zip › Non-targets_2/image_0757.jpg]

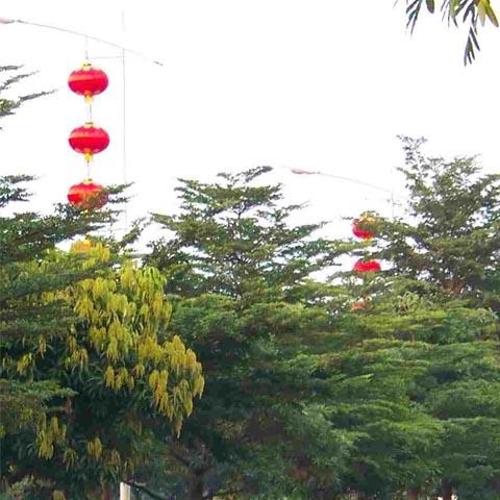

Supplement: Supplementary file 4 [file Presentation_4.zip › Non-targets_2/image_0758.jpg]

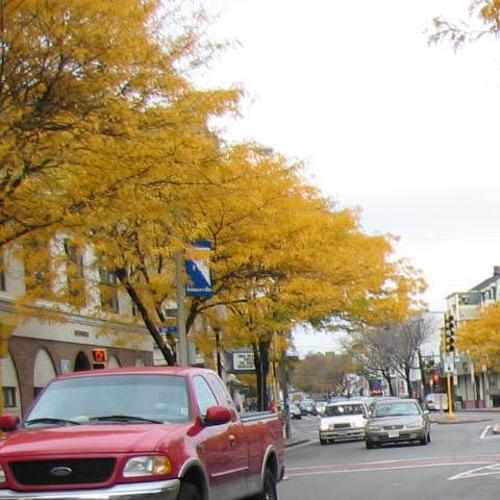

Supplement: Supplementary file 4 [file Presentation_4.zip › Non-targets_2/image_0759.jpg]

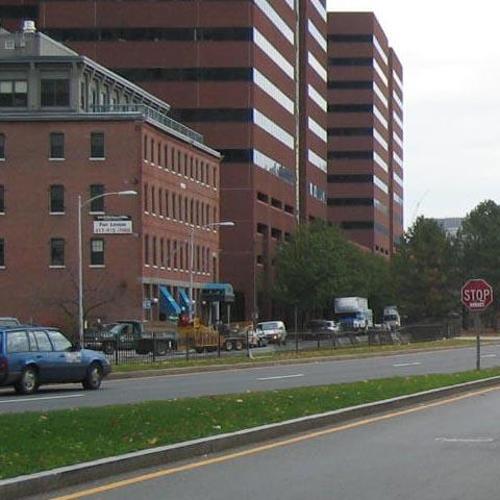

Supplement: Supplementary file 4 [file Presentation_4.zip › Non-targets_2/image_0760.jpg]

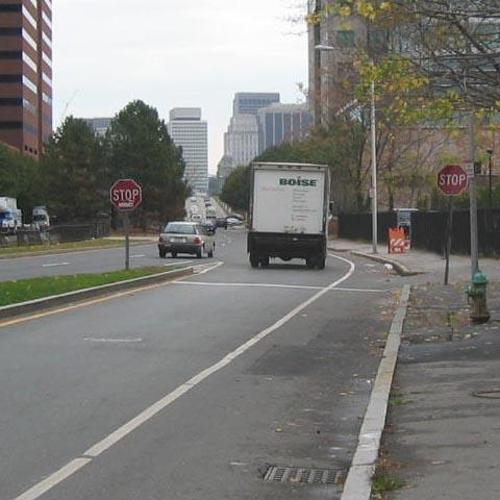

Supplement: Supplementary file 4 [file Presentation_4.zip › Non-targets_2/image_0761.jpg]

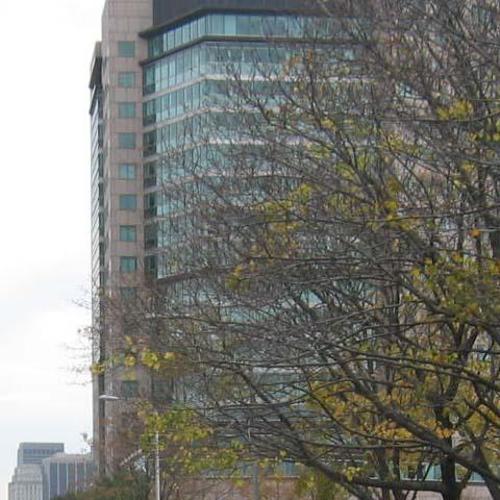

Supplement: Supplementary file 4 [file Presentation_4.zip › Non-targets_2/image_0762.jpg]

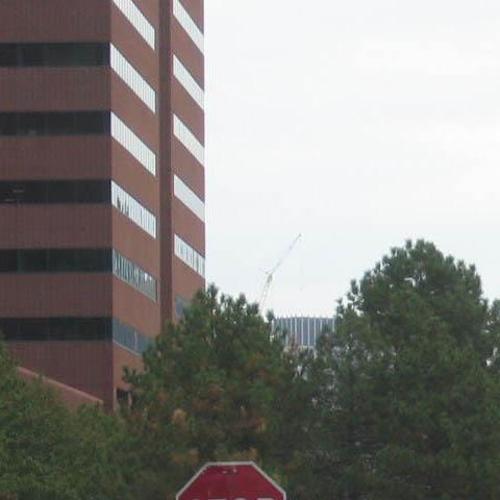

Supplement: Supplementary file 4 [file Presentation_4.zip › Non-targets_2/image_0763.jpg]

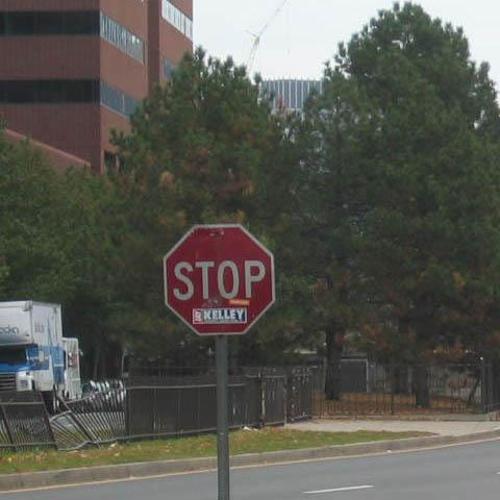

Supplement: Supplementary file 4 [file Presentation_4.zip › Non-targets_2/image_0764.jpg]

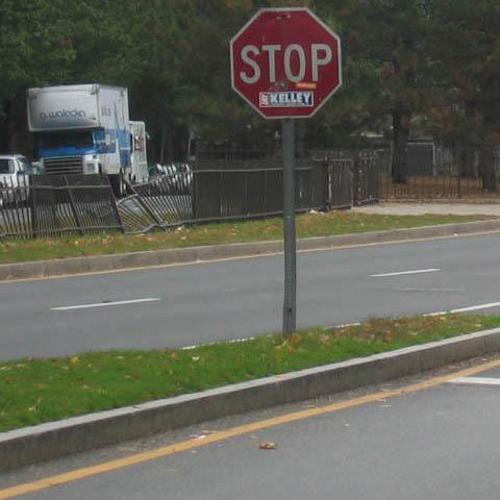

Supplement: Supplementary file 4 [file Presentation_4.zip › Non-targets_2/image_0765.jpg]

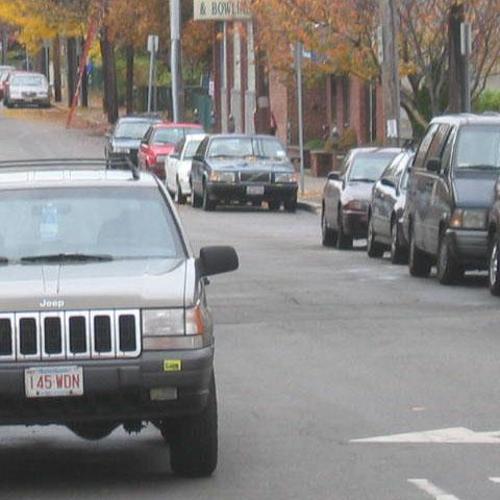

Supplement: Supplementary file 4 [file Presentation_4.zip › Non-targets_2/image_0766.jpg]

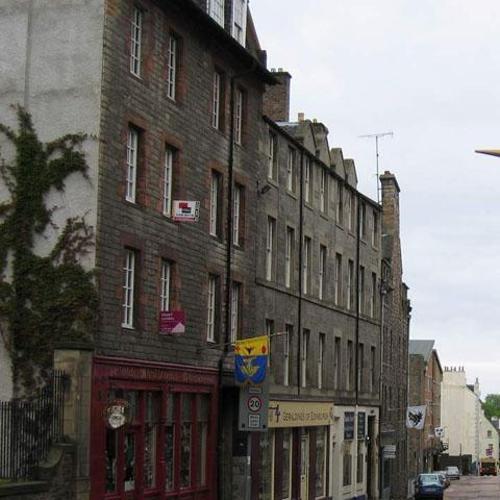

Supplement: Supplementary file 4 [file Presentation_4.zip › Non-targets_2/image_0767.jpg]

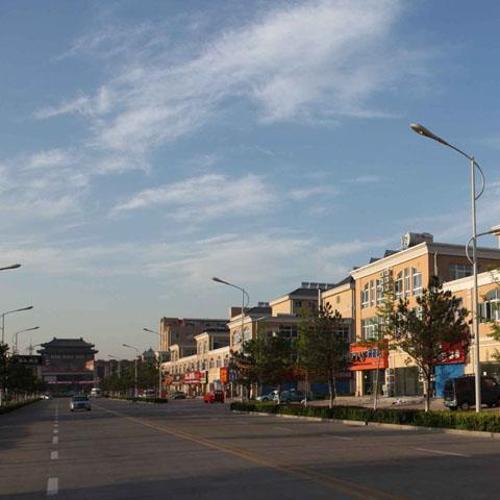

Supplement: Supplementary file 4 [file Presentation_4.zip › Non-targets_2/image_0768.jpg]

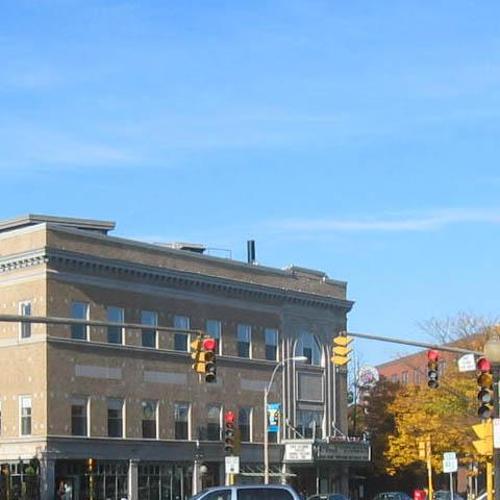

Supplement: Supplementary file 4 [file Presentation_4.zip › Non-targets_2/image_0769.jpg]

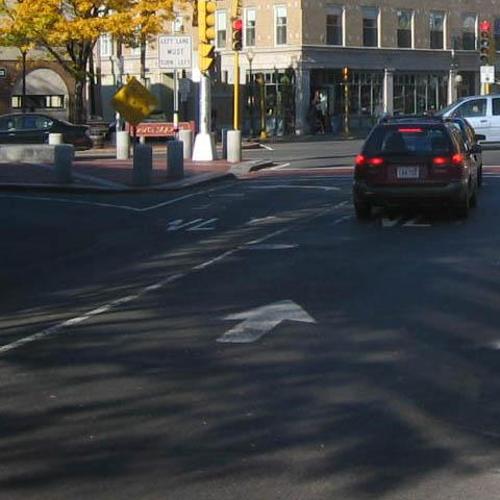

Supplement: Supplementary file 4 [file Presentation_4.zip › Non-targets_2/image_0770.jpg]

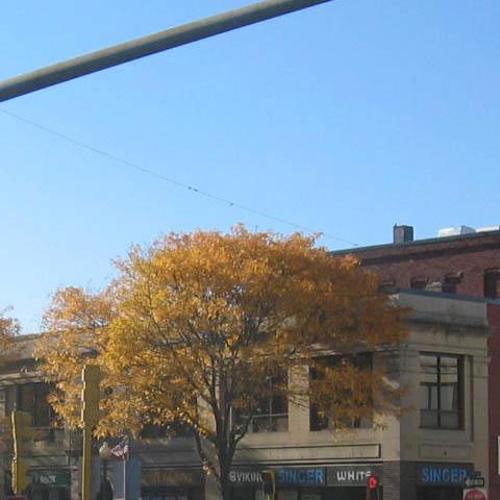

Supplement: Supplementary file 4 [file Presentation_4.zip › Non-targets_2/image_0771.jpg]

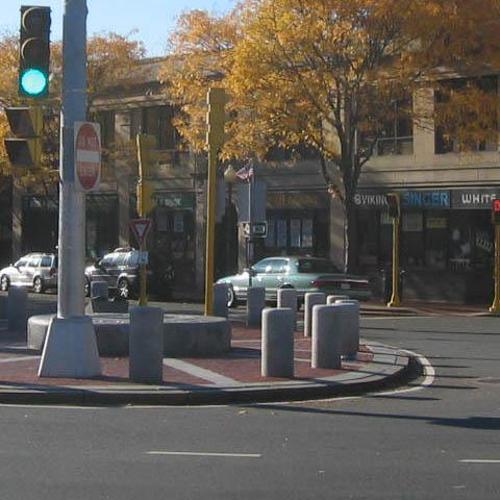

Supplement: Supplementary file 4 [file Presentation_4.zip › Non-targets_2/image_0772.jpg]

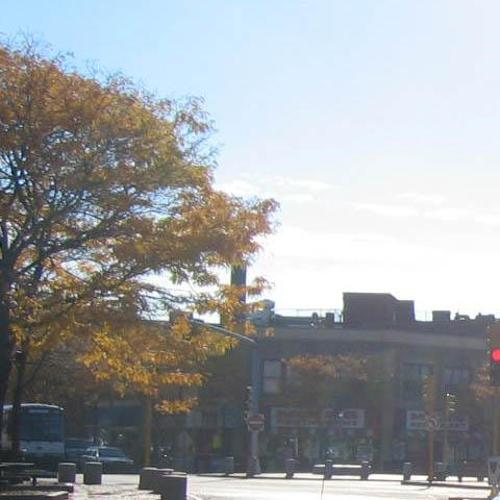

Supplement: Supplementary file 4 [file Presentation_4.zip › Non-targets_2/image_0773.jpg]

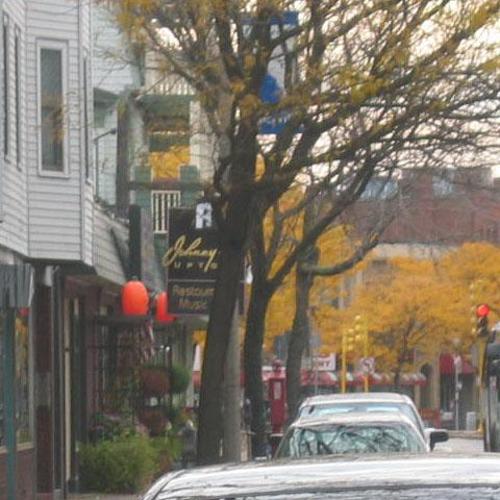

Supplement: Supplementary file 4 [file Presentation_4.zip › Non-targets_2/image_0774.jpg]

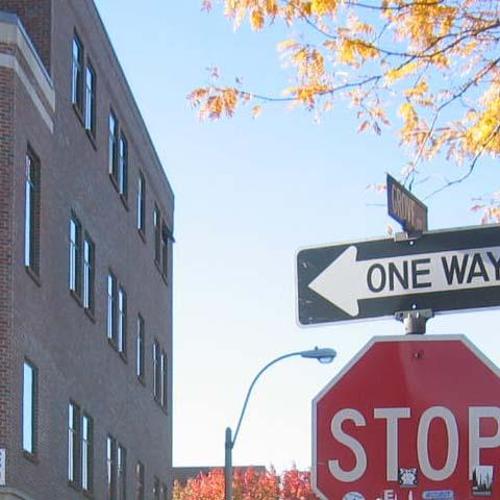

Supplement: Supplementary file 4 [file Presentation_4.zip › Non-targets_2/image_0775.jpg]

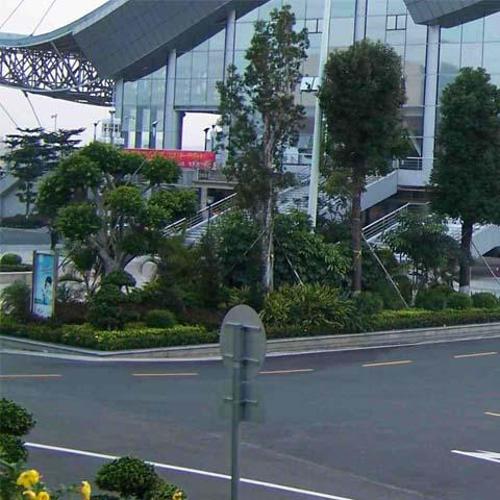

Supplement: Supplementary file 4 [file Presentation_4.zip › Non-targets_2/image_0776.jpg]

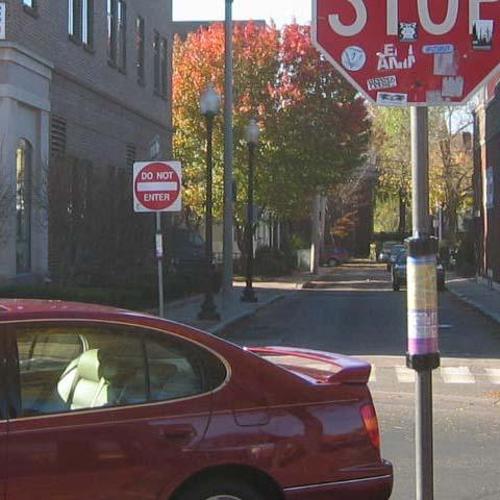

Supplement: Supplementary file 4 [file Presentation_4.zip › Non-targets_2/image_0777.jpg]

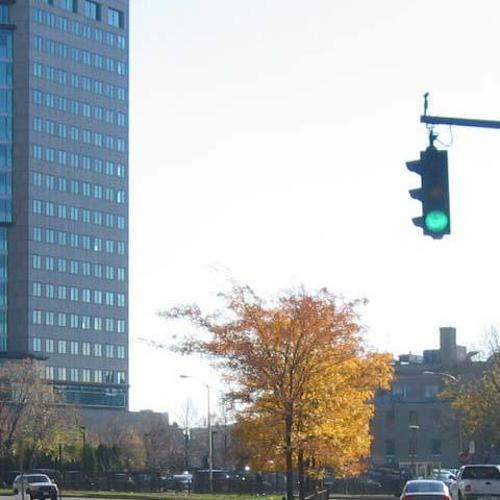

Supplement: Supplementary file 4 [file Presentation_4.zip › Non-targets_2/image_0778.jpg]

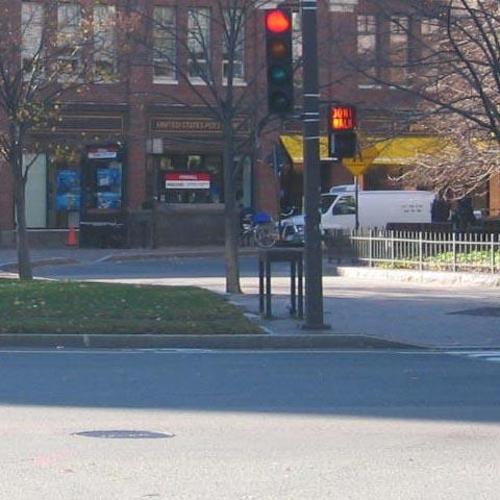

Supplement: Supplementary file 4 [file Presentation_4.zip › Non-targets_2/image_0779.jpg]

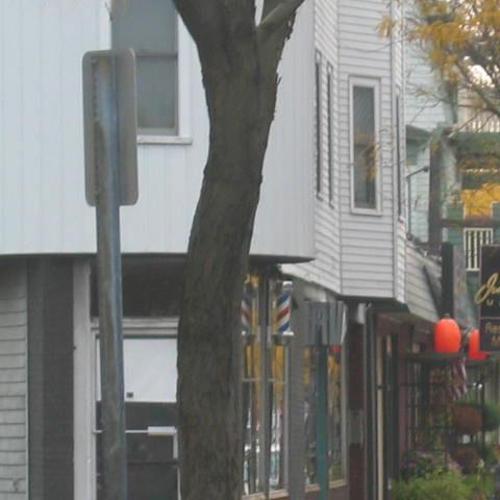

Supplement: Supplementary file 4 [file Presentation_4.zip › Non-targets_2/image_0780.jpg]

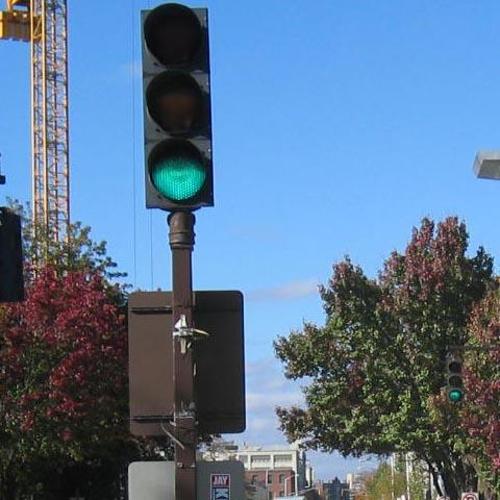

Supplement: Supplementary file 4 [file Presentation_4.zip › Non-targets_2/image_0781.jpg]

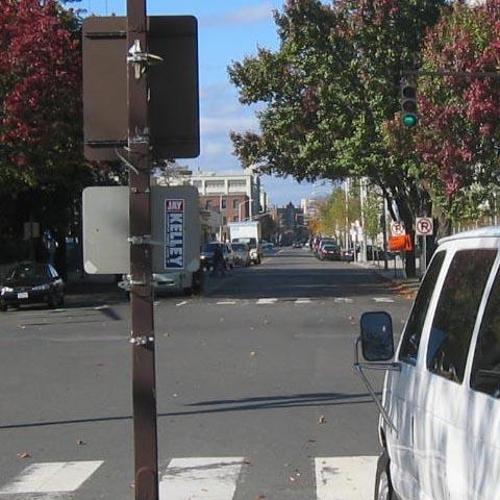

Supplement: Supplementary file 4 [file Presentation_4.zip › Non-targets_2/image_0782.jpg]

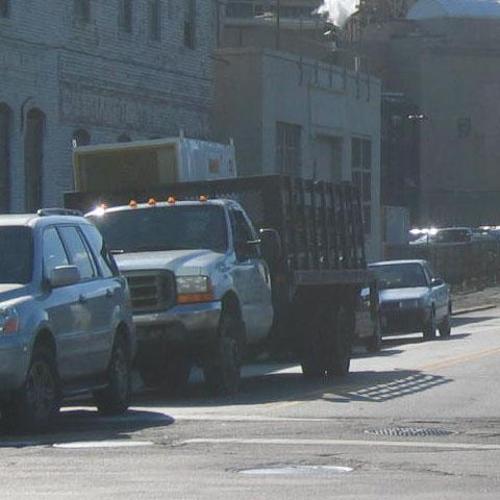

Supplement: Supplementary file 4 [file Presentation_4.zip › Non-targets_2/image_0783.jpg]

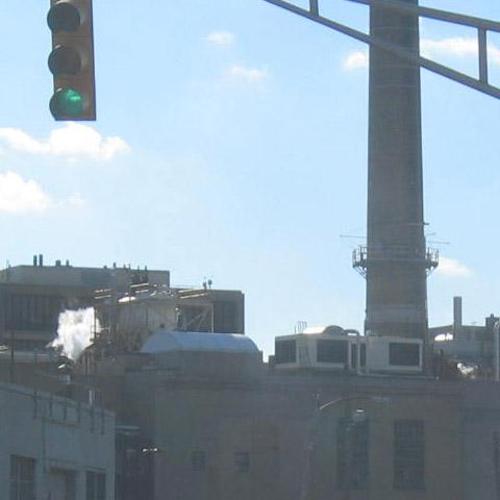

Supplement: Supplementary file 4 [file Presentation_4.zip › Non-targets_2/image_0784.jpg]

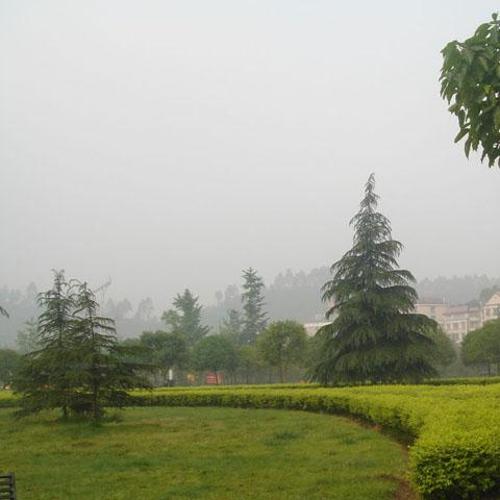

Supplement: Supplementary file 4 [file Presentation_4.zip › Non-targets_2/image_0785.jpg]

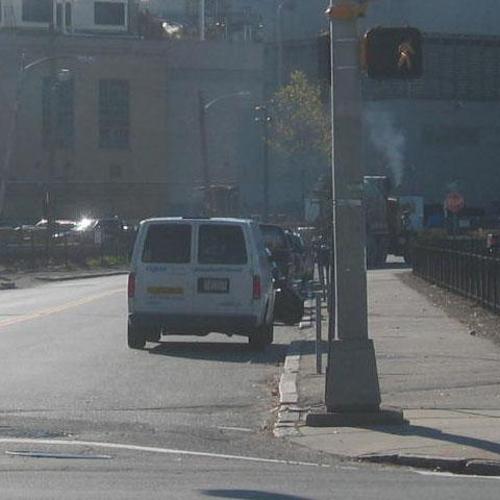

Supplement: Supplementary file 4 [file Presentation_4.zip › Non-targets_2/image_0786.jpg]

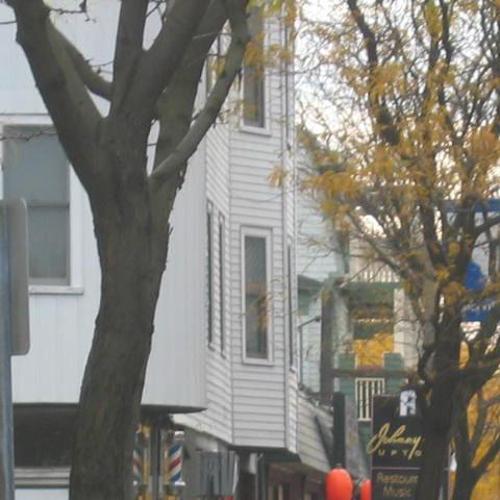

Supplement: Supplementary file 4 [file Presentation_4.zip › Non-targets_2/image_0787.jpg]

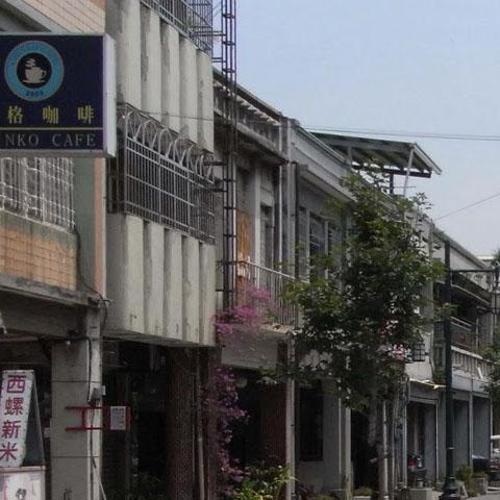

Supplement: Supplementary file 4 [file Presentation_4.zip › Non-targets_2/image_0788.jpg]

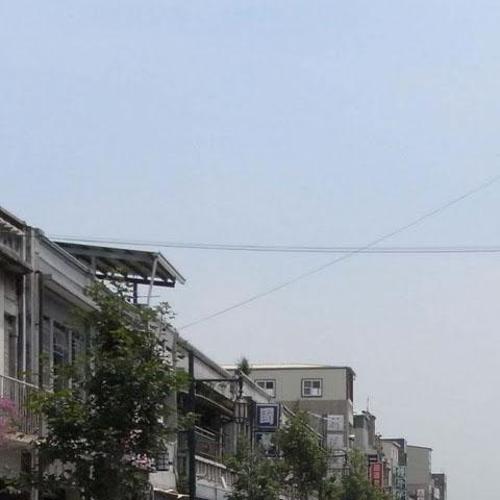

Supplement: Supplementary file 4 [file Presentation_4.zip › Non-targets_2/image_0789.jpg]

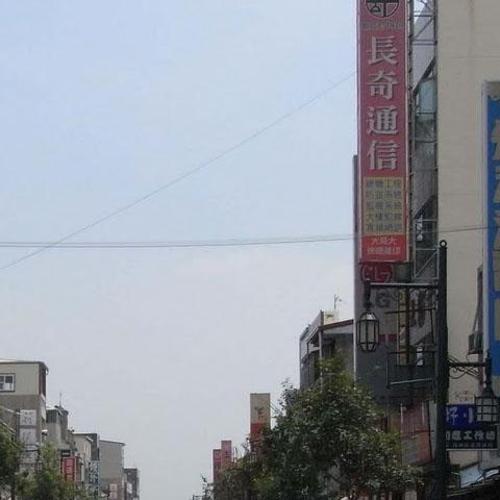

Supplement: Supplementary file 4 [file Presentation_4.zip › Non-targets_2/image_0790.jpg]

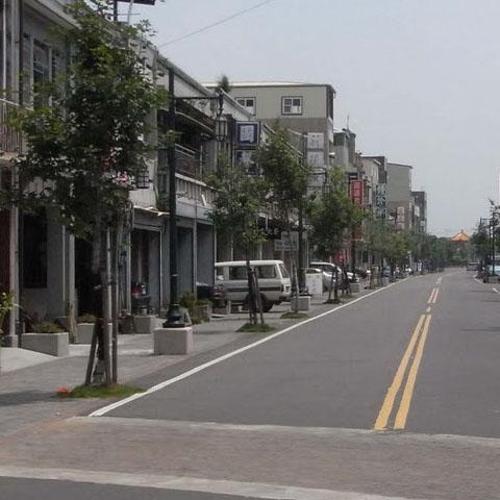

Supplement: Supplementary file 4 [file Presentation_4.zip › Non-targets_2/image_0791.jpg]

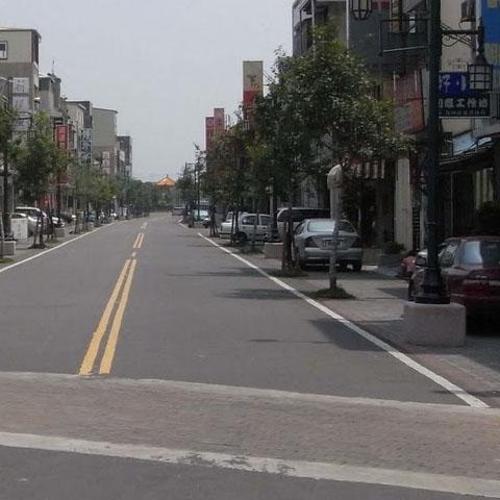

Supplement: Supplementary file 4 [file Presentation_4.zip › Non-targets_2/image_0792.jpg]

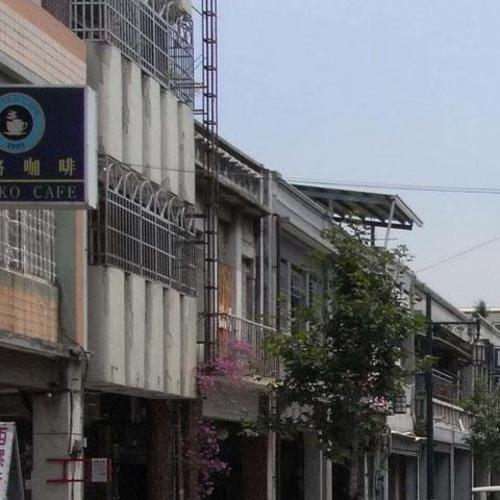

Supplement: Supplementary file 4 [file Presentation_4.zip › Non-targets_2/image_0793.jpg]

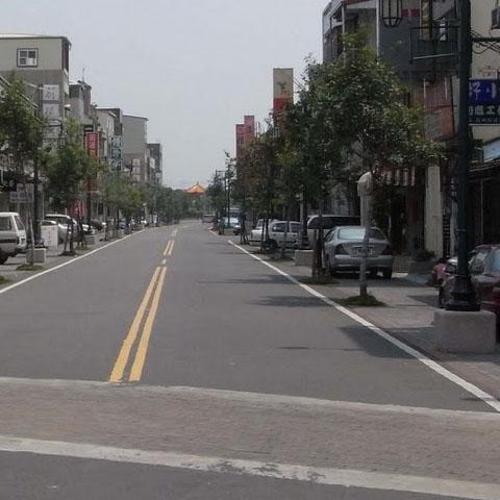

Supplement: Supplementary file 4 [file Presentation_4.zip › Non-targets_2/image_0794.jpg]

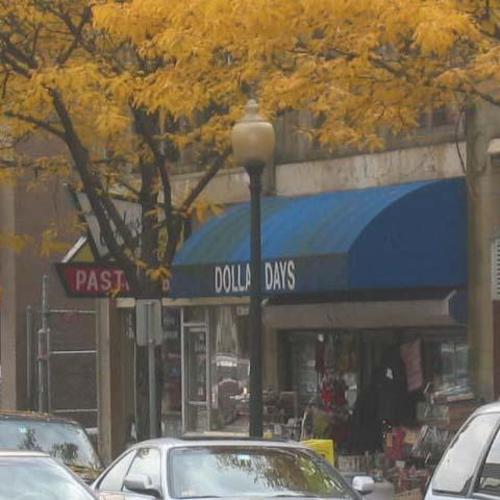

Supplement: Supplementary file 4 [file Presentation_4.zip › Non-targets_2/image_0795.jpg]

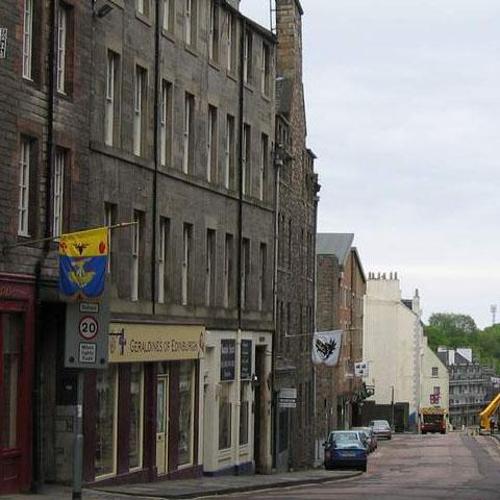

Supplement: Supplementary file 4 [file Presentation_4.zip › Non-targets_2/image_0796.jpg]

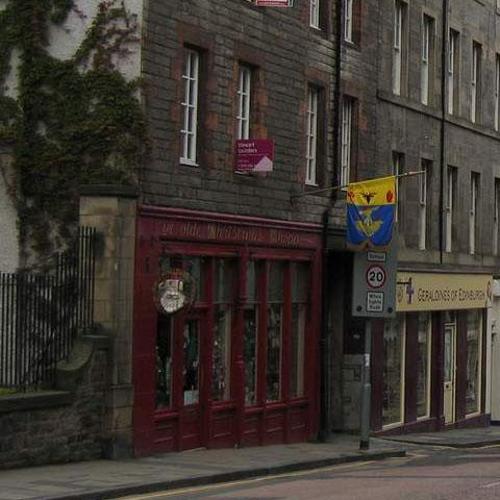

Supplement: Supplementary file 4 [file Presentation_4.zip › Non-targets_2/image_0797.jpg]

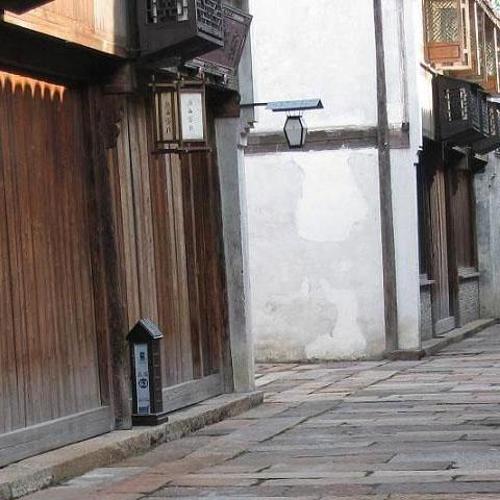

Supplement: Supplementary file 4 [file Presentation_4.zip › Non-targets_2/image_0798.jpg]

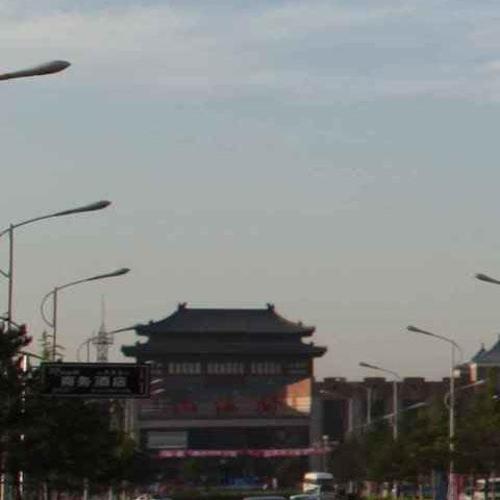

Supplement: Supplementary file 4 [file Presentation_4.zip › Non-targets_2/image_0800.jpg]

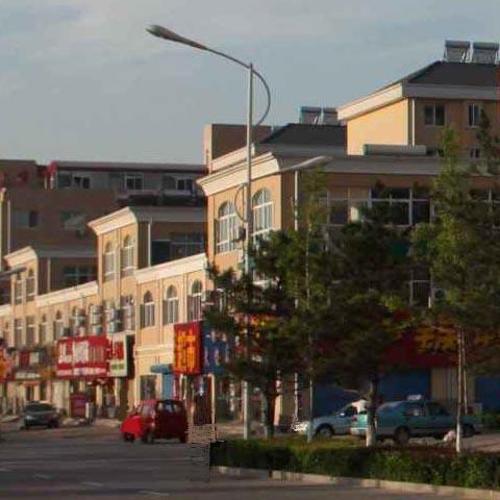

Supplement: Supplementary file 4 [file Presentation_4.zip › Non-targets_2/image_0801.jpg]

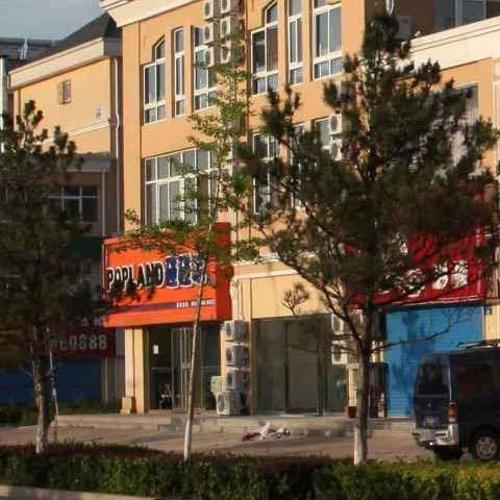

Supplement: Supplementary file 4 [file Presentation_4.zip › Non-targets_2/image_0802.jpg]

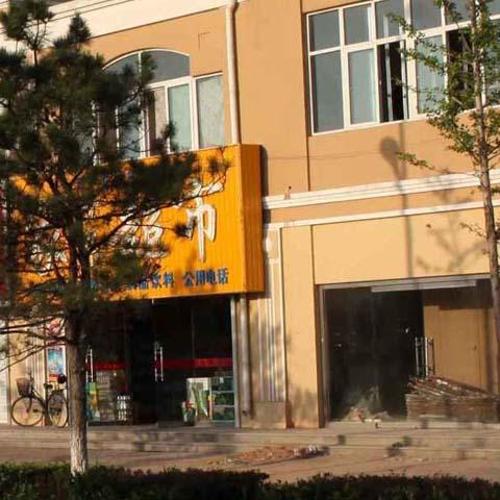

Supplement: Supplementary file 4 [file Presentation_4.zip › Non-targets_2/image_0803.jpg]

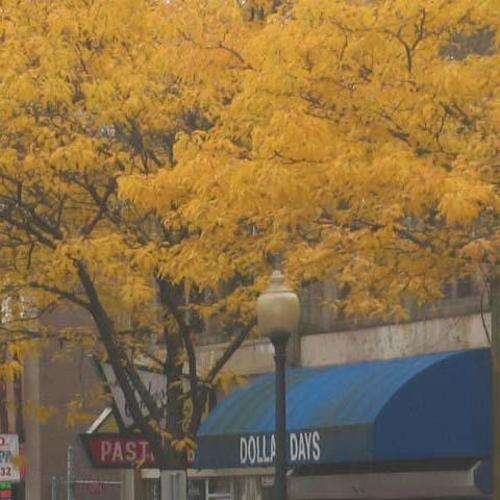

Supplement: Supplementary file 4 [file Presentation_4.zip › Non-targets_2/image_0804.jpg]

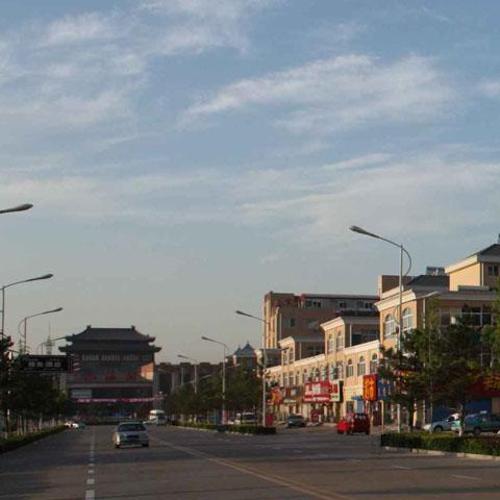

Supplement: Supplementary file 4 [file Presentation_4.zip › Non-targets_2/image_0805.jpg]

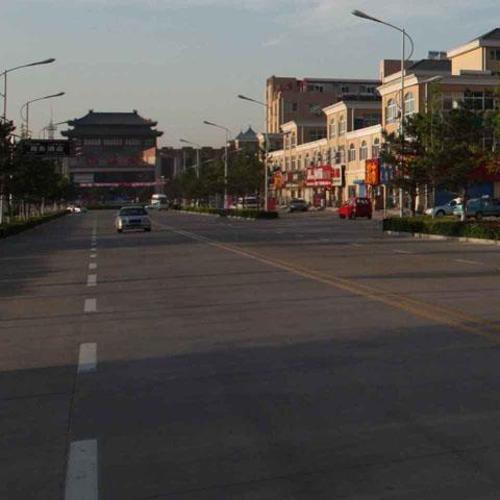

Supplement: Supplementary file 4 [file Presentation_4.zip › Non-targets_2/image_0806.jpg]

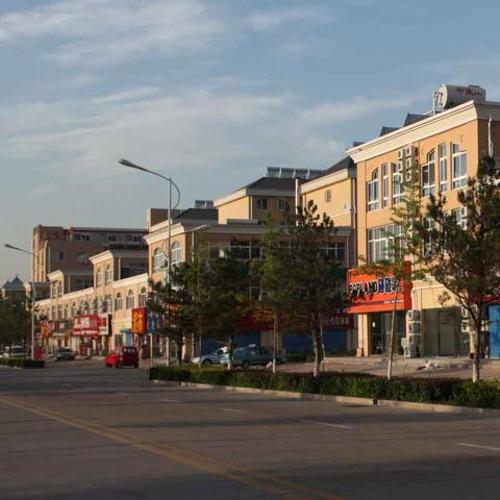

Supplement: Supplementary file 4 [file Presentation_4.zip › Non-targets_2/image_0807.jpg]

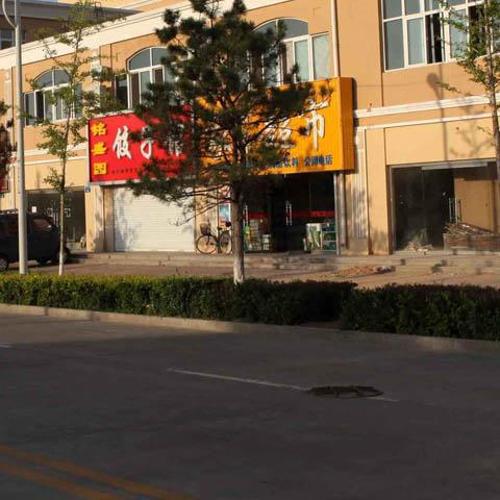

Supplement: Supplementary file 4 [file Presentation_4.zip › Non-targets_2/image_0808.jpg]

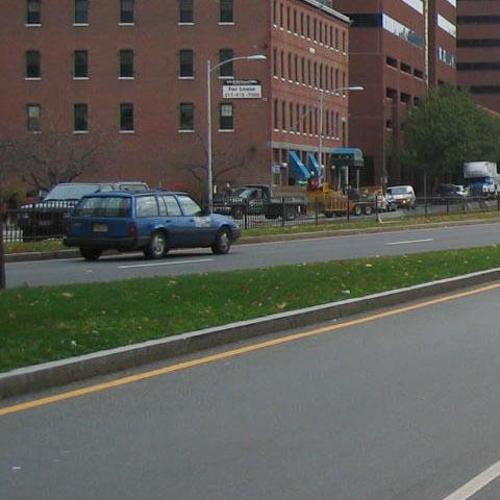

Supplement: Supplementary file 4 [file Presentation_4.zip › Non-targets_2/image_0809.jpg]

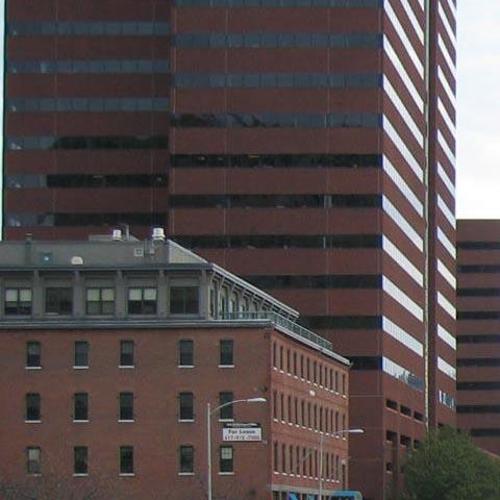

Supplement: Supplementary file 4 [file Presentation_4.zip › Non-targets_2/image_0810.jpg]

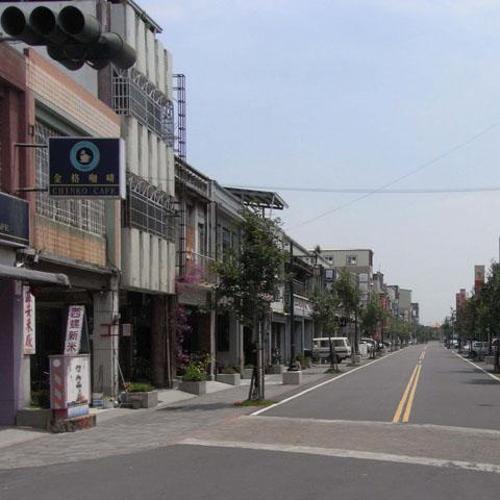

Supplement: Supplementary file 4 [file Presentation_4.zip › Non-targets_2/image_0811.jpg]

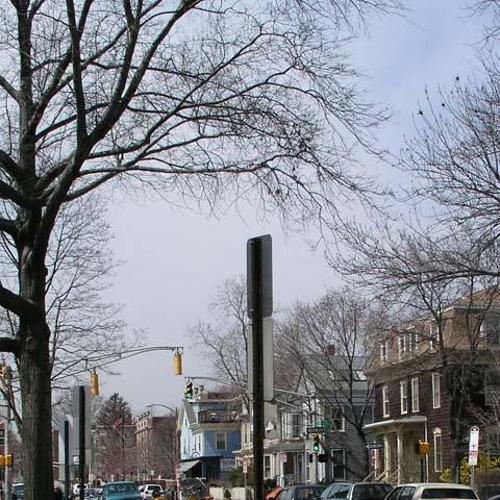

Supplement: Supplementary file 4 [file Presentation_4.zip › Non-targets_2/image_0812.jpg]

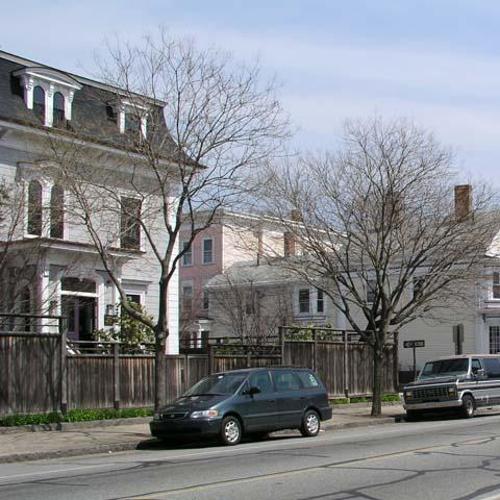

Supplement: Supplementary file 4 [file Presentation_4.zip › Non-targets_2/image_0813.jpg]

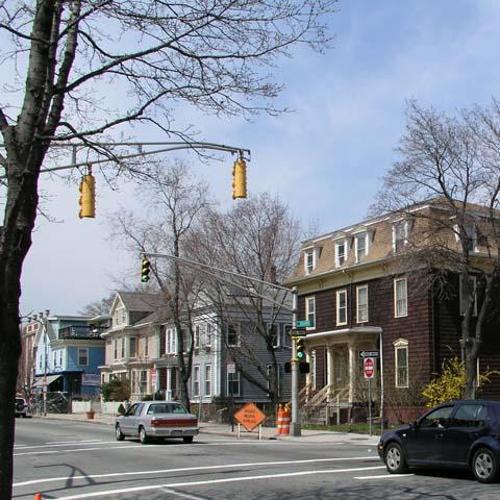

Supplement: Supplementary file 4 [file Presentation_4.zip › Non-targets_2/image_0814.jpg]

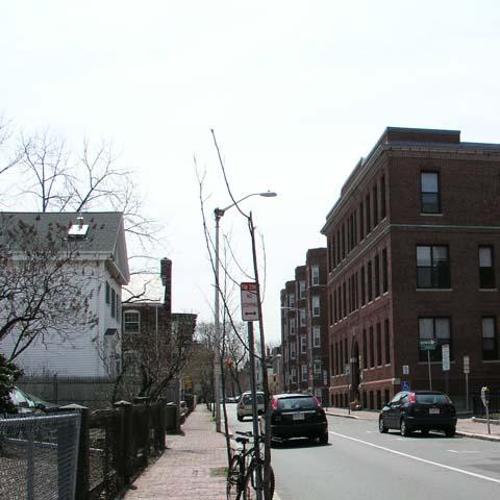

Supplement: Supplementary file 4 [file Presentation_4.zip › Non-targets_2/image_0815.jpg]

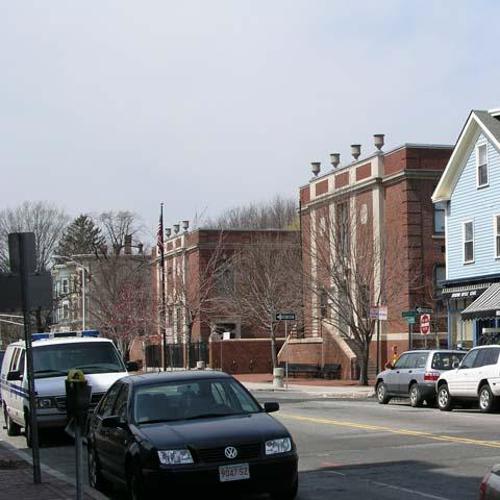

Supplement: Supplementary file 4 [file Presentation_4.zip › Non-targets_2/image_0816.jpg]

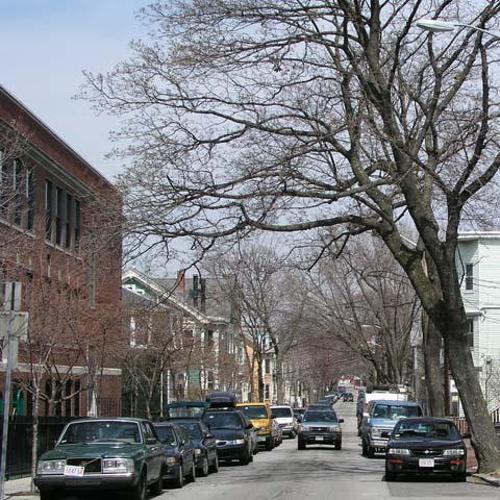

Supplement: Supplementary file 4 [file Presentation_4.zip › Non-targets_2/image_0817.jpg]

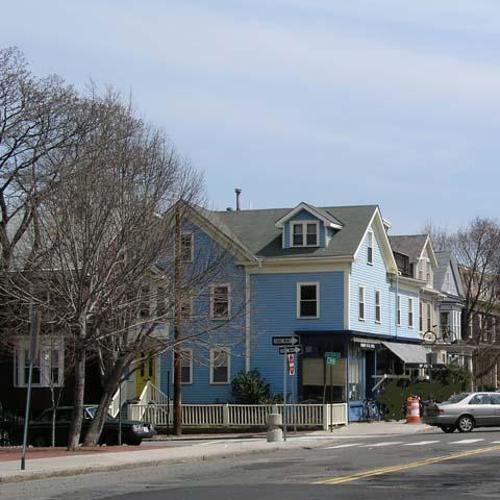

Supplement: Supplementary file 4 [file Presentation_4.zip › Non-targets_2/image_0818.jpg]

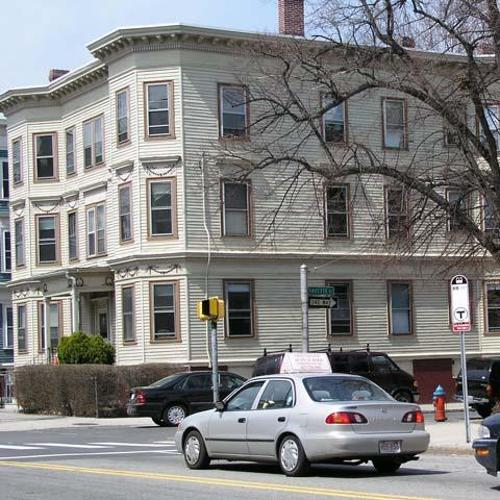

Supplement: Supplementary file 4 [file Presentation_4.zip › Non-targets_2/image_0819.jpg]

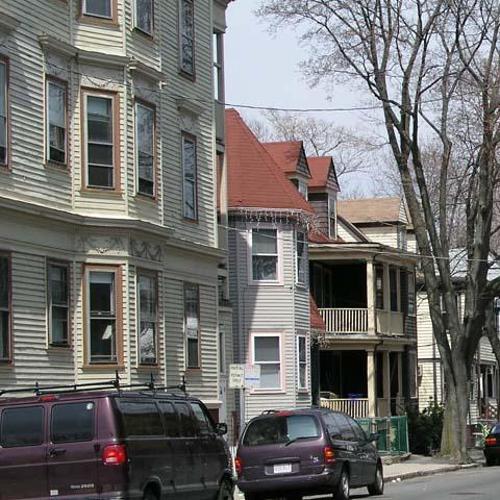

Supplement: Supplementary file 4 [file Presentation_4.zip › Non-targets_2/image_0820.jpg]

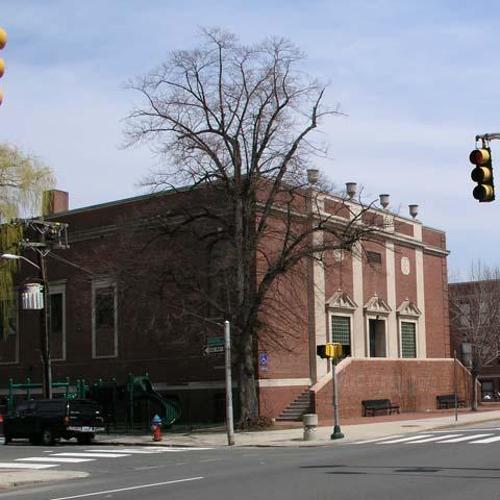

Supplement: Supplementary file 4 [file Presentation_4.zip › Non-targets_2/image_0821.jpg]

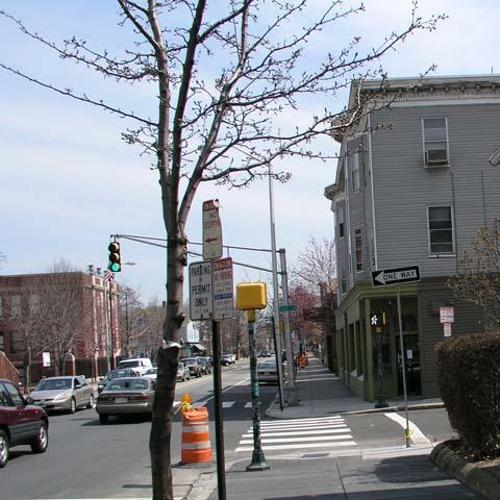

Supplement: Supplementary file 4 [file Presentation_4.zip › Non-targets_2/image_0822.jpg]

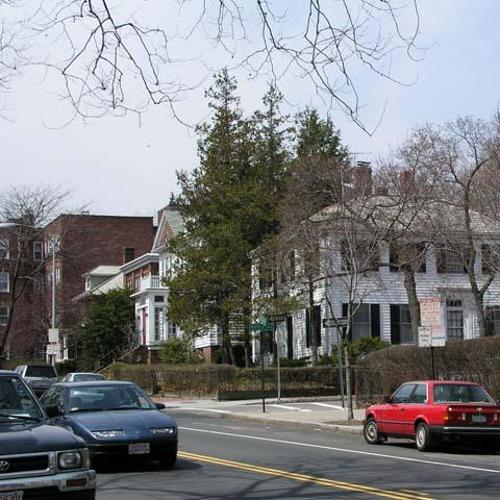

Supplement: Supplementary file 4 [file Presentation_4.zip › Non-targets_2/image_0823.jpg]

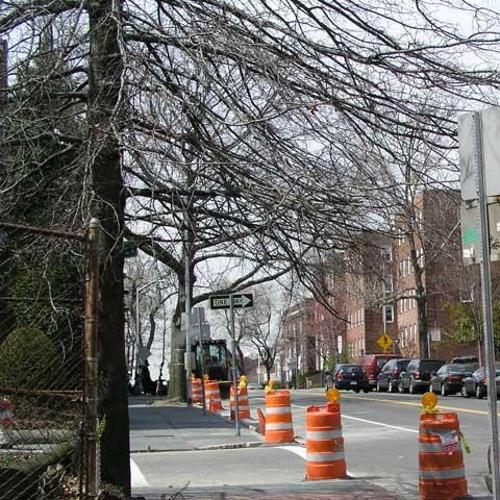

Supplement: Supplementary file 4 [file Presentation_4.zip › Non-targets_2/image_0824.jpg]

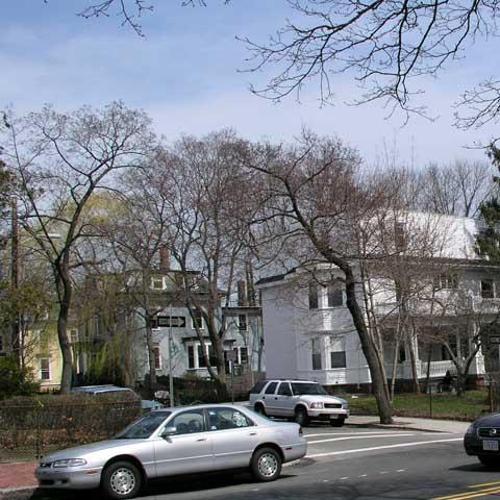

Supplement: Supplementary file 4 [file Presentation_4.zip › Non-targets_2/image_0825.jpg]

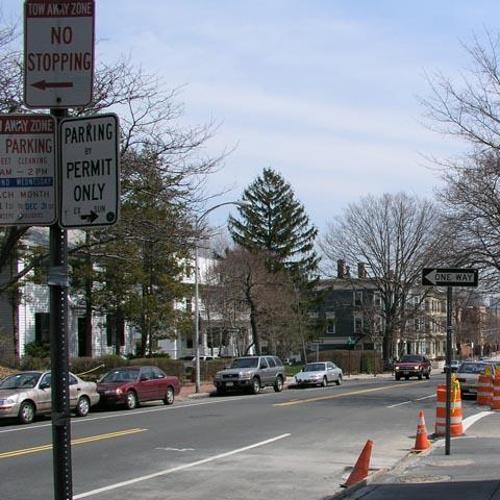

Supplement: Supplementary file 4 [file Presentation_4.zip › Non-targets_2/image_0826.jpg]

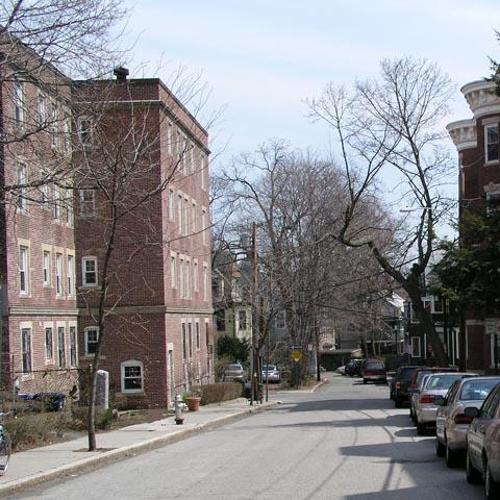

Supplement: Supplementary file 4 [file Presentation_4.zip › Non-targets_2/image_0827.jpg]

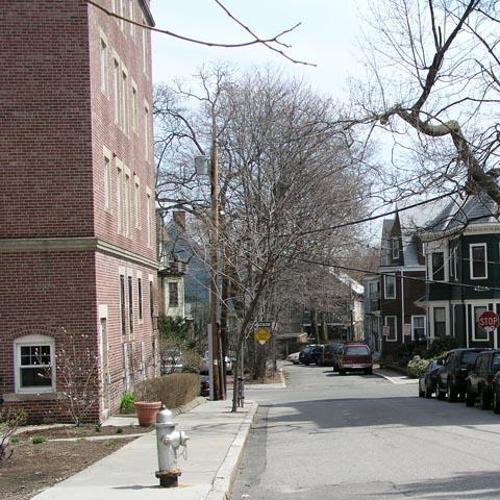

Supplement: Supplementary file 4 [file Presentation_4.zip › Non-targets_2/image_0828.jpg]

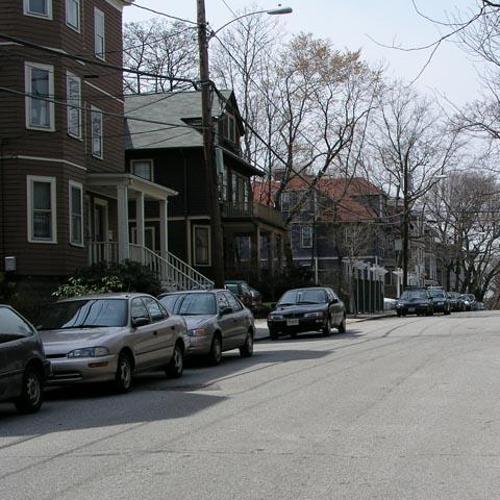

Supplement: Supplementary file 4 [file Presentation_4.zip › Non-targets_2/image_0829.jpg]

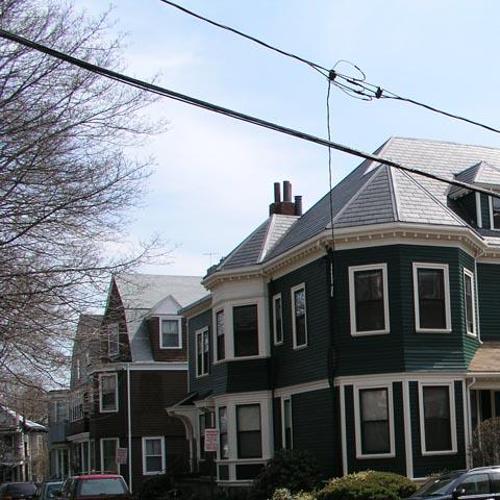

Supplement: Supplementary file 4 [file Presentation_4.zip › Non-targets_2/image_0830.jpg]

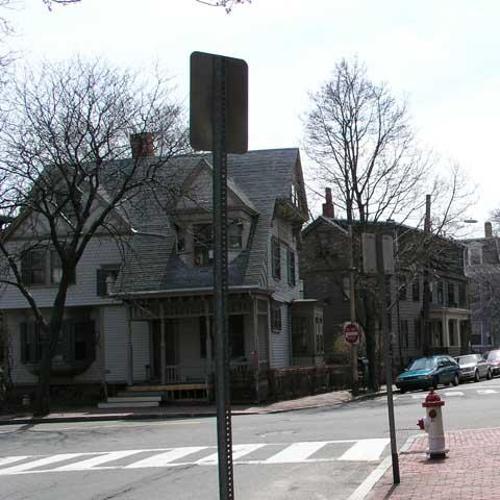

Supplement: Supplementary file 4 [file Presentation_4.zip › Non-targets_2/image_0831.jpg]

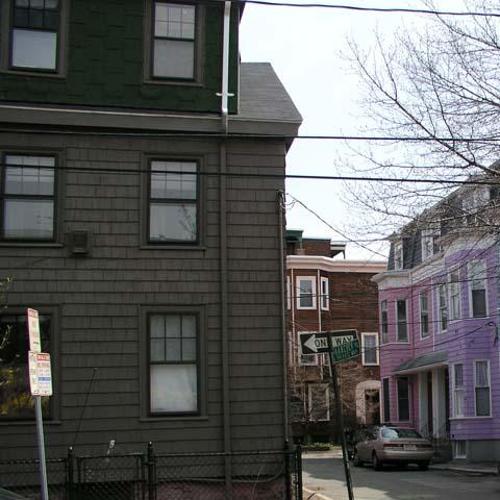

Supplement: Supplementary file 4 [file Presentation_4.zip › Non-targets_2/image_0832.jpg]

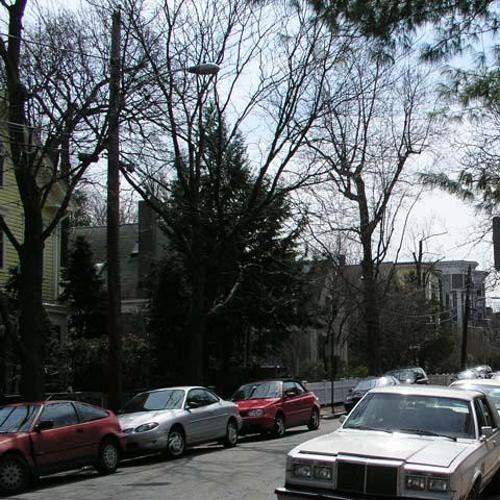

Supplement: Supplementary file 4 [file Presentation_4.zip › Non-targets_2/image_0833.jpg]

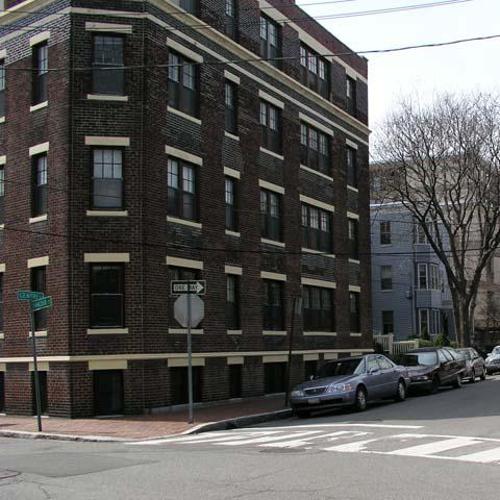

Supplement: Supplementary file 4 [file Presentation_4.zip › Non-targets_2/image_0834.jpg]

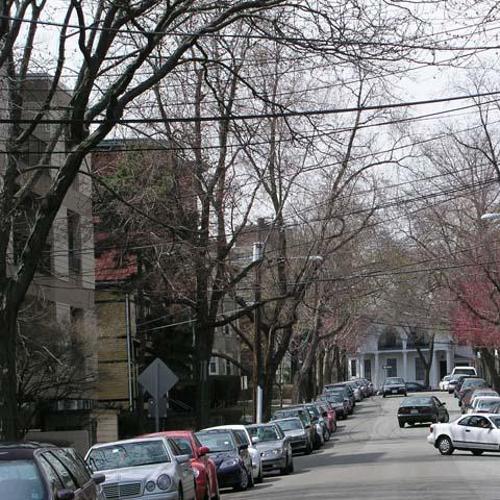

Supplement: Supplementary file 4 [file Presentation_4.zip › Non-targets_2/image_0835.jpg]

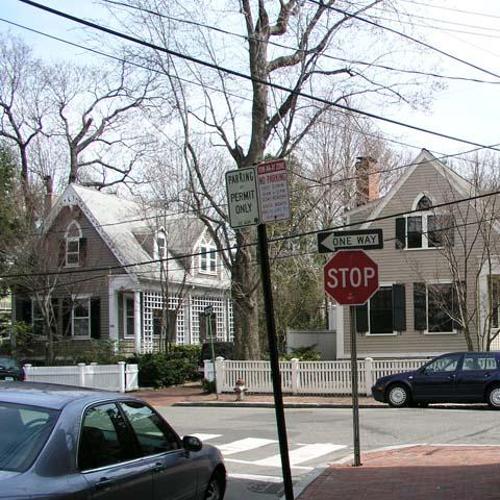

Supplement: Supplementary file 4 [file Presentation_4.zip › Non-targets_2/image_0836.jpg]

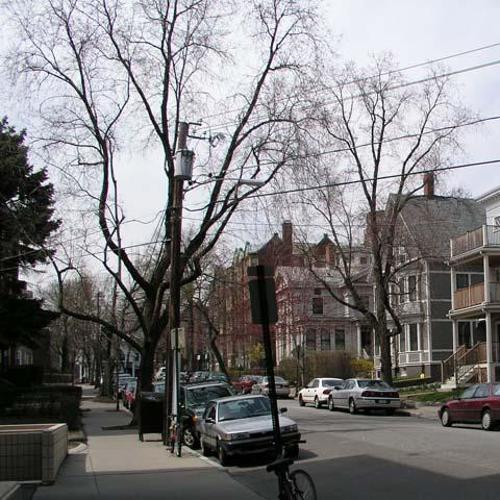

Supplement: Supplementary file 4 [file Presentation_4.zip › Non-targets_2/image_0837.jpg]

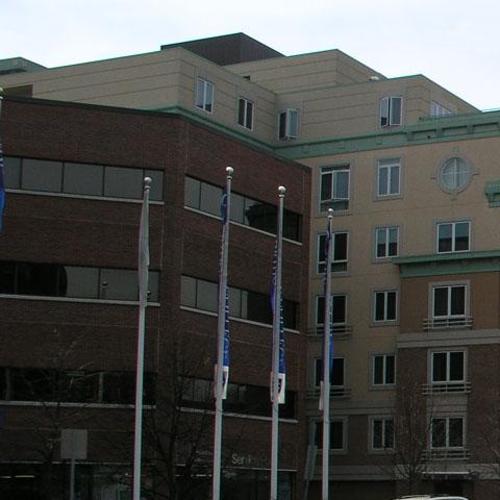

Supplement: Supplementary file 4 [file Presentation_4.zip › Non-targets_2/image_0838.jpg]

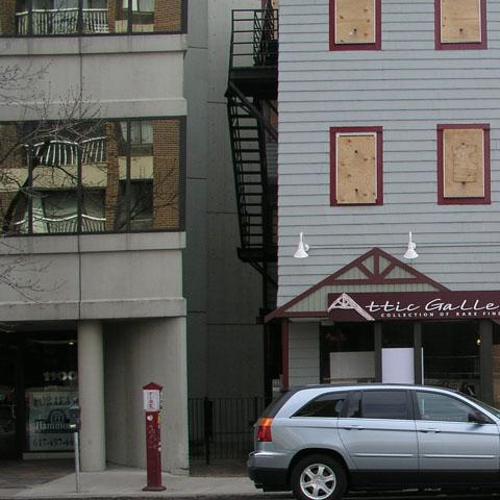

Supplement: Supplementary file 4 [file Presentation_4.zip › Non-targets_2/image_0839.jpg]

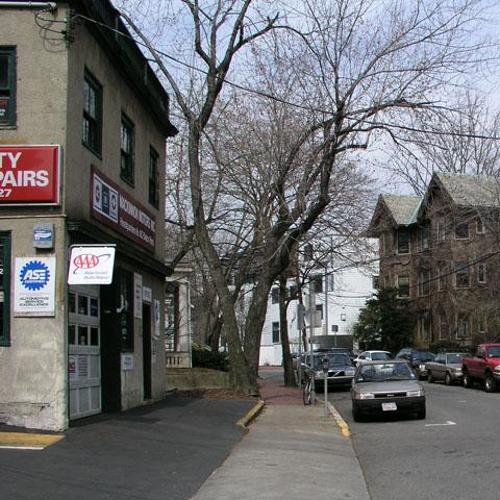

Supplement: Supplementary file 4 [file Presentation_4.zip › Non-targets_2/image_0840.jpg]

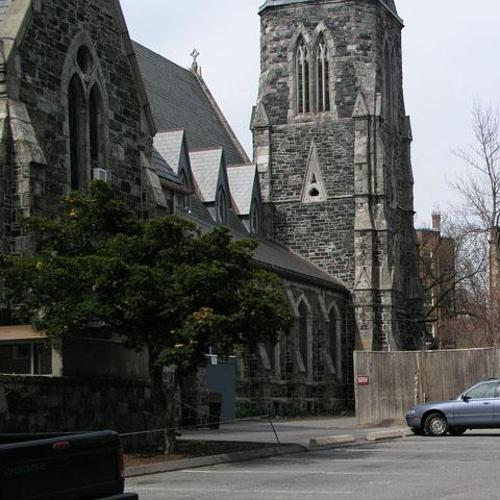

Supplement: Supplementary file 4 [file Presentation_4.zip › Non-targets_2/image_0841.jpg]

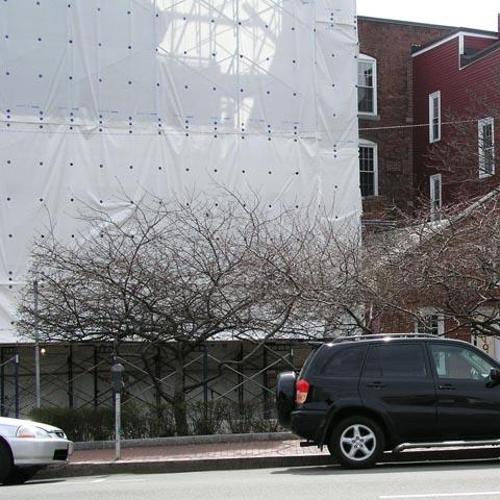

Supplement: Supplementary file 4 [file Presentation_4.zip › Non-targets_2/image_0842.jpg]

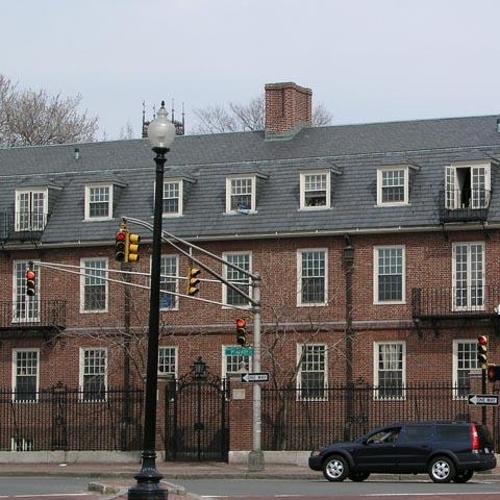

Supplement: Supplementary file 4 [file Presentation_4.zip › Non-targets_2/image_0843.jpg]

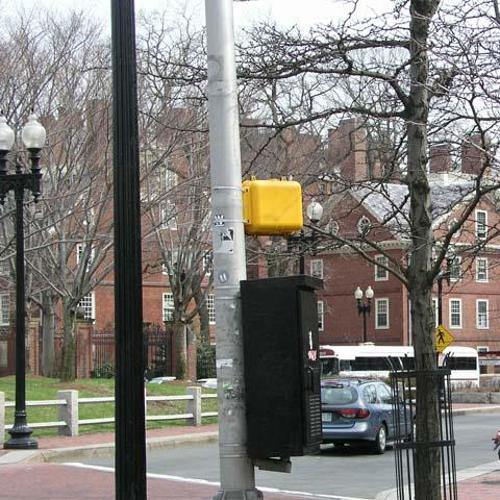

Supplement: Supplementary file 4 [file Presentation_4.zip › Non-targets_2/image_0844.jpg]

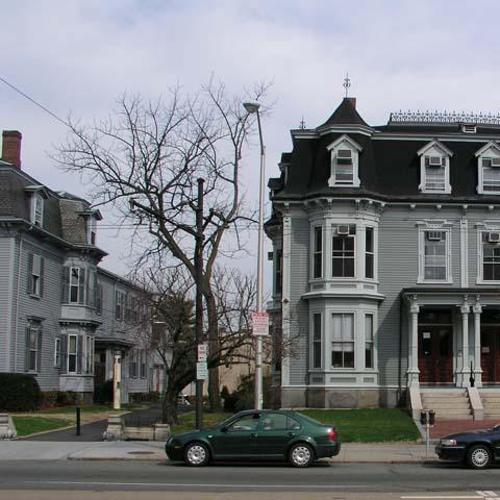

Supplement: Supplementary file 4 [file Presentation_4.zip › Non-targets_2/image_0845.jpg]

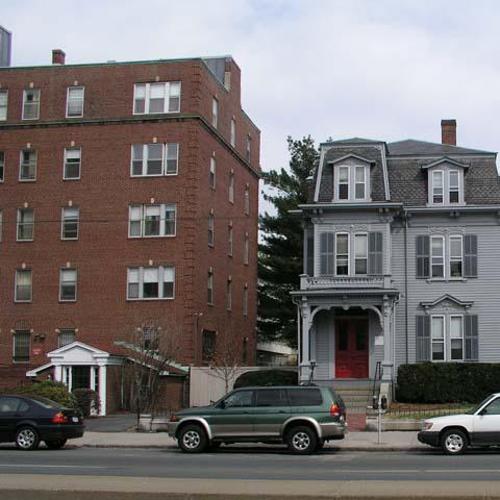

Supplement: Supplementary file 4 [file Presentation_4.zip › Non-targets_2/image_0846.jpg]
